# Monotherapy treatment of epilepsy in pregnancy: congenital malformation outcomes in the child

**DOI:** 10.1002/14651858.CD010224.pub3

**Published:** 2023-08-29

**Authors:** Rebecca Bromley, Naghme Adab, Matt Bluett-Duncan, Jill Clayton-Smith, Jakob Christensen, Katherine Edwards, Janette Greenhalgh, Ruaraidh A Hill, Cerian F Jackson, Sonia Khanom, Ronan N McGinty, Catrin Tudur Smith, Jennifer Pulman, Anthony G Marson

**Affiliations:** Division of NeuroscienceUniversity of ManchesterManchesterUK; Royal Manchester Children's HospitalManchesterUK; Department of Neurology, A5 CorridorWalsgrave Hospital, University Hospitals Coventry and Warwickshire NHS TrustCoventryUK; Institute of Human DevelopmentUniversity of ManchesterManchesterUK; Department of NeurologyAarhus University HospitalAarhusDenmark; Liverpool Reviews and Implementation GroupUniversity of LiverpoolLiverpoolUK; Liverpool Reviews and Implementation Group, Department of Health Data ScienceUniversity of LiverpoolLiverpoolUK; Department of Molecular and Clinical PharmacologyInstitute of Translational Medicine, University of LiverpoolLiverpoolUK; Nuffield Department of Clinical NeurosciencesJohn Radcliffe HospitalOxfordUK; Department of Health Data ScienceUniversity of LiverpoolLiverpoolUK; Department of Pharmacology and TherapeuticsInstitute of Systems, Molecular and Integrative Biology, University of LiverpoolLiverpoolUK

**Keywords:** Child, Female, Humans, Male, Pregnancy, Cohort Studies, Epilepsy, Epilepsy/drug therapy, Epilepsy/epidemiology, Lamotrigine, Phenytoin, Prenatal Exposure Delayed Effects, Prospective Studies, Topiramate

## Abstract

**Background:**

Prenatal exposure to certain anti‐seizure medications (ASMs) is associated with an increased risk of major congenital malformations (MCM). The majority of women with epilepsy continue taking ASMs throughout pregnancy and, therefore, information on the potential risks associated with ASM treatment is required.

**Objectives:**

To assess the effects of prenatal exposure to ASMs on the prevalence of MCM in the child.

**Search methods:**

For the latest update of this review, we searched the following databases on 17 February 2022: Cochrane Register of Studies (CRS Web), MEDLINE (Ovid, 1946 to February 16, 2022), SCOPUS (1823 onwards), and ClinicalTrials.gov, WHO International Clinical Trials Registry Platform (ICTRP). No language restrictions were imposed.

**Selection criteria:**

We included prospective cohort controlled studies, cohort studies set within pregnancy registries, randomised controlled trials and epidemiological studies using routine health record data. Participants were women with epilepsy taking ASMs; the two control groups were women without epilepsy and untreated women with epilepsy.

**Data collection and analysis:**

Five authors independently selected studies for inclusion. Eight authors completed data extraction and/or risk of bias assessments. The primary outcome was the presence of an MCM. Secondary outcomes included specific types of MCM. Where meta‐analysis was not possible, we reviewed included studies narratively.

**Main results:**

From 12,296 abstracts, we reviewed 283 full‐text publications which identified 49 studies with 128 publications between them. Data from ASM‐exposed pregnancies were more numerous for prospective cohort studies (n = 17,963), than data currently available for epidemiological health record studies (n = 7913). The MCM risk for children of women without epilepsy was 2.1% (95% CI 1.5 to 3.0) in cohort studies and 3.3% (95% CI 1.5 to 7.1) in health record studies.

The known risk associated with sodium valproate exposure was clear across comparisons with a pooled prevalence of 9.8% (95% CI 8.1 to 11.9) from cohort data and 9.7% (95% CI 7.1 to 13.4) from routine health record studies. This was elevated across almost all comparisons to other monotherapy ASMs, with the absolute risk differences ranging from 5% to 9%. Multiple studies found that the MCM risk is dose‐dependent. Children exposed to carbamazepine had an increased MCM prevalence in both cohort studies (4.7%, 95% CI 3.7 to 5.9) and routine health record studies (4.0%, 95% CI 2.9 to 5.4) which was significantly higher than that for the children born to women without epilepsy for both cohort (RR 2.30, 95% CI 1.47 to 3.59) and routine health record studies (RR 1.14, 95% CI 0.80 to 1.64); with similar significant results in comparison to the children of women with untreated epilepsy for both cohort studies (RR 1.44, 95% CI 1.05 to 1.96) and routine health record studies (RR 1.42, 95% CI 1.10 to 1.83).

For phenobarbital exposure, the prevalence was 6.3% (95% CI 4.8 to 8.3) and 8.8% (95% CI 0.0 to 9277.0) from cohort and routine health record data, respectively. This increased risk was significant in comparison to the children of women without epilepsy (RR 3.22, 95% CI 1.84 to 5.65) and those born to women with untreated epilepsy (RR 1.64, 95% CI 0.94 to 2.83) in cohort studies; data from routine health record studies was limited. For phenytoin exposure, the prevalence of MCM was elevated for cohort study data (5.4%, 95% CI 3.6 to 8.1) and routine health record data (6.8%, 95% CI 0.1 to 701.2). The prevalence of MCM was higher for phenytoin‐exposed children in comparison to children of women without epilepsy (RR 3.81, 95% CI 1.91 to 7.57) and the children of women with untreated epilepsy (RR 2.01. 95% CI 1.29 to 3.12); there were no data from routine health record studies.

Pooled data from cohort studies indicated a significantly increased MCM risk for children exposed to lamotrigine in comparison to children born to women without epilepsy (RR 1.99, 95% CI 1.16 to 3.39); with a risk difference (RD) indicating a 1% increased risk of MCM (RD 0.01. 95% CI 0.00 to 0.03). This was not replicated in the comparison to the children of women with untreated epilepsy (RR 1.04, 95% CI 0.66 to 1.63), which contained the largest group of lamotrigine‐exposed children (> 2700). Further, a non‐significant difference was also found both in comparison to the children of women without epilepsy (RR 1.19, 95% CI 0.86 to 1.64) and children born to women with untreated epilepsy (RR 1.00, 95% CI 0.79 to 1.28) from routine data studies. For levetiracetam exposure, pooled data provided similar risk ratios to women without epilepsy in cohort (RR 2.20, 95% CI 0.98 to 4.93) and routine health record studies (RR 0.67, 95% CI 0.17 to 2.66). This was supported by the pooled results from both cohort (RR 0.71, 95% CI 0.39 to 1.28) and routine health record studies (RR 0.82, 95% CI 0.39 to 1.71) when comparisons were made to the offspring of women with untreated epilepsy. For topiramate, the prevalence of MCM was 3.9% (95% CI 2.3 to 6.5) from cohort study data and 4.1% (0.0 to 27,050.1) from routine health record studies. Risk ratios were significantly higher for children exposed to topiramate in comparison to the children of women without epilepsy in cohort studies (RR 4.07, 95% CI 1.64 to 10.14) but not in a smaller comparison to the children of women with untreated epilepsy (RR 1.37, 95% CI 0.57 to 3.27); few data are currently available from routine health record studies. Exposure in utero to topiramate was also associated with significantly higher RRs in comparison to other ASMs for oro‐facial clefts. Data for all other ASMs were extremely limited.

Given the observational designs, all studies were at high risk of certain biases, but the biases observed across primary data collection studies and secondary use of routine health records were different and were, in part, complementary. Biases were balanced across the ASMs investigated, and it is unlikely that the differential results observed across the ASMs are solely explained by these biases.

**Authors' conclusions:**

Exposure in the womb to certain ASMs was associated with an increased risk of certain MCMs which, for many, is dose‐dependent.

## Plain language summary

**Treatment for epilepsy in pregnant women and the physical health of the child**

**Background**

For most women who have epilepsy, continuing their medication during pregnancy is important for their health. Over the last 40 years, research has shown that children exposed to anti‐seizure medications in the womb can be at a higher risk of having a malformation or birth defect.

**Research question**

This review aimed to understand whether exposure to anti‐seizure medication during pregnancy is linked to an increased risk of having a child with a major structural congenital malformation (also known as a birth defect).

**Characteristics of the studies**

The review included 49 published studies which included over 25,000 pregnancies where ASMs were used. We compared the children of women with epilepsy who were taking a single anti‐seizure medication to the children of women without epilepsy or women who had epilepsy but who were not being treated with anti‐seizure medications. We also made comparisons between children exposed to different anti‐seizure medications in the womb. The evidence presented in this review is up‐to‐date as of February 2022.

**Results**

The amount of data available from the studies reviewed varied greatly depending on the type of anti‐seizure medication used, and this could account for some findings.

The rate of malformations in children born to women without epilepsy was between 2.1% and 3.3% and, for children born to women with an untreated epilepsy, this rate was between 3.0% and 3.2%. Therefore, we consider that the background risk of being born with a malformation is between 2% and 3%. Overall, the data did not show a higher rate of malformation in infants exposed to either lamotrigine (2.7% to 3.5%) or levetiracetam (2.6% to 2.8%). However, in one well‐designed study, higher doses of lamotrigine were linked to a higher risk of malformations. There were fewer data regarding oxcarbazepine exposure but, based on current experience, there is not a significant increase of malformations in exposed infants (2.8% to 4.8%).

Children exposed to sodium valproate were at the highest risk of having a malformation with 9.7% to 9.8% of exposed children having one or more malformation(s). Specifically, risks were higher for spinal, skeletal, cardiac and facial malformations. The level of the risk was associated with the dose of the valproate taken; higher doses of valproate were linked to higher rates of malformation. The risk associated with valproate exposure was higher than that seen for other ASM exposures, including those with a higher risk themselves (for example, topiramate or phenobarbital).

Children exposed to phenobarbital had a higher rate of malformation with 6.3% to 8.8% of children being born with a malformation. This was higher than certain groups not exposed to anti‐seizure medications and children born exposed to other anti‐seizure medications. However, the risk was lower than that associated with valproate. Children exposed to phenobarbital were specially at risk of cardiac malformations.

Children exposed to phenytoin had a higher rate of malformation with 5.4% to 6.8% of children being born with a malformation. This risk was higher than unexposed children and children exposed to certain other anti‐seizure medications. Data were too few to understand which specific types of malformation were most likely to occur following exposure in the womb to phenytoin.

Children exposed to carbamazepine had a higher rate of malformation with 4.0% to 4.7% of children being born with a malformation. This was higher than unexposed children and children exposed to other anti‐seizure medications. The risk of malformation was found to increase at higher doses of carbamazepine.

There were fewer pregnancies in women exposed to topiramate, but a higher rate of malformation was noted with 3.9% to 4.1% of exposed children having a malformation. This was higher than in children born to women without epilepsy. The data demonstrated that children exposed to topiramate were at particular risk of facial malformations.

The data were too limited for other anti‐seizure medications to be certain about their results at this time.

**Quality of the studies**

The quality of included studies varied, but we do not consider that this accounts for the results of the review where we see different levels of risk associated with different anti‐seizure medications.

**Conclusions**

This review found that children exposed to certain anti‐seizure medications in the womb were at an increased risk of having a major malformation at birth and that the level of risk is determined, in most cases, by the dose of the medication child is exposed to. Levetiracetam and lamotrigine appear to be the anti‐seizure medications associated with the lowest level of risk, but more data are needed, particularly concerning individual types of malformation and higher doses. For many of the antiseizure medications considered in this review, there were too little data to reach conclusions.

## Summary of findings

**Summary of findings 1:** Summary of findings ‐ Lamotrigine

**Monotherapy treatment of epilepsy in pregnancy: congenital malformation outcomes in the child**					
Population: Pregnant women with epilepsy Intervention: ASM monotherapy Comparison: Lamotrigine in comparison to other ASMs Outcome: Major congenital malformation rate in the exposed children					
**Comparisons**		**Illustrative comparative risks across data types**		**Relative effect** **(95% CI)**	**N of participants (studies)**
		**Prevalence LTG** **(95% CI)**	**Prevalence** **comparator** **(95% CI)**		
Lamotrigine vs no medication (women without epilepsy)	Cohort studies	LTG 2.7% (1.9, 3.8)	No Med 2.1% (1.5, 3.0)	1.99 (1.16, 3.39)	4862 (7)
	Database studies	LTG 3.5% (2.5, 4.9)	No Med 3.3% (1.5, 7.1)	1.19 (0.86, 1.64)	373,288 (2)
Lamotrigine vs no medication (women with epilepsy)	Cohort studies	LTG 2.7% (1.9, 3.8)	No Med 3.0% (2.1, 4.2)	1.04 (0.66, 1.63)	3918 (8)
	Database studies	LTG 3.5% (2.5, 4.9)	No Med 3.2% (1.7, 6.1)	1.00 (0.79, 1.28)	13,445 (3)
Levetiracetam vs lamotrigine	Cohort studies	LTG 2.7% (1.9, 3.8)	LEV 2.6% (1.6, 4.4)	0.90 (0.58, 1.39)	5612 (10)
	Database studies	LTG 3.5% (2.5, 4.9)	LEV 2.8% (0.0, 321.9)	0.79 (0.37, 1.69)	2316 (2)
	EURAP	LTG 2.9% (2.3, 3.7)	LEV 2.8% (1.7, 4.5)	N/A	3113
Carbamazepine vs lamotrigine	Cohort studies	LTG 2.7% (1.9, 3.8)	CBZ 4.7% (3.7, 5.9)	1.37 (1.06, 1.77)	8568 (13)
	Database studies	LTG 3.5% (2.5, 4.9)	CBZ 4.0% (2.9, 5.4)	1.21 (0.88, 1.67)	4503 (4)
	EURAP	LTG 2.9% (2.3, 3.7)	LTG 5.5% (4.5, 6.6)	N/A	4471
Lamotrigine vs topiramate	Cohort studies	LTG 2.7% (1.9, 3.8)	TPM 3.9% (2.3, 6.5)	0.59 (0.36, 0.96)^a^	4780 (8)
	Database studies	LTG 3.5% (2.5, 4.9)	TPM 4.1% (0.0, 270.6)	0.68 (0.20, 2.37)	972 (2)
	EURAP	LTG 2.9% (2.3, 3.7)	TPM 3.9% (1.5, 8.4)	N/A	2666
Valproate vs lamotrigine	Cohort studies	LTG 2.7% (1.9, 3.8)	VPA 9.8% (8.1, 11.9)	3.50 (2.76, 4.46)	6896 (12)
	Database studies	LTG 3.5% (2.5, 4.9)	VPA 9.7% (7.1, 13.4)	2.49 (1.86, 3.35)	3590 (4)
	EURAP	LTG 2.9% (2.3, 3.7)	VPA 10.3% (8.8, 12.0)	N/A	3895
Lamotrigine vs oxcarbazepine	Cohort studies	LTG 2.7% (1.9, 3.8)	OXC 2.8% (1.1, 6.6)	0.73 (0.33, 1.62)	2541 (8)
	Database studies	LTG 3.5% (2.5, 4.9)	OXC 4.8% (0.7, 31.5)	1.24 (0.67, 2.30)	2535 (3)
	EURAP	LTG 2.9% (2.3, 3.7)	OXC 3.0% (1.4, 5.4)	N/A	2847
Lamotrigine vs zonisamide	Cohort studies	LTG 2.7% (1.9, 3.8)	ZNS 2.7% (0.1, 47.3)	0.66 (0.26, 1.65)^b^	3922 (4)
	Database studies	LTG 3.5% (2.5, 4.9)	N/A	N/A	N/A
	EURAP	LTG 2.9% (2.3, 3.7)	N/A	N/A	N/A

^a^ RD was non‐significant; ^b^ Random‐effects RR was calculated due to heterogeneity. ASM: Anti‐Seizure Medication  CBZ: Carbamazepine CI: Confidence Interval LEV: Levetiracetam  LTG: Lamotrigine MED: Medication N/A: not available OXC: Oxcarbazepine TPM: Topiramate VPA: Sodium Valproate

**Summary of findings 2:** Summary of findings ‐ Levetiracetam

**Monotherapy treatment of epilepsy in pregnancy: congenital malformation outcomes in the child**					
Population: Pregnant women with epilepsy Intervention: ASM monotherapy Comparison: Levetiracetam in comparison to other ASMs Outcome: Major congenital malformation rate in the exposed children					
**Comparison**		**Illustrative comparative risks across data types**		**Relative effect (95% CI)**	**N of participants (studies)**
		**Prevalence LEV (95% CI)**	**Prevalence** **comparator (95% CI)**		
Levetiracetam vs no medication (women without epilepsy)	Cohort studies	LEV 2.6% (1.6, 4.4)	2.1% (1.5, 3.0)	2.20 (0.98, 4.93)	1596 (4)
	Database studies	LEV 2.8% (0.0, 321.9)	3.3% (1.5, 7.1)	0.67 (0,17, 2.66)	369,385 (1)
Levetiracetam vs no medication (women with epilepsy)	Cohort studies	LEV 2.6% (1.6, 4.4)	3.0% (2.1, 4.2)	0.71 (0.39, 1.28)	1825 (6)
	Database studies	LEV 2.8% (0.0, 321.9)	3.2% (1.7, 6.1)	0.82 (0.39, 1.71)	10,625 (2)
Levetiracetam vs lamotrigine	Cohort studies	LEV 2.6% (1.6, 4.4)	LTG 2.7% (1.9, 3.8)	0.90 (0.58‐ 1.39)	5612 (10)
	Database studies	LEV 2.8% (0.0, 321.9)	LTG 3.5% (2.5, 4.9)	0.79 (0.37, 1.69)	2316 (2)
	EURAP	LEV 2.8% (1.7, 4.5)	LTG 2.9% (2.3, 3.7)	N/A	3113
Carbamazepine vs levetiracetam	Cohort studies	LEV 2.6% (1.6, 4.4)	CBZ 4.7% (3.7, 5.9)	1.51 (1.01, 2.26)	5056 (11)
	Database studies	LEV 2.8% (0.0, 321.9)	CBZ 4.0% (2.9, 5.4)	1.73 (0.78, 3.83)	1248 (2)
	EURAP	LEV 2.8% (1.7, 4.5)	5.5% (4.5, 6.6)	N/A	2556
Levetiracetam vs topiramate	Cohort studies	LEV 2.6% (1.6, 4.4)	TPM 3.9% (2.3, 6.5)	0.57 (0.32, 1.04)	1629 (8)
	Database studies	LEV 2.8% (0.0, 321.9)	TPM 4.1% (0.0, 27060.0)	0.41 (0.06, 2.81)	166 (1)
	EURAP	LEV 2.8% (1.7, 4.5)	TPM 3.9% (1.5, 8.4)	N/A	751
Valproate vs levetiracetam	Cohort studies	LEV 2.6% (1.6, 4.4)	VPA 9.8% (8.1, 11.9)	3.77 (2.48, 5.74)	3485 (10)
	Database studies	LEV 2.8% (0.0, 321.9)	VPA 9.7% (7.1, 13.4)	3.26 (1.51, 7.03)	911 (2)
	EURAP	LEV 2.8% (1.7, 4.5)	VPA 10.3% (8.8, 12.0)	N/A	1980
Levetiracetam vs oxcarbazepine	Cohort studies	LEV 2.6% (1.6, 4.4)	OXC 2.8% (1.1, 6.6)	1.04 (0.51, 2.09)	1166 (8)
	Database studies	LEV 2.8% (0.0, 321.9)	OXC 4.8% (0.7, 31.5)	1.17 (0.45, 3.06)	621 (2)
	EURAP	LEV 2.8% (1.7, 4.5)	OXC 3.0% (1.4, 5.4)	N/A	932
Levetiracetam vs zonisamide	Cohort studies	LEV 2.6% (1.6, 4.4)	2.7% (0.1, 47.3)	0.66 (0.25, 1.71)^a^	995 (4)
	Database studies	LEV 2.8% (0.0, 321.9)	N/A	N/A	N/A
	EURAP	LEV 2.8% (1.7, 4.5)	N/A	N/A	N/A

^a^ RD was non‐significant; ^b^ Random‐effects RR was calculated due to heterogeneity. ASM: Anti‐Seizure Medication  CBZ: Carbamazepine CI: Confidence Interval LEV: Levetiracetam  LTG: Lamotrigine N/A: Not Available OXC: Oxcarbazepine TPM: Topiramate VPA: Sodium Valproate

## Background

This review is an update of the Cochrane Review first published in 2004 (Adab 2004), and last updated in 2016 (Weston 2016).

### Description of the condition

Epilepsy is a common neurological disorder with a lifetime prevalence of 7.60 per 1000 persons (Fiest 2017). A significant number of women with epilepsy will be in their childbearing years (NICE 2022) and, of these, approximately 0.5% to 0.6% of all annual pregnancies are reportedly exposed to an anti‐seizure medication (ASM) in utero (Man 2012, NICE 2022). ASM treatment of epilepsy in the childbearing years requires careful optimisation to improve maternal outcomes whilst minimising, where possible, foetal risks. Research demonstrates an association between children born to women with epilepsy treated with ASMs and an increased risk of major congenital malformations, including cardiac, neural tube and craniofacial defects (EURAP 2018; Jentink 2010a; Meador 2008).

### Description of the intervention

ASMs are the most common treatment for epilepsy, and most women with epilepsy require treatment continuation during pregnancy.

### How the intervention might work

ASMs readily cross the placenta from the mother into the foetusus (Brent 2004; Tetro 2017). Prospective observational studies (e.g. Milan Study 1999), registry‐based studies (e.g. Tomson 2011), case‐control studies (Jentink 2010a), and epidemiological studies using datasets of routine health records (e.g. Denmark Health Record Registers) provide evidence of an association between ASM treatment and an increased prevalence of major congential malformations. The level of risk varies for different types of ASM, with first trimester valproate (VPA) exposure associated with the largest increase in prevalence (EURAP 2018; Meador 2006; Milan Study 1999; North American Epilepsy and Pregnancy Register; UK and Ireland Epilepsy and Pregnancy Register). The mechanisms through which prenatal exposure to ASMs are associated with an increased prevalence of major malformations likely differs by treatment type and may be multifactorial.

This review investigates the outcomes for monotherapy treatment with different ASMs to identify currently available evidence on which to base treatment decisions.

### Why it is important to do this review

The decision to continue ASM treatment during pregnancy requires taking a risk‐benefit decision. On the one hand, there is the potential risk posed to the foetus when the medication is a teratogen yet on the other hand, there is the health and well‐being of the mother, who requires treatment throughout her pregnancy to minimise the risk of seizures (Tomson 2015); the choice of ASM depends on the type of epilepsy and the seizures (Marson 2007). A lack of knowledge regarding foetal safety limits treatment options for women with epilepsy in their childbearing years, as women and their doctors may avoid ASMs with limited data. Conversely, a lack of evidence may lead to an ASM with a higher foetal risk profile being used extensively, prior to a full understanding of its risks.

While a number of studies indicate a teratogenic risk from certain ASMs, there are conflicting results regarding the degree of risk and the types of malformations associated with specific ASMs. Data are slow to accumulate and an earlier version of this review (Weston 2016) found extremely limited data on ASMs with a decade or more of clinical use. Such a lack of evidence makes it difficult to counsel women about treatment choices before or during pregnancy. There is, therefore, a clear need for a systematic review and meta‐analysis of existing data to inform these decisions. Randomised controlled trials (RCTs) would provide the most reliable evidence about the effects of ASMs in pregnancy, but are essentially precluded by ethical considerations and logistical challenges pertaining to study design, recruitment and interpretation.

In view of this, we performed a systematic review of all available evidence including registry‐based, prospective cohort studies, RCTs and epidemiological studies using routine health record databases. At the protocol stage, we decided not to include malformation case‐control studies (e.g. Jentink 2010a; Jentink 2010b) due to the substantial differences in the approach in these studies and how these methods compare to prospective observational cohort studies. This decision is discussed further in Overall completeness and applicability of evidence. This review is an update of two previous reviews (Adab 2004; Weston 2016). Evidence from this review, along with the related review by the same Cochrane team (Bromley 2014), will aid the decisions that clinicians and women with epilepsy have to make about the treatment of epilepsy during the potential childbearing years.

## Objectives

To assess the effects of prenatal exposure to commonly prescribed ASMs on the prevalence of major congenital malformations in the child.

This review examines the association between specific ASM exposures and the prevalence of major congenital malformations compared to the general population or unexposed pregnancies in women with epilepsy. It also compares the prevalence of specific major congenital malformations types across the ASM treatment groups.

## Methods

### Criteria for considering studies for this review

#### Types of studies

We considered the following types of studies.

1. Randomised controlled trials (RCTs). These studies included women with epilepsy who were randomised to a particular ASM prior to conception. The intervention group(s) comprised women with epilepsy taking ASM monotherapy.
2. Prospective observational cohort studies. These included consecutive participants whose clinical information was collected prior to the birth of the child. The intervention group(s) comprised women with epilepsy treated with ASM monotherapy.
3. Registry studies. These involve the collection of data from a wide region, country or number of countries, and recruitment is often based on self‐referral or clinician‐referral, leading to non‐sequential case ascertainment. We considered both disease‐based registries (e.g. pregnancy and epilepsy registries) and industry‐sponsored product registry datasets. Pregnant women with epilepsy prescribed ASM monotherapy were recruited prospectively prior to childbirth.
4. Population‐based routine health record datasets. These studies utilise data collected for routine health monitoring, administrative or reimbursement reasons for entire national populations or specific populations (e.g. medical insurance databases). Individual recruitment of participants is not required. The intervention group(s) comprised women with epilepsy taking ASM monotherapy.

#### Types of participants

Pregnant women with epilepsy taking a single ASM of interest were eligible for the intervention group.

Participants eligible for the comparator groups were:

- pregnant women with epilepsy taking an ASM;
- pregnant women with epilepsy taking no ASM; or
- pregnant women who do not have epilepsy.

We excluded studies reporting ASM use solely in pregnant women with other conditions (e.g. mood disorders, pain). We included studies involving women taking ASMs for epilepsy and other conditions if the non‐epilepsy conditions accounted for 30% or less of the total treatment group. This percentage criterion was increased from the previous review to accommodate data from population healthcare datasets, which often include a wider group of participant indications.

#### Types of interventions

##### Intervention group

Women with epilepsy who received any of the following ASMs as monotherapy: acetazolmide, brivaracetam, bromide, carbamazepine, cenobamate, clomethiazole, clonazepam, clorazepate, diazepam, dimethyloxazolidinedione, eslicarbazepine, ethosuximide, estazolam, felbamate, flunarizine, gabapentin, lacosamide, lamotrigine, levetiracetam, lorazepam, magnesium sulphate, medazepam, methylphenobarbital, mephenytoin, meprobamate, methazolamide, methsuximide, methyloxazepam, midazolam, nimetazepam, nitrazepam, oxcarbazepine, perampanel, phenobarbitone, phenytoin, primidone, pregabalin, remacemide, retigabine, rufinamide, sodium valproate, stiripentol, sulthiame, tiagabine, topiramate, trimethadione, trifluoromethoxy benzothiazole, valnoctamide, vigabatrin, or zonisamide.

##### Comparator groups

We used two separate types of comparator groups in this review, as currently there is no clear evidence regarding the reliability of combining data from these two different groups. The two comparator groups are:

- controls: women with a diagnosis of epilepsy who were not taking ASMs and women without epilepsy.
- comparator treatment: women with epilepsy treated with ASM monotherapy, evaluated in subgroup analyses to enable treatment comparisons.

#### Types of outcome measures

##### Primary outcomes

###### Major congenital malformations

The proportion of children who present with any type of major congenital malformation (as defined by study authors). Major malformations are structural abnormalities of the body or organs present from birth and which require intervention (e.g., corrective surgery) or have a significant level of impact on the child's daily functioning (EUROCAT).

##### Secondary outcomes

###### Specific major congenital malformations

The proportion of children who present with the following specific major congenital malformations by area of the body.

- Neural tube malformations.
- Cardiac malformations.
- Oro‐facial cleft/craniofacial malformation.
- Skeletal or limb malformations.

We chose the above disorders because they are important major malformations associated with exposure to ASMs in utero, because these are the most prevalent congenital malformations in the general population (ref: https://eu‐rd‐platform.jrc.ec.europa.eu/eurocat/eurocat‐data/prevalence_en), and because of the availability of data within the included studies. When extracting data from included studies, we compiled a list of all the specified malformations. Author JCS, a clinical geneticist, then reviewed the list and classified the items into one of the four specific malformation categories.

### Search methods for identification of studies

#### Electronic searches

Searches for the original review were run in January 2012. Subsequent searches were run in March 2013, May 2014, and September 2015. For the latest update, we searched the following databases on 17 February 2022:

1. Cochrane Register of Studies (CRS Web), using the search strategy set out in Appendix 1;
2. MEDLINE (Ovid, 1946 to February 16, 2022) using the search strategy set out in Appendix 2;
3. SCOPUS (1823 onwards) using the search strategy set out in Appendix 3;
4. ClinicalTrials.gov using the search strategy set out in Appendix 4;
5. WHO International Clinical Trials Registry Platform (ICTRP) using the search strategy set out in Appendix 5.

CRS Web includes randomised or quasi‐randomised, controlled trials from PubMed, Embase, ClinicalTrials.gov, the World Health Organization International Clinical Trials Registry Platform (ICTRP), the Cochrane Central Register of Controlled Trials (CENTRAL), and the Specialized Registers of Cochrane Review Groups including Epilepsy. In MEDLINE (Ovid), the coverage end date always lags a few days behind the search date. Previously we also searched Embase, Pharmline and Reprotox.

We did not impose any language restrictions in the search and, when necessary, we obtained translations of articles written in languages other than English.

#### Searching other resources

We reviewed conference abstracts from neurology meetings published from 2010 to 2022, including abstracts from the International League Against Epilepsy meetings (American Epilepsy Society, International Epilepsy Congress, European Congress on Epileptology, Asian and Oceanian Epilepsy Congress and Latin American Congress on Epilepsy) and Teratology meetings (Teratology Society and European Teratology Society). Where possible, we linked abstracts to published datasets or categorised them as awaiting classification.

We cross‐matched reference lists of original research and review articles to the studies generated from the electronic searches. We handsearched reference lists of recent review articles and contacted lead and corresponding authors in the area for any relevant unpublished material.

### Data collection and analysis

#### Selection of studies

Five authors (RB, JW, JG, KE, RMcG) reviewed the titles and abstracts of articles highlighted by the searches and removed studies that obviously did not meet the inclusion criteria. Four authors (RB, JW, KE, RMcG) used full‐text reports to determine study eligibility. We discussed disagreements and sought the opinion of a third author (JG, CJ, RB), when necessary. Multiple reports from single studies are common in this field. To ensure that each cohort was represented only once in our analysis, therefore to avoid double‐counting the population across papers of included studies, we linked studies by recruitment date and sought confirmation from authors whether reports referred to single study populations. Where this was unclear, we contacted study authors for clarification.

#### Data extraction and management

Eight authors (RB, JW, NA, JG, AM, KE, RMcG, SK, CJ) undertook data extraction of the included studies. We used pre‐standardised electronic data extraction forms that members of the review team piloted and then amended, where necessary. We then cross‐checked data extraction. All entries into RevMan were also double‐checked.

#### Assessment of risk of bias in included studies

Due to the observational design of the majority of the studies, we utilised the Risk Of Bias In Non‐Randomized Studies ‐ of Interventions (ROBINS‐I) tool which the Cochrane Non‐Randomised Studies Methods Group has developed (Sterne 2016). The ROBINS‐I tool for assessing risk of bias examines bias in the domains of confounding, selection, treatment classification, missing data, measurement and reported results. ROBINS‐I uses signalling questions on a four‐point scale to determine level of bias in specific elements of biases for each of these domains. Overall domain bias ratings are then classed as low, moderate, serious, critical or no information.

ROBINS‐I was developed for treatment studies and not pharmacovigilance studies, where the person taking the medication (the mother) is not the same person in which the outcome can occur (the child). Therefore, ROBINS‐I needed to be adapted for use in this review. The adaption was led by author RB with input from other authors. Important confounder and mediator variables were selected based on published evidence of an association both in the general population and specifically in investigations regarding in utero ASM exposure and congential malformation outcomes. See Appendix 6 for further information. Eight authors completed risk of bias ratings (RB, JW, NA, JG, AM, KE, SK, MBD). Each included study was reviewed by two independent raters and the opinion of a third author (RB) was sought where there were disagreements in the domain level ratings. For RCTs, we intended to use the original Cochrane tool for assessing risk of bias (RoB1) (Higgins 2011).

We intended, where applicable, to create Summary of findings tables for outcomes and to grade each outcome accordingly using the GRADE (Grading of Recommendations Assessment, Development and Evaluation) approach (Guyatt 2008). However, we found GRADE to not be optimised for these types of data and using it would have led to differential ratings across comparisons, depending on whether there was a difference in MCM rate or not; thus, producing ratings of lower evidence confidence for comparisons with no difference between the ASMs. Further work is required on GRADE and ROBINS‐I to optimise them for pregnancy pharmacovigilance investigations.

#### Measures of treatment effect

We considered that different study design types or comparator groups may lead to different outcome results and, therefore, we did not combine all data into a single meta‐analysis containing mixed study types, groups of different ASMs and comparator groups. Meta‐analyses were instead stratified by study type, by comparator group (e.g. women with epilepsy untreated and women without epilepsy and with no treatment), and by ASM versus ASM comparison. We computed pooled prevalences of malformations within AED (antiepileptic drug) groups (using fixed‐effect models, unless otherwise stated) and reported them at the beginning of each drug section. The primary and secondary outcomes are presented as risk ratios (RRs). We also computed risk differences (RDs) using Review Manager (RevMan) to take into account studies with no reported events. We calculated these effect estimates in accordance with the *Cochrane Handbook for Systematic Reviews of Interventions* and reported them in the results section (Higgins 2011). Where treatment effects were reported from individual studies, we used the summary effect measure that had been utilised by the study authors to report results from the study. In some cases, OR instead of RR was reported by individual study authors.

The RR is a measure of relative effect expressed as the ratio of the risk of an event in the two groups. If the 95% confidence interval includes the value of 1.00, this implies there is no difference between the groups (i.e. a non‐significant result). If the value of 1.00 lies outside the 95% confidence interval, this implies there is a difference between the groups (i.e. a significant result). The RD is a measure of absolute effect expressed as the difference of the risk of an event in the two groups. If the 95% confidence interval contains the value of 0.00, this implies there is no difference between the groups (i.e. both groups have the same risk). If the value of 0.00 lies outside the 95% confidence interval, this implies there is a difference between the groups (i.e. a significant result). The significance of the RR and RD may be different, as the RD takes into account comparisons where there were no events in either arm, whilst the other does not. Although the RR estimates are large in many comparisons, the corresponding risk difference estimates are fairly small; but even a small increase in risk for a specific major malformation is clinically meaningful. In these cases, it would be up to the patient/clinician to interpret these risk estimates in the context of the adverse outcome and in relation to the potential benefits of treatment (e.g. seizure control). We did not account for multiple testing and the totality of the evidence for a particular exposure should be considered rather than the outcomes of a single comparison. Finally, we did not carry out any formal analysis of a dose‐response relationship. We have taken any dose‐response results reported directly from the study papers.

#### Unit of analysis issues

Data published in studies are often duplicated as they are updated, particularly in the case of the prospective pregnancy registries, which update their publications as the numbers of enrolled pregnancies increase. In such cases, we considered the latest time point as the 'primary' study for inclusion. In some cohorts, this meant that we used different publications for different ASMs. Further, there are studies that report combined data from a number of different registers (e.g. EURAP 2018; Samren 1997) which also report independently and routine health record studies with cohort overlap (e.g. UK Clinical Research Practice Database; UK Health Record THIN Register). Where the combined data reported provided greater numbers for a particular ASM comparison, it was included in the meta‐analysis but, where individual initiatives had greater numbers for a specific comparison (e.g. ASM vs control group), we included the individual study data and provided a narrative report of the collaborative initiatives. We carefully examined data to ensure that we did not include them more than once in the analysis and that we did not omit any non‐duplicated data.

#### Dealing with missing data

We contacted study authors to obtain missing statistics from included studies to input into the meta‐analysis. We also investigated study reasons for missing data to determine if they were missing at random or not.

#### Assessment of heterogeneity

We assessed clinical heterogeneity by examining the differences in study characteristics in order to inform decisions regarding the combination of study data in meta‐analysis. A priori hypotheses of sources of clinical heterogeneity included: type of population (regional, national or international, single or multicentre), loss to follow‐up, maternal factors including age, duration of ASM treatment, family history of congenital malformation, lifestyle factors, monotherapy, socioeconomic status, type of epilepsy, use of other medications and years of education. Child factors included: age of assessment, sex, seizure exposure, length of follow‐up and outcome measurement.

Where applicable, we also assessed statistical heterogeneity by examining the I^2^ statistic and a Chi^2^ test, using the guidelines outlined in Higgins 2011 for interpreting the results. According to these guidelines, an I^2^ statistic of 0% to 40% may not be important, 30% to 60% may indicate moderate heterogeneity, 50% to 90% may indicate substantial heterogeneity and 75% to 100% may indicate considerable heterogeneity. Therefore, for this review, we considered an I^2^ statistic of more than 50% to indicate significant heterogeneity. The I^2^ statistic was not applicable in comparisons where there was only a single study or when only one study contributed data to the analysis. When interpreting the Chi^2^ test, a P value of less than 0.01 was considered to indicate significant heterogeneity. When we found statistical heterogeneity, we presented both fixed‐effect and random‐effects analyses to enable exploration of differences.

#### Assessment of reporting biases

We included studies using the Outcome Reporting Bias in Trials (ORBIT) classification system if we suspected selective outcome reporting bias. We requested all protocols from included study authors to enable comparison of outcomes of interest; however, we received very few responses, complicating our performance of this comparison.

Our comprehensive search of multiple sources and data types, together with our requests for unpublished data or clarification from authors, minimised the risk of publication bias.

#### Data synthesis

We employed both fixed‐effect and random‐effects meta‐analyses to synthesise the data. We presented the primary outcome (major congenital malformations) and the secondary outcome of specific malformations as a risk ratio (RR). Within certain comparisons, we have also presented the risk differences (RD) for both primary outcome (overall malformation rate). In the event that we deemed meta‐analysing inappropriate (e.g. presence of clinical heterogeneity), we applied a narrative form to the review, discussing all comparisons according to the findings presented within the studies.

Comparisons carried out included:

1. specific ASM monotherapy group versus controls on major congenital malformations;
2. specific ASM monotherapy group versus controls on specific major congential malformation types;
3. specific ASM monotherapy group versus specific ASM monotherapy group on major congential malformations;
4. specific ASM monotherapy group versus specific ASM monotherapy group on specific major congential malformations.

We stratified each comparison by control group, comparator group and study design to ensure appropriate combination of study data. For example, cases reported in a national pregnancy and epilepsy register may also be represented in epidemiological datasets of routine health data which covers the same region or a case in an administrative insurance database may also have been reported to a national epilepsy and pregnancy register and therefore data were not combined across these different data sources.

#### Subgroup analysis and investigation of heterogeneity

Subgroup analysis was stratified by ASM and type of control or comparator group. When heterogeneity was present across outcomes, we carried out a random‐effects analysis. We examined differences between analyses and reported the appropriate analysis.

#### Sensitivity analysis

We adopted a cautious approach to combining data extracted from different types of study, and also where different comparator groups were included as outlined in Measures of treatment effect. Additionally, we only included studies where over 70% of the cohort were women taking ASMs for the treatment of epilepsy. This was due to the heterogeneity around doses prescribed, across women taking ASMs for different conditions. This decision is supported by the findings of Hernandez Diaz and colleagues (US Medicaid Registers) who found that differences in the dose of topiramate prescribed for women with epilepsy compared to women prescribed it for other conditions altered the risk of oro‐facial anomalies.

#### Summary of findings and assessment of the certainty of the evidence

In this review, we considered ASM use in during pregnancy in women with epilepsy and the major malformation rate in their exposed children (Figure 1). Comparisons were made across the different ASM treatments and to unexposed children. The outcomes are summarised in Table 3 along with Table 1, Table 2 for lamotrigine and levetiracetam and in Table 4, Table 5, Table 6, and Table 7 for carbamazepine, oxcarbazepine, topiramate and valproate, respectively. The data for other ASMs were too limited at this time for useful tables to be compiled. Relative risks and risk differences are displayed in Table 8 and Table 9.

**1:**
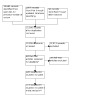
PRISMA flow diagram *50 studies were included in the original review but, due to changes to the inclusion criteria, 16 studies were excluded. ** for some studies only certain data were able to be included in the meta‐analysis.

**1:** Prevalence of major congenital malformations for each monotherapy ASM stratified by study type

	**Cohort**				**Database**				**All**			
**ASM**	**Total**	**Percentage**	**Lower 95% CI**	**Upper 95% CI**	**Total**	**Percentage**	**Lower 95% CI**	**Upper 95% CI**	**Total**	**Percentage**	**Lower 95% CI**	**Upper 95% CI**
CBZ	5415	4.7	3.7	5.9	2806	4.0	2.9	5.4	8221	4.4	3.7	5.3
CZP	95	2.1	0.2	17.3	161	2.5	0.0	131.8	256	2.3	0.8	6.6
GBP	192	2.0	0.1	32.2	18	ND	ND	ND	210	1.4	0.3	6.8
LAC	1	ND	ND	ND	0	ND	ND	ND	1	ND	ND	ND
LEV	1242	2.6	1.6	4.4	248	2.8	0.0	321.9	1490	2.8	1.8	4.3
LTG	4704	2.7	1.9	3.8	2502	3.5	2.5	4.9	7206	2.9	2.3	3.7
OXC	378	2.8	1.1	6.6	507	4.8	0.7	31.5	885	3.1	1.3	7.4
PB	840	6.3	4.8	8.3	34	8.8	0.0	9722.4	874	6.4	4.9	8.4
PHT	1327	5.4	3.6	8.1	103	6.8	0.1	701.2	1430	5.5	3.9	87.9
PRM	112	7.9	2.6	21.5	3	ND	ND	ND	115	7.6	2.5	21.0
TPM	510	3.9	2.3	6.5	49	4.1	0.0	27,060.0	559	3.9	2.4	6.3
VPA	3018	9.8	8.1	11.9	1482	9.7	7.1	13.4	4500	9.7	8.4	11.3
ZNS	130	2.7	0.1	47.3	0	ND	ND	ND	130	2.6	0.1	68.2
No med	1708	3.0	2.1	4.2	11,286	3.2	1.7	6.1	12,994	3.1	2.4	3.9
Gen POP	3537	2.1	1.5	3.0	373,028	3.3	1.5	7.1	376,565	2.5	1.8	3.3

Specific prevalences are weighted for cohort size. CBZ: Carbamazepine CI: Confidence Interval CZP: Clonazepam GBP: Gabapentin LAC: Lacosamide LEV: Levetiracetam  LTG: Lamotrigine ND: No Data OXC: Oxcarbazepine PB: Phenobarbital PHT: Phenytoin POP: Population  PRM: Primidone TPM: Topiramate VPA: Sodium Valproate ZNS: Zonisamide

**2:** Summary of findings table ‐ Carbamazepine

**Monotherapy treatment of epilepsy in pregnancy: congenital malformation outcomes in the child**					
Population: Pregnant women with epilepsy Intervention: ASM monotherapy Comparison: Carbamazepine in comparison to other ASMs Outcome: Major congenital malformation rate in exposed children					
**Comparison**		**Illustrative comparative risks across data types**		**Relative effect** **(95% CI)**	**N of participants (studies)**
		**Prevalence CBZ** **(95% CI)**	**Prevalence** **comparator** **(95% CI)**		
Carbamazepine vs no medication (women without epilepsy)	Cohort studies	CBZ 4.7% (3.7, 5.9)	2.1% (1.5, 3.0)	2.30 (1.47, 3.59)	5047 (13)
	Database studies	CBZ 4.0% (2.9, 5.4)	3.3% (1.5, 7.1)	1.14 (0.80, 1.64)	373,094 (2)
Carbamazepine vs no medication (women with epilepsy)	Cohort studies	CBZ 4.7% (3.7, 5.9)	3.0% (2.1, 4.2)	1.44 (1.05, 1.96)	5289 (20)
	Database studies	CBZ 4.0% (2.9, 5.4)	3.2% (1.7, 6.1)	1.42 (1.10, 1.83)^a^	14,334 (4)
Carbamazepine vs levetiracetam	Cohort studies	CBZ 4.7% (3.7, 5.9)	LEV 2.6% (1.6, 4.4)	1.51 (1.01, 2.26)	5056 (11)
	Database studies	CBZ 4.0% (2.9, 5.4)	LEV 2.8% (0.0, 321.9)	1.73 (0.78, 3.83)	1248 (2)
	EURAP	CBZ 5.5% (4.5, 6.6)	LEV 2.8% (1.7, 4.5)	N/A	2556
Carbamazepine vs lamotrigine	Cohort studies	CBZ 4.7% (3.7, 5.9)	LTG 2.7% (1.9, 3.8)	1.37 (1.06, 1.77)	8568 (13)
	Database studies	CBZ 4.0% (2.9, 5.4)	LTG 3.5% (2.5, 4.9)	1.21 (0.88, 1.67)	4503 (4)
	EURAP	CBZ 5.5% (4.5, 6.6)	LTG 2.9% (2.3, 3.7)	N/A	4471
Carbamazepine vs topiramate	Cohort studies	CBZ 4.7% (3.7, 5.9)	TPM 3.9% (2.3, 6.5)	0.83 (0.51, 1.33)	4156 (8)
	Database studies	CBZ 4.0% (2.9, 5.4)	TPM 4.1% (0.0, 27,060.0)	0.59 (0.17, 2.06)	1437 (2)
	EURAP	CBZ 5.5% (4.5, 6.6)	TPM 3.9% (1.5, 8.4)	N/A	2109
Carbamazepine vs valproate	Cohort studies	CBZ 4.7% (3.7, 5.9)	VPA 9.8% (8.1, 11.9)	0.44 (0.37, 0.53)	8090 (29)
	Database studies	CBZ 4.0% (2.9, 5.4)	VPA 9.7% (7.1, 13.4)	0.42 (0.33, 0.54)	4157 (5)
	EURAP	CBZ 5.5% (4.5, 6.6)	VPA 10.3% (8.8, 12.0)	N/A	3338
Carbamazepine vs oxcarbazepine	Cohort studies	CBZ 4.7% (3.7, 5.9)	OXC 2.8% (1.1, 6.6)	1.26 (0.74, 2.15)	2877 (11)
	Database studies	CBZ 4.0% (2.9, 5.4)	OXC 4.8% (0.7, 31.5)	0.64 (0.44, 0.91)^b^	3015 (4)
	EURAP	CBZ 5.5% (4.5, 6.6)	OXC 3.0% (1.4, 5.4)	N/A	2290
Carbamazepine vs zonisamide	Cohort studies	CBZ 4.7% (3.7, 5.9)	2.7% (0.1, 47.3)	0.86 (0.07, 10.35)^b^	2841 (4)
	Database studies	CBZ 4.0% (2.9, 5.4)	N/A	N/A	N/A
	EURAP	CBZ 5.5% (4.5, 6.6)	N/A	N/A	N/A

^a^ RD was non‐significant; ^b^ Random‐effects RR calculated due to heterogeneity ASM: Anti‐Seizure Medication  CBZ: Carbamazepine CI: Confidence Interval LEV: Levetiracetam  LTG: Lamotrigine N/A: Not Available OXC: Oxcarbazepine TPM: Topiramate VPA: Sodium Valproate

**3:** Summary of findings table ‐ Oxcarbazepine

**Monotherapy treatment of epilepsy in pregnancy: congenital malformation outcomes in the child**					
Population: Pregnant women with epilepsy Intervention: ASM monotherapy Comparison: Oxcarbazepine in comparison to other ASMs Outcome: Major congenital malformation rate in exposed children					
**Comparison**		**Illustrative comparative risks across data types**		**Relative effect (95% CI)**	**N of participants (studies)**
		**OXC Prevalence** **(95% CI)**	**Prevalence** **comparator** **(95% CI)**		
Oxcarbazepine vs no medication (women without epilepsy)	Cohort studies	OXC 2.8% (1.1, 6.6)	Gen Pop 2.1% (1.5, 3.0)	2.20 (0.67, 7.27)	951(2)
	Database studies	OXC 4.8% (0.7, 31.5)	Gen Pop 3.3 (1.5, 7.1)	0.70 (0.10, 4.86)	369,324 (1)
Oxcarbazepine vs no medication (women with epilepsy)	Cohort studies	OXC 2.8% (1.1, 6.6)	No Med 3.0 (2.1, 4.2)	1.40 (0.68, 2.91)	922 (6)
	Database studies	OXC 4.8% (0.7, 31.5)	No Med 3.2 (1.7, 6.1)	1.75 (1.22, 2.52)^a^	11,819 (3)
Levetiracetam vs oxcarbazepine	Cohort studies	OXC 2.8% (1.1, 6.6)	LEV 2.6% (1.6, 4.4)	1.04 (0.51, 2.09)	1166 (8)
	Database studies	OXC 4.8% (0.7, 31.5)	LEV 2.8% (0.0, 321.9)	1.17 (0.45, 3.06)	621 (2)
	EURAP	OXC 3.0% (1.4, 5.4)	LEV 2.8% (1.7, 4.5)	N/A	932
Lamotrigine Vs oxcarbazepine	Cohort studies	OXC 2.8% (1.1, 6.6)	LTG 2.7% (1.9, 3.8)	0.73 (0.33, 1.62)	2541 (8)
	Database studies	OXC 4.8% (0.7, 31.5)	LTG 3.5% (2.5, 4.9)	1.24 (0.67, 2.30)	2535 (3)
	EURAP	OXC 3.0% (1.4, 5.4)	LTG 2.9% (2.3, 3.7)	N/A	2847
Oxcarbazepine vs topiramate	Cohort studies	OXC 2.8% (1.1, 6.6)	TPM 3.9% (2.3, 6.5)	0.71 (0.28, 1.77)	706 (5)
	Database studies	OXC 4.8% (0.7, 31.5)	TPM 4.1% (0.0, 27060.0)	0.42 (0.04, 4.50)	110 (2)
	EURAP	OXC 3.0% (1.4, 5.4)	TPM 3.9% (1.5, 8.4)	N/A	485
Valproate vs oxcarbazepine	Cohort studies	OXC 2.8% (1.1, 6.6)	VPA 9.8% (8.1, 11.9)	2.48 (1.42, 4.31)	1561 (11)
	Database studies	OXC 4.8% (0.7, 31.5)	VPA 9.7% (7.1, 13.4)	1.60 (1.11, 2.29)^a^	1701 (4)
	EURAP	OXC 3.0% (1.4, 5.4)	VPA 10.3% (8.8, 12.0)	N/A	1714
Carbamazepine vs oxcarbazepine	Cohort studies	OXC 2.8% (1.1, 6.6)	CBZ 4.7% (3.7, 5.9)	1.26 (0.74, 2.15)	2887 (11)
	Database studies	OXC 4.8% (0.7, 31.5)	CBZ 4.0% (2.9, 5.4)	0.64 (0.44, 0.91)^a^	3015 (4)
	EURAP	OXC 3.0% (1.4, 5.4)	CBZ 5.5% (4.5, 6.6)	N/A	2290
Oxcarbazepine vs zonisamide	Cohort studies	OXC 2.8% (1.1, 6.6)	ZNS 2.7% (0.1, 47.3)	4.48 (0.24, 82.23)	277 (2)
	Database studies	OXC 4.8% (0.7, 31.5)	N/A	N/A	N/A
	EURAP	OXC 3.0% (1.4, 5.4)	N/A	N/A	N/A

^a^ Random‐effects RR calculated due to heterogeneity ASM: Anti‐Seizure Medication  CBZ: Carbamazepine CI: Confidence Interval LEV: Levetiracetam  LTG: Lamotrigine N/A: Not Available OXC: Oxcarbazepine TPM: Topiramate VPA: Sodium Valproate

**4:** Summary of findings table ‐ Topiramate

**Monotherapy treatment of epilepsy in pregnancy: congenital malformation outcomes in the child**					
Population: Pregnant women with epilepsy Intervention: ASM monotherapy Comparison: Topiramate in comparison to other ASMs Outcome: Major congenital malformation rate in exposed children					
**Comparison**		**Illustrative comparative risks across data types**		**Relative effect** **(95% CI)**	**N of participants (studies)**
		**TPM Prevalence** **(95% CI)**	**Prevalence** **comparator** **(95% CI)**		
Topiramate vs no medication (women without epilepsy)	Cohort studies	TPM 3.9% (2.3, 6.5)	Gen Pop 2.1% (1.5, 3.0)	4.07 (1.64, 10.14)	1192 (3)
	Database studies	TPM 4.1% (0.0, 27060.0)	Gen Pop 3.3 (1.5, 7.1)	1.65 (0.43, 6.42)	369,315 (1)
Topiramate vs no medication (women with epilepsy)	Cohort studies	TPM 3.9% (2.3, 6.5)	No Med 3.0 (2.1, 4.2)	1.37 (0.57, 3.27)	1219 (5)
	Database studies	TPM 4.1% (0.0, 27060.0)	No Med 3.2 (1.7, 6.1)	1.62 (0.40, 6.45)	1948 (1)
Levetiracetam vs topiramate	Cohort studies	TPM 3.9% (2.3, 6.5)	LEV 2.6% (1.6, 4.4)	0.57 (0.32, 1.04)	1629 (8)
	Database studies	TPM 4.1% (0.0, 27060.0)	LEV 2.8% (0.0, 321.9)	0.41 (0.06, 2.81)	166 (1)
	EURAP	TPM 3.9% (1.5, 8.4)	LEV 2.8% (1.7, 4.5)	N/A	751
Lamotrigine vs topiramate	Cohort studies	TPM 3.9% (2.3, 6.5)	LTG 2.7% (1.9, 3.8)	0.59 (0.36, 0.96)	4780 (8)
	Database studies	TPM 4.1% (0.0, 27060.0)	LTG 0.68% (0.20, 2.37)	0.68 (0.20, 2.37)	972 (2)
	EURAP	TPM 3.9% (1.5, 8.4)	LTG 2.9% (2.3, 3.7)	N/A	2666
Oxcarbazepine vs topiramate	Cohort studies	TPM 3.9% (2.3, 6.5)	OXC 2.8% (1.1, 6.6)	0.71 (0.28, 1.77)	706 (5)
	Database studies	TPM 4.1% (0.0, 27060.0)	OXC 4.8% (0.7, 31.5)	0.42 (0.04, 4.50)	110 (2)
	EURAP	TPM 3.9% (1.5, 8.4)	OXC 3.0% (1.4, 5.4)	N/A	485
Valproate vs topiramate	Cohort studies	TPM 3.9% (2.3, 6.5)	VPA 9.8% (8.1, 11.9)	2.47 (1.50, 4.08)	2723 (7)
	Database studies	TPM 4.1% (0.0, 27060.0)	VPA 9.7% (7.1, 13.4)	1.27 (0.36, 4.39)	650 (2)
	EURAP	TPM 3.9% (1.5, 8.4)	VPA 10.3% (8.8, 12.0)	N/A	1533
Carbamazepine vs topiramate	Cohort studies	TPM 3.9% (2.3, 6.5)	CBZ 4.7% (3.7, 5.9)	0.83 (0.51, 1.33)	4156 (8)
	Database studies	TPM 4.1% (0.0, 27060.0)	CBZ 4.0% (2.9, 5.4)	0.59 (0.17, 2.06)	1437 (2)
	EURAP	TPM 3.9% (1.5, 8.4)	CBZ 5.5% (4.5, 6.6)	N/A	2109
Topiramate vs zonisamide	Cohort studies	TPM 3.9% (2.3, 6.5)	ZNS 2.7% (0.1, 47.3)	1.59 (0.54, 4.66)^a^	570 (4)
	Database studies	TPM 4.1% (0.0, 27060.0)	N/A	N/A	N/A
	EURAP	TPM 3.9% (1.5, 8.4)	N/A	N/A	N/A

^a^ Random‐effects RR calculated due to heterogeneity ASM: Anti‐Seizure Medication  CBZ: Carbamazepine CI: Confidence Interval Gen pop: General population LEV: Levetiracetam  LTG: Lamotrigine N/A: Not Available OXC: Oxcarbazepine TPM: Topiramate VPA: Sodium Valproate

**5:** Summary of findings table ‐ Valproate

**Monotherapy treatment of epilepsy in pregnancy: congenital malformation outcomes in the child**					
Population: Pregnant women with epilepsy Intervention: ASM monotherapy Comparison: Valproate in comparison to other ASMs Outcome: Major congenital malformation rate in exposed children					
**Comparison**		**Illustrative comparative risks across data types**		**Relative effect** **(95% CI)**	**N of participants (studies)**
		**VPA Prevalence** **(95% CI)**	**Prevalence** **comparator** **(95% CI)**		
Valproate vs no medication (women without epilepsy)	Cohort studies	VPA 9.8% (8.1, 11.9)	Gen Pop 2.1% (1.5, 3.0)	5.53 (3.29, 9.29)	3135 (10)
	Database studies	VPA 9.7% (7.1, 13.4)	Gen Pop 3.3 (1.5, 7.1)	2.29 (1.71, 3.08)	373,649 (3)
Valproate vs no medication (women with epilepsy)	Cohort studies	VPA 9.8% (8.1, 11.9)	No Med 3.0 (2.1, 4.2)	2.77 (2.03, 3.79)	3998 (17)
	Database studies	VPA 9.7% (7.1, 13.4)	No Med 3.2 (1.7, 6.1)	3.01 (2.42, 3.75)^a^	13,369 (4)
Valproate vs levetiracetam	Cohort studies	VPA 9.8% (8.1, 11.9)	LEV 2.6% (1.6, 4.4)	3.77 (2.48, 5.74)	3485(10)
	Database studies	VPA 9.7% (7.1, 13.4)	LEV 2.8% (0.0, 321.9)	3.26 (1.51, 7.03)	911 (2)
	EURAP	VPA 10.3% (8.8, 12.0)	LEV 2.8% (1.7, 4.5)	N/A	1980
Valproate vs lamotrigine	Cohort studies	VPA 9.8% (8.1, 11.9)	LTG 2.7% (1.9, 3.8)	3.50 (2.76, 4.46)	6896 (12)
	Database studies	VPA 9.7% (7.1, 13.4)	LTG 0.68% (0.20, 2.37)	2.49 (1.86, 3.35)	3590 (4)
	EURAP	VPA 10.3% (8.8, 12.0)	LTG 2.9% (2.3, 3.7)	N/A	3894
Valproate vs oxcarbazepine	Cohort studies	VPA 9.8% (8.1, 11.9)	OXC 2.8% (1.1, 6.6)	2.48 (1.42, 4.31)	1561 (11)
	Database studies	VPA 9.7% (7.1, 13.4)	OXC 4.8% (0.7, 31.5)	1.60 (1.11, 2.29)^a^	1701 (4)
	EURAP	VPA 10.3% (8.8, 12.0)	OXC 3.0% (1.4, 5.4)	N/A	1714
Valproate vs topiramate	Cohort studies	VPA 9.8% (8.1, 11.9)	TPM 3.9% (2.3, 6.5)	2.47 (1.50, 4.08)	2723 (7)
	Database studies	VPA 9.7% (7.1, 13.4)	TPM 4.1% (0.0, 27060.0)	1.27 (0.36, 4.39)	650 (2)
	EURAP	VPA 10.3% (8.8, 12.0)	TPM 3.9% (1.5, 8.4)	N/A	152
Carbamazepine vs valproate	Cohort studies	VPA 9.8% (8.1, 11.9)	CBZ 4.7% (3.7, 5.9)	0.44 (0.37, 0.53)	8090 (29)
	Database studies	VPA 9.7% (7.1, 13.4)	CBZ 4.0% (2.9, 5.4)	0.42 (0.33, 0.54)	4157 (5)
	EURAP	VPA 10.3% (8.8, 12.0)	CBZ 5.5% (4.5, 6.6)	N/A	3338
Valproate vs zonisamide	Cohort studies	VPA 9.8% (8.1, 11.9)	ZNS 2.7% (0.1, 47.3)	2.34 (0.95, 5.80)^a^	1677 (3)
	Database studies	VPA 9.7% (7.1, 13.4)	N/A	N/A	N/A
	EURAP	VPA 10.3% (8.8, 12.0)	N/A	N/A	N/A

^a^ Random‐effects RR calculated due to heterogeneity ASM: Anti‐Seizure Medication  CBZ: Carbamazepine CI: Confidence Interval Gen pop: General population LEV: Levetiracetam  LTG: Lamotrigine N/A: Not Available OXC: Oxcarbazepine TPM: Topiramate VPA: Sodium Valproate

**6:** Relative risks (RRs) for specific ASM comparisons

	**Gen Pop**	**No Med**	**CBZ**	**CZP**	**GBP**	**LEV**	**LTG**	**OXC**	**PB**	**PHT**	**PRM**	**TPM**	**VPA**	**ZNS**
**CBZ**	**2.30 (1.47 to 3.59)**	**1.44 (1.05 to 1.96)**		1.82 (0.63 to 5.26)	1.55 (0.57 to 4.26)	**1.51 (1.01 to 2.26)**	**1.37 (1.06 to 1.77)**	1.26 (0.74 to 2.15)	0.83 (0.61 to 1.13)	0.83 (0.62 to 1.11)	0.59 (0.23 to 1.56)	0.83 (0.51 to 1.33)	**0.44 (0.37 to 0.53)**	0.94, (0.36 to 2.44)
**CZP**	2.76 (0.55 to 13.94)	1.08 (0.21 to 5.42)	1.82 (0.63 to 5.26)		ND	1.06 (0.32 to 3.44)	0.92 (0.29 to 2.91)	0.25 (0.01 to 5.75)	0.83 (0.05 to 13.02)	0.71 (0.10 to 5.11)	NE	0.67 (0.03 to 15.83)	**0.29 (0.09 to 0.90)**	ND
**GBP**	1.78 (0.50 to 6.29)	1.77 (0.46 to 6.90)	1.55 (0.57 to 4.26)	ND		1.61 (0.46 to 5.63)	0.92 (0.34 to 2.47)	0.53 (0.13 to 2.17)	0.30 (0.08 to 1.14)	2.15 (0.69 to 6.73)	ND	0.32 (0.09 to 1.19)	**4.27 (1.60 to 11.35)**	0.53 (0.10 to 2.76)
**LEV**	2.20 (0.98 to 4.93)	0.71 (0.39 to 1.28)	**1.51 (1.01 to 2.26)**	1.06 (0.32 to 3.44)	1.61 (0.46 to 5.63)		0.90 (0.58 to 1.39)	1.04 (0.51 to 2.09)	0.54 (0.29 to 1.02)	**0.58 (0.34 to 0.97)**	0.24 (0.02 to 3.37)	0.57 (0.32 to 1.04)	**3.77 (2.48 to 5.74)**	0.66 (0.25 to 1.71)
**LTG**	**1.99 (1.16 to 3.39)**	1.04 (0.66 to 1.63)	**1.37 (1.06 to 1.77)**	0.92 (0.29 to 2.91)	0.92 (0.34 to 2.47)	0.90 (0.58 to 1.39)		0.73 (0.33 to 1.62)	**0.32 (0.17 to 0.59)**	**0.55 (0.35 to 0.87)**	0.30 (0.02 to 3.93)	**0.59 (0.36 to 0.96)**	**3.50 (2.76 to 4.46)**	0.66 (0.26 to 1.65)
**OXC**	2.20 (0.67 to 7.27)	1.40 (0.68 to 2.91)	1.26 (0.74 to 2.15)	0.25 (0.01 to 5.75)	0.53 (0.13 to 2.17)	1.04 (0.51 to 2.09)	0.73 (0.33 to 1.62)		1.61 (0.83 to 3.14)	0.94 (0.48 to 1.85)	0.58 (0.08 to 4.03)	0.71 (0.28 to 1.77)	**2.48 (1.42 to 4.31)**	4.48 (0.24 to 82.23)
**PB**	**3.22 (1.84 to 5.65)**	1.64 (0.94 to 2.83)	0.83 (0.61 to 1.13)	0.83 (0.05 to 13.02)	0.30 (0.08 to 1.14)	0.54 (0.29 to 1.02)	**0.32 (0.17 to 0.59)**	1.61 (0.83 to 3.14)		0.84 (0.57 to 1.23)	0.50 (0.21 to 1.16)	1.38 (0.68 to 2.81)	**1.49 (1.08 to 2.07)**	10.46 (0.62 to 175.67)
**PHT**	**3.81 (1.91 to 7.57)**	**2.01 (1.29 to 3.12)**	0.83 (0.62 to 1.11)	0.71 (0.10 to 5.11)	2.15 (0.69 to 6.73)	**0.58 (0.34 to 0.97)**	**0.55 (0.35 to 0.87)**	0.94 (0.48 to 1.85)	0.84 (0.57 to 1.23)		0.78 (0.39 to 1.56)	0.88 (0.48 to 1.61)	**1.92 (1.44 to 2.56)**	1.28 (0.42 to 3.93)
**PRM**	NE	**3.61 (1.41 to 9.23)**	0.59 (0.23 to 1.56)	NE	ND	0.24 (0.02 to 3.37)	0.30 (0.02 to 3.93)	0.58 (0.08 to 4.03)	0.50 (0.21 to 1.16)	0.78 (0.39 to 1.56)		6.00 (0.30 to 118.36)	0.74 (0.39 to 1.40)	ND
**TPM**	**4.07 (1.64 to 10.14)**	1.37 (0.57 to 3.27)	0.83 (0.51 to 1.33)	0.67 (0.03 to 15.83)	0.32 (0.09 to 1.19)	0.57 (0.32 to 1.04)	**0.59 (0.36 to 0.96)**	0.71 (0.28 to 1.77)	1.38 (0.680 to 2.81)	0.88 (0.48 to 1.61)	6.00 (0.30 to 118.36)		**2.47 (1.50 to 4.08)**	1.59 (0.54 to 4.66)
**VPA**	**5.53 (3.29 to 9.29)**	**2.77 (2.03 to 3.79)**	**0.44 (0.37 to 0.53)**	**0.29 (0.09 to 0.90)**	**4.27 (1.60 to 11.35)**	**3.77 (2.48 to 5.74)**	**3.50 (2.76 to 4.46)**	**2.48 (1.42 to 4.31)**	**1.49 (1.08 to 2.07)**	**1.92 (1.44 to 2.56)**	0.74 (0.39 to 1.40)	**2.47 (1.50 to 4.08)**		2.34 (0.95 to 5.80)
**ZNS**	1.13 (0.21 to 6.11)	**3.20 (1.09 to 9.43)**	0.94, (0.36 to 2.44)	ND	0.53 (0.10 to 2.76)	0.66 (0.25 to 1.71)	0.66 (0.26 to 1.65)	4.48 (0.24 to 82.23)	10.46 (0.62 to 175.67)	1.28 (0.42to 3.93)	ND	1.59 (0.54 to 4.66)	2.34 (0.95 to 5.80)	

Bold indicates statistically significant ASM: Anti‐Seizure Medication CBZ: Carbamazepine CZP: Clonazepam GBP: Gabapentin LEV: Levetiracetam  LTG: Lamotrigine ND: No Data NE: Not Estimable OXC: Oxcarbazepine PB: Phenobarbital PHT: Phenytoin POP: Population  PRM: Primidone RR: Relative Risk  TPM: Topiramate VPA: Sodium Valproate ZNS: Zonisamide

**7:** Risk differences (RDs) for specific ASM comparisons

	**Gen Pop**	**No Med**	**CBZ**	**CZP**	**GBP**	**LEV**	**LTG**	**OXC**	**PB**	**PHT**	**PRM**	**TPM**	**VPA**	**ZNS**
**CBZ**	**0.02 (0.01 to 0.03)**	**0.01 (0.00 to 0.02)**		**0.04,** **(‐0.00 to 0.08)**	0.02 (‐0.00 to 0.04)	**0.01 (0.00 to 0.02)**	**0.01 (0.00 to 0.02)**	0.01 ( −0.01 to 0.03)	−0.01 (−0.03 to 0.01)	−0.01 (−0.02 to 0.01)	−0.02 (−0.09 to 0.05)	−0.01 (−0.02 to 0.01)	**−0.05 (−0.06 to −0.04)**	0.00 (‐0.03 to 0.03)
**CZP**	0.02 (‐0.03 to 0.07)	‐0.03 (‐0.11 to 0.04)	**0.04,** **(‐0.00 to 0.08)**		−0.04 (−0.14 to 0.05)	−0.01 (−0.05 to 0.03)	0.01 (‐0.03 to 0.04)	−0.05 (−0.18 to 0.07)	−0.08 (−0.66 to 0.51)	−0.04 (−0.13 to 0.06)	NE	−0.02 (−0.09 to 0.05)	**−0.09 (−0.13 to 0.04)**	ND
**GBP**	0.19 (‐0.37 to 0.74)	0.01 (−0.05 to 0.07)	0.02 (‐0.00 to 0.04)	−0.04 (−0.14 to 0.05)		0.01 (−0.01 to 0.03)	−0.01 (−0.03 to 0.01)	−0.01 (−0.04 to 0.01)	**−0.04 (−0.08 to 0.00)**	0.02 (‐0.00 to 0.04)	ND	**−0.03 (−0.05 to −0.01)**	**0.08 (0.01 to 0.14)**	‐0.03 (‐0.15 to 0.10)
**LEV**	0.01 (−0.00 to 0.03)	−0.01 (−0.03 to 0.00)	**0.01 (0.00 to 0.02)**	−0.01 (−0.05 to 0.03)	0.01 (−0.01 to 0.03)		−0.00 (−0.01 to 0.01)	0.00 (−0.02 to 0.03)	−0.02 (−0.05 to 0.01)	−0.02 (−0.04 to −0.00)	0.04 (‐0.39 to 0.46)	−0.02 (−0.04 to 0.00)	**0.07 (0.05 to 0.08)**	0.01 (‐0.04 to 0.03)
**LTG**	**0.01 (0.00 to 0.03)**	0.00 (−0.01 to 0.01)	**0.01 (0.00 to 0.02)**	0.01 (‐0.03 to 0.04)	−0.01 (−0.03 to 0.01)	−0.00 (−0.01 to 0.01)		‐0.01 (−0.03 to 0.02)	**−0.04 (−0.07 to −0.01)**	**−0.02 (−0.03 to −0.00)**	0.05 (−0.37 to 0.47)	−0.02 (−0.03 to 0.00)	**0.06 (0.05 to 0.08)**	‐0.03 (‐0.16 to 0.11)
**OXC**	0.01 (−0.02 to 0.04)	0.02 (−0.03 to 0.07)	0.01 ( −0.01 to 0.03)	−0.05 (−0.18 to 0.07)	−0.01 (−0.04 to 0.01)	0.00 (−0.02 to 0.03)	‐0.01 (−0.03 to 0.02)		0.02 (−0.02 to 0.06)	0.00 (−0.03 to 0.03)	−0.02 (−0.34 to 0.30)	−0.01 (−0.04 to 0.02)	**0.06 (0.03 to 0.09)**	0.02 (‐0.01 to 0.05)
**PB**	**0.04 (0.01 to 0.07)**	0.02 (−0.01 to 0.06)	−0.01 (−0.03 to 0.01)	−0.08 (−0.66 to 0.51)	**−0.04 (−0.08 to 0.00)**	−0.02 (−0.05 to 0.01)	**−0.04 (−0.07 to −0.01)**	0.02 (−0.02 to 0.06)		−0.01 (−0.03 to 0.02)	−0.05 (−0.12 to 0.02)	0.02 (−0.02 to 0.05)	**0.04 (0.01 to 0.06)**	**0.05 (0.02 to 0.09)**
**PHT**	**0.03 (0.01 to 0.06)**	**0.03 (0.01 to 0.05)**	−0.01 (−0.02 to 0.01)	−0.04 (−0.13 to 0.06)	0.02 (‐0.00 to 0.04)	−0.02 (−0.04 to −0.00)	0.02 (‐0.00 to 0.04)	0.00 (−0.03 to 0.03)	−0.01 (−0.03 to 0.02)		−0.02 (−0.09 to 0.06)	−0.00 (−0.03 to 0.02)	**0.05 (0.03 to 0.07)**	0.00 (‐0.11 to 0.11)
**PRM**	NE	**0.07 (0.00 to 0.14)**	−0.02 (−0.09 to 0.05)	NE	ND	0.04 (‐0.39 to 0.46)	0.05 (−0.37 to 0.47)	−0.02 (−0.34 to 0.30)	−0.05 (−0.12 to 0.02)	−0.02 (−0.09 to 0.06)		−0.02 (−0.44 to 0.41)	0.04 (‐0.13 to 0.04)	ND
**TPM**	**0.03 (0.01 to 0.06)**	0.01 (−0.03 to 0.04)	−0.01 (−0.02 to 0.01)	−0.02 (−0.09 to 0.05)	**−0.03 (−0.05 to −0.01)**	−0.02 (−0.04 to 0.00)	−0.02 (−0.03 to 0.00)	−0.01 (−0.04 to 0.02)	0.02 (−0.02 to 0.05)	−0.00 (−0.03 to 0.02)	−0.02 (−0.44 to 0.41)		**0.07 (0.02 to 0.11)**	0.02 (0.02 to 0.06)
**VPA**	**0.07 (0.04 to 0.10)**	**0.06 (0.04 to 0.07)**	**−0.05 (−0.06 to −0.04)**	**−0.09 (−0.13 to 0.04)**	**0.08 (0.01 to 0.14)**	**0.07 (0.05 to 0.08)**	**0.06 (0.05 to 0.08)**	**0.06 (0.03 to 0.09)**	**0.04 (0.01 to 0.06)**	**0.05 (0.03 to 0.07)**	0.04 (‐0.13 to 0.04)	**0.07 (0.02 to 0.11)**		0.04 (0.11 to 0.19)
**ZNS**	−0.00 (−0.03 to 0.02)	0.07 (−0.03 to 0.18)	0.00 (‐0.03 to 0.03)	ND	‐0.03 (‐0.15 to 0.10)	0.01 (‐0.04 to 0.03)	‐0.03 (‐0.16 to 0.11)	0.02 (‐0.01 to 0.05)	**0.05 (0.02 to 0.09)**	0.00 (‐0.11 to 0.11)	ND	0.02 (0.02 to 0.06)	0.04 (0.11 to 0.19)	

Bold indicates statistical significance ASM: Anti‐Seizure Medication CBZ: Carbamazepine CZP: Clonazepam GBP: Gabapentin LEV: Levetiracetam  LTG: Lamotrigine ND: No Data NE: Not Estimable OXC: Oxcarbazepine PB: Phenobarbital PHT: Phenytoin POP: Population  PRM: Primidone TPM: Topiramate VPA: Sodium Valproate ZNS: Zonisamide

The Robins‐I was adapted for use here to understand the risk of biases but is not yet optimised for pregnancy pharmacovigilance work and, therefore, caution is required in the interpretation of its ratings. It did, however, show that different methodological approaches have different patterns of biases and are therefore in part complimentary (Figure 2). Cohort studies with primary data collection, for example, tend to have lower risks of misclassification of treatment and standardised review of the congenital malformation outcome in the children (leading to low risk of bias ratings), yet they are at higher risk of bias for cohort selection. The use of routine health record data at a national population level does not have these selection risks, however. Stratification of the results by study type provides an internal validation for the results (Figure 3) and the evidence presented in this review should be considered more certain when the results of different comparisons are consistent across study types.

**2:**
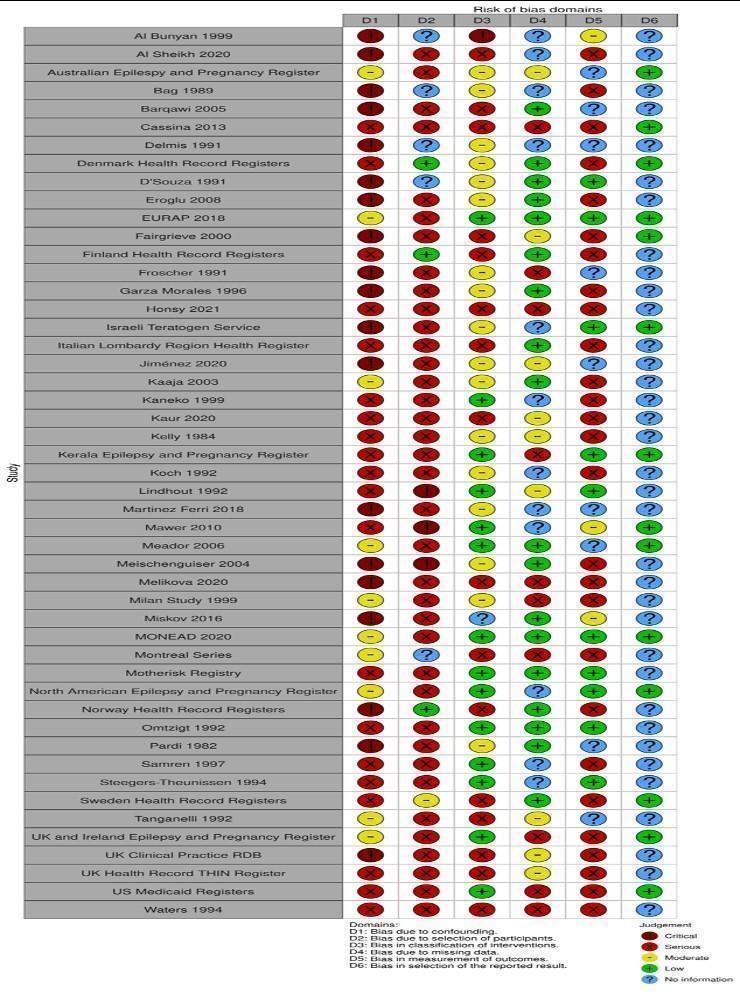
Risk of bias for included studies by individual domain

**3:**
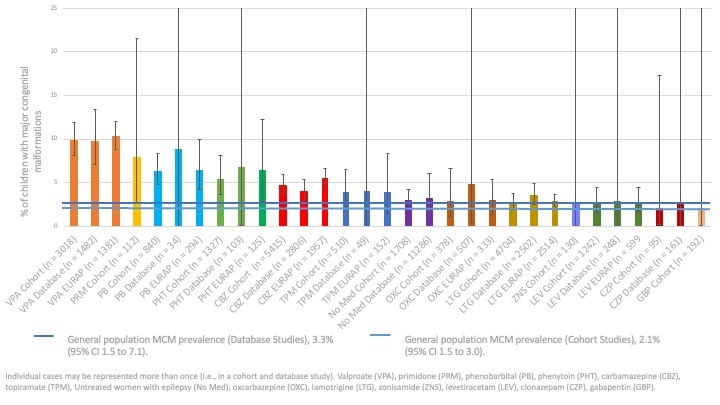
Prevalence and 95% CI of major congenital malformations for each anti‐seizure medication by data source

Malformations are rare outcomes and therefore larger groups are needed to reliably detect a higher risk of malformation in one group over another. Therefore, the certainty of the evidence is greater for medications such as VPA, carbamazepine (CBZ) and lamotrigine (LTG) where the numbers of children are higher within and across the comparisons. The available data were more moderate for levetiracetam (LEV), phenytoin (PHT) and phenobarbital (PB) in certain comparisons. Care should be taken in the interpretation of comparisons where there were fewer than 1000 pregnancies.

## Results

### Description of studies

#### Results of the search

In this updated review, electronic searches identified 1067 additional publications; this was in addition to the 11,695 records previously detected in searches for an earlier version of this review (Weston 2016). We found two additional records through handsearching. Following the removal of duplicates, 12,296 abstracts were screened for inclusion in the review across the original and this update. We excluded 12,013 abstracts due to irrelevance, leaving 283 full texts (156 new for this update) to be assessed for eligibility. As the inclusion criteria had been extended to include studies using routine health records in this update, we re‐evaluated search results from the last version for such studies and identified eight additional studies (14 papers). In total, we excluded 155 full‐text papers where they did not meet the inclusion criteria. See Characteristics of excluded studies and Figure 1 for the study flow diagram. We ultimately included 49 studies (128 publications) in this review. Of these, 113 records and 45 studies contributed data to the meta‐analyses, two studies had certain data included in the meta‐analysis whilst other data were narratively reviewed.

#### Included studies

A total of 128 included full‐text publications reported on the 49 independent studies included in this review, of which all but one were non‐randomised studies. The high number of publications per study were from longitudinal research initiatives such as epilepsy and pregnancy registers which update their results periodically. These full texts were related to an included study, as they presented information on the same cohort of children but either at a different time point or on a related, but not included, outcome (i.e. obstetric or neurodevelopmental outcome). Reported outcomes for each ASM were taken from the most relevant publication within a series; therefore, malformation information for specific ASMs may come from different publications within a series.

#### Excluded studies

We excluded 42 studies (55 papers) from the review (Excluded studies). Several of these papers were not written in the English language and, therefore, were sent for translation and data extraction in order to determine the study design and methodology used. The most frequent reasons for exclusion, however, were absence of reported ASM monotherapy‐specific malformation outcomes, retrospective study design, and case‐control study design. Studies were also excluded where the maternal indication was not epilepsy in 70% or more of participants, or if a subgroup analysis was not provided for women with epilepsy indication. These decisions were made to limit the likely heterogeneity regarding doses of ASMs used across indications, as dose is a significant driver of higher malformation risk (Brent 2004).

### Risk of bias in included studies

Robins‐I ratings are displayed in Figure 2.

#### Bias in confounding

For bias in confounding, no studies were rated as low as no studies were comparable to a randomised controlled trial. Ten studies were rated as moderate (Australian Epilepsy and Pregnancy Register; EURAP 2018; Kaaja 2003; Meador 2006; Milan Study 1999; MONEAD 2020; Montreal Series; North American Epilepsy and Pregnancy Register; Tanganelli 1992; UK and Ireland Epilepsy and Pregnancy Register) which is the highest rating for non‐randomised studies in this domain. Twenty studies were rated as serious due to a lack of control for key confounders (Cassina 2013; Denmark Health Record Registers; Finland Health Record Registers; Hosny 2021; Italian Lombardy Region Health Register; Kaneko 1999; Kaur 2020; Kelly 1984; Kerala Epilepsy and Pregnancy Registry; Koch 1992; Lindhout 1992; Mawer 2010; Motherisk Registry; Omtzigt 1992; Samren 1997; Steegers‐Theunissen 1994; Sweden Health Record Registers; UK Health Record THIN Register; US Medicaid Registers; Waters 1994), and nine studies were rated as critical (Al Bunyan 1999; AlSheikh 2020; Bag 1989; Barqawi 2005; Delmiš 1991, D'Souza 1991; Eroglu 2008; Fairgrieve 2000; Fröscher 1991; Garza‐Morales 1996; Israeli Teratogen Service; Jimenez 2020; Martinez Ferri 2018; Meischenguiser 2004; Melikova 2020; Miskov 2016; Norwegian Health Record Registers; Pardi 1982; UK Clinical Research Practice Database).

#### Bias in selection

For bias in selection, three studies were rated as low (Denmark Health Record Registers, Finland Health Record Registers, Norwegian Health Record Registers) as they represented national datasets and one study was rated as moderate (Sweden Health Record Registers). All cohort or pregnancy register studies were at risk of selection biases and therefore 37 studies were rated as serious (AlSheikh 2020; Australian Epilepsy and Pregnancy Register; Barqawi 2005; Cassina 2013; Eroglu 2008; EURAP 2018; Fairgrieve 2000; Fröscher 1991; Garza‐Morales 1996; Hosny 2021; Israeli Teratogen Service; Italian Lombardy Region Health Register; Jimenez 2020; Kaaja 2003; Kaneko 1999; Kaur 2020; Kelly 1984; Kerala Epilepsy and Pregnancy Registry; Koch 1992; Martinez Ferri 2018; Mawer 2010; Meador 2006; Melikova 2020; Milan Study 1999; Miskov 2016; MONEAD 2020; Motherisk Registry; North American Epilepsy and Pregnancy Register; Omtzigt 1992; Samren 1997; Steegers‐Theunissen 1994; Tanganelli 1992; UK and Ireland Epilepsy and Pregnancy Register; UK Clinical Research Practice Database; UK Health Record THIN Register; US Medicaid Registers; Waters 1994) and three studies were rated as critical due to the risk of selection biases (Fröscher 1991; Mawer 2010; Meischenguiser 2004). There was not sufficient information to rate five studies (Al Bunyan 1999; Bag 1989; D'Souza 1991; Delmiš 1991; Montreal Series).

#### Bias in classification

For bias in classification, 14 studies were rated as low (EURAP 2018; Kaneko 1999; Kerala Epilepsy and Pregnancy Registry; Lindhout 1992; Mawer 2010; Meador 2006; MONEAD 2020; Motherisk Registry; North American Epilepsy and Pregnancy Register; Omtzigt 1992; Samren 1997; Steegers‐Theunissen 1994; UK and Ireland Epilepsy and Pregnancy Register; US Medicaid Registers), 17 studies were rated as moderate (Australian Epilepsy and Pregnancy Register; Bag 1989; D'Souza 1991; Delmiš 1991; Denmark Health Record Registers; Eroglu 2008; Fröscher 1991; Garza‐Morales 1996; Israeli Teratogen Service; Jimenez 2020; Kaaja 2003; Kelly 1984; Koch 1992; Martinez Ferri 2018; Meischenguiser 2004; Milan Study 1999; Pardi 1982), 16 studies were rated as serious (AlSheikh 2020; Barqawi 2005; Cassina 2013; Fairgrieve 2000; Finland Health Record Registers; Hosny 2021; Italian Lombardy Region Health Register; Kaur 2020; Melikova 2020; Montreal Series; Norwegian Health Record Registers; Sweden Health Record Registers; Tanganelli 1992; UK Health Record THIN Register;UK Clinical Research Practice Database; Waters 1994), one study was rated as critical (Al Bunyan 1999) and the other had limited information (Miskov 2016).

#### Bias in missing data

For bias in missing data, 17 studies were rated as low (Barqawi 2005; D'Souza 1991; Denmark Health Record Registers; Eroglu 2008; EURAP 2018; Finland Health Record Registers; Garza‐Morales 1996; Italian Lombardy Region Health Register; Kaaja 2003; Meador 2006; Meischenguiser 2004; MONEAD 2020; Motherisk Registry; Norwegian Health Record Registers; Omtzigt 1992; Pardi 1982; Sweden Health Record Registers), nine studies were rated as moderate (Australian Epilepsy and Pregnancy Register; Fairgrieve 2000; Jimenez 2020; Kaaja 2003; Kaur 2020; Kelly 1984; Lindhout 1992; Tanganelli 1992; UK Health Record THIN Register; UK Clinical Research Practice Database), ten studies were rated as serious (Cassina 2013; Fröscher 1991; Hosny 2021; Kerala Epilepsy and Pregnancy Registry; Melikova 2020; Milan Study 1999; Montreal Series; UK and Ireland Epilepsy and Pregnancy Register; US Medicaid Registers; Waters 1994), and no studies were rated as critical. There was not sufficient information to rate levels of missing data in 13 studies, however (Al Bunyan 1999, AlSheikh 2020; Bag 1989; Delmiš 1991; Israeli Teratogen Service; Kaneko 1999; Koch 1992; Martinez Ferri 2018; Mawer 2010; Miskov 2016; North American Epilepsy and Pregnancy Register; Samren 1997; Steegers‐Theunissen 1994).

#### Bias in measurement

For bias in measurement, 11 studies were rated as low (D'Souza 1991; EURAP 2018; Fröscher 1991; Israeli Teratogen Service; Kerala Epilepsy and Pregnancy Registry; Lindhout 1992; MONEAD 2020; Motherisk Registry; North American Epilepsy and Pregnancy Register; Omtzigt 1992; Steegers‐Theunissen 1994) due to undertaking standardised reviews of the outcomes blinded to ASM exposure history. Two studies were rated as moderate (Mawer 2010; Miskov 2016) and 27 studies were rated as serious (AlSheikh 2020; Bag 1989; Cassina 2013; Denmark Health Record Registers; Eroglu 2008; Fairgrieve 2000; Finland Health Record Registers; Garza‐Morales 1996; Hosny 2021; Italian Lombardy Region Health Register; Kaaja 2003; Kaneko 1999; Kaur 2020; Kelly 1984; Koch 1992; Meischenguiser 2004; Melikova 2020; Milan Study 1999; Montreal Series; Norwegian Health Record Registers; Samren 1997; Sweden Health Record Registers; UK and Ireland Epilepsy and Pregnancy Register; UK Health Record THIN Register; US Medicaid Registers; UK Health Record THIN Register; Waters 1994) due to their use of routine clinical data which did not have standardised assessment and were not blinded to ASM exposure history. No studies were rated as critical, but there was insufficient information to rate the likelihood of measurement biases in nine studies (Australian Epilepsy and Pregnancy Register; Barqawi 2005; Delmiš 1991; Fröscher 1991Jimenez 2020; Martinez Ferri 2018; Meador 2006; Pardi 1982; Tanganelli 1992).

#### Bias in reporting

This domain was difficult to assess as, for most of the studies, no protocol was available (particularly for older studies) or contact with the authors could not be established (Al Bunyan 1999; AlSheikh 2020; Bag 1989; Barqawi 2005; D'Souza 1991; Delmiš 1991; Eroglu 2008; Finland Health Record Registers; Fröscher 1991; Garza‐Morales 1996; Hosny 2021; Italian Lombardy Region Health Register; Jimenez 2020; Kaaja 2003; Kaneko 1999; Kaur 2020; Kelly 1984; Koch 1992; Lindhout 1992; Martinez Ferri 2018; Meischenguiser 2004; Melikova 2020; Milan Study 1999; Miskov 2016; Montreal Series; Motherisk Registry; Norwegian Health Record Registers; Omtzigt 1992; Pardi 1982; Samren 1997; Steegers‐Theunissen 1994; Tanganelli 1992; UK Clinical Research Practice Database; UK Health Record THIN Register; UK Clinical Research Practice Database). Fourteen studies were rated as having low risk for reporting bias, where the protocol could be reviewed in relation to the outcomes and comparisons investigated (Australian Epilepsy and Pregnancy Register; Cassina 2013; Denmark Health Record Registers; EURAP 2018; Fairgrieve 2000; Israeli Teratogen Service; Kerala Epilepsy and Pregnancy Registry; Mawer 2010; Meador 2006; MONEAD 2020; North American Epilepsy and Pregnancy Register; Sweden Health Record Registers; UK and Ireland Epilepsy and Pregnancy Register; US Medicaid Registers).

### Effects of interventions

See: Table 1; Table 2

Each included comparison is reviewed below with both the meta‐analysis results being reported alongside any studies which required narrative review only. In comparisons where there were less than 50 children in both groups, the meta‐analysis is not reported, but the data is summarised narratively. Summary tables displaying the pooled prevalences, RR and RDs for each comparison are available in Table 3 along with Table 1 for lamotrigine; Table 2 for levetiracetam, Table 4 for carbamazepine, Table 5 for oxcarbazepine, Table 6 for topiramate, and Table 7 for valproate. A complete summary of all included ASM pooled prevalences, RR and RDs can be found in Table 3, Table 8, and Table 9, respectively with a visual presentation of the major malformation rates displayed in Figure 3.

#### Women without epilepsy

The prevalence of major malformations (any type) in the cohort studies for children of women without epilepsy (N = 3537), based on data from 12 studies, was 2.1% (95% CI 1.5 to 3.0). The prevalence of major malformations in routine health record studies for children of women without epilepsy (N = 373,028), based on data from three studies, was 3.3% (95% CI 1.5 to 7.1).

#### Women with epilepsy (no medication)

The prevalence of major malformations (any type) in the cohort studies for children of women with epilepsy (no medication) (N = 1708), based on data from 21 studies, was 3.0% (95% CI 2.1 to 4.2). The prevalence of major malformations in routine health record studies for children of women with epilepsy (no medication) (N = 11,286), based on data from three studies, was 3.2% (95% CI 1.7 to 6.1).

#### Carbamazepine

The prevalence of major malformations (any type) in the cohort studies for children exposed to carbamazepine (CBZ) (N = 5415), based on data from 37 studies, was 4.7% (95% CI 3.7 to 5.9). The prevalence of major malformations in routine health record studies for children exposed to CBZ (N = 2806), based on data from five studies, was 4.0% (95% CI 2.9 to 5.4).

##### 1 CBZ versus controls

###### 1.1 All major malformations

####### 1.1.1 CBZ versus no medication (in women without epilepsy): cohort studies

Pooled results from 13 cohort studies suggested an increased risk with CBZ (RR 2.30, 95% CI 1.47 to 3.59; I^2^ = 0%), with children exposed to CBZ (N = 1448) experiencing more major malformations than control children (N = 3599) (Analysis 1.1). The RD also suggested a higher absolute risk (RD 0.02, 95% CI 0.01 to 0.03; I^2^ = 0%) (Analysis 1.1).

The multicentre study, Samren 1997, reported 22 (8%) cases of major malformations from 280 infants exposed to CBZ. However, the numbers from centres with a control group were smaller, with four cases of malformation out of just 14 exposed infants. This suggested an increased risk relative to the control children born to women without epilepsy (RR 4.9, 95% CI 1.3 to 18.0).

####### 1.1.2 CBZ versus no medication (in women with epilepsy): cohort studies

Pooled findings from 20 cohort studies suggested an increased risk with CBZ (RR 1.44, 95% CI 1.05 to 1.96; I^2^ = 0%), with children exposed to CBZ (N = 3598) experiencing more major malformations than control children (N = 1691) (Analysis 1.1). The RD also suggested an increased risk with CBZ (RD 0.01, 95% CI 0.00 to 0.02; I^2^ = 1%) (Analysis 1.1).

####### 1.1.3 CBZ versus no medication (in women without epilepsy): routine health record studies

Results from two routine health record studies suggested no evidence of a difference in risk (RR 1.14 95% CI 0.80 to 1.64; I^2^ = 0%), with children exposed to CBZ (N = 983) experiencing a similar major malformation rate to control children (N = 372,111) (Analysis 1.1). The RD also suggested no difference in the level of risk (RD 0.00, 95% CI ‐0.01 to 0.01; I^2^ = 0%) (Analysis 1.1).

####### 1.1.4 CBZ versus no medication (in women with epilepsy): routine health record studies

Pooled results from four routine health record studies suggested an increased risk with CBZ (RR 1.42 95% CI 1.10 to 1.83; I^2^ = 0%), with children exposed to CBZ (N = 2116) experiencing more major malformations than control children (N = 12,218) (Analysis 1.1). The RD suggested an increased level of risk for CBZ (RD 0.01, 95% CI 0.00 to 0.02; I^2^ = 0%) (Analysis 1.1).

###### 1.2 Neural tube malformations

####### 1.2.1 CBZ versus no medication (in women without epilepsy): cohort studies

Pooled results from seven cohort studies suggested no evidence of a difference in risk (RR 3.09, 95% CI 0.38 to 25.40; I^2^ = 0%), with no difference in the number of neural tube malformations in children exposed to CBZ (N = 269) and compared to control children (N = 1801) (Analysis 1.2). The RD also suggested no difference in the level of risk (RD 0.00, 95% CI ‐0.01 to 0.02; I^2^ = 0%) (Analysis 1.2).

####### 1.2.2 CBZ versus no medication (in women with epilepsy): cohort studies

Pooled results from nine cohort studies suggested a comparable level of risk (RR 2.54, 95% CI 0.63 to 10.20; I^2^ = 0%), with no difference in the number of neural tube malformations in children exposed to CBZ (N = 1194) and in control children (N = 679) (Analysis 1.2). The RD also suggested no difference in the level of risk (RD 0.01, 95% CI ‐0.01 to 0.02; I^2^ = 0%) (Analysis 1.2).

####### 1.2.3 CBZ versus no medication (in women without epilepsy): routine health record studies

There were no studies that provided data for this comparison.

####### 1.2.4 CBZ versus no medication (in women with epilepsy): routine health record studies

There were no studies that provided data for this comparison.

###### 1.3 Cardiac malformations

####### 1.3.1 CBZ versus no medication (in women without epilepsy): cohort studies

Pooled results from seven cohort studies suggested no evidence of a difference in risk (RR 1.46, 95% CI 0.43 to 4.99; I^2^ = 0%), with no difference in the number of cardiac malformations in children exposed to CBZ (N = 269) and in control children (N = 1801) (Analysis 1.3). The RD also suggested no difference in the level of risk (RD ‐0.00, 95% CI ‐0.02 to 0.01; I^2^ = 0%) (Analysis 1.3).

####### 1.3.2 CBZ versus no medication (in women with epilepsy): cohort studies

Pooled results from 11 cohort studies suggested no evidence of a difference in risk (RR 0.87, 95% CI 0.41 to 1.84; I^2^ = 0%), with no difference in the number of cardiac malformations in children exposed to CBZ (N = 1212) and control children (N = 691) (Analysis 1.3). The RD also suggested no difference in the level of risk (RD ‐0.00, 95% CI ‐0.02 to 0.01; I^2^ = 0%) (Analysis 1.3).

####### 1.3.3 CBZ versus no medication (in women without epilepsy): routine health record studies

There were no studies that provided data for this comparison.

####### 1.3.4 CBZ versus no medication (in women with epilepsy): routine health record studies

There were no studies that provided data for this comparison.

###### 1.4 Oro‐facial cleft/craniofacial malformations

####### 1.4.1 CBZ versus no medication (in women without epilepsy): cohort studies

Pooled results from seven cohort studies suggested an increased risk with CBZ (RR 9.04, 95% CI 2.16 to 37.87; I^2^ = 10%), with children exposed to CBZ (N = 269) experiencing more oro‐facial cleft/craniofacial malformations than control children (N = 1801) (Analysis 1.4). The RD suggested no difference in the level of risk (RD 0.01, 95% CI ‐0.01 to 0.03; I^2^ = 0%) (Analysis 1.4).

####### 1.4.2 CBZ versus no medication (in women with epilepsy): cohort studies

Pooled results from nine cohort studies suggested no evidence of a difference in risk (RR 0.99, 95% CI 0.27 to 3.62; I^2^ = 0%), with no difference in the number of oro‐facial cleft/craniofacial malformations in children exposed to CBZ (N = 709) and control children (N = 347) (Analysis 1.4). The RD also suggested no difference in the level of risk (RD 0.00, 95% CI ‐0.02 to 0.02; I^2^ = 0%) (Analysis 1.4).

####### 1.4.3 CBZ versus no medication (in women without epilepsy): routine health record studies

There were no studies that provided data for this comparison.

####### 1.4.4 CBZ versus no medication (in women with epilepsy): routine health record studies

There were no studies that provided data for this comparison.

###### 1.5 Skeletal/limb malformations

####### 1.5.1 CBZ versus no medication (in women with epilepsy): cohort studies

Pooled results from seven cohort studies suggested no evidence of a difference in risk (RR 5.13, 95% CI 0.52 to 50.67, I^2^ = 0%), with no difference in skeletal/limb malformations in children exposed to CBZ (N = 269) and control children (N = 1801) (Analysis 1.5). The RD also suggested a comparable level of risk (RD 0.00, 95% CI ‐0.01 to 0.02; I^2^ = 0%) (Analysis 1.5).

####### 1.5.2 CBZ versus no medication (in women with epilepsy): cohort studies

Pooled results from nine cohort studies suggested no evidence of a difference in risk (RR 0.96, 95% CI 0.35 to 2.82; I^2^ = 0%), with no difference in the number of skeletal and limb malformations in children exposed to CBZ (N = 1194) and control children (N = 679) (Analysis 1.5). The RD also suggested no difference in the level of risk (RD 0.00, 95% CI ‐0.01 to 0.01; I^2^ = 0%) (Analysis 1.5).

####### 1.5.3 CBZ versus no medication (in women without epilepsy): routine health record studies

There were no studies that provided data for this comparison.

####### 1.5.4 CBZ versus no medication (in women with epilepsy): routine health record studies

There were no studies that provided data for this comparison.

##### Carbamazepine dose

The EURAP 2018 collaboration has reported on the largest uniformly assessed group of children exposed to CBZ (N = 1957). They reported a higher malformation rate with higher doses of CBZ. Doses =/< 700 mg/d were found to have a malformation risk of 4.5% (95% CI 3.5% to 5.8%), whilst dose > 700 mg/d were associated with a prevalence of 7.2%, (95% CI 5.4 to 9.4); a difference which suggested a dose association (OR 1.56, 95% CI 1.03 to 2.37, P = 0.0352). When compared to children exposed to =/< 325 mg/d of LTG, the prevalence was higher for doses =/< 700 mg/d (OR 1.71 95% CI 1.12 to 2.61, P = 0.0143), and doses over 700 mg/d were also higher (OR 2.68, 95% CI 1.71 to 4.19, P = 0.0002). In contrast, however, the North American Epilepsy and Pregnancy Register (N = 1033) failed to document an association between the risk of major malformation and the dose of CBZ; however, this group was smaller. The Australian Epilepsy and Pregnancy Register, the UK and Ireland Epilepsy and Pregnancy Register, and a number of smaller studies also did not identify a dose effect (Kaaja 2003; Kaneko 1999; Milan Study 1999; Motherisk Registry; Samren 1997).

Data regarding the impact of dose are limited from routine healthcare record‐based studies. Data analyses from Finland Health Record Registers did not establish a dose relationship, however, the number of carbamazepine monotherapy cases was small (N = 32). Results from the Norwegian Health Record Registers and Sweden Health Record Registers did not capture ASM doses, and researchers using the UK Health Record THIN Register or the UK Clinical Research Practice Database were not able to access dose information. Dose data have not currently been provided by the Denmark Health Record Registers for CBZ dose.

#### Clonazepam

##### 2 CZP versus controls

###### 2.1 All major malformations

The prevalence of major malformations (any type) in cohort studies for children exposed to clonazepam (CZP) (N = 95), based on data from four studies, was 2.1% (95% CI 0.2 to 17.3). The prevalence of major malformations in routine health record studies for children exposed to CZP (N = 161), based on data from one study, was 2.5% (95% CI 0.0 to 131.8).

####### 2.1.1 CZP versus no medication (in women without epilepsy): cohort studies

Pooled results from two cohort studies suggested no evidence of a difference in risk (RR 2.76, 95% CI 0.55 to 13.94; I^2^ = 0%), with children exposed to CZP (N = 65) experiencing comparable rates of major malformations to control children (N = 504) (Analysis 2.1). The RD also suggested no difference in the level of risk (RD 0.02, 95% CI ‐0.03 to 0.07; I^2^ = 0%) (Analysis 2.1).

####### 2.1.2 CZP versus no medication (in women with epilepsy): cohort studies

Pooled findings from three cohort studies suggested no evidence of a difference in risk (RR 1.08, 95% CI 0.21 to 5.42; I^2^ = 0%), with children exposed to CZP (N = 31) experiencing comparable rates of major malformations to control children (N = 524) (Analysis 2.1). The RD also suggested no difference in the level of risk (RD ‐0.03, 95% CI ‐0.11 to 0.04; I^2^ = 0%) (Analysis 2.1).

####### 2.1.3 CZP versus no medication (in women without epilepsy): routine health record studies

One study suggested no evidence of a difference in risk (RR 0.70, 95% CI 0.18 to 2.77; I^2^ = NA (not available)) with children exposed to CZP (N = 113) experiencing comparable rates of major malformations to control children (N = 369,267). The RD also suggested no difference in the level of risk (RD ‐0.01, 95% CI ‐0.03 to 0.02; I^2^ = NA) (Analysis 2.1).

####### 2.1.4 CZP versus no medication (in women with epilepsy): routine health record studies

One study suggested no evidence of a difference in risk (RR 0.69, 95% CI 0.17 to 2.79; I^2^ = NA) with children exposed to CZP (N = 113) experiencing comparable rates of major malformations to control children (N = 1900). The RD also suggested no difference in the level of risk (RD ‐0.01, 95% CI ‐0.03 to 0.02; I^2^ = NA) (Analysis 2.1).

Specific malformation types were not reviewed due to the small amount of data.

###### CZP Dose

There is too little experience with CZP in pregnancy to be able to report on the potential of an association between the dose of CZP and MCM risk.

#### Gabapentin

The prevalence of major malformations (any type) in cohort studies for children exposed to gabapentin (GBP) (N = 192) based on data from four studies was 2.0% (95% CI 0.1 to 32.2). The prevalence of major malformations in routine health record studies for children exposed to GBP (N = 18), was based on data from one study and therefore could not be calculated.

##### 3 GBP versus controls

###### 3.1 All major malformations

####### 3.1.1 GBP versus no medication (in women without epilepsy): cohort studies

Pooled results from two cohort studies suggested no evidence of a difference in risk of major malformations for the children exposed to gabapentin (N = 147) in comparison to children born to women without epilepsy (N = 570) (RR 1.78, 95% CI 0.50 to ‐6.29, P = 0.37, I^2^ = 89%), but there was heterogeneity in the results (Analysis 3.1). A random‐effects RR was calculated which also suggested a comparable level of risk (RR 8.04, 95% CI 0.03 to 1898.73, P = 0.45, I^2^ = 89%). The RD also suggested no difference in the level of risk (RD 0.00, 95% CI −0.02 to 0.03; I^2^ = 75%). Due to heterogeneity, a random‐effects RD was calculated which also found a comparable level of risk (RD 0.19, 95% CI ‐0.37 to 0.74, P = 0.51, I^2^ = 75%) (Analysis 3.1).

####### 3.1.2 GBP versus no medication (in women with epilepsy): cohort studies

Pooled results from two cohort studies suggested no evidence of a difference in risk of major malformation for the children exposed to gabapentin (n=47) in comparison to control children (n= 721) (RR 1.77, 95% CI 0.46 to 6.90, P = 0.41, I^2^ = 0% (Analysis 3.1).

####### 3.1.3 GBP versus no medication (in women without epilepsy): routine health record studies

There were no studies that provided data for this comparison in a format that could be combined in a meta‐analysis. However, Patorno and colleagues (US Medicaid Registers) conducted a sensitivity analysis that was restricted to epilepsy indications and included 347 pregnancies exposed to gabapentin in comparison to an unexposed reference group of 11,861 pregnancies. There was no reported difference in the malformation outcome either in the epilepsy subgroup (RR 1.40, 95% CI 0.73 to 2.71, P = 0.31) or in the main analysis which included 3745 gabapentin‐exposed children (RR 1.07, 95% CI 0.94 to 1.21, P = 0.33).

####### 3.1.4 GBP versus no medication (in women with epilepsy): routine health record studies

There were no studies that provided data for this comparison.

###### 3.2 Neural tube malformations

####### 3.2.1 GBP versus no medication (in women without epilepsy): cohort studies

We were unable to estimate a RR for the included study due to there being no reported neural tube malformations in children exposed to GBP (N = 2) or control children (N = 128) (Analysis 3.2).

####### 3.2.2 GBP versus no medication (in women with epilepsy): cohort studies

There were no studies that provided data for this comparison.

####### 3.2.3 GBP versus no medication (in women without epilepsy): routine health record data studies

There were no studies that provided data for this comparison.

####### 3.2.4 GBP versus no medication (in women with epilepsy): routine health record data studies

There were no studies that provided data for this comparison.

###### 3.3 Cardiac malformations

####### 3.3.1 GBP versus no medication (in women without epilepsy): cohort studies

Data from one study suggested a difference in risk (RR 129.00, 95% CI 6.49 to 2562.48, I^2^ = NA) with children exposed to GBP (N = 2) being at higher risk than control children (N = 128) (Analysis 3.3). However, the RD suggested no difference in the level of risk (RD 0.50, 95% CI ‐0.07 to 1.07; I^2^ = NA)

####### 3.3.2 GBP versus no medication (in women with epilepsy): cohort studies

Included studies did not reach the threshold for reporting in the meta‐analysis (Analysis 3.3). However, available data showed that there was one case of cardiac malformation in children exposed to GBP (N = 2) in comparison to zero cases in the control children (N = 4), based on data from one study (Miskov 2016).

####### 3.3.3 GBP versus no medication (in women without epilepsy): routine health record data studies

Patorno and colleagues, using data including the US Medicaid Registers, found a comparable level of risk for cardiac anomalies in children exposed to gabapentin (N = 347) versus children born to women without epilepsy (N = 11,861) (RR 1.40, 95% CI 0.73 to 2.71, P = 0.31).

####### 3.3.4 GBP versus no medication (in women with epilepsy): routine health record data studies

There were no studies that provided data for this comparison.

###### 3.4 Oro‐Facial Cleft/Craniofacial malformations

####### 3.4.1 GBP versus no medication (in women without epilepsy): cohort studies

We were unable to estimate a RR from one study due to there being no oro‐facial cleft / craniofacial malformations in children exposed to GBP (n=2) in comparison to no cases in 128 control children (Analysis 3.4).

####### 3.4.2 GBP versus no medication (in women with epilepsy): cohort studies

There were no studies that provided data for this comparison.

####### 3.4.3 GBP versus no medication (in women without epilepsy): routine health record data studies

There were no studies that provided data for this comparison.

####### 3.4.4 GBP versus no medication (in women with epilepsy): routine health record data studies

There were no studies that provided data for this comparison.

###### 3.5 Skeletal/Limb malformations

####### 3.5.1 GBP versus no medication (in women without epilepsy): cohort studies

We were unable to estimate a RR from one study due to there being no reported skeletal/limb malformations in children exposed to GBP (n=2) or 128 control children, based on data from one study (Analysis 3.5).

####### 3.5.2 GBP versus no medication (in women with epilepsy): cohort studies

There were no studies that provided data for this comparison.

####### 3.5.3 GBP versus no medication (in women without epilepsy): routine health record data studies

There were no studies that provided data for this comparison.

####### 3.5.4 GBP versus no medication (in women with epilepsy): routine health record data studies

There were no studies that provided data for this comparison.

##### Gabapentin dose

The investigation of GBP dose and its potential association with an increased rate of malformations is limited due to the relatively small number of pregnancies where data are currently available. The US Medicaid Registers is the most reliable data source currently available. The study authors did not find that malformation risk increased with dose according to tertiles of the first and the highest prescribed daily dose filled. Doses of 600 mg/d through to 900 mg/d (RR 1.00, 95% CI 0.80 to 1.24, P = 0.98) or doses above 900 mg/d (RR 1.17, 95% CI 0.95 to 1.44, P = 0.15) were not associated with a risk above the baseline risk. The largest cohort study of GBP‐exposed pregnancies was from the North American Epilepsy and Pregnancy Register (N = 145) and no association between increasing dose and increased malformation risk was identified in this study. The participant numbers in other included studies of GBP were too small to investigate any effect of dose size and MCM risk.

#### Levetiracetam

The prevalence of major malformations (any type) in cohort studies for children exposed to levetiracetam (LEV) (N = 1242), based on data from 11 studies, was 2.6% (95% CI 1.6 to 4.4). The prevalence of major malformations in routine health record studies for children exposed to LEV (N = 248), based on data from two studies, was 2.8% (95% CI 0.0 to 321.9).

##### 4 LEV versus controls

###### 4.1 All major malformations

####### 4.1.1 LEV versus no medication (in women without epilepsy): cohort studies

Pooled results from four cohort studies suggested no evidence of a difference in risk (RR 2.20, 95% CI 0.98 to 4.93; I^2^ = 0%), with children exposed to LEV (N = 574) experiencing comparable rates of major malformations to control children (N = 1022) (Analysis 4.1). The RD also suggested no difference in the level of risk (RD 0.01, 95% CI −0.00 to 0.03; I^2^ = 0%).

####### 4.1.2 LEV versus no medication (in women with epilepsy): cohort studies

Pooled results from six cohort studies suggested no evidence of a difference in risk (RR 0.71, 95% CI 0.39 to 1.28; I^2^ = 0%), with children exposed to LEV (N = 724) experiencing comparable rates of major malformations to control children (N = 1101) (Analysis 4.1). The RD also suggested no difference in the level of risk (RD −0.01, 95% CI −0.03 to 0.00; I^2^ = 0%).

####### 4.1.3 LEV versus no medication (in women without epilepsy): routine health record data studies

One study suggested no evidence of a difference in risk (RR 0.67, 95% CI 0.17 to 2.66; I^2^ = NA) for children exposed to LEV (N = 118) experiencing comparable rates of major malformations to control children (N = 369,267). The RD also suggested no difference in the level of risk (RD ‐0.01, 95% CI ‐0.03 to 0.02; I^2^ = 0%).

####### 4.1.4 LEV versus no medication (in women with epilepsy): routine health record data studies

Pooled results from two routine health record studies suggested no evidence of a difference in risk (RR 0.82, 95% CI 0.39 to 1.71; I^2^ = 0%), with children exposed to LEV (N = 248) experiencing comparable rates of major malformations to control children (N = 10,377) (Analysis 4.1). The RD also suggested no difference in the level of risk (RD −0.01, 95% CI −0.03 to 0.01; I^2^ = 0%).

###### 4.2 Neural tube malformations

####### 4.2.1 LEV versus no medication (in women without epilepsy): cohort studies

We were unable to estimate a RR from two studies due to there being no reported cases of neural tube malformation in children exposed to LEV (N = 105) or control children (N = 383) (Analysis 4.2).

####### 4.2.2 LEV versus no medication (in women with epilepsy): cohort studies

We were unable to estimate a RR from two studies due to there being no reported cases of neural tube malformations in children exposed to LEV (N = 173) or control children (N = 361) (Analysis 4.2).

####### 4.2.3 LEV versus no medication (in women without epilepsy): routine health record data studies

No included studies reported data on this outcome.

####### 4.2.4 LEV versus no medication (in women with epilepsy): routine health record data studies

No included studies reported data on this outcome.

###### 4.3 Cardiac malformations

####### 4.3.1 LEV versus no medication (in women without epilepsy): cohort studies

Pooled results from two cohort studies suggested no evidence of a difference in risk (RR 3.92, 95% CI 0.57 to 27.07; I^2^ = 0), with children exposed to LEV (N = 105) experiencing comparable rates of major malformations to control children (N = 383) (Analysis 4.3). The RD also suggested no difference in the level of risk (RD 0.02, 95% CI −0.02 to 0.06; I^2^ = 0%).

####### 4.3.2 LEV versus no medication (in women with epilepsy): cohort studies

Pooled results from four cohort studies suggested no evidence of a difference in risk (RR 0.90, 95% CI 0.31 to 2.60; I^2^ = 0), with children exposed to LEV (N = 281) experiencing comparable rates of major malformations to control children (N = 384) (Analysis 4.3). The RD also suggested no difference in the level of risk (RD 0.00, 95% CI −0.03 to 0.03; I^2^ = 0%).

####### 4.3.3 LEV versus no medication (in women without epilepsy): routine health record data studies

No included studies reported data on this outcome.

####### 4.3.4 LEV versus no medication (in women with epilepsy): routine health record data studies

No included studies reported data on this outcome.

###### 4.4 Oro‐facial cleft/craniofacial malformations

####### 4.4.1 LEV versus no medication (in women without epilepsy): cohort studies

We were unable to estimate a RR from two studies due to there being no reported oro‐facial cleft/craniofacial malformations in children exposed to LEV (N = 105) or control children (N = 383) (Analysis 4.4).

####### 4.4.2 LEV versus no medication (in women with epilepsy): cohort studies

Pooled results from three studies suggested no evidence of a difference in risk (RR 0.14, 95% CI 0.01 to 3.18; I^2^ = N/A), with children exposed to LEV (N=186) experiencing comparable rates of oro‐facial cleft/craniofacial malformations as control children (N=44) (Analysis 4.4).

####### 4.4.3 LEV versus no medication (in women without epilepsy): routine health record data studies

No included studies reported data on this outcome.

####### 4.4.4 LEV versus no medication (in women with epilepsy): routine health record data studies

No included studies reported data on this outcome.

###### 4.5 Skeletal/limb malformations

####### 4.5.1 LEV versus no medication (in women without epilepsy): cohort studies

We were unable to estimate a RR from two studies due to there being no skeletal / limb malformations in children exposed to LEV (N = 105) or control children (N = 383) (Analysis 4.5).

####### 4.5.2 LEV versus no medication (in women with epilepsy): cohort studies

Pooled results from three studies suggested no evidence of a difference in risk (RR 3.21, 95% CI 0.46 to 22.50; I^2^ = NA), with children exposed to LEV (N = 272) experiencing comparable rates of skeletal/limb malformations to control children (N = 376) (Analysis 4.5). The RD also suggested no difference in the level of risk (RD 0.01, 95% CI −0.02 to 0.03; I^2^ = 0%).

####### 4.5.3 LEV versus no medication (in women without epilepsy): routine health record data studies

No included studies reported data on this outcome.

####### 4.5.4 LEV versus no medication (in women with epilepsy): routine health record data studies

No included studies reported data for this outcome.

##### Levetiracetam dose

EURAP 2018 had the largest cohort of LEV‐exposed children to conduct dose investigations in 599 exposed children. Whilst they did not make comparisons between different levels of LEV dose directly, they did report that there was evidence of lower risk of any LEV dose (250‐4000 mg/d) in comparison to doses of VPA </= 650 mg/d and dose of CBZ > 700 mg/d, whilst there was no evidence of difference in comparison to doses of LTG either at </= 325 mg/d or > 325 mg/d, or in comparison to OXC at doses ranging from 75‐4500 mg/d. Additionally, the North American Epilepsy and Pregnancy Register reporting on LEV‐exposed children (N = 450), the UK and Ireland Epilepsy and Pregnancy Register (N = 304), the Australian Epilepsy and Pregnancy Register (N = 139), the Kerala Epilepsy and Pregnancy Registry (N = 106) and the MONEAD 2020 study (N = 99) also failed to find an association between increasing doses of LEV and congenital anomaly risk; however, group sizes may still be too limited at higher dose levels to detect increased levels of MCM risk.

#### Lamotrigine

The prevalence of major malformations (any type) in cohort studies for children exposed to lamotrigine (LTG) (N = 4704), based on data from 15 studies, was 2.7% (95% CI 1.9 to 3.8). The prevalence of major malformations in routine health record studies for children exposed to LTG (N = 2502), based on data from four studies, was 3.5% (95% CI 2.5 to 4.9).

##### 5 LTG versus controls

###### 5.1 All major malformations

####### 5.1.1 LTG versus no medication (in women without epilepsy): cohort studies

Pooled results from seven studies suggested an increased risk with LTG (RR 1.97, 95% CI 1.16 to 3.39; I^2^ = 0%), with children exposed to LTG (N = 1899) experiencing more major malformations to control children (N = 2693) (Analysis 5.1). The RD also suggested a higher risk (RD 0.01, 95% CI 0.00 to 0.03; I^2^ = 0%).

####### 5.1.2 LTG versus no medication (in women with epilepsy): cohort studies

Pooled results from eight studies suggested no evidence of a difference in risk (RR 1.04, 95% CI 0.66 to 1.63; I^2^ = 0%), with children exposed to LTG (N = 2767) experiencing comparable rates of major malformations to control children (N = 1151) (Analysis 5.1). The RD also suggested no difference in the level of risk (RD 0.00, 95% CI −0.01 to 0.01; I^2^ = 0%).

####### 5.1.3 LTG versus no medication (in women without epilepsy): routine health record data studies

Pooled results from two studies suggested no evidence of a difference in risk (RR 1.19, 95% CI 0.86 to 1.64; I^2^ = 18%), with children exposed to LTG (N = 1177) experiencing comparable rates of major malformations to control children (N = 372,111) (Analysis 5.1). The RD also suggested no difference in the level of risk (RD 0.01, 95% CI −0.01 to 0.02; I^2^ = 22%).

####### 5.1.4 LTG versus no medication (in women with epilepsy): routine health record data studies

Pooled results from three studies suggested no evidence of a difference in risk (RR 1.00, 95% CI 0.79 to 1.28; I^2^ = 0%), with children exposed to LTG (N = 2166) experiencing comparable rates of major malformations to control children (N = 11,279) (Analysis 5.1). The RD also suggested no difference in the level of risk (RD ‐0.00, 95% CI −0.01 to 0.01; I^2^ = 0%).

###### 5.2 Neural tube malformations

####### 5.2.1 LTG versus no medication (in women without epilepsy)

Pooled results from five studies suggested an increased risk with LTG (RR 7.55, 95% CI 1.05 to 54.09; I^2^ = 0%), with children exposed to LTG (N = 313) experiencing more major malformations to control children (N = 1654) (Analysis 5.2). However, the RD suggested no difference in the level of risk (RD 0.00, 95% CI −0.01 to 0.02; I^2^ = 0%).

####### 5.2.2 LTG versus no medication (in women with epilepsy)

We were unable to estimate a RR from five studies, as there were no reported neural tube malformations in children exposed to LTG (N = 521) or control children (N = 563) (Analysis 5.2).

####### 5.2.3 LTG versus no medication (in women without epilepsy): routine health record data studies

No included studies reported data on this outcome.

####### 5.2.4 LTG versus no medication (in women with epilepsy): routine health record data studies

No included studies reported data on this outcome.

###### 5.3 Cardiac malformations

####### 5.3.1 LTG versus no medication (in women without epilepsy): cohort studies

Pooled results from six studies suggested an increased risk with LTG (RR 2.71, 95% CI 1.05 to 6.98; I^2^ = 0%), with children exposed to LTG (N = 348) experiencing more major malformations to control children (N = 1658) (Analysis 5.3). However, the RD suggested no difference in the level of risk (RD 0.01, 95% CI −0.01 to 0.03; I^2^ = 0%).

####### 5.3.2 LTG versus no medication (in women with epilepsy): cohort studies

Pooled results from six studies suggested no evidence of a difference in risk (RR 0.97, 95% CI 0.28 to 3.32; I^2^ = 0%), with children exposed to LTG (N = 541) experiencing comparable rates of major malformations to control children (N = 571) (Analysis 5.3). However, the RD suggested no difference in the level of risk (RD 0.00, 95% CI −0.02 to 0.02; I^2^ = 0%).

####### 5.3.3 LTG versus no medication (in women without epilepsy): routine health record data studies

No included studies reported data for this outcome.

####### 5.3.4 LTG versus no medication (in women with epilepsy): routine health record data studies

No included studies reported data for this outcome.

###### 5.4 Oro‐facial cleft/craniofacial malformations

####### 5.4.1 LTG versus no medication (in women without epilepsy): cohort studies

We were unable to estimate RR from the four included studies due to there being no reported oro‐facial cleft/craniofacial malformations in children exposed to LTG (N = 197) or control children (N = 826) (Analysis 5.4).

####### 5.4.2 LTG versus no medication (in women with epilepsy): cohort studies

Pooled results from five studies suggested no evidence of a difference in risk (RR 1.37, 95% CI 0.29 to 6.56; I^2^ = 65%), with no difference in the number of oro‐facial cleft/craniofacial malformations in children exposed to LTG (N = 491) and control children (N = 322) (Analysis 5.4). Due to high heterogeneity, a random‐effects RR was calculated which also found no difference (RR 0.90, 95% CI 0.03 to 32.04, P = 0.95, I^2^ = 65%). The RD suggested no difference in the level of risk (RD 0.00, 95% CI −0.01 to 0.02; I^2^ = 0%).

####### 5.4.3 LTG versus no medication (in women without epilepsy): routine health record data studies

In the study using the US Medicaid Registers by Hernandez‐Diaz and colleagues, there was no evidence of a difference in the oral cleft rates for children exposed to LTG (N = 2796) in comparison to the children born to women without epilepsy (N = 1,322,955) (RR 1.89, 95% CI 0.85 to 4.21).

####### 5.4.4 LTG versus no medication (in women with epilepsy): routine health record data studies

No included studies reported data for this outcome.

###### 5.5 Skeletal/limb malformations

####### 5.5.1 LTG versus no medication (in women without epilepsy): cohort studies

Pooled results from five studies suggested an increased risk with LTG (RR 11.29, 95% CI 2.37 to 53.91; I^2^ = 0%), with children exposed to LTG (N = 311) experiencing more major malformations to control children (N = 1654) (Analysis 5.5). However, the RD suggested no difference in the level of risk (RD 0.01, 95% CI −0.00 to 0.03; I^2^ = 0%).

####### 5.5.2 LTG versus no medication (in women with epilepsy): cohort studies

Pooled results from five studies suggested no evidence of a difference in risk (RR 0.75, 95% CI 0.20 to 2.89; I^2^ = 0%), with no difference in the number of skeletal/limb malformations in children exposed to LTG (N = 521) and control children (N = 563) (Analysis 5.5). The RD also suggested no difference in the level of risk (RD 0.00, 95% CI −0.02 to 0.01; I^2^ = 0%).

####### 5.5.3 LTG versus no medication (in women without epilepsy): routine health record data studies

No included studies reported data for this outcome.

####### 5.5.4 LTG versus no medication (in women with epilepsy): routine health record data studies

No included studies reported data for this outcome.

##### Lamotrigine dose

The EURAP 2018 collaboration has reported on a large, uniformly assessed, group of children exposed to LTG (N = 2514). It reported a higher MCM rate with higher doses of LTG. Doses =/< 325 mg/d were found to have an MCM risk of 2.5% (95% CI 1.8% to 3.3%), whilst doses > 325 mg/d were associated with MCM in 4.3% of children (95% CI 2.9% to 6.2%); a difference which suggested a dose association (OR 1.68, 95% CI 1.01 to 2.80, P = 0.0463).

When EURAP 2018 compared lower dose LTG (=/< 325 mg/d) to other monotherapy ASMs, they found evidence suggesting a lower MCM risk in comparison to CBZ at =/< 700 mg/d (OR 1.71 95% CI 1.12 to 2.61, P = 0.0143) and lower risk than CBZ doses > 700 mg/d (OR 2.68, 95% CI 1.71 to 4.19, P = 0.0002). In comparison to LEV, there was no evidence of a difference between lower doses of LTG (</= 325 mg/d) and LEV doses between =/> 250‐4000 mg/d (OR 1.11, 95% CI 0.62 to 2.00, P = 0.7282). Comparisons to VPA demonstrated lower MCM risks for lower LTG dose (=/< 325 mg/d) in comparison to VPA doses at =/< 650 mg/d (OR 2.70, 95% CI 1.67 to 4.38, P = 0.0002), > 650 mg/d to =/< 1450 mg/d (OR 4.72, 95% CI 3.11 to 7.18, P < 0.0002), or at doses of VPA > 1450 mg/d (OR 13.52, 95% CI 7.73 to 23.64, P = 0.0002). Exposure to LTG at a dose =/< 325 mg daily was associated with a lower MCM risk than PB exposure at doses of between > 80 and =/< 130 mg/d (OR 2.46, 95% CI 1.16 to 5.23, P = 0.0196) and at PB doses > 130 mg/d (OR 5.81, 95% CI 2.40 to 14.08, P = 0.0002). There was, however, no evidence of a difference in comparison of LTG doses =/< 325 mg/d to the lowest investigated PB dose of =/< 80 mg/d (OR 1.07, 95% CI 0.25 to 4.60, P = 0.923). Rates of PHT, TPM, and OXC‐exposed pregnancies were lower in the EURAP study which should be considered with regard to findings suggesting that there is no dose association here. In comparison to lower dose LTG (=/< 325 mg/d), there was no evidence of difference for PHT doses between =/30 mg/d and 730 mg/d (OR 1.93, 95% CI 0.78 to 4.75, P = 0.1554) or TPM doses =/> 25 mg/d to 500 mg/d (OR 1.67, 95% CI 0.69 to 4.04, P = 0.2524) or OXC doses between =/> 75 to 4500 mg/d (OR 1.13, 95% CI 0.55 to 2.31, P = 0.7358).

The EURAP 2018 collaboration also compared higher doses of LTG (> 325 mg/d) and found a comparable level of risk to higher doses of CBZ (> 700 mg/d, OR 0.63, 95% CI 0.38 to 1.05, P = 0.0766), to LEV doses between =/> 250‐4000 mg/d (OR 1.51, 95% CI 0.79 to 2.88, P = 0.2077) and to OXC doses between 75‐4500 mg/d (OR 1.49, 95% CI 0.70 to 3.17, P = 0.3051). Higher doses of LTG (> 325 mg/d) were not associated with lower rates of MCM compared to the lowest investigated dose range for VPA (=/< 650 mg/d, OR 0.62, 95% CI 0.36 to 1.09, P = 0.0959) but there was evidence suggesting higher doses of LTG were associated with a lower MCM risk than VPA doses between > 650 to =/< 1450 mg/d (OR 2.81, 95% 1.70 to 4.65, P = 0.0002).

In contrast to the data from EURAP, the UK and Ireland Epilepsy and Pregnancy Register (N = 2198) found no evidence of risk with increasing doses of LTG (0 to 200 mg/d vs 200 to 400 mg/d; 0 to 200 mg/d vs > 400 mg/d). The North American Epilepsy and Pregnancy Register (N = 1562), the Australian Epilepsy and Pregnancy Register (N = 406), and the Israeli Teratogen Service (N = 114) studies did not identify dose‐related risks associated with LTG. The frequency of MCM was too low in other included studies to allow reliable investigation of dose.

#### Oxcarbazepine

The prevalence of major malformations (any type) in cohort studies for children exposed to oxcarbazepine (OXC) (N = 378), based on data from 11 studies, was 2.8% (95% CI 1.1 to 6.6). The prevalence of major malformations in routine health record studies for children exposed to OXC (N = 507), based on data from four studies, was 4.8% (95% CI 0.7 to 31.5).

##### 6 OXC versus controls

###### 6.1 All major malformations

####### 6.1.1 OXC versus no medication (in women without epilepsy): cohort studies

Pooled results from two studies suggested no evidence of a difference in risk (RR 2.20, 95% CI 0.67 to 7.27; I^2^ = 18%), with children exposed to OXC (N = 184) experiencing comparable rates of major malformations to control children (N = 767) (Analysis 6.1). The RD also suggested no difference in the level of risk (RD 0.01, 95% CI −0.02 to 0.04; I^2^ = 0%).

####### 6.1.2 OXC versus no medication (in women with epilepsy): cohort studies

Pooled results from six studies suggested no evidence of a difference in risk (RR 1.40, 95% CI 0.68 to 2.91; I^2^ = 23%), with children exposed to OXC (N = 134) experiencing comparable rates of major malformations to control children (N = 788) (Analysis 6.1). The RD also suggested no difference in the level of risk (RD 0.02, 95% CI −0.03 to 0.07; I^2^ = 0%).

####### 6.1.3 OXC versus no medication (in women without epilepsy): routine health record data studies

Results from one study found no evidence of a difference in risk (RR 0.70, 95% CI 0.10 to 4.86; I^2^ = N/A), with children exposed to OXC (N = 57) experiencing comparable rates of major malformations to control children (N = 369,267) (Analysis 6.1). The RD also suggested no difference in the level of risk (RD ‐0.01, 95% CI −0.04 to 0.03; I^2^ = N/A).

####### 6.1.4 OXC versus no medication (in women with epilepsy): routine health record data studies

Pooled results from three studies suggested an increased risk with OXC (RR 1.75, 95% CI 1.22 to 2.52; I^2^ = 94%), with children exposed to OXC (N = 503) experiencing higher rates of major malformations than control children (N = 11,316) (Analysis 6.1). Due to high heterogeneity, a random‐effects RR was calculated and found no evidence of a difference in risk (RR 1.61, 95% CI 0.26 to 9.86; I^2^ = 94%). The RD suggested a higher risk for OXC (RD 0.03, 95% CI 0.01 to 0.05; I^2^ = 94%); however, a random‐effects RD due to heterogeneity found no difference in the level of risk (RD 0.04, 95% CI ‐0.05 to 0.12; I^2^ = 94%).

###### 6.2 Neural tube malformations

####### 6.2.1 OXC versus no medication (in women without epilepsy): cohort studies

No included studies reported data for this outcome.

####### 6.2.2 OXC versus no medication (in women with epilepsy): cohort studies

We were unable to estimate a RR from the two included studies due to there being no reported neural tube malformations in children exposed to OXC (N = 102) or control children (N = 361) (Analysis 6.2).

####### 6.2.3 OXC versus no medication (in women without epilepsy): routine health record data studies

No included studies reported data for this outcome.

####### 6.2.4 OXC versus no medication (in women with epilepsy): routine health record data studies

No included studies reported data on this outcome.

###### 6.3 Cardiac malformations

####### 6.3.1 OXC versus no medication (in women without epilepsy): cohort studies

We were unable to estimate a RR in the included study due to there being no reported cardiac malformations in children exposed to OXC (N = 1) or control children (N = 128) (Analysis 6.3).

####### 6.3.2 OXC versus no medication (in women with epilepsy): cohort studies

Pooled results from four studies suggested no evidence of a difference in risk (RR 1.10, 95% CI 0.36 to 3.35; I^2^ = 22%), with children exposed to OXC (N = 106) experiencing comparable rates of major malformations to control children (N = 373) (Analysis 6.3). The RD also suggested no difference in the level of risk (RD 0.00, 95% CI −0.04 to 0.05; I^2^ = 0%).

####### 6.3.3 OXC versus no medication (in women without epilepsy): routine health record data studies

No included studies reported data on this outcome.

####### 6.3.4 OXC versus no medication (in women with epilepsy): routine health record data studies

No included studies reported data on this outcome.

###### 6.4 Oro‐facial cleft/craniofacial malformations

####### 6.4.1 OXC versus no medication (in women without epilepsy): cohort studies

No included studies reported data on this outcome.

####### 6.4.2 OXC versus no medication (in women with epilepsy): cohort studies

Included studies did not reach the threshold for reporting the meta‐analysis (Analysis 6.4). However, available data showed there were 0/34 cases of oro‐facial cleft/craniofacial malformations in children exposed to OXC and 1/29 cases in control children, based on data from two studies (AlSheikh 2020; Hosny 2021).

####### 6.4.3 OXC versus no medication (in women without epilepsy): routine health record data studies

No included studies reported data on this outcome.

####### 6.4.4 OXC versus no medication (in women with epilepsy): routine health record data studies

No included studies reported data on this outcome.

###### 6.5 Skeletal/limb malformations

####### 6.5.1 OXC versus no medication (in women without epilepsy): cohort studies

No included studies reported data on this outcome.

####### 6.5.2 OXC versus no medication (in women with epilepsy): cohort studies

Pooled data from two studies suggested no evidence of a difference in risk (RR 2.39, 95% CI 0.22 to 26.05; I^2^ = NA), with children exposed to OXC (N = 102) experiencing comparable rates of major malformations to control children (N = 361) (Analysis 6.5). The RD also suggested no difference in the level of risk (RD 0.01, 95% CI −0.02 to 0.03; I^2^ = 0%).

####### 6.5.3 OXC versus no medication (in women without epilepsy): routine health record data studies

No included studies reported data on this outcome.

####### 6.5.4 OXC versus no medication (in women with epilepsy): routine health record data studies

No included studies reported data on this outcome.

##### Oxcarbazepine dose

The limited published experience of OXC in pregnancy limits dose comparisons, even in the EURAP 2018 study for different doses of OXC (N = 333). In EURAP 2018, there was no evidence that doses of OXC between =/> 75 to 4500 mg/d were different from those for lower dose LTG (=/< 325 mg/d) (OR 1.13, 95% CI 0.55 to 2.31, P = 0.7282) or dose of LTG > 325 mg/d (OR 1.49, 95% CI 0.70 to 3.17, P = 0.3051). Similarly, a lack of difference was also found in comparison to any dose of LEV exposure (OR 1.02, 95% CI 0.45 to 2.30, P = 0.9644). A lower prevalence of MCM was found for any dose of OXC (3.0%, 95% CI 1.4 to 5.4) in comparison to low dose VPA (=/< 650 mg/d, 6.3%, 95% CI 4.5 to 8.6) (OR 2.39, 95% CI 1.13 to 5.08, P = 0.0235), but was not reported for any higher‐dose VPA.

Other studies were limited to the number of OXC‐exposed pregnancies or had not published dose data.

#### Phenobarbital

The prevalence of major malformations (any type) in cohort studies for children exposed to phenobarbital (PB) (N = 840), based on data from 26 studies, was 6.3% (95% CI 4.8 to 8.3). The prevalence of major malformations in routine health record studies for children exposed to PB (N = 34), based on data from two studies, was 8.8% (95% CI 0.0 to 9722.4).

##### 7 PB versus controls

###### 7.1 All major malformations

####### 7.1.1 PB versus no medication (in women without epilepsy): cohort studies

Pooled results from eight studies suggested an increased risk with PB (RR 3.22, 95% CI 1.84 to 5.65; I^2^ = 0%), with children exposed to PB (N = 353) experiencing more major malformations than control children (N = 2042) (Analysis 7.1). The RD also suggested a higher risk for PB (RD 0.04, 95% CI 0.01 to 0.07; I^2^ = 0%).

Samren 1997 reported five cases of major malformation out of 48 exposed infants (10%). Numbers were more limited in the comparison to control children (as not all centres in the study included control children), with just one malformation case out of six PB‐exposed children; analysis suggested no evidence of a difference between the groups (RR 2.4, 95% CI 0.3 to 23.0).

####### 7.1.2 PB versus no medication (in women with epilepsy): cohort studies

Pooled results from 13 studies suggested no evidence of a difference in risk (RR 1.64, 95% CI 0.94 to 2.83; I^2^ = 0%), with no difference in the number of major malformations in children exposed to PB (N = 438) and control children (N = 999) (Analysis 7.1). The RD also suggested no difference in the level of risk (RD 0.02, 95% CI −0.01 to 0.06; I^2^ = 0%).

####### 7.1.3 PB versus no medication (in women without epilepsy): routine health record data studies

The results from one study suggested no evidence of a difference in risk (RR 2.94, 95% CI 0.77 to 11.15; I^2^ = NA), with children exposed to PB (N = 27) experiencing comparable rates of major malformations to control children (N = 369,267) (Analysis 7.1). The RD also suggested no difference in the level of risk (RD 0.05, 95% CI −0.05 to 0.15; I^2^ = NA).

####### 7.1.4 PB versus no medication (in women with epilepsy): routine health record data studies

The results from one study suggested no evidence of a difference in risk (RR 2.87, 95% CI 0.74 to 11.21; I^2^ = NA), with children exposed to PB (N = 27) experiencing comparable rates of major malformations to control children (N = 1900) (Analysis 7.1). The RD also suggested no difference in the level of risk (RD 0.05, 95% CI −0.05 to 0.15; I^2^ = NA).

###### 7.2 Neural tube malformations

####### 7.2.1 PB versus no medication (in women without epilepsy): cohort studies

We were unable to estimate a RR from two studies due to there being no neural tube malformations in children exposed to PB (N = 7) or control children (N = 244) (Analysis 7.2).

####### 7.2.2 PB versus no medication (in women with epilepsy): cohort studies

Pooled results from three studies suggested no evidence of a difference in risk (RR 3.85, 95% CI 0.47 to 31.26, I^2^ = 0%), with no difference in the number of neural tube malformations in children exposed to PB (N = 146) and control children (N = 512) (Analysis 7.2). The RD also suggested no difference in the level of risk (RD 0.01, 95% CI −0.02 to 0.03; I^2^ = 0%).

####### 7.2.3 PB versus no medication (in women without epilepsy): routine health record data studies

No included studies reported data on this outcome.

####### 7.2.4 PB versus no medication (in women with epilepsy): routine health record data studies

No included studies reported data on this outcome.

###### 7.3 Cardiac malformations

####### 7.3.1 PB versus no medication (in women without epilepsy): cohort studies

Pooled results from two studies suggested no evidence of a difference in risk (RR 7.80, 95% CI 0.36 to 168.52, I^2^ = NA), with no difference in the number of cardiac malformations in children exposed to PB (N = 7) and control children (N = 244) (Analysis 7.3). The RD also suggested no difference in the level of risk (RD ‐0.00, 95% CI −0.27 to 0.26; I^2^ = 0%).

####### 7.3.2 PB versus no medication (in women with epilepsy): cohort studies

Pooled results from four studies suggested no evidence of a difference in risk (RR 1.80, 95% CI 0.69 to 4.71, I^2^ = 0%), with no difference in the number of cardiac malformations in children exposed to PB (N = 149) and control children (N = 516) (Analysis 7.3). The RD also suggested no difference in the level of risk (RD 0.02, 95% CI −0.02 to 0.05; I^2^ = 0%).

####### 7.3.3 PB versus no medication (in women without epilepsy): routine health record data studies

No included studies reported data on this outcome.

####### 7.3.4 PB versus no medication (in women with epilepsy): routine health record data studies

No included studies reported data on this outcome.

###### 7.4 Oro‐facial cleft/craniofacial malformations

####### 7.4.1 PB versus no medication (in women without epilepsy): cohort studies

Pooled results from two studies suggested no evidence of a difference in risk (RR 3.34, 95% CI 0.20 to 56.35, I^2^ = NA), with no difference in the number of oro‐facial cleft/craniofacial malformations in children exposed to PB (N = 7) and control children (N = 244) (Analysis 7.4). The RD also suggested no difference in the level of risk (RD ‐0.01, 95% CI −0.28 to 0.25; I^2^ = 0%).

####### 7.4.2 PB versus no medication (in women with epilepsy): cohort studies

We were unable to estimate a RR from two studies due to there being no reported oro‐facial cleft/craniofacial malformations in children exposed to PB (N = 9) or control children (N = 172) (Analysis 7.4).

####### 7.4.3 PB versus no medication (in women without epilepsy): routine health record data studies

No included studies reported data on this outcome.

####### 7.4.4 PB versus no medication (in women with epilepsy): routine health record data studies

No included studies reported data on this outcome.

###### 7.5 Skeletal/limb malformations

####### 7.5.1 PB versus no medication (in women without epilepsy): cohort studies

Pooled results from two studies suggested no evidence of a difference in risk (RR 7.80, 95% CI 0.36 to 168.52, I^2^ = NA) with no difference in the number of skeletal/limb malformations in children exposed to PB (N = 7) in comparison to control children (N=244). (Analysis 7.5). The RD also suggested no difference in the level of risk (RD ‐0.00, 95% CI −0.27 to 0.26; I^2^ = NA).

####### 7.5.2 PB versus no medication (in women with epilepsy): cohort studies

Pooled results from three studies suggested no evidence of a difference in risk (RR 3.01, 95% CI 0.56 to 16.07; I^2^ = 0%), with no difference in the number of skeletal/limb malformations in children exposed to PB (N = 146) and control children (N = 512) (Analysis 7.5). The RD also suggested no difference in the level of risk (RD 0.01, 95% CI −0.02 to 0.03; I^2^ = 0%).

####### 7.5.3 PB versus no medication (in women without epilepsy): routine health record data studies

No included studies reported data on this outcome.

####### 7.5.4 PB versus no medication (in women with epilepsy): routine health record data studies

No included studies reported data on this outcome.

##### Phenobarbital dose

Despite data being reported in 26 studies, most studies did not investigate dose or report the results of analyses of PB dose with regard to MCM risk or were too limited in terms of the number of included pregnancies. EURAP 2018 included 294 PB monotherapy‐exposed cases which is the largest cohort. They found that increasing PB dose was associated with an increasing prevalence of MCM risk. Doses =/< 80 mg/d had a prevalence of 2.7% (95% CI 0.3 to 9.5), doses > 80 to =/< 130 mg/d had a prevalence of 6.2% (95%CI 3.0 to 11.1), and doses > 130 mg/d had the highest prevalence of 11.7% (95% CI 4.8 to 22.6); there was evidence of a dose association the for comparison of the lowest and highest PB dose levels investigated (OR 5.41, 95% CI 1.05 to 27.89, P = 0.0436). PB doses > 130 mg/d were associated with a higher MCM risk than LTG at doses =/< 325 mg/d (OR 5.81, 95% CI 2.40 to 14.08, P = 0.0002). There were no comparisons of the different PB dose levels to other ASM doses, however. The Kerala Epilepsy and Pregnancy Registry reported on 137 pregnancies and demonstrated an increase in MCM risk with increasing dose; PB > 200 mg/d had a prevalence of 10.3% whilst PB doses > 45 to 60 mg/d had a prevalence of 3.5%. However, it is possible that there was some case overlap with the EURAP 2018 cases as the Kerala Epilepsy and Pregnancy Registry is a EURAP collaborator. The collaboration reported by Samren 1997 and colleagues reported a likely dose association with PB. The North American Epilepsy and Pregnancy Register included 199 PB‐exposed pregnancies and did not find an association with dose. Kaneko 1999 did find an association between PB exposure (N = 79) and increased malformation rate. Other studies were too small or did not investigate an association between PB dose and MCM risk.

#### Phenytoin

The prevalence of major malformations (any type) in cohort studies for children exposed to phenytoin (PHT) (N = 1327), based on data from 26 studies, was 5.4% (95% CI 3.6 to 8.1). The prevalence of major malformations in routine health record studies for children exposed to PHT (N = 103), based on data from one study, was 6.8% (95% CI 0.1 to 91.3).

##### 8 PHT versus controls

###### 8.1 All major malformations

####### 8.1.1 PHT versus no medication (in women without epilepsy): cohort studies

Pooled results from eight studies suggested an increased risk with PHT (RR 3.81, 95% CI 1.91 to 7.57; I^2^ = 35%), with children exposed to PHT (N = 496) experiencing more major malformations than control children (N = 1397) (Analysis 8.1). The RD also suggested a higher risk for PHT (RD 0.03, 95% CI 0.01 to 0.06; I^2^ = 44%).

Samren 1997 reported nine cases of major malformation in 141 (6%) PHT‐exposed children. Outcomes at centres with a control group in this study were limited to five cases from 33 exposed children, which gave a non‐significant difference (RR 2.2, 95% CI 0.7 to 6.7).

####### 8.1.2 PHT versus no medication (in women with epilepsy): cohort studies

Pooled results from 15 studies suggested an increased risk with PHT (RR 2.01, 95% CI 1.29 to 3.12; I^2^ = 0%), with children exposed to PHT (N = 750) experiencing more major malformations than control children (N = 1588) (Analysis 8.1). The RD also suggested a higher risk for PHT (RD 0.03, 95% CI 0.01 to 0.05; I^2^ = 0%).

####### 8.1.3 PHT versus no medication (in women without epilepsy): routine health record data studies

No included studies reported data on this outcome.

####### 8.1.4 PHT versus no medication (in women with epilepsy): routine health record data studies

No included studies reported data on this outcome.

###### 8.2 Neural tube malformations

####### 8.2.1 PHT versus no medication (in women without epilepsy): cohort studies

Pooled results from four studies suggested no evidence of a difference in risk (RR 13.17, 95% CI 0.58 to 299.00, I^2^ = NA) with no difference in the number of neural tube malformations in children exposed to PHT (N = 48) and control children (N = 590) (Analysis 8.2). The RD also suggested no difference in the level of risk (RD ‐0.00, 95% CI −0.06 to 0.06; I^2^ = 0%).

####### 8.2.2 PHT versus no medication (in women with epilepsy): cohort studies

Pooled results from six studies suggested no evidence of a difference in risk (RR 2.56, 95% CI 0.64 to 10.17; I^2^ = 28%), with no difference in the number of neural tube malformations in children exposed to PHT (N = 252) and control children (N = 595) (Analysis 8.2). The RD also suggested no difference in the level of risk (RD 0.01, 95% CI −0.01 to 0.03; I^2^ = 0%).

####### 8.2.3 PHT versus no medication (in women without epilepsy): routine health record data studies

No included studies reported data on this outcome.

####### 8.2.4 PHT versus no medication (in women with epilepsy): routine health record data studies

No included studies reported data on this outcome.

###### 8.3 Cardiac malformations

####### 8.3.1 PHT versus no medication (in women without epilepsy): cohort studies

Pooled results from four studies suggested no evidence of a difference in risk (RR 6.31, 95% CI 0.75 to 52.91, I^2^ = 0%), with no difference in the number of cardiac malformations in children exposed to PHT (N = 48) and control children (N = 590) (Analysis 8.3). The RD also suggested no difference in the level of risk (RD 0.02, 95% CI −0.05 to 0.08; I^2^ = 0%).

####### 8.3.2 PHT versus no medication (in women with epilepsy): cohort studies

Pooled results from seven studies suggested no evidence of a difference in risk (RR 1.86, 95% CI 0.72 to 4.80; I^2^ = 0%), with no difference in the number of cardiac malformations in children exposed to PHT (N = 253) and control children (N = 599) (Analysis 8.3). The RD also suggested no difference in the level of risk (RD 0.01, 95% CI −0.01 to 0.04; I^2^ = 0%).

####### 8.3.3 PHT versus no medication (in women without epilepsy): routine health record data studies

No included studies reported data on this outcome.

####### 8.3.4 PHT versus no medication (in women with epilepsy): routine health record data studies

No included studies reported data on this outcome.

###### 8.4 Oro‐facial cleft/craniofacial malformations

####### 8.4.1 PHT versus no medication (in women without epilepsy): cohort studies

Pooled results from four studies suggested no evidence of a difference in risk (RR 0.67, 95% CI 0.04 to 12.54, I^2^ = NA), with no difference in the number of oro‐facial cleft/ craniofacial malformations in children exposed to PHT (N = 48) and control children (N = 590) (Analysis 8.4). The RD also suggested no difference in the level of risk (RD ‐0.01, 95% CI −0.08 to 0.05; I^2^ = 0%).

####### 8.4.2 PHT versus no medication (in women with epilepsy): cohort studies

We were unable to estimate a RR from five studies due to no reported oro‐facial cleft/craniofacial malformations in children exposed to PHT (N = 133) and control children (N = 530) (Analysis 8.4).

####### 8.4.3 PHT versus no medication (in women without epilepsy): routine health record data studies

No included studies reported data on this outcome.

####### 8.4.4 PHT versus no medication (in women with epilepsy): routine health record data studies

No included studies reported data on this outcome.

###### 8.5 Skeletal/limb malformations

####### 8.5.1 PHT versus no medication (in women without epilepsy)

Pooled results from four studies suggested no evidence of a difference in risk (RR 1.56, 95% CI 0.07 to 37.19, I^2^ = NA), with no difference in the number of skeletal/limb malformations in children exposed to PHT (N = 48) and control children (N = 590) (Analysis 8.5). The RD also suggested no difference in the level of risk (RD ‐0.00, 95% CI −0.07 to 0.06; I^2^ = 0%).

####### 8.5.2 PHT versus no medication (in women with epilepsy)

Pooled results from six studies suggested no evidence of a difference in risk (RR 1.57, 95% CI 0.31 to 7.95; I^2^ = 0%), with no difference in the number of skeletal/limb malformations in children exposed to PHT (N = 252) and control children (N = 595) (Analysis 8.5). The RD also suggested no difference in the level of risk (RD 0.00, 95% CI −0.02 to 0.02; I^2^ = 0%).

####### 8.5.3 PHT versus no medication (in women without epilepsy): routine health record data studies

No included studies reported data on this outcome.

####### 8.5.4 PHT versus no medication (in women with epilepsy): routine health record data studies

No included studies reported data on this outcome.

##### Phenytoin dose

The majority of included studies did not investigate or formally report on the relationship between the dose of PHT and malformation outcome, with many being limited by included numbers of PHT‐exposed pregnancies. The North American Epilepsy and Pregnancy Register, based on 416 exposed children, did not find an increased MCM with higher doses of PHT. Kaaja 2003 with 124 PHT‐exposed children also reported no association with dose. However, in contrast, Kaneko 1999 reported evidence of an association between PHT dose and MCM prevalence, based on 132 children exposed to monotherapy PHT (no further details given). EURAP 2018 included 125 pregnancies with PHT exposure and reported a prevalence of 6.4% (95% CI 2.8 to 12.2). They did not investigate within‐group dose associations because of group size but they did report that, in comparison to LTG at doses =/< 325 mg/d, children exposed to PHT at doses between =/> 30 mg/d to 730 mg/d demonstrated no evidence of a difference in risk (OR 1.93, 95% CI 0.78 to 4.75); but this should be considered with caution due to the wide range of PHT doses included. Data from other included studies were limited by group size or dose associations were not reported.

#### Primidone

The prevalence of major malformations (any type) in cohort studies for children exposed to primidone (PRM) (N = 112), based on data from seven studies, was 7.9% (95% CI 2.6 to 21.5). The prevalence of major malformations in routine health record studies for children exposed to PRM (N = 3), was based on data from one study and therefore was not calculated.

##### 9 PRM versus controls

###### 9.1 All major malformations

####### 9.1.1 PRM versus no medication (in women without epilepsy): cohort studies

The results from one study (Koch 1992) suggested no evidence of a difference in risk (RR 0.48, 95% CI 0.03 to 8.43, I^2^ = NA) (Analysis 9.1) with no difference in the number of major malformations in children exposed to PRM (N = 21) and control children (N = 116). The RD also suggested no difference in the level of risk (RD ‐0.04, 95% CI −0.12 to 0.03; I^2^ = NA).

Samren 1997 reported four cases of major malformations out of 43 PRM‐exposed children (9%). When limited to centres with control children, there were three cases out of 39 exposed children, which suggested no evidence of difference from control children (RR 1.0, 95% CI 0.3 to 3.8).

####### 9.1.2 PRM versus no medication (in women with epilepsy): cohort studies

Pooled results from six studies suggested an increased risk with PRM (RR 3.61, 95% CI 1.41 to 9.23; I^2^ = 8%), with children exposed to PRM (N = 108) experiencing more major malformations than control children (N = 573) (Analysis 9.1). The RD also suggested a higher risk for PRM (RD 0.07, 95% CI 0.00 to 0.14; I^2^ = 11%).

####### 9.1.3 PRM versus no medication (in women without epilepsy): routine health record data studies

No included studies reported data on this outcome.

####### 9.1.4 PRM versus no medication (in women with epilepsy): routine health record data studies

No included studies reported data on this outcome.

Specific malformation types were not reviewed due to no reported data on these outcomes.

##### Primidone dose

No included studies investigated the dose of PRM and malformation risk.

#### Topiramate

The prevalence of major malformations (any type) in cohort studies for children exposed to topiramate (TPM) (N = 510), based on data from eight studies, was 3.9% (95% CI 2.3 to 6.5). The prevalence of major malformations in routine health record studies for children exposed to TPM (N = 49), based on data from two studies, was 4.1% (95% CI 0.0 to 27,060).

##### 10 TPM versus controls

###### 10.1 All major malformations

####### 10.1.1 TPM versus no medication (in women without epilepsy): cohort studies

Pooled data from three studies suggested an increased risk with TPM (RR 4.07, 95% CI 1.64 to 10.14; I^2^ = 0%), with children exposed to TPM (N = 367) experiencing more major malformations than control children (N = 825) (Analysis 10.1). The RD also suggested a higher risk for TPM (RD 0.03, 95% CI 0.01 to 0.06; I^2^ = 0).

There was just one case of MCM in 41 monotherapy cases described by the Israeli Teratogen Service, giving a prevalence of 4.9%, which suggested no difference in risk to control children (3.4%, P value not reported).

####### 10.1.2 TPM versus no medication (in women with epilepsy): cohort studies

Pooled results from five studies suggested no evidence of a difference in risk (RR 1.37, 95% CI 0.57 to 3.27; I^2^ = 0%), with no difference in the number of major malformations in children exposed to TPM (N = 139) and control children (N = 1080) (Analysis 10.1). The RD also suggested no difference in the level of risk (RD 0.01, 95% CI −0.03 to 0.04; I^2^ = 0%).

####### 10.1.3 TPM versus no medication (in women without epilepsy): routine health record data studies

The results from one study suggested no evidence of a difference in risk (RR 1.65, 95% CI 0.43 to 6.42; I^2^ = NA), with children exposed to TPM (N = 48) experiencing comparable rates of major malformations to control children (N = 369,267) (Analysis 10.1). The RD also suggested no difference in the level of risk (RD 0.02, 95% CI −0.04 to 0.07; I^2^ = NA).

####### 10.1.4 TPM versus no medication (in women with epilepsy): routine health record data studies

The results from one study suggested no evidence of a difference in risk (RR 1.62, 95% CI 0.40 to 6.45; I^2^ = NA), with children exposed to TPM (N = 48) experiencing comparable rates of major malformations to control children (N = 1900) (Analysis 10.1). The RD also suggested no difference in the level of risk (RD 0.02, 95% CI −0.04 to 0.07; I^2^ = NA).

###### 10.2 Neural tube malformations

####### 10.2.1 TPM versus no medication (in women without epilepsy): cohort studies

We were unable to estimate a RR from two studies due to there being no reported neural tube malformations in children exposed to TPM (N = 8) or control children (N = 383) (Analysis 10.2).

####### 10.2.2 TPM versus no medication (in women with epilepsy): cohort studies

We were unable to estimate a RR from three studies due to there being no reported neural tube malformations in children exposed to TPM (N = 59) and control children (N = 383) (Analysis 10.2).

####### 10.2.3 TPM versus no medication (in women without epilepsy): routine health record data studies

No included studies reported data on this outcome.

####### 10.2.4 TPM versus no medication (in women with epilepsy): routine health record data studies

No included studies reported data on this outcome.

###### 10.3 Cardiac malformations

####### 10.3.1 TPM versus no medication (in women without epilepsy): cohort studies

Pooled data from two included studies suggested evidence of a difference in risk (RR 20.71, 95% CI 2.64 to 162.72, I^2^ = 0%), with children exposed to TPM (N = 8) experiencing more cardiac malformations than control children (N = 383) (Analysis 10.3). However, the RD suggested no difference in the level of risk (RD 0.12, 95% CI −0.16 to 0.39; I^2^ = 0%).

####### 10.3.2 TPM versus no medication (in women with epilepsy): cohort studies

Pooled data from four included studies suggested no evidence of a difference in risk (RR 2.48, 95% CI 0.49 to 12.49; I^2^ = NA), with no difference in the number of cardiac malformations in children exposed to TPM (N = 60) and control children (N = 510) (Analysis 10.3). The RD also suggested no difference in the level of risk (RD 0.01, 95% CI −0.05 to 0.06; I^2^ = 0%).

####### 10.3.3 TPM versus no medication (in women without epilepsy): routine health record data studies

No included studies reported data on this outcome.

####### 10.3.4 TPM versus no medication (in women with epilepsy): routine health record data studies

No included studies reported data on this outcome.

###### 10.4 Oro‐facial cleft/craniofacial malformations

####### 10.4.1 TPM versus no medication (in women without epilepsy): cohort studies

We were unable to estimate a RR from two studies due to there being no reported oro‐facial cleft/ craniofacial malformations in children exposed to TPM (N = 8) or control children (N = 383) (Analysis 10.4).

####### 10.4.2 TPM versus no medication (in women with epilepsy): cohort studies

Pooled data from three included studies suggested no evidence of a difference in risk (RR 1.50, 95% CI 0.09 to 24.92; I^2^ = NA), with no difference in the number of oro‐facial cleft/craniofacial malformations in children exposed to TPM (N = 51) and control children (N = 170) (Analysis 10.4). The RD also suggested no difference in the level of risk (RD ‐0.00, 95% CI −0.05 to 0.04; I^2^ = 0%).

####### 10.4.3 TPM versus no medication (in women without epilepsy): routine health record data studies

No included studies reported data on this outcome to be included in the meta‐analysis. However, the study by Hernandez‐Diaz and colleagues using US Medicaid Registers could not be included in the meta‐analysis due to a lack of reporting of specific numbers of oral clefts. In comparison to children born to women without epilepsy (N = 1,322,955), the children exposed to TPM (N = 2425) had higher rates of oral clefts of 4.1 per 1000 live births (RR 3.63, 95% CI 1.95 to 6.76).

####### 10.4.4 TPM versus no medication (in women with epilepsy): routine health record data studies

No included studies reported data on this outcome.

###### 10.5 Skeletal/limb malformations

####### 10.5.1 TPM versus no medication (in women without epilepsy): cohort studies

We were unable to estimate a RR from two studies due to there being no reported skeletal/limb malformations in children exposed to TPM (N = 8) or control children (N = 383) (Analysis 10.5).

####### 10.5.2 TPM versus no medication (in women with epilepsy): cohort studies

Pooled data from three included studies suggested no evidence of a difference in risk (RR 2.06, 95% CI 0.24 to 17.42; I^2^ = 0%), with no difference in the number of skeletal/limb malformations in children exposed to TPM (N = 59) and control children (N = 502) (Analysis 10.5). The RD also suggested no difference in the level of risk (RD ‐0.01, 95% CI −0.05 to 0.04; I^2^ = 0%).

####### 10.5.3 TPM versus no medication (in women without epilepsy): routine health record data studies

No included studies reported data on this outcome.

####### 10.5.4 TPM versus no medication (in women with epilepsy): routine health record data studies

No included studies reported data on this outcome.

##### Topiramate dose

The largest included cohort of TPM‐exposed pregnancies came from the study by Hernandez‐ Diaz using data from the US Medicaid Registers (N = 2425). This register reported the risk of oral clefts for doses ≤ 100 mg/d as 2.4 per 1000 live births, and for doses > 100 mg/d, as 7.3 per 1000 live births. The adjusted values of corresponding adjusted RRs for daily doses ≤ 100 and > 100 mg were 1.64 (95% CI 0.53 to 5.07) and 5.16 (95% CI 1.94 to 13.73) for lower and higher doses, respectively. The data were too limited to provide dose investigations specifically for women with epilepsy, but they did report that higher doses tended to be used for women requiring TPM for the treatment of epilepsy.

North American Epilepsy and Pregnancy Register found no evidence of a difference in the median dose between TPM‐exposed children (N = 359) who had MCM versus those who did not (P value not reported). The Australian Epilepsy and Pregnancy Register (N = 53), did not find a dose association for monotherapy TPM but did see an increase in risk with polytherapy (prevalence not given). The UK and Ireland Epilepsy and Pregnancy Register cohort (N = 70) also failed to find an association between the dose of TPM and the risk of overall MCM. However, caution is required due to smaller numbers from the Epilepsy and Pregnancy Register cohorts currently for monotherapy TPM exposure in pregnancy.

#### Valproate

The prevalence of major malformations (any type) in cohort studies for children exposed to valproate (VPA) (N = 3018), based on data from 31 studies, was 9.8% (95% CI 8.1 to 11.9). The prevalence of major malformations in routine health record studies for children exposed to VPA (N = 1364), based on data from six studies, was 9.7% (95% CI 7.1 to 13.4).

##### 11 VPA versus controls

###### 11.1. All major malformations

####### 11.1.1 VPA versus no medication (in women without epilepsy): cohort studies

Pooled results from 10 studies suggested an increased risk with VPA (RR 5.53, 95% CI 3.29 to 9.29; I^2^ = 0%), with children exposed to VPA (N = 501) experiencing more major malformations than control children (N = 2634) (Analysis 11.1). The RD also suggested a higher risk for VPA (RD 0.07, 95% CI 0.04 to 0.10; I^2^ = 40%).

Data from the Israeli Teratogen Service study, including women treated with VPA for epilepsy and other indications (restricted to monotherapy), reported major congenital malformations (MCM) in 3/89 (3.4%) VPA‐treated cases compared with 31/1236 (2.5%) of control children. Samren 1997 reported 16 cases of major malformations out of 184 (9%) VPA‐exposed children. When limited to the two sites with control children, investigators reported six cases with malformation out of 21 children exposed to VPA, which was higher than control children (RR 4.9, 95% CI 1.6 to 15.0).

####### 11.1.2 VPA versus no medication (in women with epilepsy): cohort studies

Pooled results from 17 studies suggested an increased risk with VPA (RR 2.77, 95% CI 2.03 to 3.79; I^2^ = 0%), with children exposed to VPA (N = 2288) experiencing more major malformations than control children (N = 1710) (Analysis 11.1). The RD also suggested a higher risk for VPA (RD 0.06, 95% CI 0.04 to 0.07; I^2^ = 32%).

####### 11.1.3 VPA versus no medication (in women without epilepsy): routine health record data studies

Pooled results from three studies suggested an increased risk with VPA (RR 2.29, 95% CI 1.71 to 3.08; I^2^ = 0%), with children exposed to VPA (N = 621) experiencing more major malformations than control children (N = 373,028) (Analysis 11.1). The RD also suggested a higher risk for VPA (RD 0.04, 95% CI 0.02 to 0.06; I^2^ = 0%).

####### 11.1.4 VPA versus no medication (in women with epilepsy): routine health record data studies

Pooled results from four studies suggested an increased risk with VPA (RR 3.01, 95% CI 2.42 to 3.75; I^2^ = 55%), with children exposed to VPA (N = 1151) experiencing more major malformations than control children (N = 12,218) (Analysis 11.1). Due to high heterogeneity, a random‐effects RR was calculated which found a similar result (RR 2.97, 95% CI 2.08 to 4.24, I^2^ =55%). The RD also suggested a higher risk for VPA (RD 0.06, 95% CI 0.05 to 0.08; I^2^ = 81%). Due to high heterogeneity, a random‐effects RD was calculated which found a similar result (RD 0.06, 95% CI 0.02 to 0.10, I^2^ =85%).

###### 11.2 Neural tube malformations

####### 11.2.1 VPA versus no medication (in women without epilepsy): cohort studies

Pooled results from four studies suggested no evidence of a difference in risk (RR 6.05, 95% CI 0.94 to 38.81; I^2^ = 20%), with no difference in the number of neural tube malformations in children exposed to VPA (N = 104) and control children (N = 836) (Analysis 11.2). The RD also suggested no difference in the level of risk (RD 0.01, 95% CI −0.02 to 0.04; I^2^ = 0%).

####### 11.2.2 VPA versus no medication (in women with epilepsy): cohort studies

Pooled results from eight studies suggested an increased risk with VPA (RR 5.64, 95% CI 1.37 to 23.24; I^2^ = 0%), with a higher number of neural tube malformations in children exposed to VPA (N = 814) than in control children (N = 664) (Analysis 11.2). The RD also suggested a higher risk for VPA (RD 0.02, 95% CI 0.01 to 0.03; I^2^ = 6%).

####### 11.2.3 VPA versus no medication (in women without epilepsy): routine health record data studies

No included studies reported data on this outcome.

####### 11.2.4 VPA versus no medication (in women with epilepsy): routine health record data studies

Data from one study suggested an increased risk with VPA (RR 8.02, 95% CI 1.48 to 43.50, I^2^ = NA), with a higher number of neural tube malformations in children exposed to VPA (N = 225) than in control children (N = 902) (Analysis 11.2). The RD also suggested a higher risk for VPA (RD 0.02, 95% CI −0.00 to 0.03; I^2^ = NA).

###### 11.3 Cardiac malformations

####### 11.3.1 VPA versus no medication (in women without epilepsy): cohort studies

Pooled results from four studies suggested an increased risk with VPA (RR 11.89 95% CI 2.88 to 49.08; I^2^ = 0%), with children exposed to VPA (N = 104) experiencing more cardiac malformations than control children (N = 836) (Analysis 11.3). However, the RD suggested no difference in the level of risk (RD 0.04, 95% CI −0.00 to 0.09; I^2^ = 28%).

####### 11.3.2 VPA versus no medication (in women with epilepsy): cohort studies

Pooled results from 10 studies suggested an increased risk with VPA (RR 2.71, 95% CI 1.42 to 5.17; I^2^ = 0%), with a higher number of cardiac malformations in children exposed to VPA (N = 821) than in control children (N = 676) (Analysis 11.3). The RD also suggested a higher risk for VPA (RD 0.03, 95% CI 0.01 to 0.05; I^2^ = 0%).

####### 11.2.3 VPA versus no medication (in women without epilepsy): routine health record data studies

No included studies reported data on this outcome.

####### 11.2.4 VPA versus no medication (in women with epilepsy): routine health record data studies

No included studies reported data on this outcome.

###### 11.4 Oro‐facial cleft/craniofacial malformations

####### 11.4.1 VPA versus no medication (in women without epilepsy): cohort studies

Pooled results from four studies suggested no evidence of a difference in risk (RR 2.76, 95% CI 0.31 to 24.78; I^2^ = NA), with no difference in the number of oro‐facial cleft/craniofacial malformations in children exposed to VPA (N = 104) and control children (N = 836) (Analysis 11.4). The RD also suggested no difference in the level of risk (RD 0.01, 95% CI −0.02 to 0.04; I^2^ = 0%).

####### 11.4.2 VPA versus no medication (in women with epilepsy): cohort studies

Pooled results from eight studies suggested an increased risk with VPA (RR 4.44, 95% CI 1.14 to 17.27; I^2^ = 2%), with more children exposed to VPA (N = 474) experiencing oro‐facial cleft/craniofacial malformations than control children (N = 332) (Analysis 11.4). The RD also suggested a higher risk for VPA (RD 0.02, 95% CI 0.00 to 0.05; I^2^ = 0%).

####### 11.4.3 VPA versus no medication (in women without epilepsy): routine health record data studies

No included studies reported data on this outcome.

####### 11.4.4 VPA versus no medication (in women with epilepsy): routine health record data studies

No included studies reported data on this outcome.

###### 11.5 Skeletal/limb malformations

####### 11.5.1 VPA versus no medication (in women without epilepsy): cohort studies

Pooled results from four studies suggested an increased risk with VPA (RR 16.48, 95% CI 2.46 to 110.49; I^2^ = 0%), with children exposed to VPA (N = 104) experiencing more skeletal/limb malformations than control children (N = 836) (Analysis 11.5). However, the RD suggested no difference in the level of risk (RD 0.03, 95% CI −0.01 to 0.07; I^2^ = 0%).

####### 11.5.2 VPA versus no medication (in women with epilepsy): cohort studies

Pooled results from eight studies suggested no evidence of a difference in risk (RR 2.38, 95% CI 0.93 to 6.12; I^2^ = 0%), with no difference in the number of skeletal/limb malformations in children exposed to VPA (N = 814) and control children (N = 664) (Analysis 11.5). The RD also suggested no difference in the level of risk (RD 0.01, 95% CI −0.00 to 0.03; I^2^ = 0%).

####### 11.5.3 VPA versus no medication (in women without epilepsy): routine health record data studies

No included studies reported data on this outcome.

####### 11.5.4 VPA versus no medication (in women with epilepsy): routine health record data studies

No included studies reported data on this outcome.

##### Valproate dose

In contrast to the results on dosage for the other AEDs, for VPA there appears to be a consistently documented and clear association between increased dose and the risk for MCM in VPA‐exposed children. EURAP 2018 reported evidence that suggested a dose‐related MCM risk for VPA exposure. In 1381 exposed pregnancies, the MCM risk ranged from 6.3% (95% CI 4.5 to 8.6%) for doses =/< 650 mg/d, to 11.3% for doses > 650 mg/d to =/< 1450 mg/day and, most concerning, 25.2% (95% CI 17.6 to 34.2) for doses > 1450 mg/d. Doses of VPA =/< 650 mg/d (OR 2.70, 95% CI 1.67 to 4.38, P = 0.0002), doses > 650 mg/d to =/< 1450 mg/day (OR 4.72, 95% CI 3.11 to 7.18, P = 0.0002) and doses > 1450 mg/d (OR 13.52, 95% CI 7.73 to 23.64, P = 0.0002) were all associated with higher risk than LTG exposure at doses < 325 mg/d. Similarly, doses of > 650 mg/d to =/< 1450 mg/day (OR 2.81, 95% CI 1.70 to 4.65, P = 0.0002) had higher risk than LTG > 325 mg/d. The highest level of VPA exposure was not statistically compared to LTG doses > 325 mg/d, but there was a large difference in prevalence (25.2% vs 4.3%). The lowest doses of VPA investigated (=/< 650 mg/d) were not associated with a lower MCM risk than higher doses (> 325 mg/d) of LTG (OR 0.62, 95% CI 0.36 to 1.09, P = 0.0959).

In the UK and Ireland Epilepsy and Pregnancy Register (N = 1220), an increase in malformation from 5.0% at doses < 600 mg/d to 10.4% for doses > 1000 mg/d (OR 2.20 95% CI 1.26 to 3.82, P = 0.0045) was reported. The Australian Epilepsy and Pregnancy Register cohort also demonstrated an association with VPA (N = 290), as did the North American Epilepsy and Pregnancy Register (N = 323), where investigators reported the median daily dose in VPA‐exposed children with a malformation to be 1000 mg/d compared with children exposed to VPA without an MCM (750 mg/d). The Kerala Epilepsy and Pregnancy Registry reported a prevalence of MCM of 3.0% for doses of VPA =/< 400 mg/d, 9.5% for doses between > 400 to 800 mg/d, and 28.6% for doses over 800 mg/d. Smaller studies including VPA‐exposed children also reported data showing an association between VPA dose or serum levels and increased MCM rate (Israeli Teratogen Service; Kaneko 1999; Lindhout 1992; Meador 2006; Milan Study 1999; Samren 1997). Kaaja 2003 was the only smaller study that investigated a dose‐response association without finding a positive correlation (N = 61 VPA‐exposed pregnancies).

Investigations from studies using population health record data are fewer, due to the lack of dose information available for the Norwegian Health Record Registers, Sweden Health Record Registers, and the absence of dose information for the Denmark Health Record Registers or UK Clinical Research Practice Database; UK Health Record THIN Register at this time. Putignano and colleagues 2019, using the Italian Lombardy Region Health Register, reported that children with MCMs had a higher dose of VPA.

#### Zonisamide

The prevalence of major malformations (any type) in cohort studies for children exposed to zonisamide (ZNS) (N = 130), based on data from four studies, was 2.7% (95% CI 0.1 to 47.3). There were no children exposed to ZNS in routine health record studies, therefore, the prevalence of major malformations rated could not be calculated.

##### 12 ZNS versus controls

###### 12.1. All major malformations

####### 12.1.1 ZNS versus no medication (in women without epilepsy): cohort studies

Pooled data from two studies suggested no evidence of a difference in risk (RR 1.13, 95% CI 0.21 to 6.11; I^2^ = 36%), with no difference in the number of major malformations in children exposed to ZNS (N = 103) and control children (N = 548) (Analysis 12.1). The RD also suggested no difference in the level of risk (RD −0.00, 95% CI −0.03 to 0.02; I^2^ = 39%).

####### 12.1.2 ZNS versus no medication (in women with epilepsy): cohort studies

Pooled data from two studies suggested an increased risk with ZNS (RR 3.20, 95% CI 1.09 to 9.43; I^2^ = 0%), with a higher number of major malformations in children exposed to ZNS (N = 39) than in control children (N = 556) (Analysis 12.1). However, the RD suggested no difference in the level of risk (RD 0.07, 95% CI −0.03 to 0.18; I^2^ = 0%).

####### 12.1.3 ZNS versus no medication (in women without epilepsy): routine health record data studies

No included studies reported data on this outcome.

####### 12.1.4 ZNS versus no medication (in women with epilepsy): routine health record data studies

No included studies reported data on this outcome.

###### 12.2. Neural tube malformations

####### 12.2.1 ZNS versus no medication (in women without epilepsy): cohort studies

We were unable to estimate a RR from one included study due to there being no reported neural tube malformations in children exposed to ZNS (N = 13) or control children (N = 106) (Analysis 12.2).

####### 12.2.2 ZNS versus no medication (in women with epilepsy): cohort studies

We were unable to estimate a RR from one included study due to there being no reported neural tube malformations in children exposed to ZNS (N = 13) or control children (N = 15) (Analysis 12.2).

####### 12.2.3 ZNS versus no medication (in women without epilepsy): routine health record data studies

No included studies reported data on this outcome.

####### 12.2.4 ZNS versus no medication (in women with epilepsy): routine health record data studies

No included studies reported data on this outcome.

###### 12.3 Cardiac malformations

####### 12.3.1 ZNS versus no medication (in women without epilepsy): cohort studies

We were unable to estimate a RR from one included study due to there being no reported cardiac malformations in children exposed to ZNS (N = 13) or control children (N = 106) (Analysis 12.3).

####### 12.3.2 ZNS versus no medication (in women with epilepsy): cohort studies

We were unable to estimate a RR from one included study due to there being no reported cardiac malformations in children exposed to ZNS (N = 13) or control children (N = 15) (Analysis 12.3).

####### 12.3.3 ZNS versus no medication (in women without epilepsy): routine health record data studies

No included studies reported data on this outcome.

####### 12.3.4 ZNS versus no medication (in women with epilepsy): routine health record data studies

No included studies reported data on this outcome.

###### 12.4 Oro‐facial cleft/craniofacial malformations

####### 12.4.1 ZNS versus no medication (in women without epilepsy): cohort studies

We were unable to estimate a RR from one included study due to there being no reported oro‐facial cleft/craniofacial malformations in children exposed to ZNS (N = 13) or control children (N = 106) (Analysis 12.4).

####### 12.4.2 ZNS versus no medication (in women with epilepsy): cohort studies

We were unable to estimate a RR from one included study due to there being no reported oro‐facial cleft/craniofacial malformations in children exposed to ZNS (N = 13) or control children (N = 15) (Analysis 12.4).

####### 12.4.3 ZNS versus no medication (in women without epilepsy): routine health record data studies

No included studies reported data on this outcome.

####### 12.4.4 ZNS versus no medication (in women with epilepsy): routine health record data studies

No included studies reported data on this outcome.

###### 12.5 Skeletal/limb malformations

####### 12.5.1 ZNS versus no medication (in women without epilepsy)

We were unable to estimate a RR from one included study due to there being no skeletal/limb malformations in children exposed to ZNS (N = 13) or control children (N = 106) (Analysis 12.5).

####### 12.5.2 ZNS versus no medication (in women with epilepsy)

We were unable to estimate a RR from one included study due to there being no reported skeletal/limb malformations in children exposed to ZNS (N = 13) or control children (N = 15) (Analysis 12.5).

####### 12.5.3 ZNS versus no medication (in women without epilepsy): routine health record data studies

No included studies reported data on this outcome.

####### 12.5.4 ZNS versus no medication (in women with epilepsy): routine health record data studies

No included studies reported data on this outcome.

##### Zonisamide dose

No included study investigated a potential association between ZNS and malformation risk.

#### ASM versus ASM comparisons

##### 13 CBZ versus CZP

###### 13.1. All major malformations

####### 13.1.1 Cohort studies

Pooled results from four studies suggested no evidence of a difference in risk (RR 1.82, 95% CI 0.63 to 5.26; I^2^ = 0%), with no difference in the number of major malformations in children exposed to CBZ (N = 1311) and children exposed to CZP (N = 95) (Analysis 13.1). However, the RD suggested a higher risk for CBZ (RD 0.04, 95% CI ‐0.00 to 0.08; I^2^ = 0%).

####### 13.1.2 Routine health record data studies

Pooled data from two studies suggested no evidence of a difference in risk (RR 1.29, 95% CI 0.47 to 3.51; I^2^ = 0%), with no difference in the number of major malformations in children exposed to CBZ (N = 1388) and children exposed to CZP (N = 161). The RD also suggested no difference in the level of risk (RD 0.01, 95% CI ‐0.00 to 0.04; I^2^ = 0%).

###### 13.2 Neural tube malformations

####### 13.2.1 Cohort studies

No included studies reported data on this outcome.

####### 13.2.2 Routine health record data studies

No included studies reported data on this outcome.

###### 13.3 Cardiac malformations

####### 13.3.1 Cohort studies

No included studies reported data on this outcome.

####### 13.3.2 Routine health record data studies

No included studies reported data on this outcome.

###### 13.4 Oro‐facial cleft/craniofacial malformations

####### 13.4.1 Cohort studies

No included studies reported data on this outcome.

####### 13.4.2 Routine health record data studies

No included studies reported data on this outcome.

###### 13.5 Skeletal/limb malformations

####### 13.5.1 Cohort studies

No included studies reported data on this outcome.

####### 13.5.2 Routine health record data studies

No included studies reported data on this outcome.

##### 14 CBZ versus GBP

###### 14.1. All major malformations

####### 14.1.1 Cohort studies

Pooled results from four studies suggested no evidence of a difference in risk(RR 1.55, 95% CI 0.57 to 4.26; I^2^ = 47%), with no difference in the number of major malformations in children exposed to CBZ (N = 3112) and children exposed to GBP (N = 192) (Analysis 14.1). The RD also suggested no difference in the level of risk (RD 0.02, 95% CI ‐0.00 to 0.04; I^2^ = 42%).

####### 14.1.2 Routine health record data studies

Data from one study suggested no evidence of a difference in risk (RR 1.54, 95% CI 0.10 to 24.27; I^2^ = NA), with no difference in the number of major malformations in children exposed to CBZ (N = 703) and children exposed to GBP (N = 18) (Analysis 14.1). The RD also suggested no difference in the level of risk (RD 0.04, 95% CI ‐0.03 to 0.11; I^2^ = NA).

###### 14.2 Neural tube malformations

####### 14.2.1 Cohort studies

Data from one included study suggested no evidence of a difference in risk (RR 0.12, 95% CI 0.01 to 2.93, I^2^ = NA) with no difference in the number of neural tube malformations in children exposed to CBZ (N= 361) and GBP‐exposed children (N = 14) (Analysis 14.2). The RD also suggested no difference in the level of risk (RD 0.00, 95% CI −0.09 to 0.09; I^2^ = NA).

####### 14.2.2 Routine health record data studies

No included studies reported data on this outcome.

###### 14.3 Cardiac malformations

####### 14.3.1 Cohort studies

Pooled results from two studies suggest n increased in risk (RR 0.13, 95% CI 0.02 to 0.95, I^2^ = 0%) with children exposed to GBP (N = 16) being at a higher risk of cardiac malformation than children exposed to CBZ (N = 374)(Analysis 14.3). However, the RD also suggested no difference in the level of risk (RD ‐0.05, 95% CI −0.18 to 0.08; I^2^ = 74%).

####### 14.3.2 Routine health record data studies

No included studies reported data on this outcome.

###### 14.4 Oro‐facial cleft/craniofacial malformations

####### 14.4.1 Cohort studies

Results from one included study suggested no evidence of a difference in risk (RR 0.37, 95% CI 0.02 to 6.62, I^2^ = NA), with no difference in the number of oro‐facial cleft/craniofacial malformations in children exposed to CBZ (N = 361) and children exposed to GBP (N = 14) (Analysis 14.4). The RD also suggested no difference in the level of risk (RD 0.01, 95% CI −0.08 to 0.10; I^2^ = NA).

####### 14.4.2 Routine health record data studies

No included studies reported data on this outcome.

###### 14.5 Skeletal/limb malformations

####### 14.5.1 Cohort studies

Results from one included study suggest no evidence of a difference in risk (RR 0.21, 95% CI 0.01 to 4.13, I^2^ = NA), with no difference in the number of skeletal/limb malformations in children exposed to CBZ (N = 361) and children exposed to GBP (N = 14) (Analysis 14.5). The RD also suggested no difference in the level of risk (RD 0.01, 95% CI −0.09 to 0.10; I^2^ = NA).

####### 14.5.2 Routine health record data studies

No included studies reported data on this outcome.

##### 15 CBZ versus LEV

###### 15.1. All major malformations

####### 15.1.1 Cohort studies

Pooled results from 11 studies suggested an increased risk with CBZ(RR 1.51, 95% CI 1.01 to 2.26; I^2^ = 0%), with more children exposed to CBZ (N = 3814) experiencing major malformations than children exposed to LEV (N = 1242) (Analysis 15.1). The RD also suggested a higher risk for CBZ (RD 0.01, 95% CI 0.00 to 0.02; I^2^ = 0%).

The EURAP 2018 collaboration reported the prevalence of MCM was 5.5% (95% CI 4.5 to 6.6%) for children exposed to CBZ and 2.8% (95% CI 1.7 to 4.5) for children exposed to LEV. No direct statistical comparison was made at the group level, investigations were made across different doses of the two ASMs (see Carbamazepine dose and Levetiracetam dose sections).

####### 15.1.2 Routine health record data studies

Pooled results from two studies suggested no evidence of a difference in risk (RR 1.73, 95% CI 0.78 to 3.83; I^2^ = 0%), with no difference in the number of major malformations in children exposed to CBZ (N = 1000) and children exposed to LEV (N = 248) (Analysis 15.1). The RD also suggested no difference in the level of risk (RD 0.02, 95% CI ‐0.00 to 0.04; I^2^ = 0%).

###### 15.2 Neural tube malformations

####### 15.2.1 Cohort studies

Pooled results from 10 studies suggested no evidence of a difference in risk (RR 1.57, 95% CI 0.41 to 6.08; I^2^ = 0%), with no difference in the number of neural tube malformations in children exposed to CBZ (N = 3731) and children exposed to LEV (N = 1148) (Analysis 15.2). The RD also suggested no difference in the level of risk (RD 0.00, 95% CI ‐0.00 to 0.01; I^2^ = 0%).

EURAP 2018 reported a prevalence of 0.35% (7/1957) for cases of neural tube anomaly in children exposed to CBZ and 0% (0/599) in children exposed to LEV.

####### 15.2.2 Routine health record data studies

No included studies reported data on this outcome.

###### 15.3 Cardiac malformations

####### 15.3.1 Cohort studies

Pooled results from 11 studies suggested no evidence of a difference in risk (RR 1.20, 95% CI 0.57 to 2.52; I^2^ = 0%), with no difference in the number of cardiac malformations in children exposed to CBZ (N = 3736) and children exposed to LEV (N = 1156) (Analysis 15.3). The RD also suggested no difference in the level of risk (RD 0.00, 95% CI ‐0.01 to 0.01; I^2^ = 0%).

EURAP 2018 reported a prevalence of 1.4% (28/1957) for cases of a cardiac anomaly in children exposed to CBZ and 0.8% (5/599) in children exposed to LEV.

####### 15.3.2 Routine health record data studies

No included studies reported data on this outcome.

###### 15.4 Oro‐facial cleft/craniofacial malformations

####### 15.4.1 Cohort studies

Pooled results from 10 studies suggested no evidence of a difference in risk (RR 1.79, 95% CI 0.43 to 7.41; I^2^ = 0%), with no difference in the number of oro‐facial cleft/craniofacial malformations in children exposed to CBZ (N = 3246) and children exposed to LEV (N = 1050) (Analysis 15.4). The RD also suggested no difference in the level of risk (RD 0.00, 95% CI ‐0.00 to 0.01; I^2^ = 0%).

EURAP 2018 reported two cases of cleft lip or palate anomaly out of 1957 children exposed to CBZ and one case out of 599 children exposed to LEV.

####### 15.4.2 Routine health record data studies

No included studies reported data on this outcome.

###### 15.5 Skeletal/limb malformations

####### 15.5.1 Cohort studies

Pooled results from 10 studies suggested no evidence of a difference in risk (RR 0.99, 95% CI 0.37 to 2.68; I^2^ = 0%), with no difference in the number of skeletal/limb malformations in children exposed to CBZ (N = 3731) and children exposed to LEV (N = 1147) (Analysis 15.5). The RD also suggested no difference in the level of risk (RD 0.00, 95% CI ‐0.00 to 0.01; I^2^ = 0%).

####### 15.5.2 Routine health record data studies

No included studies reported data on this outcome.

##### 16 CBZ versus LTG

###### 16.1. All major malformations

####### 16.1.1: Cohort studies

Pooled results from 13 cohort studies suggested an increased risk with CBZ (RR 1.37, 95% CI 1.06 to 1.77; I^2^ = 0%), with children exposed to CBZ (N = 4018) experiencing more major malformations than children exposed to LTG (N = 4550) (Analysis 16.1). The RD also suggested a higher risk for CBZ (RD 0.01, 95% CI 0.00 to 0.02; I^2^ = 0%).

The EURAP 2018 collaboration reported the prevalence of MCM was 5.5% (95% CI 4.5 to 6.6%) for children exposed to CBZ and 2.9% (95% CI 2.3 to 3.7) for children exposed to LTG. No direct statistical comparison was made at the group level; investigations were made across different doses of the two ASMs (see Carbamazepine dose and Lamotrigine dose sections).

####### 16.1.2: Routine health record studies

Pooled results from four routine health record studies suggested no evidence of a difference in risk (RR 1.21, 95% CI 0.88 to 1.67; I^2^ = 21%), with no difference in the number of major malformations in children exposed to CBZ (N = 2001) and LTG (N = 2502) (Analysis 16.1). The RD also suggested no difference in the level of risk (RD 0.01, 95% CI ‐0.01 to 0.02; I^2^ = 25%).

###### 16.2. Neural tube malformations

####### 16.2.1: Cohort studies

Pooled results from 12 cohort studies suggested no evidence of a difference in risk (RR 2.19, 95% CI 0.76 to 6.33; I^2^ = 0%), with no difference in the number of neural tube malformations in children exposed to CBZ (N = 3935) and children exposed to LTG (N = 4406) (Analysis 16.2). The RD also suggested no difference in the level of risk (RD 0.00, 95% CI ‐0.00 to 0.00; I^2^ = 0%).

In the EURAP 2018 data, the prevalence of neural tube malformations in those exposed to CBZ was 0.36% (7/1957) and 0.04% for those exposed to LTG (1/2514).

####### 16.2.2: Routine health record studies

No included studies reported data on this outcome.

###### 16.3. Cardiac malformations

####### 16.3.1: Cohort studies

Pooled results from 12 cohort studies suggested no evidence of a difference in risk (RR 1.48, 95% CI 0.87 to 2.51; I^2^ = 0%), with no difference in the number of cardiac malformations in children exposed to CBZ (N = 3933) and children exposed to LTG (N = 4407) (Analysis 16.3). The RD also suggested no difference in the level of risk (RD 0.00, 95% CI ‐0.00 to 0.01; I^2^ = 0%).

In the EURAP 2018 data, the prevalence of cardiac malformations in those exposed to CBZ was 1.43% (28/1957) and 0.59% (15/2514) for those exposed to lamotrigine.

####### 16.3.2: Routine health record studies

No included studies reported data on this outcome.

###### 16.4. Oro‐facial cleft/craniofacial malformations

####### 16.4.1: Cohort studies

Pooled results from 11 studies suggested no evidence of a difference in risk (RR 1.22, 95% CI 0.57 to 2.61; I^2^ = 0%), with no difference in the number of oro‐facial cleft/craniofacial malformations in children exposed to CBZ (N = 3443) and children exposed to LTG (N = 4357) (Analysis 16.4). However, only three studies contained occurrences of oro‐facial cleft/craniofacial malformations. The RD also suggested no difference in the level of risk (RD 0.00, 95% CI ‐0.00 to 0.00; I^2^ = 0%).

In the EURAP 2018 data, the prevalence of cleft malformations (other oro‐facial not specifically reported) in those exposed to CBZ was 0.10% (2/1957) and 0.11% for children exposed to LTG (3/2514).

####### 16.4.2: Routine health record studies

No included studies reported data on this outcome.

###### 16.5. Skeletal/limb malformations

####### 16.5.1: Cohort studies

Pooled results from 12 cohort studies suggested no evidence of a difference in risk (RR 1.86, 95% CI 0.82 to 4.22; I^2^ = 0%), with no difference in the number of skeletal/limb malformations in children exposed to CBZ (N = 3935) and children exposed to LTG (N = 4406) (Analysis 16.5). The RD also suggested no difference in the level of risk (RD 0.00, 95% CI ‐0.00 to 0.00; I^2^ = 0%).

####### 16.5.2: Routine health record studies

No included studies reported data on this outcome.

##### 17 CBZ versus OXC

###### 17.1. All major malformations

####### 17.1.1 Cohort studies

Pooled results from 11 cohort studies suggested no evidence of a difference in risk (RR 1.26, 95% CI 0.74 to 2.15; I^2^ = 20%), with no difference in the number of major malformations in children exposed to CBZ (N = 2499) and children exposed to OXC (N = 378) (Analysis 17.1). The RD also suggested no difference in the level of risk (RD 0.01, 95% CI −0.01 to 0.03; I^2^ = 0%).

The EURAP 2018 collaboration reported the prevalence of MCM was 5.5% (95% CI 4.5 to 6.6%) for children exposed to CBZ and 3.0% (95% CI 1.4 to 5.4) for children exposed to OXC. No direct statistical comparison was made at the group level; investigations were made across different doses of the two ASMs (see Carbamazepine dose and Oxcarbazepine dose sections).

####### 17.1.2 Routine health record data studies

Pooled results from four routine data suggested an increased risk with CBZ (RR 0.64, 95% CI 0.44 to 0.91; I^2^ = 89%), with children exposed to CBZ (N = 2508) experiencing more malformations than the children exposed to OXC (N = 507) (Analysis 17.1). Due to heterogeneity, a random‐effects RR was calculated which found no difference in risk (RR 0.75, 95% CI 0.15 to 3.72; I^2^ = 89%). The RD also suggested no difference in the level of risk (RD ‐0.03, 95% CI ‐0.06 to 0.00, I^2^ = 89%). Due to heterogeneity, a random‐effects RD was calculated which upheld similar findings (RD ‐0.02, 95% CI ‐0.11 to 0.07; I^2^ = 89%).

###### 17.2 Neural tube malformations

####### 17.2.1 Cohort studies

Pooled results from nine cohort studies suggested no evidence of a difference in risk (RR 0.93, 95% CI 0.22 to 3.96; I^2^ = 0%), with no difference in the number of neural tube malformations in children exposed to CBZ (N = 2403) and children exposed to OXC (N = 364) (Analysis 17.2). The RD also suggested no difference in the level of risk (RD 0.01, 95% CI ‐0.01 to 0.02; I^2^ = 0%).

In the EURAP 2018 data, the prevalence of neural tube anomalies in those exposed to CBZ was 0.36% (7/1957) and 0% for children exposed to OXC (0/333).

####### 17.2.2 Routine health record data studies

Included studies did not report any data on this outcome.

###### 17.3 Cardiac malformations

####### 17.3.1 Cohort studies

Pooled results from 11 cohort studies suggested no evidence of a difference in risk (RR 0.56, 95% CI 0.23 to 1.38; I^2^ = 0%), with no difference in the number of cardiac malformations in children exposed to CBZ (N = 2421) and children exposed to OXC (N = 368) (Analysis 17.3). The RD also suggested no difference in the level of risk (RD ‐0.00, 95% CI ‐0.02 to 0.02; I^2^ = 0%).

In the EURAP 2018 data, the prevalence of cardiac anomalies in those exposed to CBZ was 1.46% (28/1957) and 1.2% for children exposed to OXC (4/333).

####### 17.3.2 Routine health record data studies

Included studies did not report any data on this outcome.

###### 17.4 Oro‐facial cleft/craniofacial malformations

####### 17.4.1 Cohort studies

Pooled results from nine cohort studies suggested no evidence of a difference in risk (RR 0.52, 95% CI 0.12 to 2.26; I^2^ = 0%), with no difference in the number of oro‐facial cleft/craniofacial malformations in children exposed to CBZ (N = 1918) and children exposed to OXC (N = 296) (Analysis 17.4). The RD also suggested no difference in the level of risk (RD 0.00, 95% CI ‐0.02 to 0.02; I^2^ = 0%).

In the EURAP 2018 data, the prevalence of cleft malformations (other oro‐facial not specifically reported) in those exposed to CBZ was 0.10% (2/1957) and 0.30% for children exposed to OXC (1/333).

####### 17.4.2 Routine health record data studies

Included studies did not report any data on this outcome.

###### 17.5 Skeletal/limb malformations

####### 17.5.1 Cohort studies

Pooled results from nine studies suggested no evidence of a difference in risk (RR 0.53, 95% CI 0.17 to 1.66; I^2^ = 0%), with no difference in the number of skeletal/limb malformations in children exposed to CBZ (N = 2403) and children exposed to OXC (N = 364) (Analysis 17.5). The RD also suggested no difference in the level of risk (RD ‐0.00, 95% CI ‐0.01 to 0.01; I^2^ = 0%).

####### 17.5.2 Routine health record data studies

Included studies did not report any data on this outcome.

##### 18 CBZ versus PB

###### 18.1 All major malformations

####### 18.1.1. Cohort studies

Pooled results from 24 cohort studies suggested no evidence of a difference in risk (RR 0.83, 95% CI 0.61 to 1.13; I^2^ = 3%), with no difference in the number of major malformations in children exposed to CBZ (N = 3235) and children exposed to PB (N = 832) (Analysis 18.1). The RD also suggested no difference in the level of risk (RD −0.01, 95% CI −0.03 to 0.01; I^2^ = 0%).

The EURAP 2018 collaboration reported the prevalence of MCM was 5.5% (95% CI 4.5 to 6.6%) for children exposed to CBZ and 6.5% (95% CI 4.2 to 9.9) for children exposed to PB. No direct statistical comparison was made at the group level; investigations were made across different doses of the two ASMs (see Carbamazepine dose and Phenobarbital dose sections). Samren 1997 reported 22 major malformation cases in 280 (8%) CBZ‐exposed children and five cases in 48 (10%) PB‐exposed children.

####### 18.1.2 Routine health record data studies

Pooled results from two routine health record studies suggested no evidence of a difference in risk (RR 0.35, 95% CI 0.12 to 1.09; I^2^ = 0%), with no difference in the number of major malformations in children exposed to CBZ (N = 1388) and children exposed to PB (N = 34) (Analysis 18.1). The RD also suggested no difference in the level of risk (RD −0.06, 95% CI −0.15 to 0.04; I^2^ = 0%).

###### 18.2 Neural tube malformations

####### 18.2.1 Cohort studies

Pooled results from 15 cohort studies suggested no evidence of a difference in risk (RR 1.28, 95% CI 0.35 to 4.75; I^2^ = 32%), with no difference in the number of neural tube malformations in children exposed to CBZ (N = 2340) and children exposed to PB (N = 550) (Analysis 18.2). The RD also suggested no difference in the level of risk (RD ‐0.00, 95% CI ‐0.01 to 0.01; I^2^ = 0%).

In the EURAP 2018 data, the prevalence of neural tube anomalies in those exposed to CBZ was 0.36% (7/1957) and 0.68% for children exposed to PB (2/294).

####### 18.2.2 Routine health record data studies

Included studies did not report any data on this outcome.

###### 18.3 Cardiac malformations

####### 18.3.1 Cohort studies

Pooled results from 15 cohort studies suggested an increased risk with PB (RR 0.26, 95% CI 0.14 to 0.47; I^2^ = 0%), with children exposed to CBZ (N = 2340) experiencing fewer cardiac malformations than children exposed to PB (N = 550) (Analysis 18.5). The RD also suggested a higher risk for PB (RD ‐0.03, 95% CI ‐0.05 to ‐0.01; I^2^ = 0%).

In the EURAP 2018 data, the prevalence of cardiac anomalies in those exposed to CBZ was 1.46% (28/1957) and 2.7% for children exposed to PB (8/333).

####### 18.3.2 Routine health record data studies

Included studies did not report any data on this outcome.

###### 18.4 Oro‐facial cleft/craniofacial malformations

####### 18.4.1 Cohort studies

Pooled results from 15 cohort studies suggested an increased risk with PB (RR 0.18, 95% CI 0.07 to 0.48; I^2^ = 0%), with children exposed to CBZ (N = 1857) experiencing fewer oro‐facial cleft/craniofacial malformations than children exposed to PB (N = 422) (Analysis 18.4). The RD suggested no difference in the level of risk for PB (RD ‐0.01, 95% CI ‐0.03 to 0.00; I^2^ = 0%).

In the EURAP 2018 data, the prevalence of cleft malformations (other oro‐facial not specifically reported) in those exposed to CBZ was 0.10% (2/1957) and 0.34% for children exposed to PB (1/294).

####### 18.4.2 Routine health record data studies

Included studies did not report any data on this outcome.

###### 18.5 Skeletal/limb malformations

####### 18.5.1 Cohort studies

Pooled results from 15 cohort studies suggested no evidence of a difference in risk (RR 1.08, 95% CI 0.45 to 2.61; I^2^ = 6%), with no difference in the number of skeletal/limb malformations in children exposed to CBZ (N = 2340) and children exposed to PB (N = 550) (Analysis 18.5). The RD also suggested no difference in the level of risk (RD 0.00, 95% CI ‐0.01 to 0.02; I^2^ = 0%).

####### 18.5.2 Routine health record data studies

Included studies did not report any data on this outcome.

##### 19 CBZ versus PHT

###### 19.1 All major malformations

####### 19.1.1 Cohort studies

Pooled results from 23 cohort studies suggested no evidence of a difference in risk (RR 0.83, 95% CI 0.62 to 1.11; I^2^ = 0%), with no difference in the number of major malformations in children exposed to CBZ (N = 4759) and children exposed to PHT (N = 1287) (Analysis 19.1). The RD also suggested no difference in the level of risk (RD −0.01, 95% CI −0.02 to 0.01; I^2^ = 0%).

The EURAP 2018 collaboration reported the prevalence of MCM was 5.5% (95% CI 4.5 to 6.6%) for children exposed to CBZ and 6.4% (95% CI 2.8 to 12.2) for children exposed to PHT. No direct statistical comparison was made at the group level; investigations were made across different doses of the two ASMs (see Carbamazepine dose and Phenytoin dose sections). Samren 1997 reported 22 cases of major malformation out of 280 (8%) CBZ‐exposed children and 9 cases from 141 PHT‐exposed children (9%).

####### 19.1.2 Routine health record data studies

Results from one routine health record data study suggested no evidence of a difference in risk (RR 0.59, 95% CI 0.26 to 1.31; I^2^ = NA), with no difference in the number of major malformations in children exposed to CBZ (N = 703) and children exposed to PHT (N = 103) (Analysis 19.1). The RD also suggested no difference in the level of risk (RD −0.03, 95% CI −0.08 to 0.02; I^2^ = 0%).

###### 19.2 Neural tube malformations

####### 19.2.1 Cohort studies

Pooled results from 16 cohort studies suggested no evidence of a difference in risk (RR 1.12, 95% CI 0.45 to 2.83; I^2^ = 0%), with no difference in the number of neural tube malformations in children exposed to CBZ (N = 4341) and children exposed to PHT (N = 1005) (Analysis 19.2). The RD also suggested no difference in the level of risk (RD 0.00, 95% CI ‐0.00 to 0.01; I^2^ = 0%).

In the EURAP 2018 data, the prevalence of neural tube anomalies in those exposed to CBZ was 0.36% (7/1957) and 0.80% for children exposed to PB (1/125).

####### 19.2.2 Routine health record data studies

Included studies did not report any data on this outcome.

###### 19.3 Cardiac malformations

####### 19.3.1 Cohort studies

Pooled results from 16 cohort studies suggested an increased risk with PHT (RR 0.44, 95% CI 0.23 to 0.84; I^2^ = 8%), with fewer cardiac malformations in children exposed to CBZ (N = 4341) than in children exposed to PHT (N = 1005) (Analysis 19.3). However, the RD suggested no difference in the level of risk (RD ‐0.01, 95% CI ‐0.02 to 0.00; I^2^ = 0%).

In the EURAP 2018 data, the prevalence of cardiac anomalies in those exposed to CBZ was 1.46% (28/1957) and 4% for children exposed to PHT (5/125).

####### 19.3.2 Routine health record data studies

Included studies did not report any data on this outcome.

###### 19.4 Oro‐facial cleft/craniofacial malformations

####### 19.4.3 Cohort studies

Pooled results from 16 cohort studies suggested no evidence of a difference in risk (RR 0.81, 95% CI 0.32 to 2.08; I^2^ = 0%), with no difference in the number of oro‐facial cleft/craniofacial malformations in children exposed to CBZ (N = 3858) and children exposed to PHT (N = 891) (Analysis 19.4). The RD also suggested no difference in the level of risk (RD ‐0.00, 95% CI ‐0.01 to 0.01; I^2^ = 0%).

In the EURAP 2018 data, the prevalence of cleft malformations (other oro‐facial not specifically reported) in those exposed to CBZ was 0.10% (2/1957) and 0% for children exposed to PHT (0/125).

####### 19.4.2 Routine health record data studies

Included studies did not report any data on this outcome.

###### 19.5 Skeletal/limb malformations

####### 19.5.1 Cohort studies

Pooled results from 16 cohort studies suggested no evidence of a difference in risk (RR 0.88, 95% CI 0.43 to 1.82; I^2^ = 0%), with no difference in the number of skeletal/limb malformations in children exposed to CBZ (N = 4341) and children exposed to PHT (N = 1005) (Analysis 19.5). The RD also suggested no difference in the level of risk (RD 0.00, 95% CI ‐0.01 to 0.01; I^2^ = 0%).

####### 19.5.2 Routine health record data studies

Included studies did not report any data on this outcome.

##### 20 CBZ versus PRM

###### 20.1 All major malformations

####### 20.1.1 Cohort studies

Pooled results from seven cohort studies suggested no evidence of a difference in risk (RR 0.59, 95% CI 0.23 to 1.56; I^2^ = 40%), with no difference in the number of major malformations in children exposed to CBZ (N = 1076) and children with PRM (N = 112) (Analysis 20.1). The RD also suggested no difference in the level of risk (RD −0.02, 95% CI −0.09 to 0.05; I^2^ = 8%).

Samren 1997 reported 22 cases of major malformation out of 280 (8%) CBZ‐exposed children and 4 cases out of 43 (9%) PRM‐exposed children.

####### 20.1.2 Routine health record data studies

Data from one included study suggested no evidence of a difference in risk (RR 0.32, 95% CI 0.02 to 4.44, I^2^ = NA), with no difference in the number of major malformations in children exposed to CBZ (N = 703) and children exposed to PRM (N = 3) (Analysis 20.1). The RD also suggested no difference in the level of risk (RD 0.04, 95% CI −0.28 to 0.36; I^2^ = NA).

###### 20.2 Neural tube malformations

####### 20.2.1 Cohort studies

Pooled data from two studies suggested no evidence of a difference in risk (RR 0.95, 95% CI 0.04 to 22.75, I^2^ = NA), with no difference in the number of neural tube malformations in children exposed to CBZ (N = 119) and children exposed to PRM (N = 39) (Analysis 20.2). The RD also suggested no difference in the level of risk (RD 0.01, 95% CI −0.04 to 0.06; I^2^ = 0%.

####### 20.2.2 Routine health record data studies

Included studies did not report any data on this outcome.

###### 20.3 Cardiac malformations

####### 20.3.1 Cohort studies

Pooled data from two studies suggested no evidence of a difference in risk (RR 0.11, 95% CI 0.00 to 2.53, I^2^ = NA), with no difference in the number of cardiac malformations in children exposed to CBZ (N = 119) and children exposed to PRM (N = 39) (Analysis 20.3). The RD also suggested no difference in the level of risk (RD ‐0.03, 95% CI −0.10 to 0.04; I^2^ = 0%).

####### 20.3.2 Routine health record data studies

Included studies did not report any data on this outcome.

###### 20.4 Oro‐facial cleft/craniofacial malformations

####### 20.4.1 Cohort studies

We were unable to estimate a RR from two studies due to there being no reported oro‐facial cleft/craniofacial malformations in children exposed to CBZ (N = 119) or children exposed to PRM (N = 39) (Analysis 20.4).

####### 20.4.2 Routine health record data studies

Included studies did not report any data on this outcome.

###### 20.5 Skeletal/limb malformations

####### 20.5.1 Cohort studies

Pooled results from two studies suggested no evidence of a difference in risk (RR 2.84, 95% CI 0.16 to 51.53, I^2^ = NA), with no difference in the number of skeletal/limb malformations in children exposed to CBZ (N = 119) and children exposed to PRM (N = 39) (Analysis 20.5). The RD also suggested no difference in the level of risk (RD 0.03, 95% CI −0.03 to 0.09; I^2^ = 0%).

####### 20.5.2 Routine health record data studies

Included studies did not report any data on this outcome.

##### 21 CBZ versus TPM

###### 21.1 All major malformations

####### 21.1.1 Cohort studies

Pooled results from eight cohort studies suggested no evidence of a difference in risk (RR 0.83, 95% CI 0.51 to 1.33; I^2^ = 0%), with no difference in the number of major malformations in children exposed to CBZ (N = 3651) and children exposed to TPM (N = 505) (Analysis 21.1). The RD also suggested no difference in the level of risk (RD −0.01, 95% CI −0.02 to 0.01; I^2^ = 0%).

The EURAP 2018 collaboration reported the prevalence of MCM was 5.5% (95% CI 4.5 to 6.6%) for children exposed to CBZ and 3.9% (95% CI 1.5 to 8.4) for children exposed to TPM. No direct statistical comparison was made at the group level; investigations were made across different doses of the two ASMs (see Carbamazepine dose and Topiramate dose sections).

####### 21.1.2 Routine health record data studies

Pooled results from two routine health records suggested no evidence of a difference in risk (RR 0.59, 95% CI 0.17 to 2.06; I^2^ = 12%), with children exposed to CBZ (N = 1388) experiencing more major malformations than children exposed to TPM (N = 49) (Analysis 21.1). The RD also suggested no difference in the level of risk (RD ‐0.01, 95% CI −0.07 to 0.05; I^2^ = 0%).

###### 21.2 Neural tube malformations

####### 21.2.1 Cohort studies

Pooled results from seven cohort studies suggested no evidence of a difference in risk (RR 0.91, 95% CI 0.18 to 4.51; I^2^ = 0%), with no difference in the number of neural tube malformations in children exposed to CBZ (N = 3568) and children exposed to TPM (N = 496) (Analysis 21.2). The RD also suggested no difference in the level of risk (RD 0.00, 95% CI ‐0.01 to 0.01; I^2^ = 0%).

In the EURAP 2018 data, the prevalence of neural tube anomalies in those exposed to CBZ was 0.36% (7/1957) and 0% for children exposed to TPM (0/152).

####### 21.2.2 Routine health record data studies

Included studies did not report any data on this outcome.

###### 21.3 Cardiac malformations

####### 21.3.1 Cohort studies

Pooled results from eight cohort studies suggested no evidence of a difference in risk (RR 0.73, 95% CI 0.25 to 2.12; I^2^ = 0%), with no difference in the number of cardiac malformations in children exposed to CBZ (N = 3573) and children exposed to TPM (N = 497) (Analysis 21.3). The RD also suggested no difference in the level of risk (RD 0.00, 95% CI ‐0.01 to 0.01; I^2^ = 0%).

In the EURAP 2018 data, the prevalence of cardiac anomalies in those exposed to CBZ was 1.46% (28/1957) and 1.97% for children exposed to TPM (3/152).

####### 21.3.2 Routine health record data studies

Included studies did not report any data on this outcome.

###### 21.4 Oro‐facial cleft/craniofacial malformations

####### 21.4.1 Cohort studies

Pooled results from seven cohort studies suggested an increased risk with CBZ (RR 0.33, 95% CI 0.13 to 0.82; I^2^ = 40%), with children exposed to CBZ (N = 3083) experiencing more oro‐facial cleft/craniofacial malformations than children exposed to TPM (N = 488) (Analysis 21.4). However, the RD suggested no difference in the level of risk (RD ‐0.01, 95% CI ‐0.02 to 0.00; I^2^ = 0%).

In the EURAP 2018 data, the prevalence of cleft malformations (other oro‐facial not specifically reported) in those exposed to CBZ was 0.10% (2/1957) and 0% for children exposed to TPM (0/152).

####### 21.4.2 Routine health record data studies

Included studies did not report any data on this outcome.

###### 21.5 Skeletal/limb malformations

####### 21.5.1 Cohort studies

Pooled results from seven cohort studies suggested an increased risk with CBZ (RR 0.34, 95% CI 0.12 to 0.94; I^2^ = 0%), with children exposed to CBZ (N = 3568) experiencing more skeletal/limb malformations than children exposed to TPM (N = 496). However, the RD suggested no difference in the level of risk (RD ‐0.01, 95% CI ‐0.02 to 0.01; I^2^ = 0%).

####### 21.5.2 Routine health record data studies

There were no studies that provided data for this outcome.

##### 22 CBZ versus VPA

###### 22.1. All major malformations

####### 22.1.1 Cohort studies

Pooled results from 29 cohort studies suggested an increased risk with VPA (RR 0.44, 95% CI 0.37 to 0.53; I^2^ = 0%), with children exposed to CBZ (N = 5133) experiencing fewer major malformations than children exposed to VPA (N = 2957) (Analysis 22.1). The RD also suggested a higher risk for VPA (RD −0.05, 95% CI −0.06 to −0.04; I^2^ = 0%).

The EURAP 2018 collaboration reported the prevalence of MCM was 5.5% (95% CI 4.5 to 6.6%) for children exposed to CBZ and 10.3% (95% CI 8.8 to 12.0) for children exposed to VPA. No direct statistical comparison was made at the group level; investigations were made across different doses of the two ASMs (see Carbamazepine dose and Valproate dose sections). Samren 1997 reported 22 cases of major malformation out of 280 (8%) CBZ‐exposed children and six cases out of 184 (9%) VPA‐exposed children.

####### 22.1.2 Routine health record data studies

Pooled results from five routine health record studies suggested an increased risk with VPA (RR 0.42, 95% CI 0.33 to 0.54; I^2^ = 14%), with children exposed to CBZ (N = 2806) experiencing fewer major malformations than children exposed to VPA (N = 1351) (Analysis 22.1). The RD also suggested a higher risk for VPA (RD −0.06, 95% CI −0.07 to −0.04; I^2^ = 49%). Due to heterogeneity, a random‐effects RD was calculated which found a similar effect (RD ‐0.08, 95% CI ‐0.08 to ‐0.03, I^2^ = 49%).

###### 22.2 Neural tube malformations

####### 22.2.1 Cohort studies

Pooled results from 21 cohort studies suggested an increased risk with VPA (RR 0.124, 95% CI 0.14 to 0.41; I^2^ = 7%), with children exposed to CBZ (N = 4738) experiencing fewer neural tube malformations than children exposed to VPA (N = 2721) (Analysis 22.2). The RD also suggested a higher risk for VPA (RD ‐0.01, 95% CI ‐0.02 to ‐0.01; I^2^ = 14%).

In the EURAP 2018 data, the prevalence of neural tube anomalies in those exposed to CBZ was 0.36% (7/1957) and 1.6% for children exposed to VPA (16/1381).

####### 22.2.2 Routine health record data studies

Results from one routine health record study suggested no evidence of a difference in risk (RR 0.19, 95% CI 0.02 to 2.09; I^2^ = NA), with children exposed to CBZ (N = 703) experiencing comparable neural tube malformations to children exposed to VPA (N = 268) (Analysis 22.2). The RD also suggested no difference in the level of risk (RD ‐0.01, 95% CI ‐0.02 to 0.00; I^2^ = NA).

###### 22.3 Cardiac malformations

####### 22.3.1 Cohort studies

Pooled results from 22 cohort studies suggested an increased risk with VPA (RR 0.40, 95% CI 0.28 to 0.58; I^2^ = 12%), with children exposed to CBZ (N = 4743) experiencing fewer cardiac malformations than children exposed to VPA (N = 2722) (Analysis 22.3). The RD also suggested a higher risk for VPA (RD ‐0.02, 95% CI ‐0.02 to ‐0.01; I^2^ = 20%).

In the EURAP 2018 data, the prevalence of cardiac anomalies in those exposed to CBZ was 1.46% (28/1957) and 2.46% for children exposed to VPA (34/1381).

####### 22.3.2 Routine health record data studies

Results from one routine health record study suggested no evidence of a difference in risk (RR 0.38, 95% CI 0.13 to 1.08; I^2^ = NA), with children exposed to CBZ (N = 703) experiencing comparable cardiac malformations to children exposed to VPA (N = 268) (Analysis 22.3). The RD also suggested no difference in the level of risk (RD ‐0.02, 95% CI ‐0.04 to 0.00; I^2^ = NA).

###### 22.4 Oro‐facial cleft/craniofacial malformations

####### 22.4.1 Cohort studies

Pooled results from 22 cohort studies suggested an increased risk with VPA (RR 0.31, 95% CI 0.18 to 0.54; I^2^ = 0%), with children exposed to CBZ (N = 4260) experiencing fewer oro‐facial cleft/craniofacial malformations than children exposed to VPA (N = 2387) (Analysis 22.4). The RD also suggested a higher risk for VPA (RD ‐0.01, 95% CI ‐0.02 to ‐0.00; I^2^ = 0%).

In the EURAP 2018 data, the prevalence of cleft malformations (other oro‐facial not specifically reported) in those exposed to CBZ was 0.10% (2/1957) and 0.43% for children exposed to VPA (6/1381).

####### 22.4.2 Routine health record data studies

Results from one routine health record study suggested an increased risk with VPA (RR 0.15, 95% CI 0.03 to 0.78; I^2^ = NA), with children exposed to CBZ (N = 703) experiencing fewer major malformations than children exposed to VPA (N = 268) (Analysis 22.4). The RD also suggested a higher risk for VPA (RD ‐0.02, 95% CI ‐0.03 to 0.01; I^2^ = NA).

###### 22.5 Skeletal/limb malformations

####### 22.5.1 Cohort studies

Pooled results from 21 cohort studies suggested an increased risk with VPA (RR 0.31, 95% CI 0.19 to 0.51; I^2^ = 0%), with children exposed to CBZ (N = 4748) experiencing fewer skeletal/limb malformations than children exposed to VPA (N = 2711) (Analysis 22.5). The RD also suggested a higher risk for VPA (RD ‐0.01, 95% CI ‐0.02 to ‐0.01; I^2^ = 0%).

####### 22.5.2 Routine health record data studies

Results from one routine health record study suggested no evidence of a difference in risk (RR 0.38, 95% CI 0.02 to 6.07; I^2^ = NA), with children exposed to CBZ (N = 703) experiencing comparable skeletal/limb malformations to children exposed to VPA (N = 268) (Analysis 22.5). The RD also suggested no difference in the level of risk (RD ‐0.00, 95% CI ‐0.01 to 0.01; I^2^ = 0%).

##### 23 CBZ versus ZNS

###### 23.1 All major malformations

####### 23.1.1 Cohort studies

Pooled results from four cohort studies suggested no evidence of a difference in risk (RR 0.94, 95% CI 0.36 to 2.44; I^2^ = 75%), with no difference in the number of major malformations in children exposed to CBZ (N = 2711) and children exposed to ZNS (N = 130) (Analysis 23.1). Due to high heterogeneity, a random‐effects RR was calculated which also found no difference (RR 0.86, 95% CI 0.07 to 10.35, I^2^ =75%). The RD also suggested no difference in the level of risk (RD 0.00, 95% CI ‐0.03 to 0.03; I^2^ = 74%). Due to heterogeneity, a random‐effects RD was calculated which upheld a similar result (RD ‐0.02, 95% CI ‐0.15 to 0.12).

####### 23.1.2 Routine health record data studies

Included studies did not report any data on this outcome.

###### 23.2 Neural tube malformations

####### 23.2.1 Cohort studies

Pooled results from three studies suggested evidence of a difference in risk (RR 0.06, 95% CI 0.01 to 0.54, I^2^ = NA), with children exposed to CBZ (N = 1678) experiencing more neural tube malformations than children exposed to ZNS (N = 40) (Analysis 23.2). However, the RD suggested no difference in the level of risk (RD ‐0.03, 95% CI −0.10 to 0.04; I^2^ = 0%).

####### 23.2.2 Routine health record data studies

No included studies reported data on this outcome.

###### 23.3 Cardiac malformations

####### 23.3.1 Cohort studies

Pooled results from three studies suggested no evidence of a difference in risk (RR 0.47, 95% CI 0.03 to 7.72, I^2^ = NA), with no difference in the number of cardiac malformations between children exposed to CBZ (N = 1678) and children exposed to ZNS (N = 40) (Analysis 23.3). The RD also suggested no difference in the level of risk (RD 0.01, 95% CI −0.05 to 0.06; I^2^ = 0%).

####### 23.3.2 Routine health record data studies

No included studies reported data on this outcome.

###### 23.4 Oro‐facial cleft/craniofacial malformations

####### 23.4.1 Cohort studies

Pooled results from three studies suggested no evidence of a difference in risk (RR 0.15, 95% CI 0.01 to 2.66, I^2^ = NA), with no difference in the number of oro‐facial cleft/craniofacial malformations between children exposed to CBZ (N = 1678) and children exposed to ZNS (N = 40) (Analysis 23.4). The RD also suggested no difference in the level of risk (RD 0.00, 95% CI −0.06 to 0.06; I^2^ = 0%).

####### 23.4.2 Routine health record data studies

No included studies reported data on this outcome.

###### 23.5 Skeletal/limb malformations

####### 23.5.1 Cohort studies

Pooled results from three studies suggested no evidence of a difference in risk (RR 0.15, 95% CI 0.01 to 2.66, I^2^ = NA), with no difference in the number of skeletal/limb malformations between children exposed to CBZ (N = 1678) and children exposed to ZNS (N = 40) (Analysis 23.5). The RD also suggested no difference in the level of risk (RD 0.00, 95% CI −0.06 to 0.06; I^2^ = 0%).

####### 23.5.2 Routine health record data studies

No included studies reported data on this outcome.

##### 24 GBP versus LTG

###### 24.1 All major malformations

####### 24.1.1 Cohort studies

Pooled results from four cohort studies suggested no evidence of a difference in risk (RR 0.92, 95% CI 0.34 to 2.47; I^2^ = 58%), with no difference in the number of major malformations in children exposed to GBP (N = 192) and children exposed to LTG (N = 4103) (Analysis 24.1). Due to high heterogeneity, a random‐effects RR was calculated which also found no difference (RR 1.54, 95% CI 0.25 to 9.55, I^2^ =85%). The RD also suggested no difference in the level of risk (RD −0.01, 95% CI −0.03 to 0.01; I^2^ = 37%).

####### 24.1.2 Routine health record data studies

Results from one routine health record study suggested no evidence of a difference in risk (RR 0.53, 95% CI 0.03 to 9.48; I^2^ = NA), with children exposed to GBP (N = 18) experiencing more major malformations than children exposed to LTG (N = 90) (Analysis 24.1). The RD also suggested no difference in the level of risk (RD −0.04, 95% CI −0.13 to 0.01; I^2^ = 37%).

###### 24.2 Neural tube malformations

####### 24.2.1 Cohort studies

We were unable to estimate a RR from one study due to there being no reported neural tube malformations in children exposed to GBP (N = 14) or in children exposed to LTG (N = 314) (Analysis 24.2).

####### 24.2.2 Routine health record data studies

No included studies reported data on this outcome.

###### 24.3 Cardiac malformations

####### 24.3.1 Cohort studies

Pooled results from two studies suggested evidence of a difference in risk (RR 9.57, 95% CI 1.69 to 54.15, I^2^ = 30%), with children exposed to GBP (N = 16) experiencing more cardiac malformations than children exposed to LTG (N = 352) (Analysis 24.3). However, the RD suggested no difference in the level of risk (RD 0.05, 95% CI −0.08 to 0.19; I^2^ = 76%).

####### 24.3.2 Routine health record data studies

No included studies reported data on this outcome.

###### 24.4 Oro‐facial cleft/craniofacial malformations

####### 24.4.1 Cohort studies

Results from one study suggested no evidence of a difference in risk (RR 1.92, 95% CI 0.11 to 33.05, I^2^ = NA), with no difference in the number of oro‐facial cleft/craniofacial malformations between children exposed to GBP (N = 14) and children exposed to LTG (N = 315) (Analysis 24.4). The RD also suggested no difference in the level of risk (RD ‐0.02, 95% CI −0.11 to 0.08; I^2^ = NA).

####### 24.4.2 Routine health record data studies

No included studies reported data on this outcome.

###### 24.5 Skeletal/limb malformations

####### 24.5.1 Cohort studies

We were unable to estimate a RR from one study due to there being no reported skeletal/limb malformations in children exposed to GBP (N = 14) or children exposed to LTG (N = 315) (Analysis 24.5).

####### 24.5.2 Routine health record data studies

No included studies reported data on this outcome.

##### 25 GBP versus OXC

###### 25.1 All major malformations

####### 25.1.1 Cohort studies

Pooled results from three cohort studies suggested no evidence of a difference in risk (RR 0.53, 95% CI 0.13 to 2.17; I^2^ = 0%), with no difference in the number of major malformations in children exposed to GBP (N = 161) and children exposed to OXC (N = 202) (Analysis 25.1). The RD also suggested no difference in the level of risk (RD −0.01, 95% CI −0.04 to 0.01; I^2^ = 0%).

####### 25.1.2 Routine health record data studies

We were unable to estimate a RR from one study due to there being no reported major malformations in children exposed to GBP (N = 18) or children exposed to OXC (N = 4) (Analysis 25.1).

###### 25.2 Neural tube malformations

####### 25.2.1 Cohort studies

We were unable to estimate a RR from one study due to there being no reported neural tube malformations in children exposed to GBP (N = 14) or children exposed to OXC (N = 12) (Analysis 25.2).

####### 25.2.2 Routine health record data studies

No included studies reported data on this outcome.

###### 25.3 Cardiac malformations

####### 25.3.1 Cohort studies

Included studies did not reach the threshold for reporting of the meta‐analysis (Analysis 25.3). However, available data show that there were 1/15 cases of cardiac malformation in children exposed to GBP and 0/13 in OXC children, based on data from two studies (Australian Epilepsy and Pregnancy Register; Miskov 2016).

####### 25.3.2 Routine health record data studies

No included studies reported data on this outcome.

###### 25.4 Oro‐facial cleft/craniofacial malformations

####### 25.4.1 Cohort studies

We were unable to estimate a RR from one study due to there being no reported oro‐facial cleft/craniofacial malformations in children exposed to GBP (N = 14) or children exposed to OXC (N = 12) (Analysis 25.4).

####### 25.4.2 Routine health record data studies

No included studies reported data on this outcome.

###### 25.5 Skeletal/limb malformations

####### 25.5.1 Cohort studies

We were unable to estimate a RR from one study due to there being no reported skeletal/limb malformations in children exposed to GBP (N = 14) or children exposed to OXC (N = 12) (Analysis 25.5).

####### 25.5.2 Routine health record data studies

No included studies reported data on this outcome.

##### 26 GBP versus PB

###### 26.1 All major malformations

####### 26.1.1 Cohort studies

Pooled results from three cohort studies suggested no evidence of a difference in risk (RR 0.30, 95% CI 0.08 to 1.14; I^2^ = 75%), with children exposed to GBP (N = 161) experiencing no difference in major malformations to children exposed to PB (N = 204) (Analysis 26.1). Due to high heterogeneity, a random‐effects RR was calculated which also found no difference (RR 0.61, 95% CI 0.02 to 19.36, I^2^ =85%). However, the RD suggested a higher risk for PB (RD −0.04, 95% CI −0.08 to ‐0.00; I^2^ = 31%).

####### 26.1.2 Routine health record data studies

Results from one routine health record study suggested no evidence of a difference in risk (RR 0.14, 95% CI 0.01 to 3.09; I^2^ = NA), with children exposed to GBP (N = 18) experiencing more major malformations than children exposed to PB (N = 1) (Analysis 26.1). The RD also suggested no difference in the level of risk (RD ‐0.14, 95% CI ‐0.42 to 0.14).

###### 26.2 Neural tube malformations

####### 26.2.1 Cohort studies

We were unable to estimate a RR from one study due to there being no reported neural tube malformations in children exposed to GBP (N = 14) or children exposed to PB (N = 5) (Analysis 26.2).

####### 26.2.2 Routine health record data studies

No included studies reported data on this outcome.

###### 26.3 Cardiac malformations

####### 26.3.1 Cohort studies

Included studies did not reach the threshold for reporting of the meta‐analysis (Analysis 26.3). However, available data show that there were 1/16 cases of cardiac malformation in children exposed to GBP and 0/8 in children exposed to PB, based on data from two studies (Australian Epilepsy and Pregnancy Register; Miskov 2016).

####### 26.3.2 Routine health record data studies

No included studies reported data on this outcome.

###### 26.4 Oro‐facial cleft/craniofacial malformations

####### 26.4.1 Cohort studies

We were unable to estimate a RR from one study due to there being no reported oro‐facial cleft/craniofacial malformations in children exposed to GBP (N = 14) or children exposed to PB (N = 5) (Analysis 26.4).

####### 26.4.2 Routine health record data studies

No included studies reported data on this outcome.

###### 26.5 Skeletal/limb malformations

####### 26.5.1 Cohort studies

We were unable to estimate a RR from one study due to there being no reported skeletal/limb malformations in children exposed to GBP (N = 14) or children exposed to PB (N = 5) (Analysis 26.5).

####### 26.5.2 Routine health record data studies

No included studies reported data on this outcome.

##### 27 GBP versus PRM

###### 27.1 All major malformations

####### 27.1.1 Cohort studies

No included studies reported data on this outcome.

####### 27.1.2 Routine health record data studies

We were unable to estimate a RR from one study due to there being no reported major malformations in children exposed to GBP (N = 18) or children exposed to PRM (N = 8) (Analysis 27.1).

###### 27.2 Neural tube malformations

####### 27.2.1 Cohort studies

No included studies reported data on this outcome.

####### 27.2.2 Routine health record data studies

No included studies reported data on this outcome.

###### 27.3 Cardiac malformations

####### 27.3.1 Cohort studies

No included studies reported data on this outcome.

####### 27.3.2 Routine health record data studies

No included studies reported data on this outcome.

###### 27.4 Oro‐facial cleft/craniofacial malformations

####### 27.4.1 Cohort studies

No included studies reported data on this outcome.

####### 27.4.2 Routine health record data studies

No included studies reported data on this outcome.

###### 27.5 Skeletal/limb malformations

####### 27.5.1 Cohort studies

No included studies reported data on this outcome.

####### 27.5.2 Routine health record data studies

No included studies reported data on this outcome.

##### 28 GBP versus TPM

###### 28.1 All major malformations

####### 28.1.1 Cohort studies

Pooled results from three cohort studies suggested no evidence of a difference in risk (RR 0.32, 95% CI 0.09 to 1.19; I^2^ = 0%), with no difference in the number of major malformations in children exposed to GBP (N = 190) and children exposed to TPM (N = 482) (Analysis 28.1). However, the RD suggested a higher risk for TPM (RD −0.03, 95% CI −0.05 to −0.01; I^2^ = 0%).

####### 28.1.2 Routine health record data studies

We were unable to estimate a RR from one study due to there being no reported major malformations in children exposed to GBP (N = 18) or children exposed to TPM (N = 1) (Analysis 28.1).

###### 28.2 Neural tube malformations

####### 28.2.1 Cohort studies

We were unable to estimate a RR from one study due to there being no reported neural tube malformations in children exposed to GBP (N = 14) or children exposed to TPM (N = 44) (Analysis 28.2).

####### 28.2.2 Routine health record data studies

No included studies reported data on this outcome.

###### 28.3 Cardiac malformations

####### 28.3.1 Cohort studies

We were unable to estimate a RR from one study due to there being no reported cardiac malformations in children exposed to GBP (N = 14) or children exposed to TPM (N = 44) (Analysis 28.3).

####### 28.3.2 Routine health record data studies

No included studies reported data on this outcome.

###### 28.4 Oro‐facial cleft/craniofacial malformations

####### 28.4.1 Cohort studies

We were unable to estimate a RR from one study due to there being no reported oro‐facial cleft/craniofacial malformations in children exposed to GBP (N = 14) or children exposed to TPM (N = 44) (Analysis 28.4).

####### 28.4.2 Routine health record data studies

No included studies reported data on this outcome.

###### 28.5 Skeletal/limb malformations

####### 28.5.1 Cohort studies

We were unable to estimate a RR from one study due to there being no reported skeletal/limb malformations in children exposed to GBP (N = 14) or children exposed to TPM (N = 44) (Analysis 28.5).

####### 28.5.2 Routine health record data studies

No included studies reported data on this outcome.

##### 29 GBP versus ZNS

###### 29.1 All major malformations

####### 29.1.1 Cohort studies

Data from two cohort studies suggested no evidence of a difference in risk (RR 0.53, 95% CI 0.10 to 2.76; I^2^ = 0%), with no difference in the number of major malformations in children exposed to GBP (N = 176) and children exposed to ZNS (N = 116) (Analysis 29.1). The RD also suggested no difference in the level of risk (RD ‐0.01, 95% CI −0.04 to 0.02; I^2^ = 72%). Due to heterogeneity, a random‐effects RD was calculated and upheld a similar finding (RD ‐0.03, 95% CI ‐0.15 to 0.10, I^2^ =72%).

####### 29.1.2 Routine health record data studies

No included studies reported data on this outcome.

###### 29.2 Neural tube malformations

####### 29.2.1 Cohort studies

No included studies reported data on this outcome.

####### 29.2.2 Routine health record data studies

No included studies reported data on this outcome.

###### 29.3 Cardiac malformations

####### 29.3.1 Cohort studies

No included studies reported data on this outcome.

####### 29.3.2 Routine health record data studies

No included studies reported data on this outcome.

###### 29.4 Oro‐facial cleft/craniofacial malformations

####### 29.4.1 Cohort studies

No included studies reported data on this outcome.

####### 29.4.2 Routine health record data studies

No included studies reported data on this outcome.

###### 29.5 Skeletal/limb malformations

####### 29.5.1 Cohort studies

No included studies reported data on this outcome.

####### 29.5.2 Routine health record data studies

No included studies reported data on this outcome.

##### 30 LEV versus GBP

###### 30.1 All major malformations

####### 30.1.1 Cohort studies

Pooled results from three studies suggested no evidence of a difference in risk (RR 1.61, 95% CI 0.46 to 5.63; I^2^ = 43%), with no difference in the number of major malformations in children exposed to LEV (N = 893) and children exposed to GBP (N = 190) (Analysis 30.1). The RD also suggested no difference in the level of risk (RD 0.01, 95% CI −0.01 to 0.03; I^2^ = 0%).

####### 30.1.2 Routine health record data studies

No included studies reported data on this outcome.

###### 30.2 Neural tube malformations

####### 30.2.1 Cohort studies

We were unable to estimate a RR from one study due to there being no reported neural tube malformations in children exposed to LEV (N = 63) or children exposed to GBP (N = 14) (Analysis 30.2).

####### 30.2.2 Routine health record data studies

No included studies reported data on this outcome.

###### 30.3 Cardiac malformations

####### 30.3.1 Cohort studies

Results from one study suggested no evidence of a difference in risk (RR 0.70, 95% CI 0.03 to 16.42, I^2^ = NA), with no difference in the number of cardiac malformations in children exposed to LEV (N = 63) and children exposed to GBP (N = 14) (Analysis 30.3). The RD also suggested no difference in the level of risk (RD 0.02, 95% CI −0.08 to 0.11; I^2^ = NA).

####### 30.3.2 Routine health record data studies

No included studies reported data on this outcome.

###### 30.4 Oro‐facial cleft/craniofacial malformations

####### 30.4.1 Cohort studies

Results from one study suggested no evidence of a difference in risk (RR 0.70, 95% CI 0.03 to 16.42, I^2^ = NA), with no difference in the number of cardiac malformations in children exposed to LEV (N = 63) and children exposed to GBP (N = 14) (Analysis 30.4). The RD also suggested no difference in the level of risk (RD 0.02, 95% CI −0.08 to 0.11; I^2^ = NA).

####### 30.4.2 Routine health record data studies

No included studies reported data on this outcome.

###### 30.5 Skeletal/limb malformations

####### 30.5.1 Cohort studies

We were unable to estimate a RR from one study due to there being no reported skeletal/limb malformations in children exposed to LEV (N = 63) or children exposed to GBP (N = 14) (Analysis 30.5).

####### 30.5.2 Routine health record data studies

No included studies reported data on this outcome.

##### 31 LEV versus LTG

###### 31.1. All major malformations

####### 31.1.1 Cohort studies

Pooled results from 10 cohort studies suggested no evidence of a difference in risk (RR 0.90, 95% CI 0.58 to 1.39; I^2^ = 16%), with no difference in the number of major malformations in children exposed to LEV (N = 1223) and children exposed to LTG (N = 4389) (Analysis 31.1). The RD also suggested no difference in the level of risk (RD −0.00, 95% CI −0.01 to 0.01; I^2^ = 10%).

The EURAP 2018 collaboration reported the prevalence of MCM was 2.8% (95% CI 1.7 to 4.5%) for children exposed to LEV and 2.9% (95% CI 2.3 to 3.7) for children exposed to LTG. No direct statistical comparison was made at the group level; investigations were made across different doses of the two ASMs (see Levetiracetam dose and Lamotrigine dose sections).

####### 31.1.2 Routine health record data studies

Pooled results from two routine health record studies suggested no evidence of a difference in risk (RR 0.79, 95% CI 0.37 to 1.69; I^2^ = 0%), with no difference in the number of major malformations in children exposed to LEV (N = 248) and children exposed to LTG (N = 2068) (Analysis 31.1). The RD also suggested no difference in the level of risk (RD ‐0.01, 95% CI −0.03 to 0.01; I^2^ = 0%).

###### 31.2 Neural tube malformations

####### 31.2.1 Cohort studies

Pooled results from nine cohort studies suggested no evidence of a difference in risk (RR 1.59, 95% CI 0.24 to 10.38; I^2^ = 0%), with no difference in the number of neural tube malformations in children exposed to LEV (N = 1128) and children exposed to LTG (N = 4245) (Analysis 31.2). The RD also suggested no difference in the level of risk (RD 0.00, 95% CI ‐0.00 to 0.00; I^2^ = 0%).

In the EURAP 2018 data, the prevalence of neural tube anomalies in those exposed to LEV was 0% (0/599) and 0.04% for children exposed to LTG (1/2514).

####### 31.2.2 Routine health record data studies

No included studies reported data on this outcome.

###### 31.3 Cardiac malformations

####### 31.3.1 Cohort studies

Pooled results from nine cohort studies suggested no evidence of a difference in risk (RR 1.20, 95% CI 0.51 to 2.85; I^2^ = 0%), with no difference in the number of cardiac malformations in children exposed to LEV (N = 1125) and children exposed to LTG (N = 4246) (Analysis 31.3). The RD also suggested no difference in the level of risk (RD 0.00, 95% CI ‐0.00 to 0.01; I^2^ = 0%).

In the EURAP 2018 data, the prevalence of cardiac anomalies in those exposed to LEV was 0.83% (5/599) and 0.59% for children exposed to LTG (15/2514).

####### 32.3.2 Routine health record data studies

No included studies reported data on this outcome.

###### 31.4 Oro‐facial cleft/craniofacial malformations

####### 31.4.1 Cohort studies

Pooled results from eight cohort studies suggested no evidence of a difference in risk (RR 0.63, 95% CI 0.15 to 2.68; I^2^ = 0%), with no difference in the number of oro‐facial cleft/craniofacial malformations in children exposed to LEV (N = 1019) and children exposed to LTG (N = 4196) (Analysis 31.4). The RD also suggested no difference in the level of risk (RD ‐0.00, 95% CI ‐0.01 to 0.00; I^2^ = 0%).

In the EURAP 2018 data, the prevalence of cleft malformations (other oro‐facial not specifically reported) in those exposed to LEV was 0.16% (1/599) and 0.43% for children exposed to LTG (3/2514).

####### 31.4.2 Routine health record data studies

No included studies reported data on this outcome.

###### 31.5 Skeletal/limb malformations

####### 31.5.1 Cohort studies

Pooled results from nine cohort studies suggested no evidence of a difference in risk (RR 1.36, 95% CI 0.45 to 4.13; I^2^ = 0%), with no difference in the number of skeletal/limb malformations in children exposed to LEV (N = 1128) and children exposed to LTG (N = 4245) (Analysis 31.5). The RD also suggested no difference in the level of risk (RD −0.00, 95% CI −0.01 to 0.00; I^2^ = 0%).

####### 31.5.2 Routine health record data studies

No included studies reported data on this outcome.

##### 32 LEV versus OXC

###### 32.1 All major malformations

####### 32.1.1 Cohort studies

Pooled results from eight cohort studies suggested no evidence of a difference in risk (RR 1.04, 95% CI 0.51 to 2.09; I^2^ = 0%), with no difference in the number of major malformations in children exposed to LEV (N = 833) and children exposed to OXC (N = 333) (Analysis 32.1). The RD also suggested no difference in the level of risk (RD 0.00, 95% CI −0.02 to 0.03; I^2^ = 0%).

The EURAP 2018 collaboration reported the prevalence of MCM was 2.8% (95% CI 1.7 to 4.5%) for children exposed to LEV and 3.0% (95% CI 1.4 to 5.4) for children exposed to OXC. No direct statistical comparison was made at the group level; investigations were made across different doses of the two ASMs (see Levetiracetam dose and Oxcarbazepine dose sections).

####### 32.1.2 Routine health record data studies

Pooled results from two routine health record studies suggested no evidence of a difference in risk (RR 1.17, 95% CI 0.45 to 3.06; I^2^ = 0%), with no difference in the number of major malformations in children exposed to LEV (N = 248) and children exposed to OXC (N = 373) (Analysis 32.1). The RD also suggested no difference in the level of risk (RD 0.00, 95% CI −0.02 to 0.03; I^2^ = 0%).

###### 32.2 Neural tube malformations

####### 32.2.1 Cohort studies

Pooled results from seven cohort studies suggested no evidence of a difference in risk (RR 1.22, 95% CI 0.05 to 29.74; I^2^ = NA), with no difference in the number of neural tube malformations in children exposed to LEV (N = 738) and children exposed to OXC (N = 320) (Analysis 32.2). The RD also suggested no difference in the level of risk (RD 0.00, 95% CI ‐0.01 to 0.01; I^2^ = 0%).

In the EURAP 2018 data, the prevalence of neural tube anomalies in those exposed to LEV was 0% (0/599) and 0% for children exposed to OXC (0/333).

####### 32.2.2 Routine health record data studies

No included studies reported data on this outcome.

###### 32.3 Cardiac malformations

####### 32.3.1 Cohort studies

Pooled results from eight cohort studies suggested no evidence of a difference in risk (RR 0.93, 95% CI 0.31 to 2.76; I^2^ = 0%), with no difference in the number of cardiac malformations in children exposed to LEV (N = 747) and children exposed to OXC (N = 323) (Analysis 32.3). The RD also suggested no difference in the level of risk (RD 0.00, 95% CI ‐0.01 to 0.02; I^2^ = 0%).

In the EURAP 2018 data, the prevalence of cardiac anomalies in those exposed to LEV was 0.83% (5/599) and 1.2% for children exposed to OXC (4/333).

####### 32.3.2 Routine health record data studies

No included studies reported data on this outcome.

###### 32.4 Oro‐facial cleft/craniofacial malformations

####### 32.4.1 Cohort studies

Pooled results from seven cohort studies suggested no evidence of a difference in risk (RR 0.25, 95% CI 0.03 to 2.12; I^2^ = 0%), with no difference in the number of oro‐facial cleft/craniofacial malformations in children exposed to LEV (N = 641) and children exposed to OXC (N = 252) (Analysis 32.4). The RD also suggested no difference in the level of risk (RD ‐0.00, 95% CI ‐0.02 to 0.01; I^2^ = 0%).

In the EURAP 2018 data, the prevalence of cardiac anomalies in those exposed to LEV was 0.83% (5/599) and 0.30% for children exposed to OXC (1/333).

####### 32.4.2 Routine health record data studies

No included studies reported data on this outcome.

###### 32.5 Skeletal/limb malformations

####### 32.5.1 Cohort studies

Pooled results from seven cohort studies suggested no evidence of a difference in risk (RR 0.80, 95% CI 0.20 to 3.29; I^2^ = 0%), with no difference in the number of skeletal/limb malformations in children exposed to LEV (N = 738) children exposed to OXC (N = 320) (Analysis 32.5). The RD also suggested no difference in the level of risk (RD ‐0.00, 95% CI ‐0.02 to 0.02; I^2^ = 0%).

####### 32.5.2 Routine health record data studies

No included studies reported data on this outcome.

##### 33 LEV versus PB

###### 33.1 All major malformations

####### 33.1.1 Cohort studies

Results from five cohort studies suggested no evidence of a difference in risk (RR 0.54, 95% CI 0.29 to 1.02; I^2^ = 0%), with children exposed to LEV (N = 726) experiencing comparable major malformations to children exposed to PB (N = 341) (Analysis 33.1). The RD also suggested no difference in the level of risk (RD −0.02, 95% CI −0.05 to 0.01; I^2^ = 0%).

The EURAP 2018 collaboration reported the prevalence of MCM was 2.8% (95% CI 1.7 to 4.5%) for children exposed to LEV and 6.5% (95% CI 4.2 to 9.9) for children exposed to PB. No direct statistical comparison was made at the group level; investigations were made across different doses of the two ASMs (see Levetiracetam dose and Phenobarbital dose sections).

####### 33.1.2 Routine health record data studies

Results from one routine health record study suggested no evidence of a difference in risk (RR 0.23, 95% CI 0.03 to 1.55; I^2^ = NA), with children exposed to LEV (N = 118) experiencing comparable major malformation rates to children exposed to PB (N = 27). The RD also suggested no difference in the level of risk (RD −0.06, 95% CI −0.16 to 0.04; I^2^ = NA).

###### 33.2 Neural tube malformations

####### 33.2.1 Cohort studies

Results from five cohort studies suggested no evidence of a difference in risk (RR 0.74, 95% CI 0.08 to 6.51; I^2^ = 0%), with no difference in the number of neural tube malformations in children exposed to LEV (N = 650) and children exposed to PB (N = 344) (Analysis 33.2). The RD also suggested no difference in the level of risk (RD ‐0.00, 95% CI ‐0.01 to 0.01; I^2^ = 0%).

In the EURAP 2018 data, the prevalence of neural tube anomalies in those exposed to LEV was 0% (0/599) and 0.68% for children exposed to PB (2/294).

####### 33.2.2 Routine health record data studies

No included studies reported data on this outcome.

###### 33.3 Cardiac malformations

####### 33.3.1 Cohort studies

Pooled results from five cohort studies suggested no evidence of a difference in risk (RR 0.33, 95% CI 0.12 to 0.88; I^2^ = 17%), with no difference in the number of cardiac malformations in children exposed to LEV (N = 650) and PB (N = 344) (Analysis 33.3). The RD also suggested no difference in the level of risk (RD ‐0.02, 95% CI ‐0.04 to 0.00; I^2^ = 0%).

In the EURAP 2018 data, the prevalence of cardiac anomalies in those exposed to LEV was 0.83% (5/599) and 2.72% for children exposed to PB (8/294%).

####### 33.3.2 Routine health record data studies

No included studies reported data on this outcome.

###### 33.4 Oro‐facial cleft/craniofacial malformations

####### 33.4.1 Cohort studies

Pooled results from four cohort studies suggested an increased risk with PB (RR 0.08, 95% CI 0.01 to 0.67; I^2^ = 0%), with children exposed to LEV (N = 544) experiencing fewer oro‐facial cleft/craniofacial malformations than children exposed to PB (N = 207) (Analysis 33.4). The RD suggested no difference in the level of risk (RD ‐0.02, 95% CI ‐0.04 to 0.01; I^2^ = 0%).

In the EURAP 2018 data, the prevalence of cleft malformations (other oro‐facial not specifically reported) in those exposed to LEV was 0.16% (1/599) and 0.34% for children exposed to PB (1/294).

####### 33.4.2 Routine health record data studies

No included studies reported data on this outcome.

###### 33.5 Skeletal/limb malformations

####### 33.5.1 Cohort studies

Pooled results from five cohort studies suggested no evidence of a difference in risk (RR 0.67, 95% CI 0.15 to 2.94; I^2^ = 23%), with no difference in the number of skeletal/limb malformations in children exposed to LEV (N = 650) and children exposed to PB (N = 344) (Analysis 33.5). The RD also suggested no difference in the level of risk (RD ‐0.00, 95% CI ‐0.02 to 0.01; I^2^ = 0%).

####### 33.5.2 Routine health record data studies

No included studies reported data on this outcome.

##### 34 LEV versus PHT

###### 34.1 All major malformations

####### 34.1.1 Cohort studies

Pooled results from five cohort studies suggested an increased risk with PHT (RR 0.58, 95% CI 0.34 to 0.97; I^2^ = 58%), with children exposed to LEV (N = 1018) experiencing fewer major malformations than children exposed to PHT (N = 687) (Analysis 34.1). Due to high heterogeneity, we undertook a random‐effects analysis, which changed found no evidence of a difference in risk (RR 0.46, 95% CI 0.17 to 1.28; I^2^ = 58%). The RD also suggested no difference in the level of risk (RD −0.02, 95% CI −0.04 to −0.00; I^2^ = 52%). Due to high heterogeneity, we undertook a random‐effects analysis, which also found no difference (RD −0.02, 95% CI −0.05 to 0.02; I^2^ = 52%).

The EURAP 2018 collaboration reported the prevalence of MCM was 2.8% (95% CI 1.7 to 4.5%) for children exposed to LEV and 6.4% (95% CI 2.8 to 12.2) for children exposed to PHT. No direct statistical comparison was made at the group level; investigations were made across different doses of the two ASMs (see Levetiracetam dose and Phenytoin dose sections).

####### 34.1.2 Routine health record data studies

No included studies reported data on this outcome.

###### 34.2 Neural tube malformations

####### 34.2.1 Cohort studies

Pooled results from four cohort studies suggested no evidence of a difference in risk (RR 0.68, 95% CI 0.13 to 3.44; I^2^ = 0%), with no difference in the number of neural tube malformations in children exposed to LEV (N = 913) and children exposed to PHT (N = 661) (Analysis 34.2). The RD also suggested no difference in the level of risk (RD ‐0.00, 95% CI ‐0.01 to 0.01; I^2^ = 0%).

In the EURAP 2018 data, the prevalence of neural tube anomalies in those exposed to LEV was 0% (0/599) and 0.80% for children exposed to PHT (1/125).

####### 34.2.2 Routine health record data studies

No included studies reported data on this outcome.

###### 34.3 Cardiac malformations

####### 34.3.1 Cohort studies

Pooled results from four cohort studies suggested no evidence of a difference in risk (RR 0.43, 95% CI 0.16 to 1.13; I^2^ = 0%), with no difference in the number of cardiac malformations in children exposed to LEV (N = 911) and children exposed to PHT (N = 611) (Analysis 34.3). The RD also suggested no difference in the level of risk (RD ‐0.01, 95% CI ‐0.02 to 0.00; I^2^ = 0%).

In the EURAP 2018 data, the prevalence of cardiac anomalies in those exposed to LEV was 0.83% (5/599) and 4.0% for children exposed to PHT (5/125).

####### 34.3.2 Routine health record data studies

No included studies reported data on this outcome.

###### 34.4 Oro‐facial cleft/craniofacial malformations

####### 34.4.1 Cohort studies

Pooled results from three studies suggested no evidence of a difference in risk (RR 0.37, 95% CI 0.09 to 1.61; I^2^ = 4%), with no difference in the number of oro‐facial cleft/craniofacial malformations in children exposed to LEV (N = 807) and children exposed to PHT (N = 542) (Analysis 34.4). The RD also suggested no difference in the level of risk (RD ‐0.00, 95% CI ‐0.01 to 0.01; I^2^ = 0%).

In the EURAP 2018 data, the prevalence of cleft malformations (other oro‐facial not specifically reported) in those exposed to LEV was 0.16% (1/599) and 0% for children exposed to PHT (0/125).

####### 34.4.2 Routine health record data studies

No included studies reported data on this outcome.

###### 34.5 Skeletal/limb malformations

####### 34.5.1 Cohort studies

Pooled results from four studies suggested no evidence of a difference in risk (RR 0.46, 95% CI 0.11 to 1.96; I^2^ = 63%), with no difference in the number of skeletal/limb malformations in children exposed to LEV (N = 913) and children exposed to PHT (N = 661) (Analysis 34.5). Due to high heterogeneity, a random‐effects RR was calculated, which also found no difference (RR 0.54, 95% CI 0.02 to 11.85, I^2^ =63%). The RD also suggested no difference in the level of risk (RD ‐0.00, 95% CI ‐0.01 to 0.00; I^2^ = 0%).

####### 34.5.2 Routine health record data studies

No included studies reported data on this outcome.

##### 35 LEV versus PRM

###### 35.1 All major malformations

####### 35.1.1. Cohort studies

Results from one study suggested no evidence of a difference in risk (RR 0.24, 95% CI 0.02 to 3.37, I^2^ = NA), with no difference in the number of major malformations in children exposed to LEV (N = 139) and children exposed to PRM (N = 2) (Analysis 35.1). The RD also suggested no difference in the level of risk (RD 0.04, 95% CI −0.39 to 0.46; I^2^ = NA).

####### 35.1.2. Routine health record studies

No included studies reported data on this outcome.

###### 35.2 Neural tube malformations

####### 35.2.1. Cohort studies

No included studies reported data on this outcome.

####### 35.2.2. Routine health record studies

No included studies reported data on this outcome.

###### 35.3 Cardiac malformations

####### 35.3.1. Cohort studies

No included studies reported data on this outcome.

####### 35.3.2. Routine health record studies

No included studies reported data on this outcome.

###### 35.4 Oro‐facial cleft/craniofacial malformations

####### 35.4.1. Cohort studies

No included studies reported data on this outcome.

####### 35.4.2. Routine health record studies

No included studies reported data on this outcome.

###### 35.5 Skeletal/limb malformations

####### 35.5.1. Cohort studies

No included studies reported data on this outcome.

####### 35.5.2. Routine health record studies

No included studies reported data on this outcome.

##### 36 LEV versus TPM

###### 36.1 All major malformations

####### 36.1.1 Cohort studies

Pooled results from eight cohort studies suggested no evidence of a difference in risk (RR 0.57, 95% CI 0.32 to 1.04; I^2^ = 0%), with children exposed to LEV (N = 1124) experiencing comparable major malformations to children exposed to TPM (N = 505) (Analysis 36.1). The RD also suggested no difference in the level of risk (RD −0.02, 95% CI −0.04 to 0.00; I^2^ = 0%).

The EURAP 2018 collaboration reported the prevalence of MCM was 2.8% (95% CI 1.7 to 4.5%) for children exposed to LEV and 3.9% (95% CI 1.5 to 8.4) for children exposed to TPM. No direct statistical comparison was made at the group level; investigations were made across different doses of the two ASMs (see Levetiracetam dose and Topiramate dose sections).

####### 36.1.2 Routine health record studies

Results from one routine health record study suggested no evidence of a difference in risk (RR 0.41, 95% CI 0.06 to 2.81; I^2^ = NA), with children exposed to LEV (N = 118) experiencing comparable major malformation rate to children exposed to TPM (N = 48) (Analysis 36.1). The RD also suggested no difference in the level of risk (RD −0.02, 95% CI −0.09 to 0.04; I^2^ = NA).

###### 36.2 Neural tube malformations

####### 36.2.1 Cohort studies

Pooled results from seven cohort studies suggested no evidence of a difference in risk (RR 2.39, 95% CI 0.10 to 58.61; I^2^ = NA), with no difference in the number of neural tube malformations in children exposed to LEV (N = 1030) and children exposed to TPM (N = 496) (Analysis 36.2). The RD also suggested no difference in the level of risk (RD 0.00, 95% CI −0.01 to 0.01; I^2^ = 0%).

In the EURAP 2018 data, the prevalence of neural tube anomalies in those exposed to LEV was 0% (0/599) and 0% for children exposed to TPM (0/152).

####### 36.2.2 Routine health record studies

No included studies reported data on this outcome.

###### 36.3 Cardiac malformations

####### 36.3.1 Cohort studies

Pooled results from eight studies suggested no evidence of a difference in risk (RR 0.72, 95% CI 0.21 to 2.53; I^2^ = 0%), with no difference in the number of cardiac malformations in children exposed to LEV (N = 1039) and children exposed to TPM (N = 497) (Analysis 36.2). The RD also suggested no difference in the level of risk (RD ‐0.00, 95% CI −0.01 to 0.01; I^2^ = 0%).

In the EURAP 2018 data, the prevalence of cardiac anomalies in those exposed to LEV was 0.83% (5/599) and 1.97% for children exposed to TPM (3/152).

####### 36.3.2 Routine health record studies

No included studies reported data on this outcome.

###### 36.4 Oro‐facial cleft/craniofacial malformations

####### 36.4.1 Cohort studies

Pooled results from seven cohort studies suggested an increased risk with TPM (RR 0.19, 95% CI 0.05 to 0.70; I^2^ = 48%), with children exposed to LEV (N = 933) experiencing fewer oro‐facial/craniofacial malformations than children exposed to TPM (N = 488) (Analysis 36.4). However, the RD suggested no difference in the level of risk (RD ‐0.01, 95% CI −0.03 to 0.00; I^2^ = 0%).

In the EURAP 2018 data, the prevalence of cleft malformations (other oro‐facial not specifically reported) in those exposed to LEV was 0.16% (1/599) and 0% for children exposed to TPM (0/125).

####### 36.4.2 Routine health record studies

No included studies reported data on this outcome.

###### 36.5 Skeletal/limb malformations

####### 36.5.1 Cohort studies

Pooled results from seven cohort studies suggested an increased risk with TPM (RR 0.12, 95% CI 0.02 to 0.98; I^2^ = 0%), with children exposed to LEV (N = 1030) experiencing fewer skeletal/limb malformations than children exposed to TPM (N = 496) (Analysis 36.5). However, the RD suggested no difference in the level of risk (RD ‐0.01, 95% CI −0.02 to 0.00; I^2^ = 0%).

####### 36.5.2 Routine health record studies

No included studies reported data on this outcome.

##### 37 LEV versus ZNS

###### 37.1 All major malformations

####### 37.1.1 Cohort studies

Pooled results from four cohort studies suggested no evidence of a difference in risk (RR 0.66, 95% CI 0.25 to 1.71; I^2^ = 79%), with no difference in the number of major malformations in children exposed to LEV (N = 865) and children exposed to ZNS (N = 130) (Analysis 37.1). Due to high heterogeneity, a random‐effects RR was calculated, which also found no difference (RD 0.48, 95% CI 0.03 to 7.24, I^2^ = 79%). The RD also suggested no difference in the level of risk (RD 0.01, 95% CI ‐0.04 to 0.03; I^2^ = 76%). Due to heterogeneity, a random‐effects RD was calculated, which maintained similar findings (RD ‐0.03, 95% CI ‐0.16 to 0.10, I^2^ = 76%).

####### 37.1.2 Routine health record studies

No included studies reported data on this outcome.

###### 37.2 Neural tube malformations

####### 37.2.1 Cohort studies

Pooled results from three included studies suggested evidence of a difference in risk (RR 0.03, 95% CI 0.00 to 0.71, I^2^ = NA), with children exposed to ZNS (N = 40) experiencing more neural tube malformations than children exposed to LEV (N = 415) (Analysis 37.2). However, the RD suggested no difference in the level of risk (RD ‐0.03, 95% CI −0.10 to 0.05; I^2^ = 0%).

####### 37.2.2 Routine health record studies

No included studies reported data on this outcome.

###### 37.3 Cardiac malformations

####### 37.3.1 Cohort studies

Pooled results from three included studies suggested no evidence of a difference in risk (RR 0.98, 95% CI 0.05 to 17.99, I^2^ = NA), with no difference in cardiac malformations between children exposed to LEV (N = 415) and children exposed to ZNS (N = 40) (Analysis 37.3). The RD suggested no difference in the level of risk (RD 0.01, 95% CI −0.05 to 0.07; I^2^ = 0%).

####### 37.3.2 Routine health record studies

No included studies reported data on this outcome.

###### 37.4 Oro‐facial cleft/craniofacial malformations

####### 37.4.1 Cohort studies

We were unable to estimate a RR from three studies due to there being no reported oro‐facial cleft/craniofacial malformations in children exposed to LEV (N = 415) and ZNS (N = 40) (Analysis 37.4).

####### 37.4.2 Routine health record studies

No included studies reported data on this outcome.

###### 37.5 Skeletal/limb malformations

####### 37.5.1 Cohort studies

We were unable to estimate a RR from three studies due to there being no reported skeletal/limb malformations in children exposed to LEV (N = 415) and ZNS (N = 40) (Analysis 37.5).

####### 37.5.2 Routine health record studies

No included studies reported data on this outcome.

##### 38 LTG versus CZP

###### 38.1 All major malformations

####### 38.1.1 Cohort studies

Pooled results from three cohort studies suggested no evidence of a difference in risk (RR 0.92, 95% CI 0.29 to 2.91; I^2^ = 0%), with no difference in the number of major malformations in children exposed to LTG (N = 2018) and children exposed to CZP (N = 94) (Analysis 38.1). The RD also suggested no difference in the level of risk (RD 0.01, 95% CI ‐0.03 to 0.04; I^2^ = 33%).

####### 38.1.2 Routine health record studies

Pooled results from two routine health record studies suggested no evidence of a difference in risk (RR 1.54, 95% CI 0.53 to 4.54; I^2^ = 0%), with no difference in the number of major malformations in children exposed to LTG (N = 923) and children exposed to CZP (N = 161) (Analysis 38.1). The RD also suggested no difference in the level of risk (RD 0.01, 95% CI −0.01 to 0.04; I^2^ = 0%).

###### 38.2 Neural tube malformations

####### 38.2.1 Cohort studies

No included studies reported data on this outcome.

####### 38.2.2 Routine health record studies

No included studies reported data on this outcome.

###### 38.3 Cardiac malformations

####### 38.3.1 Cohort studies

No included studies reported data on this outcome.

####### 38.3.2 Routine health record studies

No included studies reported data on this outcome.

###### 38.4 Oro‐facial cleft/craniofacial malformations

####### 38.4.1 Cohort studies

No included studies reported data on this outcome.

####### 38.4.2 Routine health record studies

No included studies reported data on this outcome.

###### 38.5 Skeletal/limb malformations

####### 38.5.1 Cohort studies

No included studies reported data on this outcome.

####### 38.5.2 Routine health record studies

No included studies reported data on this outcome.

##### 39 LTG versus LAC

###### 39.1 All major malformations

####### 39.1.1 Cohort studies

We were unable to estimate the RR from one study due to there being no major malformations observed in children exposed to LTG (N = 19) or LAC (N = 1) (Analysis 39.1).

####### 39.1.2 Routine health record studies

No included studies reported data on this outcome.

###### 39.2 Neural tube malformations

####### 39.2.1 Cohort studies

We were unable to estimate a RR from one study due to there being no reported neural tube malformations in children exposed to LTG (N = 19) or LAC (N = 1) (Analysis 39.2).

####### 39.2.2 Routine health record studies

No included studies reported data on this outcome.

###### 39.3 Cardiac malformations

####### 39.3.1 Cohort studies

We were unable to estimate a RR from one study due to there being no reported cardiac malformations in children exposed to LTG (N = 19) or LAC (N = 1) (Analysis 39.3).

####### 39.3.2 Routine health record studies

No included studies reported data on this outcome.

###### 39.4 Oro‐facial cleft/craniofacial malformations

####### 39.4.1 Cohort studies

We were unable to estimate a RR from one study due to there being no reported oro‐facial cleft/craniofacial malformations in children exposed to LTG (N = 19) or LAC (N = 1) (Analysis 39.3).

####### 39.4.2 Routine health record studies

No included studies reported data on this outcome.

###### 39.5 Skeletal/limb malformations

####### 39.5.1 Cohort studies

We were unable to estimate a RR from one study due to there being no reported skeletal/limb malformations in children exposed to LTG (N = 19) or LAC (N = 1) (Analysis 39.3).

####### 39.5.2 Routine health record studies

No included studies reported data on this outcome.

##### 40 LTG versus OXC

###### 40.1 All major malformations

####### 40.1.1 Cohort studies

Pooled results from eight cohort studies suggested no evidence of a difference in risk(RR 0.73, 95% CI 0.33 to 1.62; I^2^ = 0%), with no difference in the number of major malformations in children exposed to LTG (N = 2208) and children exposed to OXC (N = 333) (Analysis 40.1). The RD also suggested no difference in the level of risk (RD ‐0.01, 95% CI −0.03 to 0.02; I^2^ = 0%).

The EURAP 2018 collaboration reported the prevalence of MCM was 2.9% (95% CI 2.3 to 3.7%) for children exposed to LTG and 3.0% (95% CI 1.4 to 5.4) for children exposed to OXC. No direct statistical comparison was made at the group level; investigations were made across different doses of the two ASMs (see Lamotrigine dose and Oxcarbazepine dose sections).

####### 40.1.2 Routine health record studies

Pooled results from three routine health record studies suggested no evidence of a difference in risk (RR 1.24, 95% CI 0.67 to 2.30; I^2^ = 0%), with no difference in the number of major malformations in children exposed to LTG (N = 2158) and children exposed to OXC (N = 377) (Analysis 40.1). The RD also suggested no difference in the level of risk (RD 0.01, 95% CI −0.01 to 0.03; I^2^ = 0%).

###### 40.2 Neural tube malformations

####### 40.2.1 Cohort studies

Pooled results from six cohort studies suggested no evidence of a difference in risk (RR 0.59, 95% CI 0.03 to 12.15; I^2^ = NA), with no difference in the number of neural tube malformations in children exposed to LTG (N = 2027) and children exposed to OXC (N = 319) (Analysis 40.2). The RD also suggested no difference in the level of risk (RD 0.00, 95% CI −0.01 to 0.01; I^2^ = 0%).

In the EURAP 2018 data, the prevalence of neural tube anomalies in those exposed to LTG was 0.3% (1/2514) and 0% for children exposed to OXC (0/333).

####### 40.2.2 Routine health record studies

No included studies reported data on this outcome.

###### 40.3 Cardiac malformations

####### 40.3.1 Cohort studies

Pooled results from eight cohort studies suggested no evidence of a difference in risk (RR 0.59, 95% CI 0.15 to 2.30; I^2^ = 0%), with no difference in the number of cardiac malformations in children exposed to LTG (N = 2084) and children exposed to OXC (N = 323) (Analysis 40.3). The RD also suggested no difference in the level of risk (RD 0.00, 95% CI −0.02 to 0.02; I^2^ = 0%).

In the EURAP 2018 data, the prevalence of cardiac anomalies in those exposed to LTG was 5.9% (15/2514) and 1.2% for children exposed to OXC (4/333).

####### 40.3.2 Routine health record studies

No included studies reported data on this outcome.

###### 40.4 Oro‐facial cleft/craniofacial malformations

####### 40.4.1 Cohort studies

Pooled results from six cohort studies suggested no evidence of a difference in risk (RR 0.64, 95% CI 0.12 to 3.46; I^2^ = 0%), with no difference in the number of oro‐facial cleft/craniofacial malformations in children exposed to LTG (N = 1997) and children exposed to OXC (N = 251) (Analysis 40.4). The RD also suggested no difference in the level of risk (RD ‐0.00, 95% CI −0.02 to 0.02; I^2^ = 0%).

In the EURAP 2018 data, the prevalence of cleft malformations (other oro‐facial not specifically reported) in those exposed to LTG was 0.11% (3/2514) and 0.30% for children exposed to OXC (1/333).

####### 40.4.2 Routine health record studies

No included studies reported data on this outcome.

###### 40.5 Skeletal/limb malformations

####### 40.5.1 Cohort studies

Pooled results from six cohort studies suggested no evidence of a difference in risk (RR 0.29, 95% CI 0.06 to 1.56; I^2^ = NA), with no difference in the number of skeletal/limb malformations in children exposed to LTG (N = 2027) and children exposed to OXC (N = 319) (Analysis 40.5). The RD also suggested no difference in the level of risk (RD ‐0.00, 95% CI −0.02 to 0.01; I^2^ = 0%).

####### 40.5.2 Routine health record studies

No included studies reported data on this outcome.

##### 41 LTG versus PB

###### 41.1 All major malformations

####### 41.1.1 Cohort studies

Pooled results from seven cohort studies suggested an increased risk with PB (RR 0.32, 95% CI 0.17 to 0.59; I^2^ = 0%), with children exposed to LTG (N = 2156) experiencing fewer major malformations than children exposed to PB (N = 421) (Analysis 41.1). The RD also suggested a higher risk for PB (RD −0.04, 95% CI −0.07 to −0.01; I^2^ = 0%).

The EURAP 2018 collaboration reported the prevalence of MCM was 2.9% (95% CI 2.3 to 3.7%) for children exposed to LTG and 6.5% (95% CI 4.2 to 9.9) for children exposed to PB. No direct statistical comparison was made at the group level; investigations were made across different doses of the two ASMs (see Lamotrigine dose and Phenobarbital dose sections).

####### 41.1.2 Routine health record studies

Pooled results from two routine health record studies suggested no evidence of a difference in risk (RR 0.41, 95% CI 0.13 to 1.28; I^2^ = 0%), with children exposed to LTG (N = 923) experiencing comparable major malformations to children exposed to PB (N = 34) (Analysis 41.1). The RD also suggested no difference in the level of risk (RD −0.05, 95% CI −0.15 to 0.04; I^2^ = 0%).

###### 41.2 Neural tube malformations

####### 41.2.1 Cohort studies

Pooled results from six cohort studies suggested no evidence of a difference in risk (RR 0.76, 95% CI 0.09 to 6.88; I^2^ = 0%), with no difference in the number of neural tube malformations in children exposed to LTG (N = 2009) and children exposed to PB (N = 412) (Analysis 41.2). The RD also suggested no difference in the level of risk (RD ‐0.00, 95% CI −0.01 to 0.01; I^2^ = 0%).

In the EURAP 2018 data, the prevalence of neural tube anomalies in those exposed to LTG was 0.3% (1/2514) and 0.68% for children exposed to PB (2/294).

####### 41.2.2 Routine health record studies

No included studies reported data on this outcome.

###### 41.3 Cardiac malformations

####### 41.3.1 Cohort studies

Pooled results from five cohort studies suggested an increased risk with PB (RR 0.21, 95% CI 0.08 to 0.56; I^2^ = 0%), with children exposed to LTG (N = 1990) experiencing fewer cardiac malformations than children exposed to PB (N = 411) (Analysis 41.3). The RD also suggested a higher risk for PB (RD ‐0.02, 95% CI −0.04 to ‐0.00; I^2^ = 0%).

In the EURAP 2018 data, the prevalence of cardiac anomalies in those exposed to LTG was 5.9% (15/2514) and 2.72% for children exposed to PB (8/294).

####### 41.3.2 Routine health record studies

No included studies reported data on this outcome.

###### 41.4 Oro‐facial cleft/craniofacial malformations

####### 41.4.1 Cohort studies

Pooled results from four cohort studies suggested an increased risk with PB (RR 0.22, 95% CI 0.07 to 0.68; I^2^ = 0%), with fewer oro‐facial cleft/craniofacial malformations in children exposed to LTG (N = 1940) compared to PB (N = 274) (Analysis 41.4). However, the RD suggested no difference in the level of risk (RD ‐0.01, 95% CI −0.03 to 0.01; I^2^ = 0%).

In the EURAP 2018 data, the prevalence of cleft malformations (other oro‐facial not specifically reported) in those exposed to LTG was 0.11% (3/2514) and 0.34% for children exposed to PB (1/294).

####### 41.4.2 Routine health record studies

No included studies reported data on this outcome.

###### 41.5 Skeletal/limb malformations

####### 41.5.1 Cohort studies

Pooled results from six cohort studies suggested no evidence of a difference in risk (RR 0.38, 95% CI 0.06 to 2.58; I^2^ = 0%), with no difference in the number of skeletal/limb malformations in children exposed to LTG (N = 2009) and children exposed to PB (N = 413) (Analysis 41.5). The RD also suggested no difference in the level of risk (RD ‐0.00, 95% CI −0.02 to 0.01; I^2^ = 0%).

####### 41.5.2 Routine health record studies

No included studies reported data on this outcome.

##### 42 LTG versus PHT

###### 42.1 All major malformations

####### 42.1.1 Cohort studies

Pooled results from six cohort studies suggested an increased risk with PHT (RR 0.55, 95% CI 0.35 to 0.87; I^2^ = 24%), with children exposed to LTG (N = 4251) experiencing fewer major malformations than children exposed to PHT (N = 742) (Analysis 42.1). The RD also suggested a higher risk for PHT (RD −0.02, 95% CI −0.03 to −0.00; I^2^ = 31%).

The EURAP 2018 collaboration reported the prevalence of MCM was 2.9% (95% CI 2.3 to 3.7%) for children exposed to LTG and 6.4% (95% CI 2.8 to 12.2) for children exposed to PHT. No direct statistical comparison was made at the group level; investigations were made across different doses of the two ASMs (see Lamotrigine dose and Phenytoin dose sections).

####### 42.1.2 Routine health record studies

One routine health record study suggested no evidence of a difference in risk (RR 0.65, 95% CI 0.20 to 2.16; I^2^ = NA%), with children exposed to LTG (N = 90) experiencing comparable major malformations to children exposed to PHT (N = 103) (Analysis 42.1). The RD also suggested no difference in the level of risk (RD −0.02, 95% CI −0.09 to 0.04; I^2^ = NA).

###### 42.2 Neural tube malformations

####### 43.2.1 Cohort studies

Pooled results from six cohort studies suggested no evidence of a difference in risk (RR 0.40, 95% CI 0.11 to 1.51; I^2^ = 0%), with no difference in the number of neural tube malformations in children exposed to LTG (N = 4127) and children exposed to PHT (N = 718) (Analysis 42.2). The RD also suggested no difference in the level of risk (RD ‐0.00, 95% CI −0.01 to 0.01; I^2^ = 0%).

In the EURAP 2018 data, the prevalence of neural tube anomalies in those exposed to LTG was 0.3% (1/2514) and 0.80% for children exposed to PHT (1/125).

####### 42.2.2 Routine health record studies

No included studies reported data on this outcome.

###### 42.3 Cardiac malformations

####### 42.3.1 Cohort studies

Pooled results from six cohort studies suggested an increased risk with PHT (RR 0.41, 95% CI 0.17 to 0.98; I^2^ = 0%), with children exposed to LTG (N = 4127) experiencing fewer cardiac malformations than children exposed to PHT (N = 718) (Analysis 42.3). However, the RD suggested no difference in the level of risk (RD ‐0.01, 95% CI −0.02 to 0.00; I^2^ = 0%).

In the EURAP 2018 data, the prevalence of cardiac anomalies in those exposed to LTG was 5.9% (15/2514) and 4.0% for children exposed to PHT (5/125).

####### 42.3.2 Routine health record studies

No included studies reported data on this outcome.

###### 42.4 Oro‐facial cleft/craniofacial malformations

####### 42.4.1 Cohort studies

Pooled results from five cohort studies suggested no evidence of a difference in risk (RR 0.73, 95% CI 0.23 to 2.28; I^2^ = 45%), with no difference in the number of oro‐facial cleft/craniofacial malformations in children exposed to LTG (N = 4077) and children exposed to PHT (N = 599) (Analysis 42.4). The RD also suggested no difference in the level of risk (RD ‐0.00, 95% CI −0.01 to 0.01; I^2^ = 0%).

In the EURAP 2018 data, the prevalence of cleft malformations (other oro‐facial not specifically reported) in those exposed to LTG was 0.11% (3/2514) and 0% for children exposed to PHT (0/125).

####### 42.4.2 Routine health record studies

No included studies reported data on this outcome.

###### 42.5 Skeletal/limb malformations

####### 42.5.1 Cohort studies

Pooled results from six cohort studies suggested an increased risk with PHT (RR 0.28, 95% CI 0.09 to 0.86; I^2^ = 0%), with children exposed to LTG (N = 4127) experiencing fewer skeletal/limb malformations than children exposed to PHT (N = 718) (Analysis 42.5). However, the RD suggested no difference in the level of risk (RD ‐0.01, 95% CI −0.01 to 0.00; I^2^ = 0%).

####### 42.5.2 Routine health record studies

No included studies reported data on this outcome.

##### 43 LTG versus PRM

###### 43.1 All major malformations

####### 43.1.1 Cohort studies

One cohort study suggested no evidence of a difference in risk (RR 0.30, 95% CI 0.02 to 3.93; I^2^ = NA), with children exposed to LTG (N = 406) experiencing comparable major malformations to children exposed to PRM (N = 2) (Analysis 43.1). The RD also suggested no difference in the level of risk (RD 0.05, 95% CI −0.37 to 0.47; I^2^ = NA).

####### 43.1.2 Routine health record studies

Results from one routine health record study suggested no evidence of a difference in risk (RR 0.40, 95% CI 0.03 to 6.16; I^2^ = NA), with children exposed to LTG (N = 90) experiencing comparable major malformations to children exposed to PRM (N = 2) (Analysis 43.1). The RD also suggested no difference in the level of risk (RD 0.04, 95% CI −0.28 to 0.37; I^2^ = NA).

###### 43.2 Neural tube malformations

####### 43.2.1 Cohort studies

No included studies reported data on this outcome.

####### 43.2.2 Routine health record studies

No included studies reported data on this outcome.

###### 43.3 Cardiac malformations

####### 43.3.1 Cohort studies

No included studies reported data on this outcome.

####### 43.3.2 Routine health record studies

No included studies reported data on this outcome.

###### 43.4 Oro‐facial cleft/craniofacial malformations

####### 43.4.1 Cohort studies

No included studies reported data on this outcome.

####### 43.4.2 Routine health record studies

No included studies reported data on this outcome.

###### 43.5 Skeletal/limb malformations

####### 43.5.1 Cohort studies

No included studies reported data on this outcome.

####### 43.5.2 Routine health record studies

No included studies reported data on this outcome.

##### 44 LTG versus TPM

###### 44.1 All major malformations

####### 44.1.1 Cohort studies

Pooled results from eight cohort studies suggested an increased risk with TPM (RR 0.59, 95% CI 0.36 to 0.96; I^2^ = 0%), with children exposed to LTG (N = 4275) experiencing fewer major malformations than children exposed to TPM (N = 505) (Analysis 44.1). However, the RD suggested no difference in the level of risk (RD −0.02, 95% CI −0.03 to 0.00; I^2^ = 0%).

The EURAP 2018 collaboration reported the prevalence of MCM was 2.9% (95% CI 2.3 to 3.7%) for children exposed to LTG and 3.9% (95% CI 1.5 to 8.4) for children exposed to TPM. No direct statistical comparison was made at the group level; investigations were made across different doses of the two ASMs (see Lamotrigine dose and Topiramate dose sections).

####### 44.1.2 Routine health record studies

Pooled results from two routine health record studies suggested no evidence of a difference in risk (RR 0.68, 95% CI 0.20 to 2.37; I^2^ = 0%), with children exposed to LTG (N = 923) experiencing comparable major malformation rates to children exposed to TPM (N = 49) (Analysis 44.1). The RD also suggested no difference in the level of risk (RD ‐0.01, 95% CI −0.07 to 0.06; I^2^ = 0%).

###### 44.2 Neural tube malformations

####### 44.2.1 Cohort studies

Pooled results from seven cohort studies suggested no evidence of a difference in risk (RR 0.62, 95% CI 0.08 to 4.94; I^2^ = 0%), with no difference in the number of neural tube malformations in children exposed to LTG (N = 4131) and children exposed to TPM (N = 496) (Analysis 44.2). The RD also suggested no difference in the level of risk (RD 0.00, 95% CI −0.01 to 0.01; I^2^ = 0%).

In the EURAP 2018 data, the prevalence of neural tube anomalies in those exposed to LTG was 0.3% (1/2514) and 0% for children exposed to TPM (0/152).

####### 44.2.2 Routine health record studies

No included studies reported data on this outcome.

###### 44.3 Cardiac malformations

####### 44.3.1 Cohort studies

Pooled results from eight cohort studies suggested no evidence of a difference in risk (RR 0.58, 95% CI 0.19 to 1.81; I^2^ = 0%), with no difference in the number of cardiac malformations in children exposed to LTG (N = 4151) and children exposed to TPM (N = 497) (Analysis 44.3). The RD also suggested no difference in the level of risk (RD ‐0.00, 95% CI −0.01 to 0.02; I^2^ = 0%).

In the EURAP 2018 data, the prevalence of cardiac anomalies in those exposed to LTG was 5.9% (15/2514) and 1.97% for children exposed to TPM (3/152).

####### 44.3.2 Routine health record studies

No included studies reported data on this outcome.

###### 44.4 Oro‐facial cleft/craniofacial malformations

####### 44.4.1 Cohort studies

Pooled results from seven cohort studies suggested evidence of a difference in risk (RR 0.31, 95% CI 0.13 to 0.74; I^2^ = 68%), with children exposed to LTG (N = 4101) experiencing less oro‐facial cleft/craniofacial malformations than children exposed to TPM (N = 488) (Analysis 44.4). Due to high heterogeneity, we undertook a random‐effects analysis, which found no difference (RR 0.22, 95% CI 0.03 to 1.48; I^2^ = 68%). The RD also suggested no difference in the level of risk (RD ‐0.01, 95% CI −0.02 to 0.00; I^2^ = 0%).

In the EURAP 2018 data, the prevalence of cleft malformations (other oro‐facial not specifically reported) in those exposed to LTG was 0.11% (3/2514) and 0% for children exposed to TPM (0/152).

####### 44.4.2 Routine health record studies

No included studies reported data on this outcome that could be included in the meta‐analysis. One study by Hernandez‐Diaz and colleagues using US Medicaid Registers could not be included in the meta‐analysis due to a lack of reporting of specific numbers of oral clefts. In this study, children born to women taking TPM had higher rates of oral clefts (N = 2425, 4.1 per 1000 live births) than the children born to women taking LTG (N = 2796, 1.5 per 1000 live births), but this was not reported to be statistically significant (RR 2.30, 95% CI 0.69 to 7.64).

###### 44.5 Skeletal/limb malformations

####### 44.5.1 Cohort studies

Pooled results from seven cohort studies suggested no evidence of a difference in risk (RR 0.17, 95% CI 0.06 to 0.52; I^2^ = 0%), with children exposed to LTG (N = 4131) experiencing fewer skeletal/limb malformations than children exposed to TPM (N = 496) (Analysis 44.5). The RD also suggested no difference in the level of risk (RD ‐0.01, 95% CI −0.02 to 0.00; I^2^ = 0%).

####### 44.5.2 Routine health record studies

No included studies reported data on this outcome.

##### 45 LTG versus ZNS

###### 45.1 All major malformations

####### 45.1.1 Cohort studies

Pooled results from four cohort studies suggested no evidence of a difference in risk (RR 0.66, 95% CI 0.26 to 1.65; I^2^ = 66%), with no difference in the number of major malformations in children exposed to LTG (N = 3792) and children exposed to ZNS (N = 130) (Analysis 45.1). Due to high heterogeneity, a random‐effects RR was calculated which also found no difference (RD 0.57, 95% CI 0.09 to 3.81, I^2^ = 66%). The RD also suggested no difference in the level of riskThe RD also suggested no difference in the level of risk (RD −0.01, 95% CI −0.04 to 0.02; I^2^ = 77%). Due to heterogeneity, a random‐effects RD was calculated, which upheld similar findings (RD ‐0.03, 95% CI ‐0.16 to 0.11, I^2^ = 77%).

####### 45.1.2 Routine health record studies

No included studies reported data on this outcome.

###### 45.2 Neural tube malformations

####### 45.2.1 Cohort studies

Pooled data from three included studies suggested evidence of a difference in risk (RR 0.02, 95% CI 0.00 to 0.26, I^2^ = NA), with children exposed to ZNS (N = 40) experiencing more neural tube malformations than children exposed to LTG (N = 2230) (Analysis 45.2). However, the RD suggested no difference in the level of risk (RD ‐0.03, 95% CI −0.09 to 0.04; I^2^ = 0%).

####### 45.2.2 Routine health record studies

No included studies reported data on this outcome.

###### 45.3 Cardiac malformations

####### 45.3.1 Cohort studies

Pooled data from two included studies suggested no evidence of a difference in risk (RR 0.30, 95% CI 0.04 to 2.52, I^2^ = 0%), with no difference in the number of cardiac malformations in children exposed to LTG (N = 2211) and children exposed to ZNS (N = 39) (Analysis 45.2). The RD also suggested no difference in the level of risk (RD 0.01, 95% CI −0.04 to 0.05; I^2^ = 0%).

####### 45.3.2 Routine health record studies

No included studies reported data on this outcome.

###### 45.4 Oro‐facial cleft/craniofacial malformations

####### 45.4.1 Cohort studies

Pooled data from two included studies suggested no evidence of a difference in risk (RR 0.06, 95% CI 0.00 to 1.31, I^2^ = NA), with no difference in the number of oro‐facial cleft/craniofacial malformations in children exposed to LTG (N = 2211) and children exposed to ZNS (N = 39) (Analysis 45.4). The RD also suggested no difference in the level of risk (RD 0.00, 95% CI −0.05 to 0.05; I^2^ = 0%).

####### 45.4.2 Routine health record studies

No included studies reported data on this outcome.

###### 45.5 Skeletal/limb malformations

####### 45.5.1 Cohort studies

Pooled data from three included studies suggested no evidence of a difference in risk (RR 0.22, 95% CI 0.03 to 1.93, I^2^ = 0%, with no difference in the number of skeletal/limb malformations in children exposed to LTG (N = 2230) and children exposed to ZNS (N = 40) (Analysis 45.5). The RD also suggested no difference in the level of risk (RD 0.00, 95% CI −0.05 to 0.06; I^2^ = 0%).

####### 45.5.2 Routine health record studies

No included studies reported data on this outcome.

##### 46 PHT versus GBP

###### 46.1 All major malformations

####### 46.1.1 Cohort studies

Pooled results from four cohort studies suggested no evidence of a difference in risk (RR 2.15, 95% CI 0.69 to 6.73; I^2^ = 0%), with no difference in the number of major malformations in children exposed to PHT (N = 567) and children exposed to GBP (N = 192) (Analysis 46.1). The RD also suggested no difference in the level of risk (RD 0.02, 95% CI ‐0.00 to 0.04; I^2^ = 0%).

####### 46.1.2 Routine health record studies

Results from one routine health record study suggested no evidence of a difference in risk (RR 2.74, 95% CI 0.16 to 46.00; I^2^ = 0%), with children exposed to PHT (N = 103) experiencing comparable major malformations to children exposed to GBP (N = 18) (Analysis 46.1). The RD also suggested no difference in the level of risk (RD 0.07, 95% CI ‐0.02 to 0.16; I^2^ = NA).

###### 46.2 Neural tube malformations

####### 46.2.1 Cohort studies

Included studies did not meet the threshold for reporting of the meta‐analysis (Analysis 46.2). However, available data showed there were 1/45 cases of neural tube malformations in children exposed to PHT and 0/16 cases in children exposed to GBP, based on data from two studies (Australian Epilepsy and Pregnancy Register; Miskov 2016).

####### 46.2.2 Routine health record studies

No included studies reported data on this outcome.

###### 46.3 Cardiac malformations

####### 46.3.1 Cohort studies

Included studies did not meet the threshold for reporting of the meta‐analysis (Analysis 46.3). However, available data showed there were 1/45 cases of cardiac malformations in children exposed to PHT and 1/16 cases in children exposed to GBP, based on data from two studies (Australian Epilepsy and Pregnancy Register; Miskov 2016).

####### 46.3.2 Routine health record studies

No included studies reported data on this outcome.

###### 46.4 Oro‐facial cleft/craniofacial malformations

####### 46.4.1 Cohort studies

We were unable to estimate a RR from two studies due to there being no reported oro‐facial cleft/craniofacial malformations in children exposed to PHT (N = 45) or GBP (N = 16) (Analysis 46.4).

####### 46.4.2 Routine health record studies

No included studies reported data on this outcome.

###### 46.5 Skeletal/limb malformations

####### 46.5.1 Cohort studies

We were unable to estimate a RR due to there being no reported cases of skeletal/limb malformations in children exposed to PHT (N = 45) or GBP (N = 16) (Analysis 46.5).

####### 46.5.2 Routine health record studies

No included studies reported data on this outcome.

##### 47 PHT versus OXC

###### 47.1 All major malformations

####### 47.1.1 Cohort studies

Pooled results from six cohort studies suggested no evidence of a difference in risk (RR 0.94, 95% CI 0.48 to 1.85; I^2^ = 0%), with no difference in the number of major malformations in children exposed to PHT (N = 706) and children exposed to OXC (N = 283) (Analysis 47.1). The RD also suggested no difference in the level of risk (RD 0.00, 95% CI −0.03 to 0.03; I^2^ = 0%).

The EURAP 2018 collaboration reported the prevalence of MCM was 6.4% (95% CI 2.8 to 12.2) for children exposed to PHT and 3.0% (95% CI 1.4 to 5.4) for children exposed to OXC. No direct statistical comparison was made at the group level; investigations were made across different doses of the two ASMs (see Phenytoin dose and Oxcarbazepine dose sections).

####### 47.1.2 Routine health record studies

Results from one routine health record study suggested an increased risk with PHT (RR 0.72, 95% CI 0.05 to 0.93; I^2^ = NA), with children exposed to PHT (N = 103) experiencing more major malformations than children exposed to OXC (N = 4) (Analysis 47.1). However, the RD suggested no difference in the level of risk (RD 0.07, 95% CI −0.20 to 0.34; I^2^ = NA).

###### 47.2 Neural tube malformations

####### 47.2.1 Cohort studies

Pooled results from four cohort studies suggested no evidence of a difference in risk (RR 1.16, 95% CI 0.13 to 10.29; I^2^ = 0%), with no difference in the number of neural tube malformations in children exposed to PHT (N = 703) and children exposed to OXC (N = 271) (Analysis 47.2). The RD also suggested no difference in the level of risk (RD 0.00, 95% CI −0.01 to 0.02; I^2^ = 0%).

In the EURAP 2018 data, the prevalence of neural tube anomalies in those exposed to PHT was 0.80% (1/125) and 0% for children exposed to OXC (0/333).

####### 47.2.2 Routine health record studies

No included studies reported data on this outcome.

###### 47.3 Cardiac malformations

####### 47.3.1 Cohort studies

Pooled results from five cohort studies suggested no evidence of a difference in risk (RR 1.33, 95% CI 0.43 to 14.17; I^2^ = 0%), with no difference in the number of cardiac malformations in children exposed to PHT (N = 704) and children exposed to OXC (N = 272) (Analysis 47.3). The RD also suggested no difference in the level of risk (RD 0.01, 95% CI −0.01 to 0.03; I^2^ = 0%).

In the EURAP 2018 data, the prevalence of cardiac anomalies in those exposed to PHT was 4.0% (5/125) and 1.2% for children exposed to OXC (4/333).

####### 47.3.2 Routine health record studies

No included studies reported data on this outcome.

###### 47.4 Oro‐facial cleft/craniofacial malformations

####### 47.4.1 Cohort studies

Pooled results from three cohort studies suggested no evidence of a difference in risk (RR 0.62, 95% CI 0.10 to 4.05; I^2^ = 0%), with no difference in the number of oro‐facial cleft/craniofacial malformations in children exposed to PHT (N = 584) and children exposed to OXC (N = 200) (Analysis 47.4). The RD also suggested no difference in the level of risk (RD ‐0.00, 95% CI −0.02 to 0.02; I^2^ = 0%).

In the EURAP 2018 data, the prevalence of cleft malformations (other oro‐facial not specifically reported) in those exposed to PHT was 0% (0/125) and 0.30% for children exposed to OXC (1/333).

####### 47.4.2 Routine health record studies

No included studies reported data on this outcome.

###### 47.5 Skeletal/limb malformations

####### 47.5.1 Cohort studies

Pooled results from four cohort studies suggested no evidence of a difference in risk (RR 1.20, 95% CI 0.23 to 6.35; I^2^ = 0%), with no difference in the number of skeletal/limb malformations in children exposed to PHT (N = 703) and children exposed to OXC (N = 271) (Analysis 47.5). The RD also suggested no difference in the level of risk (RD 0.00, 95% CI −0.01 to 0.02; I^2^ = 0%).

####### 47.5.2 Routine health record studies

No included studies reported data on this outcome.

##### 48 PHT versus PB

###### 48.1 All major malformations

####### 48.1.1 Cohort studies

Pooled results from 20 cohort studies suggested no evidence of a difference in risk (RR 0.84, 95% CI 0.57 to 1.23; I^2^ = 0%), with no difference in the number of major malformations in children exposed to PHT (N = 1095) and children exposed to PB (N = 634) (Analysis 48.1). The RD also suggested no difference in the level of risk (RD −0.01, 95% CI −0.03 to 0.02; I^2^ = 0%).

The EURAP 2018 collaboration reported the prevalence of MCM was 6.4% (95% CI 2.8 to 12.2) for children exposed to PHT and 6.5% (95% CI 4.2 to 9.9) for children exposed to PHT. No direct statistical comparison was made at the group level; investigations were made across different doses of the two ASMs (see Phenytoin dose and Phenobarbital dose sections). Samren 1997 reported nine cases of major malformation in 141 (6%) PHT cases and five cases in 48 (10%) PB‐exposed children.

####### 48.1.2 Routine health record studies

Results from one routine health record study suggested no evidence of a difference in risk (RR 0.48, 95% CI 0.07 to 3.35; I^2^ = NA), with children exposed to PHT (N = 103) experiencing comparable major malformations to children exposed to PB (N = 7) (Analysis 48.1). The RD also suggested no difference in the level of risk (RD −0.07, 95% CI −0.34 to 0.19; I^2^ = NA).

###### 48.2 Neural tube malformations

####### 48.2.1 Cohort studies

Pooled results from 11 studies suggested no evidence of a difference in risk (RR 0.79, 95% CI 0.10 to 5.94; I^2^ = 0%), with no difference in the number of neural tube malformations in children exposed to PHT (N = 707) and children exposed to PB (N = 475) (Analysis 48.2). The RD also suggested no difference in the level of risk (RD 0.00, 95% CI −0.01 to 0.01; I^2^ = 0%).

In the EURAP 2018 data, the prevalence of neural tube anomalies in those exposed to PHT was 0.80% (1/125) and 0.68% for children exposed to PB (2/294).

####### 48.2.2 Routine health record studies

No included studies reported data on this outcome.

###### 48.3 Cardiac malformations

####### 48.3.1 Cohort studies

Pooled results from 11 cohort studies suggested no evidence of a difference in risk (RR 0.56, 95% CI 0.29 to 1.07; I^2^ = 0%), with children exposed to PHT (N = 707) experiencing no more cardiac malformations than children exposed to PB (N = 475) (Analysis 48.3). The RD also suggested no difference in the level of risk (RD ‐0.02, 95% CI −0.04 to 0.01; I^2^ = 0%).

In the EURAP 2018 data, the prevalence of cardiac anomalies in those exposed to PHT was 4.0% (5/125) and 2.72% for children exposed to PB (8/294).

####### 48.3.2 Routine health record studies

No included studies reported data on this outcome.

###### 48.4 Oro‐facial cleft/craniofacial malformations

####### 48.4.1 Cohort studies

Pooled results from 11 cohort studies suggested no evidence of a difference in risk (RR 0.25, 95% CI 0.07 to 0.82; I^2^ = 0%), with children exposed to PHT (N = 593) experiencing fewer oro‐facial cleft/craniofacial malformations than children exposed to PB (N = 347) (Analysis 48.4). The RD also suggested no difference in the level of risk (RD ‐0.02, 95% CI −0.04 to 0.01; I^2^ = 0%).

In the EURAP 2018 data, the prevalence of cleft malformations (other oro‐facial not specifically reported) in those exposed to PHT was 0% (0/125) and 0.34% for children exposed to PB (1/294).

####### 48.4.2 Routine health record studies

No included studies reported data on this outcome.

###### 48.5 Skeletal/limb malformations

####### 48.5.1 Cohort studies

Pooled results from 11 cohort studies suggested no evidence of a difference in risk (RR 1.31, 95% CI 0.39 to 4.39; I^2^ = 0%), with no difference in the number of skeletal/limb malformations in children exposed to PHT (N = 707) and children exposed to PB (N = 475) (Analysis 48.5). The RD also suggested no difference in the level of risk (RD 0.00, 95% CI −0.01 to 0.02; I^2^ = 0%).

####### 48.5.2 Routine health record studies

No included studies reported data on this outcome.

##### 49 PHT versus PRM

###### 49.1 All major malformations

####### 49.1.1 Cohort studies

Pooled results from six cohort studies suggested no evidence of a difference in risk (RR 0.78, 95% CI 0.39 to 1.56; I^2^ = 19%), with no difference in the number of major malformations in children exposed to PHT (N = 360) and children exposed to PRM (N = 103) (Analysis 49.1). The RD also suggested no difference in the level of risk (RD −0.02, 95% CI −0.09 to 0.06; I^2^ = 0%).

####### 49.1.2 Routine health record studies

Results from one routine health record study suggested no evidence of a difference in risk (RR 0.58, 95% CI 0.04 to 8.44; I^2^ = NA), with children exposed to PHT (N = 103) experiencing no more major malformations than children exposed to PRM (N = 3) (Analysis 49.1). The RD also suggested no difference in the level of risk (RD 0.07, 95% CI −0.26 to 0.40; I^2^ = NA).

###### 49.2 Neural tube malformations

####### 49.2.1 Cohort studies

We were unable to estimate a RR from two studies due to there being no cases of neural tube malformations in children exposed to PHT (N = 36) or PRM (N = 39) (Analysis 49.2).

####### 49.2.2 Routine health record studies

No included studies reported data on this outcome.

###### 49.3 Cardiac malformations

####### 49.3.1 Cohort studies

Included studies did not meet the threshold for reporting of the meta‐analysis (Analysis 49.3). However, available data showed there were 0/36 cases of cardiac malformations in children exposed to PHT and 1/39 cases in children exposed to PRM, based on data from two studies (Milan Study 1999; Pardi 1982).

####### 49.3.2 Routine health record studies

No included studies reported data on this outcome.

###### 49.4 Oro‐facial cleft/craniofacial malformations

####### 49.4.1 Cohort studies

We were unable to estimate a RR due to there being no reported cases of oro‐facial cleft/craniofacial malformations in children exposed to PHT (N = 36) or PRM (N = 39) (Analysis 49.2).

####### 49.4.2 Routine health record studies

No included studies reported data on this outcome.

###### 49.5 Skeletal/limb malformations

####### 49.5.1 Cohort studies

Included studies did not meet the threshold for reporting of the meta‐analysis (Analysis 49.5). However, available data showed there were 1/36 cases of skeletal/limb malformations in children exposed to PHT and 0/39 cases in children exposed to PRM, based on data from two studies (Milan Study 1999; Pardi 1982).

####### 49.5.2 Routine health record studies

No included studies reported data on this outcome.

##### 50 PHT versus TPM

###### 50.1 All major malformations

####### 50.1.1 Cohort studies

Pooled results from four cohort studies showed no evidence of a difference in risk (RR 0.88, 95% CI 0.48 to 1.61; I^2^ = 0%), with no difference in the number of major malformations in children exposed to PHT (N = 685) and children exposed to TPM (N = 491) (Analysis 50.1). The RD also suggested no difference in the level of risk (RD −0.00, 95% CI −0.03 to 0.02; I^2^ = 0%).

The EURAP 2018 collaboration reported the prevalence of MCM was 2.9% (95% CI 2.3 to 3.7%) for children exposed to PHT and 3.9% (95% CI 1.5 to 8.4) for children exposed to TPM. No direct statistical comparison was made at the group level; investigations were made across different doses of the two ASMs (see Phenytoin dose and Topiramate dose sections).

####### 50.1.2 Routine health record studies

Results from one study suggested no evidence of a difference in risk (RR 0.29, 95% CI 0.02 to 3.51, I^2^ = NA), with no difference in the number of major malformations in children exposed to PHT (N = 103) and children exposed to PRM (N = 1) (Analysis 50.1). The RD also suggested no difference in the level of risk (RD 0.07, 95% CI ‐0.53 to 0.67; I^2^ = NA).

###### 50.2 Neural tube malformations

####### 50.2.1 Cohort studies

Pooled results from four cohort studies suggested no evidence of a difference in risk (RR 1.23, 95% CI 0.17 to 8.87; I^2^ = 24%), with no difference in the number of neural tube malformations in children exposed to PHT (N = 661) and children exposed to TPM (N = 483) (Analysis 50.2). The RD also suggested no difference in the level of risk (RD 0.00, 95% CI −0.01 to 0.01; I^2^ = 0%).

In the EURAP 2018 data, the prevalence of neural tube anomalies in those exposed to PHT was 0.80% (1/125) and 0% for children exposed to TPM (0/152).

####### 50.2.2 Routine health record studies

No included studies reported data on this outcome.

###### 50.3 Cardiac malformations

####### 50.3.1 Cohort studies

Pooled results from four cohort studies suggested no evidence of a difference in risk (RR 2.46, 95% CI 0.65 to 9.36; I^2^ = 0%), with no difference in the number of cardiac malformations in children exposed to PHT (N = 661) and children exposed to TPM (N = 483) (Analysis 50.3). The RD also suggested no difference in the level of risk (RD 0.01, 95% CI −0.00 to 0.02; I^2^ = 0%).

In the EURAP 2018 data, the prevalence of cardiac anomalies in those exposed to PHT was 4.0% (5/125) and 1.97% for children exposed to TPM (3/152).

####### 50.3.2 Routine health record studies

No included studies reported data on this outcome.

###### 50.4 Oro‐facial cleft/craniofacial malformations

####### 50.4.1 Cohort studies

Pooled results from three cohort studies suggested no evidence of a difference in risk (RR 0.37, 95% CI 0.10 to 1.42; I^2^ = 0%), with no difference in the number of oro‐facial cleft/craniofacial malformations in children exposed to PHT (N = 542) and children exposed to TPM (N = 474) (Analysis 50.4). The RD also suggested no difference in the level of risk(RD ‐0.01, 95% CI −0.02 to 0.00; I^2^ = 0%).

In the EURAP 2018 data, the prevalence of cleft malformations (other oro‐facial not specifically reported) in those exposed to PHT was 0% (0/125) and 0% for children exposed to TPM (0/152).

####### 50.4.2 Routine health record studies

No included studies reported data on this outcome.

###### 50.5 Skeletal/limb malformations

####### 50.5.1 Cohort studies

Pooled results from four cohort studies suggested no evidence of a difference in risk (RR 0.63, 95% CI 0.19 to 2.09; I^2^ = 0%), with no difference in the number of skeletal/limb malformations in children exposed to PHT (N = 661) and children exposed to TPM (N = 483) (Analysis 50.5). The RD also suggested no difference in the level of risk (RD ‐0.00, 95% CI −0.02 to 0.01; I^2^ = 0%).

####### 50.5.2 Routine health record studies

No included studies reported data on this outcome.

##### 51 PHT versus ZNS

###### 51.1 All major malformations

####### 51.1.1 Cohort studies

Pooled results from two cohort studies suggested no evidence of a difference in risk (RR 1.28, 95% CI 0.42 to 3.93; I^2^ = 61%), with no difference in the number of major malformations in children exposed to PHT (N = 522) and children exposed to ZNS (N = 116) (Analysis 51.1). Due to high heterogeneity, a random‐effects RR was calculated which found a similar effect (RR 1.28, 95% CI 0.42 to 3.93, I^2^ = 61%). The RD also suggested no difference in the level of risk (RD 0.01, 95% CI −0.02 to 0.05; I^2^ = 68%). Due to heterogeneity, a random‐effects RD was calculated which also found no difference in risk (RD 0.00, 95% CI ‐0.11 to 0.11, I^2^ = 68%)

####### 51.1.2 Routine health record studies

No included studies reported data on this outcome.

###### 51.2 Neural tube malformations

####### 51.2.1 Cohort studies

Results from one study suggested no evidence of a difference in risk (RR 0.11, 95% CI 0.00 to 2.58, I^2^ = NA), with no difference in the number of neural tube malformations in children exposed to PHT (N = 82) and children exposed to ZNS (N = 26) (Analysis 51.2). The RD also suggested no difference in the level of risk (RD ‐0.04, 95% CI −0.13 to 0.05; I^2^ = NA).

####### 51.2.2 Routine health record studies

No included studies reported data on this outcome.

###### 51.3 Cardiac malformations

####### 51.3.1 Cohort studies

Results from one study suggested no evidence of a difference in risk (RR 0.98, 95% CI 0.04 to 23.26, I^2^ = NA), with no difference in the number of cardiac malformations in children exposed to PHT (N = 82) and children exposed to ZNS (N = 26) (Analysis 51.3). The RD also suggested no difference in the level of risk (RD 0.01, 95% CI −0.05 to 0.07; I^2^ = NA).

####### 51.3.2 Routine health record studies

No included studies reported data on this outcome.

###### 51.4 Oro‐facial cleft/craniofacial malformations

####### 51.4.1 Cohort studies

Results from one study suggested no evidence of a difference in risk (RR 0.98, 95% CI 0.04 to 23.26, I^2^ = NA), with no difference in the number of oro‐facial cleft/craniofacial malformations in children exposed to PHT (N = 82) and children exposed to ZNS (N = 26) (Analysis 51.4). The RD also suggested no difference in the level of risk (RD 0.01, 95% CI −0.05 to 0.07; I^2^ = NA).

####### 51.4.2 Routine health record studies

No included studies reported data on this outcome.

###### 51.5 Skeletal/limb malformations

####### 51.5.1 Cohort studies

We were unable to estimate a RR from one study due to there being no reported skeletal/limb malformations in children exposed to PHT (N = 82) or ZNS (N = 26) (Analysis 51.5).

####### 51.5.2 Routine health record studies

No included studies reported data on this outcome.

##### 52 PB versus OXC

###### 52.1 All major malformations

####### 52.1.1 Cohort studies

Pooled results from eight cohort studies suggested no evidence of a difference in risk (RR 1.61, 95% CI 0.83 to 3.14; I^2^ = 19%), with no difference in the number of major malformations in children exposed to PB (N = 354) and children exposed to OXC (N = 322) (Analysis 52.1). The RD also suggested no difference in the level of risk (RD 0.02, 95% CI −0.02 to 0.06; I^2^ = 0%).

The EURAP 2018 collaboration reported the prevalence of MCM was 6.5% (95% CI 4.2 to 9.9) for children exposed to PB and 3.0% (95% CI 1.4 to 5.4) for children exposed to OXC. No direct statistical comparison was made at the group level; investigations were made across different doses of the two ASMs (see Phenobarbital dose and Oxcarbazepine dose sections).

####### 52.1.2 Routine health record studies

Pooled results from two routine health record studies suggested no evidence of a difference in risk (RR 3.07, 95% CI 0.50 to 18.92; I^2^ = 0%), with no difference in the number of major malformations in children exposed to PB (N = 34) and children exposed to OXC (N = 61) (Analysis 52.1). The RD also suggested no difference in the level of risk (RD 0.07, 95% CI −0.04 to 0.17; I^2^ = 0%).

###### 52.2 Neural tube malformations

####### 52.2.1 Cohort studies

Pooled results from six cohort studies suggested no evidence of a difference in risk (RR 1.57, 95% CI 0.06 to 37.94; I^2^ = NA), with no difference in the number of neural tube malformations in children exposed to PB (N = 349) and children exposed to OXC (N = 305) (Analysis 52.2). The RD also suggested no difference in the level of risk (RD 0.00, 95% CI −0.01 to 0.02; I^2^ = 0%).

In the EURAP 2018 data, the prevalence of neural tube anomalies in those exposed to PB was 0.68% (2/294) and 0% for children exposed to OXC (0/333).

####### 52.2.2 Routine health record studies

No included studies reported data on this outcome.

###### 52.3 Cardiac malformations

####### 52.3.1 Cohort studies

Pooled results from seven cohort studies suggested no evidence of a difference in risk (RR 2.58, 95% CI 0.94 to 7.09; I^2^ = 51%), with children exposed to PB (N = 352) experiencing comparable cardiac malformations to children exposed to OXC (N = 306) (Analysis 52.3). Due to high heterogeneity, a random‐effects RR was calculated which also found no difference (RR 3.84, 95% CI 0.54 to 27.19, I^2^ = 51%). The RD also suggested no difference in the level of risk (RD 0.02, 95% CI −0.01 to 0.05; I^2^ = 0%).

In the EURAP 2018 data, the prevalence of cardiac anomalies in those exposed to PB was 2.72% (8/294) and 1.20% for children exposed to OXC (4/333).

####### 52.3.2 Routine health record studies

No included studies reported data on this outcome.

###### 52.4 Oro‐facial cleft/craniofacial malformations

####### 52.4.1 Cohort studies

Pooled results from five cohort studies suggested no evidence of a difference in risk (RR 3.66, 95% CI 0.41 to 32.43; I^2^ = NA), with no difference in the number of oro‐facial cleft/craniofacial malformations in children exposed to PB (N = 212) and children exposed to TPM (N = 234) (Analysis 52.4). The RD also suggested no difference in the level of risk (RD 0.01, 95% CI −0.01 to 0.04; I^2^ = 0%).

In the EURAP 2018 data, the prevalence of cleft malformations (other oro‐facial not specifically reported) in those exposed to PB was 0.34% (1/294) and 0.30% for children exposed to OXC (1/333).

####### 52.4.2 Routine health record studies

No included studies reported data on this outcome.

###### 52.5 Skeletal/limb malformations

####### 52.5.1 Cohort studies

Pooled results from six cohort studies suggested no evidence of a difference in risk (RR 0.98, 95% CI 0.16 to 5.97; I^2^ = 0%), with no difference in the number of skeletal/limb malformations in children exposed to PB (N = 349) and children exposed to OXC (N = 305) (Analysis 52.5). The RD also suggested no difference in the level of risk (RD ‐0.00, 95% CI −0.02 to 0.02; I^2^ = 0%).

####### 52.5.2 Routine health record studies

No included studies reported data on this outcome.

##### 53 PB versus PRM

###### 53.1 All major malformations

####### 53.1.1 Cohort studies

Pooled results from six cohort studies suggested no evidence of a difference in risk (RR 0.50, 95% CI 0.21 to 1.16; I^2^ = 0%), with no difference in the number of major malformations in children exposed to PB (N = 241) and children exposed to PRM (N = 110) (Analysis 53.1). The RD also suggested no difference in the level of risk (RD −0.05, 95% CI −0.12 to 0.02; I^2^ = 0%).

####### 53.1.2 Routine health record studies

Included studies did not meet the threshold for reporting of the meta‐analysis (Analysis 53.1). However, available data showed there were 1/7 cases of major malformations in children exposed to PB and 0/3 cases in children exposed to PRM, based on data from one study (Sweden Health Record Registers).

###### 53.2 Neural tube malformations

####### 53.2.1 Cohort studies

We were unable to estimate a RR from two studies due to there being no reported no cases of neural tube malformations in children exposed to PB (N = 95) or PRM (N = 39) (Analysis 53.2).

####### 53.2.2 Routine health record studies

No included studies reported data on this outcome.

###### 53.3 Cardiac malformations

####### 53.3.1 Cohort studies

Pooled results from two studies suggested no evidence of a difference in risk (RR 0.42, 95% CI 0.03 to 6.55, I^2^ = NA), with no difference in the number of cardiac malformations in children exposed to PB (N = 95) and children exposed to PRM (N = 39) (Analysis 53.1). The RD also suggested no difference in the level of risk (RD ‐0.01, 95% CI −0.08 to 0.05; I^2^ = 0%).

####### 53.3.2 Routine health record studies

No included studies reported data on this outcome.

###### 53.4 Oro‐facial cleft/craniofacial malformations

####### 53.4.1 Cohort studies

We were unable to estimate a RR from two studies due to there being no reported cases of oro‐facial cleft/craniofacial malformations in children exposed to PB (N = 95) or PRM (N = 39) (Analysis 53.4).

####### 53.4.2 Routine health record studies

No included studies reported data on this outcome.

###### 53.5 Skeletal/limb malformations

####### 53.5.1 Cohort studies

Pooled results from two studies suggested no evidence of a difference in risk (RR 1.29, 95% CI 0.05 to 30.82, I^2^ = NA), with no difference in the number of skeletal/limb malformations in children exposed to PB (N = 95) and children exposed to PRM (N = 39) (Analysis 53.5). The RD also suggested no difference in the level of risk (RD 0.01, 95% CI −0.05 to 0.07; I^2^ = 0%).

####### 53.5.2 Routine health record studies

No included studies reported data on this outcome.

##### 54 PB versus TPM

###### 54.1 All major malformations

####### 54.1.1 Cohort studies

Pooled results from four cohort studies suggested no evidence of a difference in risk (RR 1.38, 95% CI 0.68 to 2.81; I^2^ = 0%), with no difference in the number of major malformations in children exposed to PB (N = 340) and children exposed to TPM (N = 426) (Analysis 54.1). The RD also suggested no difference in the level of risk (RD 0.02, 95% CI −0.02 to 0.05; I^2^ = 0%).

The EURAP 2018 collaboration reported the prevalence of MCM was 6.5% (95% CI 4.2 to 9.9) for children exposed to PB and 3.9% (95% CI 1.5 to 8.4) for children exposed to TPM. No direct statistical comparison was made at the group level; investigations were made across different doses of the two ASMs (see Phenobarbital dose and Topiramate dose sections).

####### 54.1.2 Routine health record studies

Included studies did not meet the threshold for reporting of the meta‐analysis (Analysis 54.1). However, available data showed there were 3/34 cases of major malformations in children exposed to PB and 2/49 cases in children exposed to TPM, based on data from two studies (Norwegian Health Record Registers; Sweden Health Record Registers).

###### 54.2 Neural tube malformations

####### 54.2.1 Cohort studies

Pooled results from four cohort studies suggested no evidence of a difference in risk (RR 0.22, 95% CI 0.01 to 5.00; I^2^ = NA), with no difference in the number of neural tube malformations in children exposed to PB (N = 343) and children exposed to TPM (N = 417) (Analysis 54.2). The RD also suggested no difference in the level of risk (RD 0.00, 95% CI −0.02 to 0.02; I^2^ = 0%).

In the EURAP 2018 data, the prevalence of neural tube anomalies in those exposed to PB was 0.68% (2/294) and 0% for children exposed to TPM (0/152).

####### 54.2.2 Routine health record studies

No included studies reported data on this outcome.

###### 54.3 Cardiac malformations

####### 54.3.1 Cohort studies

Pooled results from four cohort studies suggested an increased risk with PB (RR 4.44, 95% CI 0.98 to 20.12; I^2^ = 37%), with children exposed to PB (N = 343) experiencing more cardiac malformations than children exposed to TPM (N = 417) (Analysis 54.3). However, the RD suggested no difference in the level of risk (RD 0.02, 95% CI −0.00 to 0.05; I^2^ = 0%).

In the EURAP 2018 data, the prevalence of cardiac anomalies in those exposed to PB was 2.72% (8/294) and 1.97% for children exposed to TPM (3/152).

####### 54.3.2 Routine health record studies

No included studies reported data on this outcome.

###### 54.4 Oro‐facial cleft/craniofacial malformations

####### 54.4.1 Cohort studies

Pooled results from three cohort studies suggested no evidence of a difference in risk (RR 1.44, 95% CI 0.39 to 5.31; I^2^ = NA), with no difference in the number of oro‐facial cleft/craniofacial malformations in children exposed to PB (N = 206) and children exposed to TPM (N = 408) (Analysis 54.4). The RD also suggested no difference in the level of risk (RD 0.01, 95% CI −0.02 to 0.03; I^2^ = 0%).

In the EURAP 2018 data, the prevalence of cleft malformations (other oro‐facial not specifically reported) in those exposed to PB was 0.34% (1/294) and 0% for children exposed to TPM (0/152).

####### 54.4.2 Routine health record studies

No included studies reported data on this outcome.

###### 54.5 Skeletal/limb malformations

####### 54.5.1 Cohort studies

Pooled results from four cohort studies suggested no evidence of a difference in risk (RR 0.36, 95% CI 0.06 to 2.19; I^2^ = 0%), with no difference in the number of skeletal/limb malformations in children exposed to PB (N = 343) and children exposed to TPM (N = 417) (Analysis 54.5). The RD also suggested no difference in the level of risk (RD ‐0.01, 95% CI −0.03 to 0.01; I^2^ = 0%).

####### 54.5.2 Routine health record studies

No included studies reported data on this outcome.

##### 55 PB versus ZNS

###### 55.1 All major malformations

####### 55.1.1 Cohort studies

Pooled results from two cohort studies suggested no evidence of a difference in risk (RR 10.46, 95% CI 0.62 to 175.67; I^2^ = NA), with no difference in the number of major malformations in children exposed to PB (N = 201) and children exposed to ZNS (N = 91) (Analysis 55.1). The RD suggested a higher rate of major malformation observed in the PB‐exposed group (RD 0.05, 95% CI 0.02 to 0.09; I^2^ = 0%).

####### 55.1.2 Routine health record studies

No included studies reported data on this outcome.

###### 55.2 Neural tube malformations

####### 55.2.1 Cohort studies

We were unable to estimate a RR from one study due to there being no cases of neural tube malformations in children exposed to PB (N = 2) or ZNS (N = 1) (Analysis 55.2).

####### 55.2.2 Routine health record studies

No included studies reported data on this outcome.

###### 55.3 Cardiac malformations

####### 55.3.1 Cohort studies

We were unable to estimate a RR from one study due to there being no cases of cardiac malformations in children exposed to PB (N = 2) or ZNS (N = 1) (Analysis 55.3).

####### 55.3.2 Routine health record studies

No included studies reported data on this outcome.

###### 55.4 Oro‐facial cleft/craniofacial malformations

####### 55.4.1 Cohort studies

We were unable to estimate a RR from one study due to there being no cases of oro‐facial cleft /craniofacial malformations PB (N = 2) or ZNS (N = 1) (Analysis 55.4).

####### 55.4.2 Routine health record studies

No included studies reported data on this outcome.

###### 55.5 Skeletal/limb malformations

####### 55.5.1 Cohort studies

We were unable to estimate a RR from one study due to there being no cases of skeletal/limb malformations in children exposed to PB (N = 2) or ZNS (N = 1) (Analysis 55.5).

####### 55.5.2 Routine health record studies

No included studies reported data on this outcome.

##### 56 TPM versus ZNS

###### 56.1 All major malformations

####### 56.1.1 Cohort studies

Pooled results from four cohort studies suggested no evidence of a difference in risk (RR 1.59, 95% CI 0.54 to 4.66; I^2^ = 58%), with no difference in the number of major malformations in children exposed to TPM (N = 440) and children exposed to ZNS (N = 130) (Analysis 56.1). Due to high heterogeneity, a random‐effects RR was calculated which also found no difference (RD 1.44, 95% CI 0.19 to 10.82, I^2^ =58%). The RD also suggested no difference in the level of risk (RD 0.02, 95% CI ‐0.02 to 0.06; I^2^ = 35%).

####### 56.1.2 Routine health record studies

No included studies reported data on this outcome.

###### 56.2 Neural tube malformations

####### 56.2.1 Cohort studies

We were unable to estimate a RR from two studies due to there being no cases of neural tube malformations in children exposed to TPM (N = 11) or ZNS (N = 14) (Analysis 56.2).

####### 56.2.2 Routine health record studies

No included studies reported data on this outcome.

###### 56.3 Cardiac malformations

####### 56.3.1 Cohort studies

Pooled results from two studies suggested no evidence of a difference in risk (RR 6.00 95% CI 0.28 to 129.16, I^2^ = NA), with no difference in the number of cardiac malformations in children exposed to TPM (N = 81) and children exposed to ZNS (N = 40) (Analysis 56.3). The RD also suggested no difference in the level of risk (RD 0.03, 95% CI −0.06 to 0.12; I^2^ = 0%).

####### 56.3.2 Routine health record studies

No included studies reported data on this outcome.

###### 56.4 Oro‐facial cleft/craniofacial malformations

####### 56.4.1 Cohort studies

Pooled results from two studies suggested no evidence of a difference in risk (RR 1.90 95% CI 0.09 to 38.34, I^2^ = NA), with no difference in the number of oro‐facial cleft/craniofacial malformations in children exposed to TPM (N = 81) and children exposed to ZNS (N = 40) (Analysis 56.4). The RD also suggested no difference in the level of risk (RD 0.02, 95% CI −0.06 to 0.10; I^2^ = 0%).

####### 56.4.2 Routine health record studies

No included studies reported data on this outcome.

###### 56.5 Skeletal/limb malformations

####### 56.5.1 Cohort studies

We were unable to estimate a RR from three studies due to there being no cases of skeletal/limb malformations in children exposed to TPM (N = 81) or ZNS (N = 40) (Analysis 56.5).

####### 56.5.2 Routine health record studies

No included studies reported data on this outcome.

##### 57 TPM vs LAC

###### 57.1 All major malformations

####### 57.1.1 Cohort studies

We were unable to estimate a RR from one cohort study due to there being no major malformations observed in children exposed to TPM (N = 5) or LAC (N = 1) (Analysis 57.1).

####### 57.1.2 Routine health record studies

No included studies reported data on this outcome.

###### 57.2 Neural tube malformations

####### 57.2.1 Cohort studies

We were unable to estimate a RR from one study due to there being no cases of neural tube malformations in children exposed to TPM (N = 5) or LAC (N = 1) (Analysis 57.2).

####### 57.2.2 Routine health record studies

No included studies reported data on this outcome.

###### 57.3 Cardiac malformations

####### 57.3.1 Cohort studies

We were unable to estimate a RR from one study due to there being no cases of cardiac malformations in children exposed to TPM (N = 5) or LAC (N = 1) (Analysis 57.3).

####### 57.3.2 Routine health record studies

No included studies reported data on this outcome.

###### 57.4 Oro‐facial cleft/craniofacial malformations

####### 57.4.1 Cohort studies

We were unable to estimate a RR from one study due to there being no cases of oro‐facial cleft/ craniofacial malformations in children exposed to TPM (N = 5) or LAC (N = 1) (Analysis 57.4).

####### 57.4.2 Routine health record studies

No included studies reported data on this outcome.

###### 57.5 Skeletal/limb malformations

####### 57.5.1 Cohort studies

We were unable to estimate a RR from one study due to there being no cases of skeletal/limb malformations in children exposed to TPM (N = 5) or LAC (N = 1) (Analysis 57.5).

####### 57.5.2 Routine health record studies

No included studies reported data on this outcome.

##### 58 VPA versus GBP

###### 58.1 All major malformations

####### 58.1.1 Cohort studies

Pooled results from four cohort studies suggested an increased risk with VPA (RR 4.27, 95% CI 1.60 to 11.35; I^2^ = 58%), with children exposed to VPA (N = 1839) experiencing more major malformations than children exposed to GBP (N = 192) (Analysis 58.1). Due to high heterogeneity, a random‐effects RR was calculated which found no difference in the level of risk (RD 2.43, 95% CI 0.40 to 14.64, I^2^ = 58%). However, both the fixed‐effect RD analysis (RD 0.08, 95% CI 0.04 to 0.11; I^2^ = 60%) and a random‐effects RD also suggested a higher absolute risk for VPA (RD 0.08, 95% CI 0.01 to 0.14, I^2^ = 60%).

####### 58.1.2 Routine health record studies

Results from one study suggested no evidence of a difference in risk (RR 3.74, 95% CI 0.24 to 59.08, I^2^ = NA), with no difference in the number of major malformations in children exposed to VPA (N = 268) and children exposed to GBP (N = 18) (Analysis 58.1). However, the RD suggested a difference in the level of risk (RD 0.10, 95% CI 0.02 to 0.18; I^2^ = NA).

###### 58.2 Neural tube malformations

####### 58.2.1 Cohort studies

Pooled results from two studies suggested no evidence of a difference in risk (RR 0.83, 95% CI 0.05 to 13.81, I^2^ = NA), with no difference in the number of neural tube malformations in children exposed to VPA (N = 277) and children exposed to GBP (N = 18) (Analysis 58.2). The RD also suggested no difference in the level of risk (RD 0.02, 95% CI ‐0.09 to 0.13; I^2^ = 0%).

####### 58.2.2 Routine health record studies

No included studies reported data on this outcome.

###### 58.3 Cardiac malformations

####### 58.3.1 Cohort studies

Pooled results from two studies suggested no evidence of a difference in risk (RR 0.46, 95% CI 0.08 to 2.70, I^2^ = 4%), with no difference in the number of cardiac malformations in children exposed to VPA (N = 277) and children exposed to GBP (N = 16) (Analysis 58.3). The RD also suggested no difference in the level of risk (RD ‐0.02, 95% CI ‐0.14 to 0.11; I^2^ = 74%).

####### 58.3.2 Routine health record studies

No included studies reported data on this outcome.

###### 58.4 Oro‐facial cleft/craniofacial malformations

####### 58.4.1 Cohort studies

Pooled results from two studies suggested no evidence of a difference in risk (RR 1.38, 95% CI 0.09 to 22.19, I^2^ = NA), with no difference in the number of oro‐facial cleft/craniofacial malformations in children exposed to VPA (N = 277) and children exposed to GBP (N = 16) (Analysis 58.4). The RD also suggested no difference in the level of risk (RD 0.04, 95% CI ‐0.07 to 0.15; I^2^ = 0%).

####### 58.4.2 Routine health record studies

No included studies reported data on this outcome.

###### 58.5 Skeletal/limb malformations

####### 58.5.1 Cohort studies

Pooled results from two studies suggested no evidence of a difference in risk (RR 0.72, 95% CI 0.04 to 12.14, I^2^ = NA), with no difference in the number of skeletal/limb malformations in children exposed to VPA (N = 277) and children exposed to GBP (N = 16)(Analysis 58.5). The RD also suggested no difference in the level of risk (RD 0.02, 95% CI ‐0.09 to 0.13; I^2^ = 0%).

####### 58.5.2 Routine health record studies

No included studies reported data on this outcome.

##### 59 VPA vs LAC

###### 59.1 All major malformations

####### 59.1.1 Cohort studies

Included studies did not meet the threshold for reporting of the meta‐analysis (Analysis 59.1). However, available data showed there were 4/17 cases of major malformations in children exposed to VPA and 0/1 cases in children exposed to LAC, based on data from one study (Jimenez 2020).

####### 59.1.2 Routine health record studies

No included studies reported data on this outcome.

###### 59.2 Neural tube malformations

####### 59.2.1 Cohort studies

Included studies did not meet the threshold for reporting of the meta‐analysis (Analysis 59.3). However, available data showed there were 1/17 cases of neural tube malformations in children exposed to VPA and 0/1 cases in children exposed to LAC, based on data from one study (Jimenez 2020).

####### 59.2.2 Routine health record studies

No included studies reported data on this outcome.

###### 59.3 Cardiac malformations

####### 59.3.1 Cohort studies

Included studies did not meet the threshold for reporting of the meta‐analysis (Analysis 59.4). However, available data showed there were 1/17 cases of cardiac malformations in children exposed to VPA and 0/1 cases in children exposed to LAC, based on data from one study (Jimenez 2020).

####### 59.3.2 Routine health record studies

No included studies reported data on this outcome.

###### 59.4 Oro‐facial cleft/craniofacial malformations

####### 59.4.1 Cohort studies

Included studies did not meet the threshold for reporting of the meta‐analysis (Analysis 59.5). However, available data showed there were 1/17 cases of oro‐facial cleft/craniofacial malformations in children exposed to VPA and 0/1 cases in children exposed to LAC, based on data from one study (Jimenez 2020).

####### 59.4.2 Routine health record studies

No included studies reported data on this outcome.

###### 59.5 Skeletal/limb malformations

####### 59.5.1 Cohort studies

Included studies did not meet the threshold for reporting of the meta‐analysis (Analysis 59.2). However, available data showed that there were 0/17 cases of skeletal/limb malformations in children exposed to VPA (N = 17) and 0/1 in children exposed to LAC, based on data from one study (Jimenez 2020).

####### 59.5.2 Routine health record studies

No included studies reported data on this outcome.

##### 60 VPA versus LEV

###### 60.1 All major malformations

####### 60.1.1 Cohort studies

Pooled results from 10 cohort studies suggested an increased risk with VPA (RR 3.77, 95% CI 2.48 to 5.74; I^2^ = 17%), with children exposed to VPA (N = 2342) experiencing more major malformations than children exposed to LEV (N = 1143) (Analysis 60.1). The RD also suggested a higher absolute risk for VPA (RD 0.07, 95% CI 0.05 to 0.08; I^2^ = 11%).

The EURAP 2018 collaboration reported the prevalence of MCM was 10.3% (95% CI 8.8 to 12.0) for children exposed to VPA and 2.8% (95% CI 1.7 to 4.5) for children exposed to LEV. No direct statistical comparison was made at the group level; investigations were made across different doses of the two ASMs (see Valproate dose and Levetiracetam dose sections).

####### 60.1.2 Routine health record studies

Pooled results from two routine health record studies suggested an increased risk with VPA (RR 3.26, 95% CI 1.51 to 7.03; I^2^ = 0%), with children exposed to VPA (N = 663) experiencing more major malformations than children exposed to LEV (N = 248) (Analysis 60.1). The RD also suggested a higher risk for VPA (RD 0.06, 95% CI 0.03 to 0.09; I^2^ = 28%).

###### 60.2 Neural tube malformations

####### 60.2.1 Cohort studies

Pooled results from nine cohort studies suggested an increased risk with VPA (RR 3.76, 95% CI 1.22 to 11.55; I^2^ = 0%), with children exposed to VPA (N = 2298) experiencing more neural tube malformations than children exposed to LEV (N = 1048) (Analysis 60.2). The RD also suggested a higher risk for VPA (RD 0.01, 95% CI 0.00 to 0.02; I^2^ = 0%).

In the EURAP 2018 data, the prevalence of neural tube anomalies in those exposed to VPA was 1.15% (16/1381) and 0% for children exposed to LEV (0/599).

####### 60.2.2 Routine health record studies

No included studies reported data on this outcome.

###### 60.3 Cardiac malformations

####### 60.3.1 Cohort studies

Pooled results from 10 cohort studies suggested an increased risk with VPA (RR 3.04, 95% CI 1.46 to 6.34; I^2^ = 0%), with children exposed to VPA (N = 2299) experiencing more cardiac malformations than children exposed to LEV (N = 1057) (Analysis 60.3). The RD also suggested a higher risk for VPA (RD 0.02, 95% CI 0.01 to 0.03; I^2^ = 0%).

In the EURAP 2018 data, the prevalence of cardiac anomalies in those exposed to VPA was 2.46% (34/1381) and 0.83% for children exposed to LEV 0.83% (5/599).

####### 60.3.2 Routine health record studies

No included studies reported data on this outcome.

###### 60.4 Oro‐facial cleft/craniofacial malformations

####### 60.4.1 Cohort studies

Pooled results from nine studies suggested an increased risk with VPA (RR 3.75, 95% CI 1.19 to 11.77; I^2^ = 0%), with children exposed to VPA (N = 1958) experiencing more oro‐facial cleft/craniofacial malformations than children exposed to LEV (N = 951) (Analysis 60.4). The RD also suggested a higher risk for VPA (RD 0.01, 95% CI 0.00 to 0.02; I^2^ = 0%).

In the EURAP 2018 data, the prevalence of cleft malformations (other oro‐facial not specifically reported) in those exposed to VPA was 0.43% (6/1381) and 0.16% for children exposed to LEV (1/599).

####### 60.4.2 Routine health record studies

No included studies reported data on this outcome.

###### 60.5 Skeletal/limb malformations

####### 60.5.1 Cohort studies

Pooled results from nine cohort studies suggested an increased risk with VPA (RR 2.41, 95% CI 0.99 to 5.85; I^2^ = 55%), with children exposed to VPA (N = 2298) experiencing more skeletal/limb malformations than children exposed to LEV (N = 1048) (Analysis 60.5). Due to high heterogeneity, a random‐effects RR was calculated which found no difference in the level of risk (RR 1.89, 95% CI 0.34 to 10.60, I^2^ = 55%). However, both the fixed‐effect RD analysis (RD 0.01, 95% CI 0.00 to 0.02; I^2^ = 12%) and the random‐effects RD analysis (RD 0.01, 95% CI 0.00 to 0.02; I^2^ = 12%) also suggested a higher absolute risk for VPA.

####### 60.5.2 Routine health record studies

No included studies reported data on this outcome.

##### 61 VPA versus LTG

###### 61.1 All major malformations

####### 61.1.1 Cohort studies

Pooled results from 12 cohort studies suggested an increased risk with VPA (RR 3.50, 95% CI 2.76 to 4.46; I^2^ = 0%), with children exposed to VPA (N = 2459) experiencing more major malformations than children exposed to LTG (N = 4437) (Analysis 61.1). The RD suggested an increased risk for VPA (RD 0.06, 95% CI 0.05 to 0.08; I^2^ = 34%).

The EURAP 2018 collaboration reported the prevalence of MCM was 10.3% (95% CI 8.8 to 12.0) for children exposed to VPA and 2.9% (95% CI 2.3 to 3.7) for children exposed to LTG. No direct statistical comparison was made at the group level; investigations were made across different doses of the two ASMs (see Valproate dose and Lamotrigine dose sections).

####### 61.1.2 Routine health record studies

Pooled results from four routine health record studies suggested an increased risk with VPA (RR 2.49, 95% CI 1.86 to 3.35; I^2^ = 0%), with children exposed to VPA (N = 1088) experiencing more major malformations than children exposed to LTG (N = 2502) (Analysis 61.1). The RD also suggested an increased level of risk for VPA (RD 0.05, 95% CI 0.03 to 0.07; I^2^ = 42%).

###### 61.2 Neural tube malformations

####### 61.2.1 Cohort studies

Pooled results from 11 cohort studies suggested an increased risk with VPA (RR 7.48, 95% CI 3.27 to 17.13; I^2^ = 0%), with children exposed to VPA (N = 2415) experiencing more neural tube malformations than children exposed to LTG (N = 4293) (Analysis 61.2). The RD also suggested an increased level of risk for VPA (RD 0.01, 95% CI 0.01 to 0.02; I^2^ = 0%).

In the EURAP 2018 data, the prevalence of neural tube anomalies in those exposed to VPA was 1.15% (16/1381) and 0.3% for children exposed to LTG (1/2514).

####### 61.2.2 Routine health record studies

No included studies reported data on this outcome.

###### 61.3 Cardiac malformations

####### 61.3.1 Cohort studies

Pooled results from 12 cohort studies suggested an increased risk with VPA (RR 3.39, 95% CI 2.06 to 5.60; I^2^ = 0%), with children exposed to VPA (N = 2416) experiencing more neural tube malformations than children exposed to LTG (N = 4313) (Analysis 61.3). The RD also suggested an increased level of risk for VPA (RD 0.02, 95% CI 0.01 to 0.02; I^2^ = 3%).

In the EURAP 2018 data, the prevalence of cardiac anomalies in those exposed to VPA was 2.46% (34/1381) and 5.9% for children exposed to LTG (15/2514).

####### 61.3.2 Routine health record studies

No included studies reported data on this outcome.

###### 61.4 Oro‐facial cleft/craniofacial malformations

####### 61.4.1 Cohort studies

Pooled results from 11 cohort studies suggested an increased risk with VPA (RR 4.16, 95% CI 2.14 to 8.08; I^2^ = 0%), with children exposed to VPA (N = 2075) experiencing more craniofacial malformations than children exposed to LTG (N = 4263) (Analysis 61.4). The RD also suggested an increased level of risk for VPA (RD 0.01, 95% CI 0.01 to 0.02; I^2^ = 0%).

In the EURAP 2018 data, the prevalence of cleft malformations (other oro‐facial not specifically reported) in those exposed to VPA was 0.43% (6/1381) and 0.11% for children exposed to LTG (3/2514).

####### 61.4.2 Routine health record studies

No included studies reported data on this outcome.

###### 61.5 Skeletal/limb malformations

####### 61.5.1 Cohort studies

Pooled results from 11 cohort studies suggested an increased risk with VPA (RR 6.09, 95% CI 2.91 to 12.76; I^2^ = 0%), with children exposed to VPA (N = 2415) experiencing more craniofacial malformations than children exposed to LTG (N = 4293) (Analysis 61.5). The RD also suggested an increased level of risk for VPA (RD 0.01, 95% CI 0.01 to 0.02; I^2^ = 26%).

####### 61.5.2 Routine health record studies

No included studies reported data on this outcome.

##### 62 VPA versus TPM

###### 62.1 All major malformations

####### 62.1.1 Cohort studies

Pooled results from seven cohort studies suggested an increased risk with VPA (RR 2.47, 95% CI 1.50 to 4.08; I^2^ = 0%), with children exposed to VPA (N = 2219) experiencing more major malformations than children exposed to TPM (N = 504) (Analysis 62.1). The RD also suggested a higher absolute risk for VPA (RD 0.06, 95% CI 0.03 to 0.09; I^2^ = 41%). Due to high heterogeneity, we undertook a random‐effects analysis which found a similar effect (RD 0.07, 95% CI 0.02 to 0.11; I^2^ = 41%).

The EURAP 2018 collaboration reported the prevalence of MCM was 10.3% (95% CI 8.8 to 12.0) for children exposed to VPA and 3.9% (95% CI 1.5 to 8.4) for children exposed to TPM. No direct statistical comparison was made at the group level; investigations were made across different doses of the two ASMs (see Valproate dose and Topiramate dose sections).

####### 62.1.2 Routine health record studies

Pooled results from two studies suggested no evidence of a difference in risk (RR 1.27, 95% CI 0.36 to 4.39, I^2^ = 0%), with no difference in the number of major malformations in children exposed to VPA (N = 601) and children exposed to TPM (N = 49) (Analysis 62.1). The RD also suggested no difference in the level of risk (RD 1.27, 95% CI 0.36 to 4.39; I^2^ = 0%).

###### 62.2 Neural tube malformations

####### 62.2.1 Cohort studies

Pooled results from six cohort studies suggested no evidence of a difference in risk (RR 2.39, 95% CI 0.73 to 7.80; I^2^ = 2%), with no difference in the number of neural tube malformations in children exposed to VPA (N = 2175) and children exposed to TPM (N = 490) (Analysis 62.2). The RD suggested a higher risk for VPA (RD 0.01, 95% CI 0.00 to 0.03; I^2^ = 0%).

In the EURAP 2018 data, the prevalence of neural tube anomalies in those exposed to VPA was 1.15% (16/1381) and 0% for children exposed to TPM (0/152).

####### 62.2.2 Routine health record studies

No included studies reported data on this outcome.

###### 62.3 Cardiac malformations

####### 62.3.1 Cohort studies

Pooled results from six cohort studies suggested an increased risk with VPA (RR 3.48, 95% CI 1.16 to 10.48; I^2^ = 0%), with children exposed to VPA (N = 2175) experiencing more cardiac malformations than children exposed to TPM (N = 495) (Analysis 62.3). The RD also suggested a higher absolute risk for VPA (RD 0.02, 95% CI 0.01 to 0.04; I^2^ = 0%).

In the EURAP 2018 data, the prevalence of cardiac anomalies in those exposed to VPA was 2.46% (34/1381) and 1.97% for children exposed to TPM (3/152).

####### 62.3.2 Routine health record studies

No included studies reported data on this outcome.

###### 62.4 Oro‐facial cleft/craniofacial malformations

####### 62.4.1 Cohort studies

Pooled results from six cohort studies suggested no evidence of a difference in risk (RR 0.89 95% CI 0.37 to 2.13; I^2^ = 0%), with no difference in the number of oro‐facial cleft/craniofacial malformations in children exposed to VPA (N = 1835) and children exposed to TPM (N = 482) (Analysis 62.4). The RD also suggested no difference in the level of risk (RD 0.00, 95% CI ‐0.02 to 0.02; I^2^ = 0%).

In the EURAP 2018 data, the prevalence of cleft malformations (other oro‐facial not specifically reported) in those exposed to VPA was 0.43% (6/1381) and 0% for children exposed to TPM (0/152).

####### 62.4.2 Routine health record studies

No included studies reported data on this outcome.

###### 62.5 Skeletal/limb malformations

####### 62.5.1 Cohort studies

Pooled results from six cohort studies suggested no evidence of a difference in risk (RR 1.45, 95% CI 0.55 to 3.82; I^2^ = 0%), with no difference in the number of skeletal/limb malformations in children exposed to VPA (N = 2199) and children exposed to TPM (N = 490) (Analysis 62.5). The RD also suggested no difference in the level of risk (RD 0.01, 95% CI ‐0.00 to 0.02; I^2^ = 0%).

####### 62.5.2 Routine health record studies

No included studies reported data on this outcome.

##### 63 VPA versus OXC

###### 63.1 All major malformations

####### 63.1.1 Cohort studies

Pooled results from 11 cohort studies suggested an increased risk with VPA (RR 2.48, 95% CI 1.42 to 4.31; I^2^ = 13%), with children exposed to VPA (N = 1183) experiencing more major malformations than children exposed to OXC (N = 378) (Analysis 63.1). The RD also suggested a higher risk for VPA (RD 0.06, 95% CI 0.03 to 0.09; I^2^ = 0%).

The EURAP 2018 collaboration reported the prevalence of MCM was 10.3% (95% CI 8.8 to 12.0) for children exposed to VPA and 3.0% (95% CI 1.4 to 5.4) for children exposed to OXC. No direct statistical comparison was made at the group level; investigations were made across different doses of the two ASMs (see Valproate dose and Oxcarbazepine dose sections).

####### 63.1.2 Routine health record studies

Results from four routine health record studies suggested an increased risk with VPA (RR 1.60, 95% CI 1.11 to 2.29; I^2^ = 80%), with children exposed to VPA (N = 1194) experiencing more major malformations than children exposed to OXC (N = 507) (Analysis 63.1). Due to heterogeneity, a random‐effects RR was calculated, which showed no evidence of a difference in risk (RR 1.80, 95% CI 0.57 to 5.67; I^2^ = 80%). The RD suggested a higher risk for VPA (RD 0.04, 95% CI 0.01 to 0.08; I^2^ = 65%). Due to heterogeneity, a random‐effects RD was calculated which showed no evidence of a difference in risk (RD 0.04, 95% CI ‐0.01 to 0.10, I^2^ = 65%).

####### 63.2.1 Cohort studies

Pooled results from nine cohort studies suggested no evidence of a difference in risk (RR 1.55, 95% CI 0.49 to 4.89; I^2^ = 0%), with no difference in the number of neural tube malformations in children exposed to VPA (N = 1133) and children exposed to OXC (N = 364) (Analysis 63.2). The RD also suggested no difference in the level of risk (RD 0.01, 95% CI ‐0.00 to 0.03; I^2^ = 0%).

In the EURAP 2018 data, the prevalence of neural tube anomalies in those exposed to VPA was 1.15% (16/1381) and 0% for children exposed to OXC (0/333).

####### 63.2.2 Routine health record studies

No included studies reported data on this outcome.

###### 63.3 Cardiac malformations

####### 63.3.1 Cohort studies

Pooled results from 11 cohort studies suggested no evidence of a difference in risk (RR 1.80, 95% CI 0.84 to 3.88; I^2^ = 0%), with no difference in the number of cardiac malformations in children exposed to VPA (N = 1140) and children exposed to OXC (N = 457) (Analysis 63.3). The RD suggested a higher risk for VPA (RD 0.02, 95% CI 0.00 to 0.04; I^2^ = 0%).

In the EURAP 2018 data, the prevalence of cardiac anomalies in those exposed to VPA was 2.46% (34/1381) and 1.20% for children exposed to OXC (4/333).

####### 63.3.2 Routine health record studies

No included studies reported data on this outcome.

###### 63.4 Oro‐facial cleft/craniofacial malformations

####### 63.4.1 Cohort studies

Pooled results from nine cohort studies suggested no evidence of a difference in risk (RR 2.14, 95% CI 0.76 to 6.06; I^2^ = 0%), with no difference in the number of oro‐facial cleft/craniofacial malformations in children exposed to VPA (N = 793) and children exposed to OXC (N = 385) (Analysis 63.4). The RD also suggested no difference in the level of risk (RD 0.02, 95% CI ‐0.00 to 0.04; I^2^ = 0%).

In the EURAP 2018 data, the prevalence of cleft malformations (other oro‐facial not specifically reported) in those exposed to VPA was 0.43% (6/1381) and 0.30% for children exposed to OXC (1/333).

####### 63.4.2 Routine health record studies

No included studies reported data on this outcome.

###### 63.5 Skeletal/limb malformations

####### 63.5.1 Cohort studies

Pooled results from nine cohort studies suggested no evidence of a difference in risk (RR 1.37, 95% CI 0.42 to 4.49; I^2^ = 0%), with no difference in the number of skeletal/limb malformations in children exposed to VPA (N = 1133) and children exposed to OXC (N = 364) (Analysis 63.5). The RD also suggested no difference in the level of risk (RD 0.01, 95% CI ‐0.01 to 0.02; I^2^ = 0%).

####### 63.5.2 Routine health record studies

No included studies reported data on this outcome.

##### 64 VPA versus PB

###### 64.1 All major malformations

####### 64.1.1 Cohort studies

Pooled results from 23 cohort studies suggested an increased risk with VPA (RR 1.49, 95% CI 1.08 to 2.07; I^2^ = 0%), with children exposed to VPA (N = 1557) experiencing more major malformations than children exposed to PB (N = 759) (Analysis 64.1). The RD also suggested a higher risk for VPA (RD 0.04, 95% CI 0.01 to 0.06; I^2^ = 0%).

The EURAP 2018 collaboration reported the prevalence of MCM was 10.3% (95% CI 8.8 to 12.0) for children exposed to VPA and 6.5% (95% CI 4.2 to 9.9) for children exposed to PB. No direct statistical comparison was made at the group level; investigations were made across different doses of the two ASMs (see Valproate dose and Phenobarbital dose sections). Samren 1997 reported six cases of major malformation out of 184 (9%) VPA‐exposed children and five cases from 48 (10%) PB‐exposed children.

####### 64.1.2 Routine health record studies

Pooled results from two studies suggested no evidence of a difference in risk (RR 0.79, 95% CI 0.26 to 2.42, I^2^ = 0%), with no difference in the number of major malformations in children exposed to VPA (N = 601) and children exposed to PRM (N = 34) (Analysis 64.1). The RD also suggested no difference in the level of risk (RD ‐0.02, 95% CI −0.12 to 0.08; I^2^ = 0%).

###### 64.2 Neural tube malformations

####### 64.2.1 Cohort studies

Pooled results from 14 cohort studies suggested evidence of a difference in risk (RR 3.04, 95% CI 1.27 to 7.30; I^2^ = 1%), with children exposed to VPA (N = 1174) experiencing more neural tube malformations to children exposed to PB (N = 546) (Analysis 64.2). The RD also suggested a difference in the level of risk (RD 0.02, 95% CI 0.01 to 0.04; I^2^ = 7%).

In the EURAP 2018 data, the prevalence of neural tube anomalies in those exposed to VPA was 1.15% (16/1381) and 0.68% for children exposed to PB (2/294).

####### 64.2.2 Routine health record studies

No included studies reported data on this outcome.

###### 64.3 Cardiac malformations

####### 64.3.1 Cohort studies

Pooled results from 14 cohort studies suggested no evidence of a difference in risk (RR 0.84, 95% CI 0.50 to 1.43; I^2^ = 0%), with no difference in the number of cardiac malformations in children exposed to VPA (N = 1174) and children exposed to PB (N = 546) (Analysis 64.3). The RD also suggested no difference in the level of risk (RD −0.00, 95% CI −0.03 to 0.02; I^2^ = 0%).

In the EURAP 2018 data, the prevalence of cardiac anomalies in those exposed to VPA was 2.46% (34/1381) and 2.72% for children exposed to PB (8/294).

####### 64.3.2 Routine health record studies

No included studies reported data on this outcome.

###### 64.4 Oro‐facial cleft/craniofacial malformations

####### 64.4.1 Cohort studies

Pooled results from 14 cohort studies suggested no evidence of a difference in risk (RR 0.54, 95% CI 0.23 to 1.27; I^2^ = 0%), with no difference in the number of oro‐facial cleft/craniofacial malformations in children exposed to VPA (N = 839) and children exposed to PB (N = 418) (Analysis 64.4). The RD also suggested no difference in the level of risk (RD −0.01, 95% CI −0.03 to 0.02; I^2^ = 0%).

In the EURAP 2018 data, the prevalence of cleft malformations (other oro‐facial not specifically reported) in those exposed to VPA was 0.43% (6/1381) and 0.34% for children exposed to PB (1/294).

####### 64.4.2 Routine health record studies

No included studies reported data on this outcome.

###### 64.5 Skeletal/limb malformations

####### 64.5.1 Cohort studies

Pooled results from 14 cohort studies suggested no evidence of a difference in risk (RR 1.62, 95% CI 0.70 to 3.74; I^2^ = 0%), with no difference in the number of skeletal/limb malformations in children exposed to VPA (N = 1174) and children exposed to PB (N = 546) (Analysis 64.5). The RD also suggested no difference in the level of risk (RD 0.01, 95% CI ‐0.01 to 0.03; I^2^ = 0%).

####### 64.5.2 Routine health record studies

No included studies reported data on this outcome.

##### 65 VPA versus PHT

###### 65.1 All major malformations

####### 65.1.1 Cohort studies

Pooled results from 21 cohort studies suggested an increased risk with VPA (RR 1.92, 95% CI 1.44 to 2.56; I^2^ = 0%), with children exposed to VPA (N = 2650) experiencing more major malformations than children exposed to PHT (N = 1247) (Analysis 65.1). The RD also suggested a higher risk for VPA (RD 0.05, 95% CI 0.03 to 0.07; I^2^ = 4%).

The EURAP 2018 collaboration reported the prevalence of MCM was 10.3% (95% CI 8.8 to 12.0) for children exposed to VPA and 6.4% (95% CI 2.8 to 12.2) for children exposed to PHT. No direct statistical comparison was made at the group level; investigations were made across different doses of the two ASMs (see Valproate dose and Phenytoin dose sections). Samren 1997 reported six cases of major malformation in 184 (9%) children exposed to VPA and nine in 141 (6%) PHT‐exposed children.

####### 65.1.2 Routine health record studies

Results from one routine health record study suggested no evidence of a difference in risk (RR 1.43, 95% CI 0.64 to 3.19; I^2^ = 0%), with no difference in the number of major malformations in children exposed to VPA (N = 268) and children exposed to PHT (N = 103) (Analysis 65.1). The RD also suggested no difference in the level of risk (RD 0.03, 95% CI −0.03 to 0.09; I^2^ = NA).

###### 65.2 Neural tube malformations

####### 65.2.1 Cohort studies

Pooled results from 14 cohort studies suggested an increased risk with VPA (RR 3.75, 95% CI 1.57 to 8.94; I^2^ = 0%), with children exposed to VPA (N = 2419) experiencing more neural tube malformations than children exposed to PHT (N = 974) (Analysis 65.2). The RD also suggested a higher risk for VPA (RD 0.02, 95% CI 0.01 to 0.03; I^2^ = 2%).

In the EURAP 2018 data, the prevalence of neural tube anomalies in those exposed to VPA was 1.15% (16/1381) and 0.80% for children exposed to PHT (1/125).

####### 65.2.2 Routine health record studies

No included studies reported data on this outcome.

###### 65.3 Cardiac malformations

####### 65.3.1 Cohort studies

Pooled results from 14 cohort studies suggested an increased risk with VPA (RR 1.90, 95% CI 1.07 to 3.36; I^2^ = 0%), with children exposed to VPA (N = 2419) experiencing more cardiac malformations than children exposed to PHT (N = 974) (Analysis 65.3). The RD also suggested a higher risk for VPA (RD 0.02, 95% CI 0.00 to 0.03; I^2^ = 0%).

In the EURAP 2018 data, the prevalence of cardiac anomalies in those exposed to VPA was 2.46% (34/1381) and 4.0% for children exposed to PHT (5/125).

####### 65.3.2 Routine health record studies

No included studies reported data on this outcome.

###### 65.4 Oro‐facial cleft/craniofacial malformations

####### 65.4.1 Cohort studies

Pooled results from 14 cohort studies suggested no evidence of a difference in risk (RR 2.24, 95% CI 0.89 to 5.58; I^2^ = 0%), with no difference in the number of oro‐facial cleft/craniofacial malformations in children exposed to VPA (N = 2084) and children exposed to PHT (N = 860) (Analysis 65.4). The RD also suggested no difference in the level of risk (RD 0.01, 95% CI ‐0.00 to 0.02; I^2^ = 0%).

In the EURAP 2018 data, the prevalence of cleft malformations (other oro‐facial not specifically reported) in those exposed to VPA was 0.43% (6/1381) and 0% for children exposed to PHT (0/125).

####### 65.4.2 Routine health record studies

No included studies reported data on this outcome.

###### 65.5 Skeletal/limb malformations

####### 65.5.1 Cohort studies

Pooled results from 14 cohort studies suggested an increased risk with VPA (RR 2.12, 95% CI 1.01 to 4.45; I^2^ = 0%), with children exposed to VPA (N = 2419) experiencing more skeletal/limb malformations than children exposed to PHT (N = 975) (Analysis 65.5). The RD also suggested a higher risk for VPA (RD 0.01, 95% CI 0.00 to 0.02; I^2^ = 0%).

####### 65.5.2 Routine health record studies

No included studies reported data on this outcome.

##### 66 VPA versus ZNS

###### 66.1 All major malformations

####### 66.1.1 Cohort studies

Pooled results from three cohort studies suggested no evidence of a difference in risk (RR 2.34, 95% CI 0.95 to 5.80; I^2^ = 77%), with children exposed to VPA (N = 1560) experiencing comparable major malformations to children exposed to ZNS (N = 117) (Analysis 66.1). Due to high heterogeneity, a random‐effects RR analysis was completed which also found no difference (RR 1.81, 95% CI 0.14 to 22.75; I^2^ = 77%). The RD suggested a higher risk for VPA (RD 0.06, 95% CI 0.01 to 0.10; I^2^ = 72%). However, due to heterogeneity, a random‐effects RD was calculated which found no difference in the level of risk (RD 0.04, 95% CI ‐0.11 to 0.19, I^2^ = 72%).

####### 66.1.2 Routine health record studies

No included studies reported data on this outcome.

###### 66.2 Neural tube malformations

####### 66.2.1 Cohort studies

Pooled results from two studies suggested no evidence of a difference in risk (RR 0.29, 95% CI 0.06 to 1.51, I^2^ = 0%), with no difference in the number of neural tube malformations in children exposed to VPA (N = 1237) and children exposed to ZNS (N = 27) (Analysis 66.2). The RD also suggested no difference in the level of risk (RD ‐0.02, 95% CI −0.11 to 0.06; I^2^ = 0%).

####### 66.2.2 Routine health record studies

No included studies reported data on this outcome.

###### 66.3 Cardiac malformations

####### 66.3.1 Cohort studies

Pooled results from two studies suggested no evidence of a difference in risk (RR 0.49, 95% CI 0.07 to 3.65, I^2^ = 0%), with no difference in the number of cardiac malformations in children exposed to VPA (N = 1237) and children exposed to ZNS (N = 27) (Analysis 66.3). The RD also suggested no difference in the level of risk (RD 0.01, 95% CI −0.05 to 0.08; I^2^ = 0%).

####### 66.3.2 Routine health record studies

No included studies reported data on this outcome.

###### 66.4 Oro‐facial cleft/craniofacial malformations

####### 66.4.1 Cohort studies

Pooled results from two studies suggested no evidence of a difference in risk (RR 0.47, 95% CI 0.06 to 3.49, I^2^ = 0%), with no difference in the number of oro‐facial cleft/craniofacial malformations in children exposed to VPA (N = 1237) and children exposed to ZNS (N = 27) (Analysis 66.4). The RD also suggested no difference in the level of risk (RD 0.01, 95% CI −0.05 to 0.08; I^2^ = 0%).

####### 66.4.2 Routine health record studies

No included studies reported data on this outcome.

###### 66.5 Skeletal/limb malformations

####### 66.5.1 Cohort studies

Pooled results from two studies suggested no evidence of a difference in risk (RR 0.46, 95% CI 0.03 to 7.72, I^2^ = NA), with no difference in the number of skeletal/limb malformations in children exposed to VPA (N = 1237) and children exposed to ZNS (N = 27) (Analysis 66.5). The RD also suggested no difference in the level of risk (RD 0.01, 95% CI −0.06 to 0.07; I^2^ = 0%).

####### 66.5.2 Routine health record studies

No included studies reported data on this outcome.

##### 67 CZP vs VPA

###### 67.1 All major malformations

####### 67.1.1 Cohort studies

Pooled results from four cohort studies suggested an increased risk with VPA (RR 0.29, 95% CI 0.09 to 0.90; I^2^ = 0%), with children exposed to VPA (N = 955) experiencing more major malformations than children exposed to CZP (N = 95) (Analysis 67.1). The RD also suggested a higher risk for VPA (RD −0.09, 95% CI −0.13 to ‐0.04; I^2^ = 30%).

####### 67.1.2 Routine health record studies

Pooled results from two routine health record studies suggested an increased risk with VPA (RR 0.34, 95% CI 0.13 to 0.94; I^2^ = 0%), with children exposed to VPA (N = 601) experiencing more major malformations than children exposed to CZP (N = 161) (Analysis 67.1). The RD suggested no difference in the level of risk (RD −0.05, 95% CI −0.12 to 0.01; I^2^ = 0%).

###### 67.2 Neural tube malformations

####### 67.2.1 Cohort studies

Results from one study suggested no evidence of a difference in risk (RR 9.77, 95% CI 0.58 to 165.35, I^2^ = NA), with no difference in the number of neural tube malformations in children exposed to CZP (N = 4) and children exposed to VPA (N = 341) (Analysis 67.2). The RD also suggested no difference in the level of risk (RD ‐0.01, 95% CI −0.27 to 0.25; I^2^ = NA).

####### 67.2.2 Routine health record studies

No included studies reported data on this outcome.

###### 67.3 Cardiac malformations

####### 67.3.1 Cohort studies

Results from one study suggested no evidence of a difference in risk (RR 1.67, 95% CI 0.12 to 23.92, I^2^ = NA), with no difference in the number of cardiac malformations in children exposed to CZP (N = 4) and children exposed to VPA (N = 341) (Analysis 67.3). The RD also suggested no difference in the level of risk (RD ‐0.06, 95% CI −0.32 to 0.21; I^2^ = NA).

####### 67.3.2 Routine health record studies

No included studies reported data on this outcome.

###### 67.4 Skeletal/limb malformations

####### 67.4.1 Cohort studies

Results from one study suggested no evidence of a difference in risk (RR 7.60, 95% CI 0.47 to 123.14, I^2^ = NA), with no difference in the number of skeletal/limb malformations in children exposed to CZP (N = 4) and children exposed to VPA (N = 341) (Analysis 67.4). The RD also suggested no difference in the level of risk (RD ‐0.01, 95% CI −0.27 to 0.25; I^2^ = NA).

####### 67.4.2 Routine health record studies

No included studies reported data on this outcome.

###### 67.5 Oro‐facial cleft/craniofacial malformations

####### 67.5.1 Cohort studies

No included studies reported data on this outcome.

####### 67.5.2 Routine health record studies

No included studies reported data on this outcome.

##### 68 CZP versus LEV

###### 68.1 All major malformations

####### 68.1.1 Cohort studies

Pooled results from three cohort studies suggested no evidence of a difference in risk (RR 1.06, 95% CI 0.32 to 3.44; I^2^ = 0%), with children exposed to CZP (N = 94) experiencing more major malformations than children exposed to LEV (N = 695) (Analysis 68.1). The RD also suggested no difference in the level of risk (RD −0.01, 95% CI −0.05 to 0.03; I^2^ = 0%).

####### 68.1.2 Routine health record studies

Results from one routine health record study suggested no evidence of a difference in risk (RR 1.04, 95% CI 0.15 to 7.29; I^2^ = NA), with no difference in the number of major malformations in children exposed to CZP (N = 113) and children exposed to LEV (N = 118) (Analysis 68.1). The RD also suggested no difference in the level of risk (RD 0.00, 95% CI −0.03 to 0.03; I^2^ = 0%).

###### 68.2 Neural tube malformations

####### 68.2.1 Cohort studies

No included studies reported data on this outcome.

####### 68.2.2 Routine health record studies

No included studies reported data on this outcome.

###### 68.3 Cardiac malformations

####### 68.3.1 Cohort studies

No included studies reported data on this outcome.

####### 68.3.2 Routine health record studies

No included studies reported data on this outcome.

###### 68.4 Oro‐facial cleft/craniofacial malformations

####### 68.4.1 Cohort studies

No included studies reported data on this outcome.

####### 68.4.2 Routine health record studies

No included studies reported data on this outcome.

###### 68.5 Skeletal/limb malformations

####### 68.5.1 Cohort studies

No included studies reported data on this outcome.

####### 68.5.2 Routine health record studies

No included studies reported data on this outcome.

##### 69 OXC versus PRM

###### 69.1 All major malformations

####### 69.1.1 Cohort studies

Pooled results from two cohort studies suggested no evidence of a difference in risk (RR 0.58, 95% CI 0.08 to 4.03; I^2^ = 0%), with no difference in the number of major malformations in children exposed to OXC (N = 28) and children exposed to PRM (N = 8) (Analysis 69.1). The RD also suggested no difference in the level of risk (RD −0.02, 95% CI −0.34 to 0.30; I^2^ = 0%).

####### 69.1.2 Routine health record studies

We were unable to estimate the RR for one routine health record study due to there being no reported major malformations observed in children exposed to OXC (N = 4) or PRM (N = 3) (Analysis 69.1).

###### 69.2 Neural tube malformations

####### 69.2.1 Cohort studies

No included studies reported data on this outcome.

####### 69.2.2 Routine health record studies

No included studies reported data on this outcome.

###### 69.3 Cardiac malformations

####### 69.3.1 Cohort studies

No included studies reported data on this outcome.

####### 69.3.2 Routine health record studies

No included studies reported data on this outcome.

###### 69.4 Oro‐facial cleft/craniofacial malformations

####### 69.4.1 Cohort studies

No included studies reported data on this outcome.

####### 69.4.2 Routine health record studies

No included studies reported data on this outcome.

###### 69.5 Skeletal/limb malformations

####### 69.5.1 Cohort studies

No included studies reported data on this outcome.

####### 69.5.2 Routine health record studies

No included studies reported data on this outcome.

##### 70 OXC versus TPM

###### 70.1 All major malformations

####### 70.1.1 Cohort studies

Pooled results from five cohort studies suggested no evidence of a difference in risk (RR 0.71, 95% CI 0.28 to 1.77; I^2^ = 0%), with no difference in the number of major malformations in children exposed to OXC (N = 279) and children exposed to TPM (N = 427) (Analysis 70.1). The RD also suggested no difference in the level of risk (RD −0.01, 95% CI −0.04 to 0.02; I^2^ = 0%).

The EURAP 2018 collaboration reported the prevalence of MCM was 3.0% (95% CI 1.4 to 5.4) for children exposed to OXC and 3.9% (95% CI 1.5 to 8.4) for children exposed to TPM. No direct statistical comparison was made at the group level; investigations were made across different doses of the two ASMs (see oxcarbazepine dose and topiramate dose sections).

####### 70.1.2 Routine health record studies

Included studies did not meet the threshold for reporting of the meta‐analysis (Analysis 70.1). However, available data showed there were 1/61 cases of major malformations in children exposed to OXC and 2/49 cases in children exposed to TPM, based on data from two studies (Norwegian Health Record Registers; Sweden Health Record Registers).

###### 70.2 Neural tube malformations

####### 70.2.1 Cohort studies

We could not estimate a RR from four cohort studies due to there being no reported neural tube malformations in children exposed to OXC (N = 266) or children exposed to TPM (N = 418) (Analysis 70.2).

In the EURAP 2018 data, the prevalence of neural tube anomalies in those exposed to OXC was 0% (0/333) and 0% for children exposed to TPM (0/152).

####### 70.2.2 Routine health record studies

No included studies reported data on this outcome.

###### 70.3 Cardiac malformations

####### 70.3.1 Cohort studies

Pooled results from five cohort studies suggested no evidence of a difference in risk (RR 0.80, 95% CI 0.09 to 6.81; I^2^ = 0%), with no difference in the number of cardiac malformations in children exposed to OXC (N = 269) and children exposed to TPM (N = 419) (Analysis 70.3). The RD also suggested no difference in the level of risk (RD 0.00, 95% CI ‐0.02 to 0.02; I^2^ = 0%).

In the EURAP 2018 data, the prevalence of cardiac anomalies in those exposed to OXC was 1.20% (4/333) and 1.97% for children exposed to TPM (3/152).

####### 70.3.2 Routine health record studies

No included studies reported data on this outcome.

###### 70.4 Oro‐facial cleft/craniofacial malformations

####### 70.4.1 Cohort studies

Pooled results from four studies suggested no evidence of a difference in risk (RR 0.39, 95% CI 0.05 to 3.35, I^2^ = NA), with no difference in the number of oro‐facial cleft/craniofacial malformations in children exposed to OXC (N = 198) and children exposed to TPM (N = 410) (Analysis 70.4). The RD also suggested no difference in the level of risk (RD ‐0.01, 95% CI ‐0.03 to 0.01; I^2^ = 0%).

In the EURAP 2018 data, the prevalence of cleft malformations (other oro‐facial not specifically reported) in those exposed to OXC was 0.30% (1/333) and 0% for children exposed to TPM (0/152).

####### 70.4.2 Routine health record studies

No included studies reported data on this outcome.

###### 70.5 Skeletal/limb malformations

####### 70.5.1 Cohort studies

Pooled results from four cohort studies suggested no evidence of a difference in risk (RR 0.40, 95% CI 0.07 to 2.44; I^2^ = 0%), with no difference in the number of skeletal/limb malformations in children exposed to OXC (N = 266) and children exposed to TPM (N = 418) (Analysis 70.5). The RD also suggested no difference in the level of risk (RD −0.01, 95% CI −0.03 to 0.01; I^2^ = 0%).

####### 70.5.2 Routine health record studies

No included studies reported data on this outcome.

##### 71 OXC versus ZNS

###### 71.1 All major malformations

####### 71.1.1 Cohort studies

Pooled results from two cohort studies suggested no evidence of a difference in risk (RR 4.48, 95% CI 0.24 to 82.23; I^2^ = NA), with no difference in the number of major malformations in children exposed to OXC (N = 186) and children exposed to ZNS (N = 91) ( Analysis 71.1). The RD also suggested no difference in the level of risk (RD 0.02, 95% CI −0.01 to 0.05; I^2^ = 0%).

####### 71.1.2 Routine health record studies

No included studies reported data on this outcome.

###### 71.2 Neural tube malformations

####### 71.2.1 Cohort studies

We were unable to estimate a RR from one study due to there being no reported cases of neural tube malformations in children exposed to OXC (N = 4) or ZNS (N = 1) (Analysis 71.2).

####### 71.2.2 Routine health record studies

No included studies reported data on this outcome.

###### 71.3 Cardiac malformations

####### 71.3.1 Cohort studies

We were unable to estimate a RR from one study due to there being no reported cases of cardiac malformations in children exposed to OXC (N = 4) or ZNS (N = 1) (Analysis 71.3).

####### 71.3.2 Routine health record studies

No included studies reported data on this outcome.

###### 71.4 Oro‐facial cleft/craniofacial malformations

####### 71.4.1 Cohort studies

We were unable to estimate a RR from one study due to there being no reported cases of oro‐facial cleft/ craniofacial malformations in children exposed to OXC (N = 4) or ZNS (N = 1) (Analysis 71.4).

####### 71.4.2 Routine health record studies

No included studies reported data on this outcome.

###### 71.5 Skeletal/limb malformations

####### 71.5.1 Cohort studies

We were unable to estimate a RR from one study due to there being no reported cases of skeletal/limb malformations in children exposed to OXC (N = 4) or ZNS (N = 1) (Analysis 71.5).

####### 71.5.2 Routine health record studies

No included studies reported data on this outcome.

##### 72 PRM versus TPM

###### 72.1 All major malformations

####### 72.1.1 Cohort studies

Results from one study suggested no evidence of a difference in risk (RR 6.00, 95% CI 0.30 to 118.36, I^2^ = NA), with no difference in the number of major malformations in children exposed to PRM (N = 2) and children exposed to TPM (N = 53) (Analysis 72.1). The RD also suggested no difference in the level of risk (RD ‐0.02, 95% CI −0.44 to 0.41; I^2^ = NA).

####### 72.1.2 Routine health record studies

We were unable to estimate a RR from one study due to there being no reported cases of major malformations in children exposed to PRM (N = 3) or TPM (N = 1) (Analysis 72.1).

###### 72.2 Neural tube malformations

####### 72.2.1 Cohort studies

No included studies reported data on this outcome.

####### 72.2.2 Routine health record studies

No included studies reported data on this outcome.

###### 72.3 Cardiac malformations

####### 72.3.1 Cohort studies

No included studies reported data on this outcome.

####### 72.3.2 Routine health record studies

No included studies reported data on this outcome.

###### 72.4 Oro‐facial cleft/craniofacial malformations

####### 72.4.1 Cohort studies

No included studies reported data on this outcome.

####### 72.4.2 Routine health record studies

No included studies reported data on this outcome.

###### 72.5 Skeletal/limb malformations

####### 72.5.1 Cohort studies

No included studies reported data on this outcome.

####### 72.5.2 Routine health record studies

No included studies reported data on this outcome.

##### 73 PRM versus VPA

###### 73.1 All major malformations

####### 73.1.1 Cohort studies

Pooled results from six cohort studies suggested no evidence of a difference in risk (RR 0.74, 95% CI 0.39 to 1.40; I^2^ = 21%), with no difference in the number of major malformations in children exposed to PRM (N = 103) and children exposed to VPA (N = 491), (Analysis 73.1). The RD also suggested no difference in the level of risk (RD −0.04, 95% CI −0.13 to 0.04; I^2^ = 1%).

####### 73.1.2 Routine health record studies

Results from one study suggested no evidence of a difference in risk (RR 1.27, 95% CI 0.09 to 17.39, I^2^ = NA), with no difference in the number of major malformations in children exposed to PRM (N = 3) and children exposed to VPA (N = 268) (Analysis 73.1). The RD also suggested no difference in the level of risk (RD ‐0.10, 95% CI −0.42 to 0.23; I^2^ = NA).

###### 73.2 Neural tube malformations

####### 73.2.1 Cohort studies

Included studies did not reach the threshold for reporting of the meta‐analysis (Analysis 73.2). However, available data showed there were 0/39 cases of neural tube malformations in children exposed to PRM and 5/45 cases in children exposed to VPA, based on data from two studies (Milan Study 1999; Pardi 1982).

####### 73.2.2 Routine health record studies

No included studies reported data on this outcome.

###### 73.3 Cardiac malformations

####### 73.3.1 Cohort studies

Included studies did not meet the threshold for reporting of the meta‐analysis (Analysis 73.3). However, available data showed there were 1/39 cases of cardiac malformations in children exposed to PRM and 0/45 cases in children exposed to VPA, based on data from two studies (Milan Study 1999; Pardi 1982).

####### 73.3.2 Routine health record studies

No included studies reported data on this outcome.

###### 73.4 Oro‐facial cleft/craniofacial malformations

####### 73.4.1 Cohort studies

We were unable to estimate a RR from two studies due to there being no reported oro‐facial cleft/craniofacial malformations in children exposed to PRM (N = 39) or VPA (N = 45). (Analysis 73.4).

####### 73.4.2 Routine health record studies

No included studies reported data on this outcome.

###### 73.5 Skeletal/limb malformations

####### 73.5.1 Cohort studies

Included studies did not meet the threshold for reporting of the meta‐analysis (Analysis 73.5). However, available data showed there were 0/39 cases of skeletal/limb malformations in children exposed to PRM and 1/45 cases in children exposed to VPA, based on data from two studies (Milan Study 1999; Pardi 1982).

####### 73.5.2 Routine health record studies

No included studies reported data on this outcome.

##### 74 LEV vs LAC

###### 74.1 All major malformations

####### 74.1.1 Cohort studies

We were unable to estimate a RR for one cohort study due to there being no major malformations observed in children exposed to LEV (N = 12) or LAC (N = 1) (Analysis 74.1).

####### 74.1.2 Routine health record studies

No included studies reported data on this outcome.

Due to limited numbers, we did not investigate specific malformation types.

##### 75 CBZ versus LAC

###### 75.1 All major malformations

####### 75.1.1 Cohort studies

We could not estimate RR from one cohort study as there were no malformations observed in children exposed to CBZ (N = 7) and children exposed to LAC (N = 1) (Analysis 75.1).

####### 75.1.2 Routine health record studies

No included studies reported data on this outcome.

Due to limited numbers, we did not investigate specific malformation types.

##### 76 OXC vs LAC

###### 76.1 All major malformations

####### 76.1.1 Cohort studies

We were unable to estimate the RR for one cohort study due to there being no major malformations observed in children exposed to OXC (N = 4) or LAC (N = 1) (Analysis 76.1).

####### 76.1.2 Routine health record studies

No included studies reported data on this outcome.

Due to limited numbers, we did not investigate specific malformation types.

##### 77 PB versus LAC

###### 77.1 All major malformations

####### 77.1.1 Cohort studies

We were unable to estimate a RR from one cohort study due to there being no malformations observed in children exposed to PB (N = 2) and children exposed to LAC (N = 1) (Analysis 77.1).

####### 77.1.2 Routine health record studies

No included studies reported data on this outcome.

Due to limited numbers, we did not investigate specific malformation types.

##### 78 LAC vs ZNS

###### 78.1 All major malformations

####### 78.1.1 Cohort studies

We were unable to estimate a RR from one cohort study due to there being no major malformations observed in children exposed to LAC (N = 1) or ZNS (N = 1) (Analysis 78.1).

####### 78.1.2 Routine health record studies

No included studies reported data on this outcome.

Due to limited numbers, we did not investigate specific malformation types.

##### 79 GPB versus PGB

###### 79.1 All major malformations

####### 79.1.1 Cohort studies

We could not estimate RR from one cohort study as there were no malformations observed in children exposed to GPB (N = 14) and children exposed to PGB (N = 1) (Analysis 79.1).

####### 79.1.2 Routine health record studies

No included studies reported data on this outcome.

Due to limited numbers, we did not investigate specific malformation types.

##### 80 GBP vs CZP

###### 80.1 All major malformations

####### 80.1.1 Cohort studies

No included studies reported data on this outcome.

####### 80.1.2 Routine health record studies

Included studies did not meet the threshold for reporting of the meta‐analysis (Analysis 80.1). However, available data showed there were 0/18 cases of major malformations in children exposed to GBP and 2/48 cases in children exposed to CZP from one study (Sweden Health Record Registers).

Due to limited numbers, we did not investigate specific malformation types.

##### 81 VPA vs BNZ

###### 81.1 All major malformations

####### 81.1.1 Cohort studies

Included studies did not meet the threshold for reporting of the meta‐analysis (Analysis 81.1). However, available data showed there were 4/44 cases of major malformations in children exposed to VPA and 0/5 cases in children exposed to BNZ from two studies (Jimenez 2020; Melikova 2020).

####### 81.1.2 Routine health record studies

No included studies reported data on this outcome.

Due to limited numbers, we did not investigate specific malformation types.

##### 82 LTG versus BNZ

###### 82.1 All major malformations

####### 82.1.1 Cohort studies

We were unable to estimate a RR from two cohort studies due to there being no malformations observed in children exposed to LTG (N = 26) and children exposed to BNZ (N = 5) (Analysis 82.1).

####### 82.1.2 Routine health record studies

No included studies reported data on this outcome.

Due to limited numbers, we did not investigate specific malformation types.

##### 83 LEV versus BNZ

###### 83.1 All major malformations

####### 83.1.1 Cohort studies

We were unable to estimate a RR from two cohort studies due to there being no malformations observed in children exposed to LEV (N = 18) and children exposed to BNZ (N = 5) (Analysis 83.1).

####### 83.1.2 Routine health record studies

No included studies reported data on this outcome.

Due to limited numbers, we did not investigate specific malformation types.

##### 84 CBZ vs BNZ

###### 84.1 All major malformations

####### 84.1.1 Cohort studies

Included studies did not meet the threshold for reporting of the meta‐analysis (Analysis 84.1). However, available data showed there were 1/43 cases of major malformations in children exposed to CBZ and 0/5 cases in children exposed to BNZ, based on data from two studies (Jimenez 2020; Melikova 2020).

####### 84.1.2 Routine health record studies

No included studies reported data on this outcome.

Due to limited numbers, we did not investigate specific malformation types.

##### 85 OXC versus BNZ

###### 85.1 All major malformations

####### 85.1.1 Cohort studies

We were unable to estimate a RR from one cohort study due to there being no malformations observed in children exposed to OXC (N = 4) and children exposed to BNZ (N = 2) (Analysis 85.1).

####### 85.1.2 Routine health record studies

No included studies reported data on this outcome.

##### 86 PB vs BNZ

###### 86.1 All major malformations

####### 86.1.1 Cohort studies

We were unable to estimate a RR for one cohort study due to there being no major malformations observed in children exposed to PB (N = 2) or BNZ (N = 2) (Analysis 86.1).

####### 86.1.2 Routine health record studies

No included studies reported data on this outcome.

Due to limited numbers, we did not investigate specific malformation types.

##### 87 LAC versus BNZ

###### 87.1 All major malformations

####### 87.1.1 Cohort studies

We were unable to estimate a RR from one cohort study due to there being no malformations observed in children exposed to OXC (N = 4) and children exposed to BNZ (N = 2) (Analysis 87.1).

####### 87.1.2 Routine health record studies

No included studies reported data on this outcome.

Due to limited numbers, we did not investigate specific malformation types.

##### 88 ZNS vs BNZ

###### 88.1 All major malformations

####### 88.1.1 Cohort studies

We were unable to estimate a RR for one cohort study due to there being no major malformations observed in children exposed to ZNS (N = 1) or BNZ (N = 2) (Analysis 88.1).

####### 88.1.2 Routine health record studies

No included studies reported data on this outcome.

Due to limited numbers, we did not investigate specific malformation types.

##### 89 CZP versus TPM

###### 89.1 All major malformations

####### 89.1.1 Cohort studies

Pooled results from two studies suggested no evidence of a difference in risk (RR 0.67, 95% CI 0.07 to 1.87, I^2^ = NA), with no difference in the number of major malformations in children exposed to CZP (N = 26) and children exposed to TPM (N = 53) (Analysis 89.1). The RD also suggested no difference in the level of risk (RD ‐0.02, 95% CI −0.09 to 0.05; I^2^ = NA).

####### 89.1.2 Routine health record studies

Pooled results from two studies suggested no evidence of a difference in risk (RR 0.37, 95% CI 0.03 to 15.83, I^2^ = 0%), with no difference in the number of major malformations in children exposed to CZP (N = 26) and children exposed to TPM (N = 53) (Analysis 89.1). The RD also suggested no difference in the level of risk (RD ‐0.02, 95% CI −0.09 to 0.05; I^2^ = 0%).

Due to limited numbers, we did not investigate specific malformation types.

##### 90 CZP vs OXC

###### 90.1 All major malformations

####### 90.1.1 Cohort studies

Included studies did not meet the threshold for reporting of the meta‐analysis (Analysis 90.1). However, available data showed there were 0/26 cases of major malformations in children exposed to CZP and 1/19 cases in children exposed to OXC, based on data from one study (Australian Epilepsy and Pregnancy Register).

####### 90.1.2 Routine health record studies

Pooled results from two routine health record studies suggested no evidence of a difference in risk (RR 0.81, 95% CI 0.13 to 5.06; I^2^ = 0%), with no difference between children exposed to CZP (N = 161) and children exposed to OXC (N = 61) (Analysis 90.1). The RD also suggested no difference in the level of risk (RD ‐0.04, 95% CI −0.04 to 0.05; I^2^ = 0%).

Due to limited numbers, we did not investigate specific malformation types.

##### 91 CZP versus COZ

###### 91.1 All major malformations

####### 91.1.1 Cohort studies

We were unable to estimate a RR for one cohort study due to there being no major malformations observed in children exposed to CZP (N = 26) or COZ (N = 2) (Analysis 91.1).

####### 91.1.2 Routine health record studies

No included studies reported data on this outcome.

Due to limited numbers, we did not investigate specific malformation types.

##### 92 CZP vs ESM

###### 92.1 All major malformations

####### 92.1.1 Cohort studies

We were unable to estimate the RR for one cohort study due to there being no reported major malformations observed in children exposed to CZP (N = 48) or ESM (N = 8) (Analysis 92.1).

####### 92.1.2 Routine health record studies

Included studies did not meet the threshold for reporting of the meta‐analysis (Analysis 92.1). However, available data showed there were 2/48 cases of major malformations in children exposed to CZP and 0/8 cases in children exposed to ESM, based on data from one study (Sweden Health Record Registers).

Due to limited numbers, we did not investigate specific malformation types.

##### 93 CZP versus PRG

###### 93.1 All major malformations

####### 93.1.1 Cohort studies

We were unable to estimate a RR for one cohort study due to there being no major malformations observed in children exposed to CZP (N = 26) or children exposed to PRG (N = 1) (Analysis 93.1).

####### 93.1.2 Routine health record studies

No included studies reported data on this outcome.

Due to limited numbers, we did not investigate specific malformation types.

##### 94 CZP vs PRM

###### 94.1 All major malformations

####### 94.1.1 Cohort studies

We were unable to estimate a RR for one cohort study due to there being no major malformations observed in children exposed to CZP (N = 26) or PRM (N = 2) (Analysis 94.1).

####### 94.1.2 Routine health record studies

Included studies did not meet the threshold for reporting of the meta‐analysis (Analysis 94.1). However, available data showed there were 2/48 cases of major malformations in children exposed to CZP and 0/3 cases in children exposed to PRM, based on data from one study (Sweden Health Record Registers).

Due to limited numbers, we did not investigate specific malformation types.

##### 95 CZP versus VGB

###### 95.1 All major malformations

####### 95.1.1 Cohort studies

We were unable to estimate RR for one cohort study due to there being no major malformations observed in children exposed to CZP (N = 26) or VGB (N = 1) (Analysis 95.1).

####### 95.1.2 Routine health record studies

Included studies did not meet the threshold for reporting of the meta‐analysis (Analysis 95.1). However, available data showed there were 2/48 cases of major malformations in children exposed to CZP and 0/3 cases in children exposed to VGB, based on data from one study (Sweden Health Record Registers).

Due to limited numbers, we did not investigate specific malformation types.

##### 96 TPM vs BNZ

###### 96.1 All major malformations

####### 96.1.1 Cohort studies

We were unable to estimate a RR for two cohort studies due to there being no major malformations observed in children exposed to TPM (N = 7) or BNZ (N = 5) (Analysis 96.1).

####### 96.1.2 Routine health record studies

No included studies reported data on this outcome.

Due to limited numbers, we did not investigate specific malformation types.

##### 97 ESM versus VPA

###### 97.1 All major malformations

####### 97.1.1 Cohort studies

Results from one study suggested no evidence of a difference in risk (RR 0.56, 95% CI 0.04 to 8.03, I^2^ = NA), with no difference in the number of major malformations in children exposed to ESM (N = 5) and children exposed to VPA (N = 290) (Analysis 97.1). The RD also suggested no difference in the level of risk (RD ‐0.15, 95% CI −0.37 to 0.08; I^2^ = NA).

####### 97.1.2 Routine health record studies

Results from one study suggested no evidence of a difference in risk (RR 0.56, 95% CI 0.04 to 8.84, I^2^ = NA), with no difference in the number of major malformations in children exposed to ESM (N = 8) and children exposed to VPA (N = 268) (Analysis 97.1). The RD also suggested no difference in the level of risk (RD ‐0.10, 95% CI −0.25 to 0.06; I^2^ = NA).

Due to limited numbers, we did not investigate specific malformation types.

##### 98 ESM vs CBZ

###### 98.1 All major malformations

####### 98.1.1 Cohort studies

Results from one study suggested no evidence of a difference in risk (RR 1.39, 95% CI 0.10 to 20.37, I^2^ = NA), with no difference in the number of major malformations in children exposed to ESM (N = 4) and children exposed to CBZ (N = 409) (Analysis 98.1). The RD also suggested no difference in the level of risk (RD ‐0.06, 95% CI −0.28 to 0.16; I^2^ = NA).

####### 98.1.2 Routine health record studies

Results from one study suggested no evidence of a difference in risk (RR 1.37, 95% CI 0.09 to 20.78, I^2^ = NA), with no difference in the number of major malformations in children exposed to ESM (N = 8) and children exposed to CBZ (N = 703) (Analysis 98.1). The RD also suggested no difference in the level of risk (RD ‐0.04, 95% CI −0.19 to 0.11; I^2^ = NA).

Due to limited numbers, we did not investigate specific malformation types.

##### 99 ESM versus PRM

###### 99.1 All major malformations

####### 99.1.1 Cohort studies

We were unable to estimate a RR for one cohort study due to there being no major malformations observed in children exposed to ESM (N = 5) or PRM (N = 2) (Analysis 99.1).

####### 99.1.2 Routine health record studies

We were unable to estimate a RR for one routine health record study due to there being no major malformations observed in children exposed to ESM (N = 8) or PRM (N = 3) (Analysis 99.1).

Due to limited numbers, we did not investigate specific malformation types.

##### 100 ESM vs PB

###### 100.1 All major malformations

####### 100.1.1 Cohort studies

We were unable to estimate the RR for one cohort study due to there being no reported major malformations observed in children exposed to ESM (N = 5) or PB (N = 2) (Analysis 100.1).

####### 100.1.2 Routine health record studies

Included studies did not meet the threshold for reporting of the meta‐analysis (Analysis 100.1). However, available data showed there were 0/8 cases of major malformations in children exposed to ESM and 1/7 cases in children exposed to PB, based on data from one study (Sweden Health Record Registers).

Due to limited numbers, we did not investigate specific malformation types.

##### 101 ESM versus PHT

###### 101.1 All major malformations

####### 101.1.1 Cohort studies

Included studies did not meet the threshold for reporting of the meta‐analysis (Analysis 101.1). However, available data showed there were 0/5 cases of major malformations in children exposed to ESM and 1/44 cases in children exposed to PHT, based on data from one study (Australian Epilepsy and Pregnancy Register).

####### 101.1.2 Routine health record studies

Results from one study suggested no evidence of a difference in risk (RR 0.77, 95% CI 0.05 to 12.42, I^2^ = NA), with no difference in the number of major malformations in children exposed to ESM (N = 8) and children exposed to PHT (N = 103) (Analysis 101.1). The RD also suggested no difference in the level of risk (RD ‐0.07, 95% CI −0.23 to 0.09; I^2^ = NA).

Due to limited numbers, we did not investigate specific malformation types.

##### 102 ESM vs OXC

###### 102.1 All major malformations

####### 102.1.1 Cohort studies

Included studies did not meet the threshold for reporting of the meta‐analysis (Analysis 102.1). However, available data showed there were 0/5 cases of major malformations in children exposed to ESM and 1/19 cases in children exposed to OXC, based on data from one study (Australian Epilepsy and Pregnancy Register).

####### 102.1.2 Routine health record studies

We were unable to estimate the RR for one routine health record study due to there being no reported no major malformations observed in children exposed to ESM (N = 8) or OXC (N = 4) (Analysis 102.1).

Due to limited numbers, we did not investigate specific malformation types.

##### 103 ESM versus VGB

###### 103.1 All major malformations

####### 103.1.1 Cohort studies

We were unable to estimate a RR for one cohort study due to there being no major malformations observed in children exposed to ESM (N = 5) or VBG (N = 1) (Analysis 103.1).

####### 103.1.2 Routine health record studies

We were unable to estimate a RR for one routine health record study due to there being no major malformations observed in children exposed to ESM (N = 8) or VGB (N = 3) (Analysis 103.1).

Due to limited numbers, we did not investigate specific malformation types.

##### 104 ESM vs LTG

###### 104.1 All major malformations

####### 104.1.1 Cohort studies

Results from one study suggested no evidence of a difference in risk (RR 1.65, 95% CI 0.11 to 24.30, I^2^ = NA), with no difference in the number of major malformations in children exposed to ESM (N = 5) and children exposed to LTG (N = 406) (Analysis 104.1). The RD also suggested no difference in the level of risk (RD ‐0.05, 95% CI −0.27 to 0.17; I^2^ = NA).

####### 104.1.2 Routine health record studies

Results from one study suggested no evidence of a difference in risk (RR 1.12, 95% CI 0.07 to 19.24, I^2^ = NA), with no difference in the number of major malformations in children exposed to ESM (N = 8) and children exposed to LTG (N = 90) (Analysis 104.1). The RD also suggested no difference in the level of risk (RD ‐0.04, 95% CI −0.20 to 0.11; I^2^ = NA).

Due to limited numbers, we did not investigate specific malformation types.

##### 105 ESM versus TPM

###### 105.1 All major malformations

####### 105.1.1 Cohort studies

Results from one study suggested no evidence of a difference in risk (RR 3.00, 95% CI 0.14 to 65.77, I^2^ = NA), with no difference in the number of major malformations in children exposed to ESM (N = 8) and children exposed to TPM (N = 53) (Analysis 105.1) The RD also suggested no difference in the level of risk (RD ‐0.02, 95% CI −0.24 to 0.21; I^2^ = NA).

####### 105.1.2 Routine health record studies

We were unable to estimate the RR for one routine health record study due to there being no major malformations observed in children exposed to ESM (N = 8) or TPM (N = 1) (Analysis 105.1).

Due to limited numbers, we did not investigate specific malformation types.

##### 106 ESM vs GBP

###### 106.1 All major malformations

####### 106.1.1 Cohort studies

No included studies reported data on this outcome.

####### 106.1.2 Routine health record studies

We were unable to estimate the RR for one routine health record study due to there being no reported major malformations observed in children exposed to ESM (N = 8) or GBP (N = 18) (Analysis 106.1).

Due to limited numbers, we did not investigate specific malformation types.

##### 107 VGB versus VPA

###### 107.1 All major malformations

####### 107.1.1 Cohort studies

Results from one study suggested no evidence of a difference in risk (RR 1.67, 95% CI 0.15 to 18.73, I^2^ = NA), with no difference in the number of major malformations in children exposed to VGB (N = 1) and children exposed to VPA (N = 290) (Analysis 107.1). The RD also suggested no difference in the level of risk (RD ‐0.15, 95% CI −0.75 to 0.45; I^2^ = NA).

####### 107.1.2 Routine health record studies

Results from one study suggested no evidence of a difference in risk (RR 1.27, 95% CI 0.09 to 17.39, I^2^ = NA), with no difference in the number of major malformations in children exposed to VGB (N = 3) and children exposed to VPA (N = 268) (Analysis 107.1). The RD also suggested no difference in the level of risk (RD ‐0.10, 95% CI −0.42 to 0.23; I^2^ = NA).

Due to limited numbers, we did not investigate specific malformation types.

##### 108 VGB vs CBZ

###### 108.1 All major malformations

####### 108.1.1 Cohort studies

Results from one study suggested no evidence of a difference in risk (RR 4.18, 95% CI 0.37 to 47.57, I^2^ = NA), with no difference in the number of major malformations in children exposed to VGB (N = 1) and children exposed to CBZ (N = 409) (Analysis 108.1). The RD also suggested no difference in the level of risk (RD ‐0.06, 95% CI −0.66 to 0.54; I^2^ = NA).

####### 108.1.2 Routine health record studies

Results from one study suggested no evidence of a difference in risk (RR 3.09, 95% CI 0.23 to 42.31, I^2^ = NA), with no difference in the number of major malformations in children exposed to VGB (N = 3) and children exposed to CBZ (N = 703) (Analysis 108.1). The RD also suggested no difference in the level of risk (RD ‐0.04, 95% CI −0.36 to 0.28; I^2^ = NA).

Due to limited numbers, we did not investigate specific malformation types.

##### 109 VGB versus PRM

###### 109.1 All major malformations

####### 109.1.1 Cohort studies

We were unable to estimate a RR for one cohort study due to there being no major malformations observed in children exposed to VGB (N = 1) or PRM (N = 2) (Analysis 109.1).

####### 109.1.2 Routine health record studies

We were unable to estimate a RR for one routine health record study due to there being no major malformations observed in children exposed to VGB (N = 3) or PRM (N = 3) (Analysis 109.1).

Due to limited numbers, we did not investigate specific malformation types.

##### 110 VGB vs PB

###### 110.1 All major malformations

####### 110.1.1 Cohort studies

No included studies reported data on this outcome.

####### 110.1.2 Routine health record studies

Included studies did not meet the threshold for reporting of the meta‐analysis (Analysis 110.1). However, available data showed there were 0/3 cases of major malformations in children exposed to VGB and 1/7 cases in children exposed to PB, based on data from one study (Sweden Health Record Registers).

Due to limited numbers, we did not investigate specific malformation types.

##### 111 VGB versus PHT

###### 111.1 All major malformations

####### 111.1.1 Cohort studies

Included studies did not meet the threshold for reporting of the meta‐analysis (Analysis 111.1). However, available data showed there were 0/1 cases of major malformations in children exposed to VGB and 1/44 cases in children exposed to PHT, based on data from one study (Australian Epilepsy and Pregnancy Register).

####### 111.1.2 Routine health record studies

Results from one study suggested no evidence of a difference in risk (RR 1.73, 95% CI 0.13 to 25.35, I^2^ = NA), with no difference in the number of major malformations in children exposed to VGB (N = 3) and children exposed to PHT (N = 103) (Analysis 111.1). The RD also suggested no difference in the level of risk (RD ‐0.07, 95% CI −0.40 to 0.28; I^2^ = NA).

Due to limited numbers, we did not investigate specific malformation types.

##### 112 VGB vs OXC

###### 112.1 All major malformations

####### 112.1.1 Cohort studies

Included studies did not meet the threshold for reporting of the meta‐analysis (Analysis 112.1). However, available data showed there were 0/1 cases of major malformations in children exposed to VGB and 1/19 cases in children exposed to OXC, based on data from one study (Australian Epilepsy and Pregnancy Register).

####### 112.1.2 Routine health record studies

We were unable to estimate the RR for one routine health record study due to there being no reported major malformations observed in children exposed to VGB (N = 3) or OXC (N = 4) (Analysis 112.1).

Due to limited numbers, we did not investigate specific malformation types.

##### 113 VGB versus LTG

###### 113.1 All major malformations

####### 113.1.1 Cohort studies

Results from one study suggested no evidence of a difference in risk (RR 3.31, 95% CI 0.25 to 43.03, I^2^ = NA), with no difference in the number of major malformations in children exposed to VGB (N = 2) and children exposed to LTG (N = 406) (Analysis 113.1). The RD also suggested no difference in the level of risk (RD ‐0.05, 95% CI −0.47 to 0.37; I^2^ = NA).

####### 113.1.2 Routine health record studies

Results from one study suggested no evidence of a difference in risk (RR 2.53, 95% CI 0.16 to 39.34, I^2^ = NA), with no difference in the number of major malformations in children exposed to VGB (N = 3) and children exposed to LTG (N = 90) (Analysis 113.1). The RD also suggested no difference in the level of risk (RD ‐0.04, 95% CI −0.37 to 0.28; I^2^ = NA).

Due to limited numbers, we did not investigate specific malformation types.

##### 114 VGB vs TPM

###### 114.1 All major malformations

####### 114.1.1 Cohort studies

Results from one study suggested no evidence of a difference in risk (RR 9.00, 95% CI 0.51 to 159.15, I^2^ = NA), with no difference in the number of major malformations in children exposed to VGB (N = 1) and children exposed to TPM (N = 53) (Analysis 114.1). The RD also suggested no difference in the level of risk (RD ‐0.02, 95% CI −0.62 to 0.58; I^2^ = NA).

####### 114.1.2 Routine health record studies

We were unable to estimate the RR for one routine health record study due to there being no reported major malformations observed in children exposed to VGB (N = 3) or TPM (N = 1) (Analysis 114.1).

Due to limited numbers, we did not investigate specific malformation types.

##### 115 VGB vs GBP

###### 115.1 All major malformations

####### 115.1.1 Cohort studies

No included studies reported data on this outcome.

####### 115.1.2 Routine health record studies

We were unable to estimate the RR for one routine health record study due to there being no reported no major malformations observed in children exposed to VGB (N = 3) or GBP (N = 18) (Analysis 115.1).

Due to limited numbers, we did not investigate specific malformation types.

##### 116 CZP vs PB

###### 116.1 All major malformations

####### 116.1.1 Cohort studies

Included studies did not meet the threshold for inclusion in the meta‐analysis (Analysis 116.1). However, available data showed there were 0/27 cases of major malformations in children exposed to CZP and 1/6 cases in children exposed to PB, based on data from two studies (Australian Epilepsy and Pregnancy Register; D'Souza 1991).

####### 116.1.2 Routine health record studies

Pooled results from two studies suggested no evidence of a difference in risk (RR 0.26, 95% CI 0.06 to 1.12, I^2^ = 0%), with no difference in the number of major malformations in children exposed to CZP (N = 161) and children exposed to PB (N = 34) (Analysis 116.1). The RD also suggested no difference in the level of risk (RD ‐0.07, 95% CI −0.16 to 0.03; I^2^ = 0%).

Due to limited numbers, we did not investigate specific malformation types.

##### 117 CZP vs PHT

###### 117.1 All major malformations

####### 117.1.1 Cohort studies

Pooled results from two studies suggested no evidence of a difference in risk (RR 0.71, 95% CI 0.10 to 5.11, I^2^ = 0%), with no difference in the number of major malformations in children exposed to CZP (N = 27) and children exposed to PHT (N = 66) (Analysis 117.1). The RD also suggested no difference in the level of risk (RD ‐0.04, 95% CI −0.13 to 0.06; I^2^ = 0%).

####### 117.1.2 Routine health record studies

Results from one study suggested no evidence of a difference in risk (RR 0.61, 95% CI 0.13 to 2.48, I^2^ = NA), with no difference in the number of major malformations in children exposed to CZP (N = 48) and children exposed to PHT (N = 103) (Analysis 117.1). The RD also suggested no difference in the level of risk (RD ‐0.03, 95% CI −0.10 to 0.05; I^2^ = NA).

Due to limited numbers, we did not investigate specific malformation types.

##### 118 ESM vs LEV

###### 118.1 All major malformations

####### 118.1.1 Cohort studies

Results from one study suggested no evidence of a difference in risk (RR 2.12, 95% CI 0.13 to 34.10, I^2^ = NA), with no difference in the number of major malformations in children exposed to ESM (N = 5) and children exposed to LEV (N = 139) (Analysis 118.1) The RD also suggested no difference in the level of risk (RD ‐0.04, 95% CI −0.26 to 0.19; I^2^ = NA).

####### 118.1.2 Routine health record studies

No included studies reported data on this outcome.

Due to limited numbers, we did not investigate specific malformation types.

##### 119 ESM vs Controls

###### 119.1 All major malformations

####### 119.1.1 ESM versus no medication (in women without epilepsy): cohort studies

No included studies reported data on this outcome.

####### 119.1.2 ESM versus no medication (in women with epilepsy): cohort studies

Results from one study suggested no evidence of a difference in risk (RR 2.68, 95% CI 0.17 to 43.16, I^2^ = NA), with no difference in the number of major malformations in children exposed to ESM (N = 5) and control children (N = 176) (Analysis 119.1). The RD also suggested no difference in the level of risk (RD ‐0.03, 95% CI −0.25 to 0.19; I^2^ = NA).

####### 119.1.3 ESM versus no medication (in women without epilepsy): routine health record studies

No included studies reported data on this outcome.

####### 119.1.4 ESM versus no medication (in women with epilepsy): routine health record studies

No included studies reported data on this outcome.

##### 120 VGB vs Control

###### 120.1 All major malformations

####### 120.1.1 VGB versus no medication (in women without epilepsy): cohort studies

No included studies reported data on this outcome.

####### 120.1.2 VGB versus no medication (in women with epilepsy): cohort studies

Results from one study suggested no evidence of a difference in risk (RR 8.05, 95% CI 0.64 to 101.76, I^2^ = NA), with no difference in the number of major malformations in children exposed to VGB (N = 1) and control children (N = 176) (Analysis 120.1). The RD also suggested no difference in the level of risk (RD ‐0.03, 95% CI −0.63 to 0.57; I^2^ = NA).

####### 120.1.3 VGB versus no medication (in women without epilepsy): routine health record studies

No included studies reported data on this outcome.

####### 120.1.4 VGB versus no medication (in women with epilepsy): routine health record studies

No included studies reported data on this outcome.

Due to limited numbers, we did not investigate specific malformation types.

#### Studies not included in the meta‐analysis and not narratively reported

The publications of EURAP 2018; Samren 1997 required narrative reporting due to their overlap with other research initiatives. Israeli Teratogen Service showed variability in its reporting and, therefore, required narrative reporting for certain outcomes. Further, studies using US Medicaid Registers also required narrative review due to the format of reporting of the monotherapy TPM and GBP pregnancies in women with epilepsy.

## Discussion

We reported results from three study types, meta‐analysis of data from cohort studies, data from EURAP 2018 and others, and meta‐analysis of data from epidemiological health record studies. Each study design has its inherent methodological strengths and weaknesses. We undertook a stratified approach to evidence synthesis to ensure a sensitive approach to combining data and to allow for the development of evidence groupings; this will allow for replication of findings across different study types and will also allow for increased confidence in the evidence .

The meta‐analyses included 17,964 ASM‐exposed pregnancies from cohort studies and 7913 from routine health record studies; additional exposed pregnancies from the EURAP collaboration and other studies were reviewed narratively. Individual ASM prevalence of major malformation ranged from 2.0% to 9.8% for cohort study data and 3.6% to 9.7% for studies utilising routine health record data (Table 3, Figure 3). Table 1 and Table 2 along with Table 3, Table 4, Table 5, Table 6, Table 7, Table 8 and Table 9 provide a summary of the meta‐analysis results for all comparisons for risk of major malformation. This update has included the most recent study data, which strengthens the previously identified risk associations for older ASMs such as CBZ, PB and VPA, where the comparisons include large numbers of exposed pregnancies. For other ASMs, there are some differences in the results across the comparisons, but the better powered comparisons demonstrate an increased risk for PHT and for an overall major malformation risk with CBZ and for TPM.

Whilst CBZ, PB, PHT, TPM and VPA are associated with an increase in the risk of major malformation, the level of risk varies between the four ASMs. For example, CBZ showed a lower overall major malformation risk rate than PB and VPA, and a lower risk for specific major malformation types compared to some ASMs (e.g. PB or TPM). All ASMs, regardless of their own association with an increased risk, carried a lower risk than VPA‐exposure which had the highest prevalence from both cohort (9.8%) and routine health record data (9.7%). LTG remains the lowest risk ASM with adequate cohort size (N => 4700 pregnancies from cohort studies and N = 2502 from routine health record studies). LTG does appear to have a dose effect, as do CBZ, PB and VPA. The number of LEV‐exposed pregnancies remains more limited than LTG but, in direct comparison, there is currently no significant difference between these two ASMs, and LEV‐exposed children have a lower overall major malformation risk than CBZ, VPA and PHT. TPM exposure was associated with a higher overall major malformation risk in comparison to LTG, but not other ASMs; this finding may be due to relatively small numbers of participants in the TPM studies and only a few malformation events. TPM exposure, however, was associated with higher specific risks of oro‐facial, craniofacial, skeletal and limb malformations in comparison to LTG, LEV, CBZ, but not in comparison to the two control groups; but specific major malformation data were limited for the control cohorts.

There remains limited information for GBP, OXC, TPM and ZNS and other ‘newer’ medications. The evidence for each ASM monotherapy is summarised below.

### Summary of main results

#### Carbamazepine (CBZ)

CBZ was the most frequently investigated ASM both in terms of the number of publications and the number of included pregnancies (over 8220). The pooled major malformation prevalence for CBZ was 4.7% (95% CI, 3.7 to 5.9) from cohort studies, 4.0% (95% CI 3.0 to 5.4) for routine health record studies and 2.9% (95% CI 2.9 to 5.4); these were relatively consistent with the 5.5% (95% CI 4.5 to 6.6) prevalence from EURAP 2018 (Figure 3).

In comparison to both children born to women without epilepsy and children born to women with untreated epilepsy, pooled data from the cohort studies found that children exposed to CBZ in utero had an increased risk of having a major malformation, with the difference in risk ranging from 1% to 2%, respectively. The findings from routine health record studies are more mixed. In the larger of the two comparisons, CBZ was significantly associated with a higher risk of a major malformation, which was consistent with the cohort study data.

In comparison to unexposed control children (both groups), there was no specific malformation which was increased above the background rate provided by the control children. Data were limited in terms of the specific malformation risk, mainly due to the absence of control data from some large pregnancy and epilepsy registry studies (e.g. North American Epilepsy and Pregnancy Register; UK and Ireland Epilepsy and Pregnancy Register) and reporting of this level of detail from the population datasets (e.g. Denmark Health Record Registers; UK Clinical Research Practice Database; UK Health Record THIN Register). This likely contributed to the non‐significant outcomes found for neural tube malformations, which has been an association found by others using different methodologies (Jentink 2010b).

Data from the cohort studies were more numerous for CBZ‐exposed pregnancies compared to other monotherapy ASMs. There was a higher risk of major malformation in comparison to children exposed to LEV or LTG in comparisons which included over 500 pregnancies in each arm, with the risk being 1% higher. However, comparisons at the specific malformation level were not significant; this was likely due to fewer data being available at this level of investigation. The increased risk observed in data from cohort studies was not replicated in studies using databases containing routine health records, however, patient/event numbers in the databases were smaller than the pooled experience from cohort studies.

Despite its associated higher risk, CBZ exposure was associated with a lower level of risk than VPA exposure, with a 5% difference in overall major malformation risk and a lower risk across neural tube, cardiac, oro‐facial and skeletal/limb malformations. Further, while CBZ is comparable to the increased risk from PB, PHT or TPM in data from cohort studies for overall major malformation, the specific malformation risk varied. The association of CBZ exposure with cardiac malformations was lower in comparison to PB or to PHT exposure and lower for skeletal/limb or oro‐facial/craniofacial malformation risk when compared to TPM exposure.

There was no significant increase in risk in comparison to OXC, GBP, PRM or CZP exposure from both cohort and health record studies, but data were more limited for these comparator ASMs currently and caution is required.

Dose is a key feature of teratogenic malformation risk (Brent 2004). Data from EURAP 2018 is the most reliable to investigate dose associations due to its large number of CBZ‐exposed pregnancies and its low risk of bias on the domain of outcome measurement. It demonstrates a dose‐related risk for CBZ with doses over 700 mg/d carrying a higher risk, although even their lower dose group had a high level of risk in comparison to children exposed to LTG (EURAP 2018).

#### Clonazepam (CZP)

Data relating to the use of monotherapy clonazepam during pregnancy and child major malformation risk is substantially limited with fewer than 150 pregnancies reported across both cohort and routine health record studies. The generated prevalence reported here was 2.1% (95% CI 0.2 to 17.3%) to 2.5% (95% CI 0.0 to 131.8) but the confidence intervals were very wide, due to the limited data. When compared to other ASMs, however, a lower risk was identified in comparison to VPA exposure, with a risk difference of 9%. Due to the current limited data available, no firm conclusion can be made currently whether CZP is associated with an increased risk in comparison to control children.

#### Gabapentin (GBP)

Experience with GBP exposure in pregnancy was also limited. Outcomes from only 210 reported pregnancies could be included in our meta‐analysis. The pooled prevalence of major malformation was 2.0% (95% CI 0.1 to 32.2) from cohort studies, with too few data being available from studies using routine health records to provide a pooled prevalence rate. We found no difference between the children exposed to GBP compared with either type of control group, but caution is warranted due to limited numbers. Data which could not be included in meta‐analysis, from the US Medicaid Registers, found that children exposed to GBP (N = 347) for the indication of maternal epilepsy were not at a greater risk of major malformation, which matched the wider finding from 3745 GBP‐exposed pregnancies (any indication). Whilst this study may offer reassurance, caution is required as the indications were not predominantly epilepsy and, therefore, there may be differences in typically prescribed doses; replication of this finding is also required in another adequately powered cohort.

We found no difference in overall major malformation rate or in the specific malformations investigated for the children exposed to GBP compared to CBZ, LTG, LEV, OXC, PHT, PB, TPM and ZNS, but there was a very limited number of GBP‐exposed children. In comparison to the children exposed to VPA, children exposed to GBP in utero had a significantly lower risk (8%) of having a malformation than children exposed to VPA, but data were too limited to investigate specific malformation differences.

Data for GBP dose and malformation rate were limited from cohort studies but data from the US Medicaid Registers failed to find an association between higher doses of GBP and a higher risk of malformation. However, the vast majority of women taking GBP were doing so for conditions other than epilepsy and, therefore, caution is warranted.

#### Lamotrigine (LTG)

The use of LTG has increased over the last decade in women of childbearing age (Ackers 2009; Man 2012; Wen 2015). The pooled prevalence of major malformation for LTG was 2.7% (95% CI 1.9 to 3.8%) from cohort studies, 3.5% (95% CI 2.5 to 4.9) from routine health record studies, and 2.9% (95% CI 2.3 to 3.7) from the EURAP 2018 collaboration. Most of the evidence indicated no difference in the overall major malformation rate between the children exposed to LTG and either type of control group. However, in comparison to children born to women without epilepsy, there was a significant difference from the pooled cohort data, with the risk being increased by 1% for the children exposed to LTG. Whilst levels of heterogeneity were not increased overall, one study had a much higher rate of malformation in the LTG group. Further, a larger LTG‐exposed group was available for comparison to the women with epilepsy who were not treated, which showed the LTG major malformation risk was not significantly different from the control children for overall malformation rate or for the specific malformation types investigated; this was consistent with the non‐significant finding from the routine health record studies in comparison to both control groups. Further, no increase in specific malformation types were identified, and data from the US Medicaid Registers failed to find an association with oral cleft malformations in over 2000 LTG‐exposed children in comparison to control children who were not exposed to ASMs.

In comparison to LEV, which has also seen a significant increase in use in women of childbearing age in a lot of countries (Meador 2009; Wen 2015), there were no significant differences for LTG in either the overall major malformation rate or the specific malformation types investigated. Children exposed to LTG also did not differ either in terms of overall major malformation rate or in terms of specific malformations compared with children exposed to OXC, CZP, GBP and ZNS; however, data were limited for all of these comparisons due to small numbers of OXC, CZP, GBP and ZNS‐exposed pregnancies.

The children exposed to LTG were at a significantly lower risk of overall major malformation compared with children exposed to CBZ, PB, PHT and TPM exposures (see results for specific risk levels) in cohort study data. Routine health record studies did not replicate this, however, were limited in terms of numbers of included LTG‐exposed pregnancies in comparison to PB, PHT and TPM and, therefore, were perceived as less reliable. Analyses of cohort study data showed that, at the specific malformation level, children exposed to LTG were at a lower risk of cardiac malformations in comparison to PB and PHT exposures and fewer skeletal malformations than children exposed to TPM and PHT. This latter finding was also observed in the large cohort from the US Medicaid Registers. Finally, children exposed to LTG had a three‐fold lower risk of overall major malformation when compared to the children exposed to VPA, with a risk difference showing that the significant reduction in risk was 6% for children exposed to LTG. Neural tube, cardiac, oro‐facial cleft, craniofacial, skeletal and limb malformations were all significantly lower for the LTG‐exposed children.

The large, well‐designed EURAP 2018 study has demonstrated a dose relationship between LTG treatment and major malformation risk, with exposures to LTG ≤ 325 mg/d associated with the lowest malformation prevalence. Other studies did not find a dose relationship, however, the EURAP 2018 collaboration is by far the largest and most standardised in their assessment of the malformation, comprising of participants from 42 countries (N = 2514). Therefore, higher doses of LTG may be associated with higher levels of risk and this should be considered when prescribing doses over 325 mg/d. It is possible that this dose association may account, at least in part, for the variation seen in the outcome in one of the control comparisons.

#### Levetiracetam (LEV)

The frequency of data and the number of included pregnancies exposed to LEV were more limited than for CBZ, LTG or VPA. There were 1242 LEV‐exposed pregnancies included from cohort data across all comparisons and just 248 from studies employing routine health record data currently. This delay is likely due in part to the time it takes for adequate numbers of women taking newer ASMs to accumulate, however, it undermines counselling for a now commonly established medication (Meador 2009; Wen 2015).

The pooled prevalence for major malformation occurrences following LEV‐exposure was 2.6% (95% CI 1.6 to 4.4) for cohort study data, 2.8% (95% CI 0.0 to 321.9) for routine health record study data (which had large confidence intervals) and 2.8% (95% CI 1.7 to 4.5) from EURAP 2018. There was no significant difference between the children exposed to LEV and control children in the meta‐analysis for overall major malformation rate; these comparisons also both contained > 1000 pregnancies in each arm. Data pertaining to specific malformation types in comparison to control children were limited, however, and it is not possible to draw conclusions until more data are available.

In comparison to other ASM treatments, children exposed to LEV were not significantly different from children exposed to LTG in terms of overall major malformation prevalence or the specific malformation types investigated. In addition, we found no significant difference between children exposed to LEV compared with those exposed to GBP, OXC, CZP, TPM ZNS or PRM, although data within these comparisons were somewhat limited. Children exposed to LEV had a lower overall MCM rate than the children exposed to CBZ, PB and PHT exposures, but there was no difference in terms of the specific malformation types investigated. While the overall major malformation risk was not significantly different for the children exposed to LEV versus those exposed to TPM, the children exposed to LEV were at lower risk of having an oro‐facial/craniofacial, skeletal or limb malformation in comparison to the TPM‐exposed children. Additionally, children exposed to LEV had a lower specific risk of developing a cardiac malformation in comparison to PB‐exposed children. Finally, the children exposed to LEV had a 7 to 8% lower risk of overall malformations compared with the children exposed to VPA, with a lower risk identified for all investigated specific types of malformations for the children exposed to LEV.

Investigation between dose of LEV and major malformation outcome was limited by numbers included within the individual studies (i.e. Denmark Health Record Registers; Kerala Epilepsy and Pregnancy Registry; North American Epilepsy and Pregnancy Register; UK and Ireland Epilepsy and Pregnancy Register), including the EURAP 2018 study. In 599 LEV‐exposed pregnancies, EURAP 2018 did not investigate lower versus higher‐dose LEV. Caution is required, therefore, regarding the malformation risk with above average doses of LEV, until more data are available.

#### Oxcarbazepine (OXC)

Data for pregnancy outcomes following exposure to OXC were limited to just under 400 pregnancies in cohort studies and 507 pregnancies from routine health record studies. The prevalence of major malformation was 2.8% (95% CI 1.1 to 6.6) versus 4.8% (95% CI 0.7 to 31.5) with routine health record studies containing more OXC‐exposed pregnancies across comparisons. The prevalence reported from EURAP 2018 for 333 OXC monotherapy‐exposed pregnancies was 3.0% (95% CI 1.4 to 5.4).

In comparison to control children, the pooled routine health record study data found no elevated risk for OXC exposure in comparison to controls; these results were similar in the cohort study data. Given the numbers of included OXC‐exposed pregnancies across both study types, further research is required for conclusions to be drawn in regard to OXC exposure and major malformation outcome. While limited comparisons to other ASMs could be made, where evidence was available, there was no significant difference between the overall major malformation rate or the specific malformations investigated compared with children exposed to CBZ, CZP, LEV, LTG, GBP, PHT, PB, TPM, PRM and ZNS. Children exposed to OXC were at a significantly lower risk of having an major malformation of any type compared with children exposed to VPA, with the risk difference being 4 to 6% depending on the study type. There were very limited data pertaining to specific malformation types, and caution is required.

Only EURAP 2018 reported dose and malformation rates for OXC‐exposed pregnancies. Whilst they did not compare lower versus higher OXC dose, they did report that certain dose levels of OXC were comparable to lower‐dose LTG. More studies of OXC‐exposed pregnancies are required, however, before it is determined whether a higher level of OXC dose carries a higher malformation risk.

#### Phenobarbital (PB)

Despite years of use, data from prospective studies investigating PB as monotherapy were surprisingly limited, with only 840 monotherapy‐exposed pregnancies across the different comparisons and study types. The data pooled from included studies generated a major malformation prevalence of 6.3% (95% CI 4.8 to 8.3) from cohort studies and 8.8% (95% CI 0.0 to 9722.4) from routine health record studies; the latter was limited in cohort size and the prevalence should be interpreted with caution. There was a prevalence of 6.5% (95% CI 4.2 to 9.9) from EURAP 2018.

The results regarding PB‐exposure in comparison to control children demonstrated variable results. We found a significantly increased risk of overall major malformation compared with children born to women without epilepsy, with a risk difference of 4%. However, we found no significant difference compared with children born to women without epilepsy. However, both comparisons included under 500 PB‐exposed pregnancies, which may account for the unstable pattern of the findings. Routine health record data studies included too few PB‐exposed pregnancies at this time to provide reliable estimates. Data pertaining to specific malformations were extremely limited or missing and likely contributed to the non‐significant differences found for PB in comparison to the control children. This is certainly the case for cardiac malformations, where rates of cardiac malformations are increased in comparison to numerous other ASMs exposures.

In comparison to other ASMs, children exposed to PB were not at a significantly increased rate of overall major malformation compared with children exposed to CBZ, CZP, GBP, PHT, OXC, TPM, PRM, LEV and ZNS exposures; but the comparison to CBZ exposure was the only one where the PB‐exposed group had over 500 pregnancies. PB exposure was significantly associated with an increased risk of oro‐facial clefts and craniofacial malformations when compared to LEV or LTG exposures. Children exposed to PB had a higher overall major malformation than the children exposed to LTG, but a lower risk compared with the children exposed to VPA, with the risk being 4% lower. Therefore, despite both PB and VPA being associated with an increased risk of being born with an major malformation, the risk associated with VPA is significantly higher, including for cardiac malformations.

The majority of studies did not investigate or report on a potential relationship between dose of PB and major malformation risk, due to limited included pregnancies. A dose‐mediated risk was also apparent for cardiac malformations, with the prevalence increasing from 1% to 8% for doses < 150 mg/d and those ≥ 150 mg/d, respectively (EURAP 2018). Samren 1997 also found a dose effect for PB. Given the size of the EURAP 2018 cohort and their standardised approach to reviewing, it is concluded that there is likely a strong dose relationship for PB.

#### Phenytoin (PHT)

The pooled prevalence of major malformation in the PHT‐exposed children was 5.4% (95% CI 3.6 to 8.1%) for cohort studies, 6.8% (95% CI 0.1 to 701.2%) for routine health record studies and 6.4% (95% CI 2.8 to 12.2%) for EURAP 2018. There were 1327 PHT‐exposed pregnancies included in the cohort studies, but just 103 children reported from routine health record studies. The children exposed to PHT were at a significantly increased risk in comparison with both types of control group, with the difference in risk being 3% in the cohort data. However, we found no association between PHT and specific major malformation types; although data were limited in these comparisons due to the limited control data reported in publications from the epilepsy and pregnancy registers.

In comparison to other ASMs, children exposed to PHT were not at an increased risk of overall major malformation compared with children exposed to CZP, CBZ, GBP, OXC, TPM, PRM, PB or ZNS; however, data comparing PHT with the 'newer' ASMs were limited and caution is needed in the interpretation of these non‐significant findings. In contrast, compared to studies with a greater number of included children, the children exposed to PHT were at an increased risk of overall major malformation compared with children exposed to LTG or LEV, with the risk difference indicating a 2% increase in major malformation; however, these RDs were not statistically significant. In contrast, the children exposed to PHT were significantly less likely to have a major malformation than the children exposed to VPA, with the difference in risk being 5% lower. Further, the children exposed to PHT were also at a lower risk than those exposed to VPA for their risk of neural tube, cardiac and skeletal/limb malformations.

In terms of specific malformations, children exposed to PHT were less likely than those exposed to PB to have a craniofacial malformation. There was a noted increase in cardiac, skeletal and limb malformations for the PHT‐exposed children compared with those exposed to LTG, which was one of the larger comparisons in terms of PHT‐exposed pregnancies. Finally, the rates of neural tube, cardiac, skeletal and limb malformations were significantly lower for the children exposed to PHT in comparison to the VPA‐exposed children.

The majority of studies did not report on whether the risk of being born with a major malformation was associated with dose of PHT; however, those that did investigate such an association did not show a consistent pattern (Kaaja 2003; Kaneko 1999; Motherisk Registry; North American Epilepsy and Pregnancy Register; Samren 1997), therefore, the conclusion around dose effects is uncertain.

#### Primidone (PRM)

This is an old ASM with limited utilisation currently. Evidence pertaining to PRM was extremely limited to 112 pregnancies and caution is warranted when interpreting results. Pooled data from included cohort studies gave a malformation prevalence of 7.9% (95% CI 2.6 to 21.5%). There were just 3 PRM‐exposed cases reported in the routine health record studies. The children exposed to PRM were at a higher risk of overall major malformation in comparison to the children born to women with an untreated epilepsy, which contained the larger number of PRM‐exposed pregnancies. A comparable major malformation risk was found for PRM in comparison to PHT, PB and VPA exposures, but the data were limited. There was either extremely limited or no data available to compare risks to other monotherapy ASMs.

Only one study of 19 PRM cases investigated the dose of PRM and outcome (Kaneko 1999). Therefore, it remains unknown whether there is an association between PRM dose and increased major malformation risk.

#### Topiramate (TPM)

Experience with TPM was limited to 510 exposed pregnancies in cohort studies (3.9%, 95% CI 2.3 to 6.5%). There were 49 cases from routine health record investigations which met the criteria for being included in the meta‐analyses (4.1%, 95% CI 0.0 to 27,060.0); therefore, caution is required when considering our results. The EURAP 2018 collaboration is also limited currently in its experience with TPM exposures with just 152 exposed pregnancies with a major malformation prevalence of 3.9% (95% CI 1.5 to 8.4%).

In pooled cohort data, in comparison to children born to women without epilepsy, children exposed to TPM had a higher rate of being born with an major malformation with the risk difference being 3%. We found no significant difference compared with the no medication control group, but this comparison had even fewer TPM cases. Pooled data were too limited here to allow for the investigation of specific malformation outcomes in comparison to control children. We found no significant difference in the rate of major malformation compared with children exposed to CBZ, CZP GBP, PHT, PB, PRM, OXC and ZNS. We found a significant increase in the rate of major malformation for the children exposed to TPM compared with the children exposed to LTG, with skeletal/limb and oro‐facial cleft/craniofacial being specifically increased.

Data from US Medicaid Registers provides the largest dataset regarding oro‐facial clefts and reported an association between topiramate and oral clefts from medical reimbursement databases (4.1 per 1000 live births). This is similar to a retrospective study which was not included in this review (Mines 2014), in a case‐control study (Margulis 2012), and in a previous meta‐analysis (Alsaad 2015) which were beyond the inclusion criteria of this review. This demonstrates the cohort sizes which are required to investigate very specific rare events, such as specific types of major malformation.

The overall major malformation risk was comparable to that for LEV or CBZ‐exposed children, but the LEV‐exposed children were at a lower risk of skeletal and limb malformations, as were the CBZ‐exposed children. The children exposed to TPM had a lower cardiac risk than the children exposed to PB, and they were less likely to have a malformation of any type compared with the children exposed to VPA, with the difference in risk being 7%.

Most studies were too limited to be able to provide reliable investigations into a dose association, however, Hernandez‐Diaz, using (US Medicaid Registers) data, found that the adjusted RRs for oro‐facial clefts at doses ≤ 100 mg/d and > 100 mg/d were 1.64 (95% CI 0.53 to 5.07) and 5.16 (95% CI 1.94 to 13.73) for lower and higher doses, respectively.

#### Valproate (VPA)

In utero exposure to VPA and its possible association with an increased teratological risk has been discussed in the literature since the 1980s, when the first case reports emerged documenting children with a specific constellation of malformations following exposure to VPA (Ardinger 1988; DiLiberti 1984). Larger cohorts such as EURAP 2018 and data from population‐based health records studies (e.g. Denmark Health Record Registers; Sweden Health Record Registers) as well as the pregnancy registries (Australian Epilepsy and Pregnancy Register; Kerala Epilepsy and Pregnancy Registry; North American Epilepsy and Pregnancy Register; UK and Ireland Epilepsy and Pregnancy Register) and observational studies (e.g. Meador 2006; Omtzigt 1992; Samren 1997) included here, have all provided evidence to confirm that VPA is a significant human teratogen which is associated with an increase is a variety of malformation types. Here, we reported on 3018 VPA‐exposed children from prospective cohort style studies and 1482 VPA‐exposed pregnancies from routine health record studies.

In the meta‐analyses reported here a consistent pattern emerged: children exposed to VPA were at an increased risk of both a higher overall major malformation risk and risk of specific malformations including neural tube, cardiac, oro‐facial cleft, craniofacial, skeletal and limb malformations. The prevalence of major malformation following exposure to VPA in the womb was 9.8% (95% CI 8.1 to 11.9) for cohort studies, with similar rates from routine health record studies (9.7%, 95% CI 7.1 to 13.4) and from EURAP 2018 (10.3, 95% CI 8.8 to 12.0%). Children exposed to VPA were at an increased risk of being born with a major malformation compared with both the children of women without epilepsy and the children of women with untreated epilepsy, with the risk difference being 7% and 6% compared with the respective control groups. Analysis of the risks associated with VPA treatment at the specific malformation level was limited by a lack of control data; however, children exposed to VPA remained at a significantly increased risk for neural tube, cardiac and skeletal malformations compared with control children.

In comparison to other ASMs, in the meta‐analyses reported here, children exposed to VPA were at an increased risk of major malformation compared with children exposed to CBZ, CZP, GBP, LEV, LTG, TPM, OXC, PB and PHT, with the ZNS group being non‐significant but too being small to make a reliable comparison. The increased risk associated with VPA exposure ranged from 4% to 9%, depending on the comparator ASM.

At the specific malformation level, children exposed to VPA were at an increased risk of neural tube malformation compared with the children exposed to CBZ, LEV, LTG and PHT. We did not note any increase in the specific malformation type analyses compared to children exposed to GBP, OXC, PB or TPM, but this is most likely due to limited data. However, we found an increased rate of cardiac malformation compared to CBZ, LEV, LTG, TPM, PHT and an equal cardiac risk in comparison to the increased risk for PB‐exposed children. Oro‐facial cleft and craniofacial malformations were also significantly more common in the children exposed to VPA compared with children exposed to CBZ, LEV and LTG. There was no difference in the rate of oro‐facial cleft or craniofacial malformations compared with TPM, PB or PHT, but these are found to carry their own risks of this malformation type (US Medicaid Registers). Finally, skeletal or limb malformations in children exposed to VPA compared with children exposed to CBZ, LEV, LTG or PHT were significantly higher. All specific malformation comparisons that the data compared with CZP, GBP, ZNS and OXC were too limited for conclusions to be made.

When weighing up the risks and benefits of VPA treatment, the effects of VPA on other developmental outcomes including the developing brain should also be considered when considering the level of risk posed by VPA. VPA exposure is now also recognised as a neurobehavioural teratogen, with implications for the future cognitive functioning of the exposed child (Bromley 2014), and an increased risk of neurodevelopmental disorders such as autistic spectrum disorders (Christensen 2013) and attention deficit hyperactivity disorder (Christensen 2019).

More than any other ASM, studies have reported dose associations with level of major malformation risk for VPA (Australian Epilepsy and Pregnancy Register; EURAP 2018; Fairgrieve 2000; Israeli Teratogen Service; Kaneko 1999; Lindhout 1992; Milan Study 1999; North American Epilepsy and Pregnancy Register; Samren 1997; UK and Ireland Epilepsy and Pregnancy Register). The largest data set with clear dose comparisons is the EURAP 2018 collaboration, which found that the prevalence of major congenital malformations increased from 6.3% at doses < 650 mg daily to 25.2% for doses ≥ 1450 mg daily. Interestingly, pregnancy registers have reported a decrease in the mean dose for new registrations (UK and Ireland Epilepsy and Pregnancy Register) and have noted that this is associated with a reduction in the number of observed cases of neural tube malformations and hypospadias (Australian Epilepsy and Pregnancy Register).

#### Zonisamide

Experience with ZNS exposure was limited to 130 cases described in four studies (Jimenez 2020; MONEAD 2020; North American Epilepsy and Pregnancy Register; UK and Ireland Epilepsy and Pregnancy Register), therefore, it is not possible to draw conclusions at this time. Further efforts are needed to develop experience with this medication in pregnancy, as it has been in use for a long period in certain parts of the world (Oommen 1999).

#### Other antiepileptic drugs

Either no, or very limited numbers, of pregnancies were found for other ASMs from the searches such as ethosuximide, sulthiame, perampanel, lacosamide or vigabatrin.

### Overall completeness and applicability of evidence

Efforts were made to ensure that the evidence presented here was as complete as possible by including the two dominant research study designs for this area of research; cohort study designs and datasets which contain routinely collected health records. However, we did not include case‐control congenital anomaly registers. In these registers, children are enrolled when the presence or absence of a malformation is known and, therefore, we classified recruitment as retrospective (e.g. Jentink 2010a; Jentink 2010b). Further, the nature of this data meant that it could not be directly combined into meta‐analysis with the data from the prospective observational studies. Additionally, in order to make the results of this review applicable to the treatment of women with epilepsy, included studies were required to include 70% or greater proportions of women taking ASMs for the treatment of epilepsy. This, however, will have reduced the sample size and may not be necessary. Whilst Christensen and colleagues (Denmark Health Record Registers) in 2021 found no difference in risk estimates in the children of women with epilepsy in comparison to the children born to women with other indications, Hernandez Diaz and colleagues, using US Medicaid Registers, derived data found that, in the context of TPM, the indication did alter the outcome reported. Further investigations are required to answer whether limiting this review to a high proportion of women with epilepsy is required.

Efforts were made to ensure that the most up‐to‐date information from the longitudinal research initiatives was utilised, which meant that we often had to take outcomes for different ASMs from a number of different papers, or that authors investigated malformation types separately over different papers, or published updates for certain ASMs only. The largest challenge in terms of the completeness of the evidence came from some studies not reporting specific monotherapy outcomes or reporting monotherapy and polytherapy outcomes for a particular ASM together (e.g. Richmond 2004; Sabers 2004). However, this appeared to be a more frequent finding with older studies and there was a noticeable trend regarding separate reporting for each ASM for monotherapy exposures.

The final challenge to the completeness of the data was in regard to the risk of specific types of malformations, due in a large part to the failure of included studies to publish specific malformation outcomes for all included groups. Whilst this is undoubtedly due to publication space, providing such information is critical for understanding the risks associated with specific malformation types. As demonstrated, in the case of PB or TPM, an ASM exposure may be associated with specific malformations, so reporting only an overall malformation figure may mask important associations. Further, unclear reporting and differences in the defining of certain malformation types or groups meant that we could not investigate hypospadias or gastrointestinal malformations, which have been linked to certain ASM exposures (EURAP 2018; Sweden Health Record Registers).

A few points of heterogeneity were found between included studies, which may limit the completeness of the evidence. Studies varied in how they dealt with the inclusion of foetal deaths or interruptions of pregnancy (with and without malformations) and in whether they counted genetic causes of malformation in their overall prevalence. At the outset of this review, we decided to use the author‐defined major malformation rate, as the review authors would be unlikely to have all the data required to determine information about reported major malformations. Considering this, however, we cannot confirm that all the studies applied the same criteria for classifying a major malformation. Further, there were differences between studies in the time at which the outcome was reported. For example, the UK and Ireland Epilepsy and Pregnancy Register has a major malformation reporting time before three months of age, whilst others included malformation presence at birth (e.g. Bozhinov 2009). Data from the EURAP 2018 collaboration and by Christensen and colleagues (2021) using Denmark Health Record Registers demonstrates that the reviewing of major malformation outcome at 12 months of age leads to an increased detection and, therefore, higher prevalence. Thus, data reported from some studies may in fact be an underestimation of the prevalence of major malformation if the assessment of the child occurs prior to 12 months of age.

Finally, major malformation risk is not the only outcome of importance in pregnancy exposures. Beyond the scope of this review, small‐for‐gestational‐age, prematurity, minor congential malformations as well as longer‐term child health and neurodevelopmental outcomes can be altered, with life impacting consequences (Bromley 2014; Clayton‐Smith 2019; Dean 2000) and, therefore, require consideration when understanding the total impact of an ASM exposure on the developing child. Minor malformations, for example, are an important part of the diagnostic criteria for foetal anticonvulsant syndromes, in particular (Clayton‐Smith 2019; Dean 2000) and their presence may lead to a more detailed physical examination to check for more severe physical symptoms of exposure or neurodevelopmental impairment. Neurodevelopmental impairments are also a more commonly occurring outcome in the general population and, therefore, will occur more frequently in the ASM‐exposed populations and can have a significant impact on quality of life (Bromley 2014; Clayton‐Smith 2019).

Strengths of this review update include the creation and advance publication of the review protocol, the clear inclusion criteria, extensive searches, the acquisition of unpublished data, the inclusion of articles not written in English, meta‐analysis for all possible comparisons, the consideration of specific as well as overall major malformation risk, the balance of both systematic reviewing and content expertise and the assessment of risk of bias and quality in the non‐randomised evidence. Further, we improved the quality of the meta‐analyses by stratifying by type of control group and importantly study design. The results across the different study types were summarised in meta‐analysis separately due to the potential overlap in the cases (e.g. a national epilepsy and pregnancy register may contain the same children with a malformation as a population dataset which utilised routine health records for that same region or population). Further, at the start of this review, there were concerns about likely heterogeneity coming from different measurement approaches, periods of follow‐up and different patterns of maternal indications. However, in comparisons with larger numbers of included exposed pregnancies, the prevalences were similar (Table 3, Figure 3). We therefore take the view that cohort studies and studies utilising population level health records offer complimentary evidence which can be viewed as replicating the results of each other, to ensure evidence consistency across the total available data.

Under the Cochrane guidelines, this review will continue to be updated every two years, or following the publication of a significant amount of new data, to ensure it remains up‐to‐date which adds further strength.

### Quality of the evidence

The methodological quality for each individual study is displayed in the Risk of bias in included studies and in Figure 2. Randomised controlled trials are thought to be unethical in this area due to the permanence of potential adverse effects for the foetus. Gold standard evidence for this area would, therefore, comprise data coming from a recruitment approach with low selection bias, prospective follow‐up, blinded outcome assessment to a standardised protocol and statistical methods to limit the influence of confounding or mediating variables. Obtaining all of these features in a single study is difficult and different study designs have a different set of strengths and weaknesses.

The RoB ratings provided by an adaption of ROBINS‐I, for example, showed that the certain routine health record studies scored at a lower risk of bias than the cohort studies for risk of selection biases, yet the routine health record studies were at higher risk for outcome measurement which was completed in a non‐standardised manner by clinicians who were not blinded to the ASM exposure of the child. To balance these strengths and weaknesses which are inherent within these study designs, a complimentary set of pharmacovigilance approaches are required in order to have an accurate understanding of the data pertaining to possible risk associated with ASM exposures.

It should be considered that ROBINS‐I is not optimised for pregnancy pharmacovigilance studies where the person taking the medication (mother) is not the person in which the outcome in is being assessed (child) and it was challenging to adopt the signalling questions and ratings to function for this review. Further, the recommended GRADE framework for rating the certainty of evidence was not used, as it would produce differential ratings depending on whether there were differences between the medications or not. For reviews of pregnancy pharmacovigilance data, bespoke risk of bias and certainty of evidence tools are required.

In conclusion, our risk of bias review indicates that, across the included studies, there are a number of important biases assessed as high risk which should be taken into account when interpreting the results. The biases, however, were thought to be balanced across the ASMs investigated and, therefore, it is not felt that the findings were due solely to these biases.

### Potential biases in the review process

Review authors RB and JCS were authors on three included studies (Mawer 2010; Meador 2006; UK and Ireland Epilepsy and Pregnancy Register) and author JC on one (Denmark Health Record Registers). This potential bias was reduced by delegating data extraction and risk of bias assessments to other review authors. The ROBINS‐I adaptation, all analyses and interpretation were provided to all authors for review and input.

### Agreements and disagreements with other studies or reviews

Despite many review articles in this area, there are few systematic reviews where meta‐analysis has been conducted and, where they have been completed, there are variations in study methodology (i.e. inclusion criteria). For example, the reviews by Veroniki 2017 and Meador 2008, included both prospective and retrospective studies, studies using population‐based electronic healthcare records, and data from case‐control studies. Whilst such a wide inclusion criteria led to increased numbers of included pregnancies within the meta‐analysis, the comparability of data from these different methodological types is unclear. Charlton 2008, for example, had demonstrated different rates of malformations from the UK Clinical Research Practice Database in comparison to the UK and Ireland Epilepsy and Pregnancy Register. Further, combining data from population studies using healthcare records with national epilepsy and pregnancy registers may lead to cases being represented twice; which, for rare outcomes, could alter the analyses significantly. We took a more cautious approach and did not combine data from cohort studies with data from studies that used population‐level routine health records. Whilst our findings were comparable to the more recent Veroniki 2017 review in regard to VPA, PTM, PB, PHT, and CBZ, we did not have enough data to investigate their finding that ethosuximide is associated with an increased risk of major malformation. Overall, our approach of reviewing and undertaking meta‐analysis separately for primary and secondary data sources provides internal comparison and validation of the results which, we feel, is a strength.

Further consistent findings were reported by Jentink 2010b who found the prevalence of malformation following CBZ to be 3.3% based on 2680 CBZ children from eight studies. In contrast to our review, however, Jentink 2010b found a significant association between CBZ exposure and spina bifida. However, as in our review, Jentink 2010a found that eight studies (N = 1565 pregnancies) showed a prevalence rate of 7.5% (95% CI 6.3 to 9.0) in those exposed to VPA, and noted an increase in terms of specific malformations. The data reported here pertaining to LEV is consistent with a previous systematic review (Chaudhry 2014), which also included the three prospective studies reported here (Australian Epilepsy and Pregnancy Register; North American Epilepsy and Pregnancy Register; UK and Ireland Epilepsy and Pregnancy Register) as well as studies utilising other methodologies and reported a prevalence rate of 2.2% (27/1213, 95% CI 1.53 to 3.22).

This updated meta‐analysis did not consistently replicate the reported association between TPM exposure and oral clefts, but we did narratively review data from the large US Medicaid Registers study, which reported an association. In a previously completed meta‐analysis, Alsaad 2015 had wider inclusion criteria which included 3420 patients taking TPM (mixed aetiologies and study design types) and 1,204,981 controls and reported a significant odds ratio (OR 6.26, 95% CI: 3.13 to 12.51). As noted throughout this discussion, data were limited pertaining to the newer ASMs and by the reporting of specific malformations in included studies, therefore, it is possible that the limited data that contributed to this meta‐analysis do not consistently uphold this association across all comparisons.

## Authors' conclusions

**Implications for practice**

There is consistent evidence, across different study designs, that prenatal exposure to VPA increases the risk of having a child with a major malformation with the risk including neural tube, cardiac, skeletal, limb, oro‐facial cleft and craniofacial malformations. Whilst the prevalence of major malformation is 9.8%, this outcome is only one of a constellation of symptoms associated with VPA exposure in utero (Clayton‐Smith 2019; Dean 2000; Yerby 1992) and which constitute the condition, foetal valproate spectrum disorder (ICD 11 LD2F.03) (Clayton‐Smith 2019). The impact of VPA on the developing foetus is clearly dose‐related (EURAP 2018) and this should be considered when counselling regarding the risks associated with in utero exposure to VPA. The evidence reported here therefore supports regulatory limitations on VPA’s use, unless clinically necessary, to treat maternal epilepsy (NICE 2022) and where clear counselling has been given to the patient. There are other ASMs, however, which also require careful patient counselling and these include CBZ, PB, TPM and PHT.

The increased data included in this review update did not alter the previous findings which suggested no increased risk of major malformation for children exposed to either LTG or LEV in utero compared with either control group across the different study types. There is more limited information on LEV exposure and specific malformation outcomes, however. For all other ASMs, the data are limited, and more data are required before conclusions can be drawn for either an overall major malformation risk or for specific malformation types. Further, it is now clear that the dose of ASM is a key component to major malformation risk for non‐VPA ASMS also. CBZ, PB and even LTG have demonstrated such an association when cohorts are adequately powered. For other ASMs, including LEV, the data are limited at present to inform reliably on malformation risks at higher doses. The EURAP 2018 collaboration has the largest dataset stratified by dose of ASM currently. This lack of limited data for specific doses should be openly discussed with women planning a pregnancy or who are in the childbearing years and an absence of data should not imply a lack of risk.

Given the variance in major malformation risk associated with individual ASM treatments and at different doses, preconceptual counselling should be tailored to the individual patient. Although traditional counselling has been that 90% of children born to women with epilepsy have healthy children, this oversimplifies a complex set of data. The ASM type, but also dose and considerations regarding specific malformation types, should also be central to counselling. It is also important to note that major malformation risk is just one aspect and that minor malformations and longer‐term child health and neurodevelopmental outcome risks should also feature in counselling.

Finally, every effort should be made by clinicians to inform women about local initiatives collecting data on ASM use in pregnancy and child malformation outcomes to improve the availability of evidence on which to base treatment decisions. Epilepsy and pregnancy registers have made a large contribution to the available dataset, but this is only possible with the support of referring clinicians and the women who participate.

**Implications for research**

**Implications for research and pharmacovigilance**

There is an obvious delay between the approval of a medication for use and obtaining comprehensive evidence regarding the potential major malformation risk. Some delay is inevitable, however, a longer delay than necessary will limit evidence‐based decision‐making regarding optimising the treatment of maternal epilepsy whilst limiting potential foetal risk. A failure to document the first few years’ worth of pregnancies to women on newer medications delays knowledge acquisition and new ASMs use in women of childbearing age may be unnecessarily avoided for longer than required. There are numerous medications approved for the treatment of epilepsy around the world, yet we see many without data at this time. The emergence of population level datasets using routine health record databases will likely have a positive impact on this latency, due to their automatic inclusion of large populations (Denmark Health Record Registers). Whilst low in participation selection bias, utilising routine healthcare data has reduced measurement sensitivity though, and disease pregnancy and epilepsy registers or clinical studies which employ blinded, standardised review of the malformation outcome offer a more sensitive approach to outcome measurement. The pharmacovigilance strategy for the ASMs, therefore, should actively include different study designs which balance each other's methodological areas of strength and weakness to form a reliable and comprehensive evidence base.

The RoB ratings highlight the issue that within‐study methodological improvements are required. Few studies, for example, report on how the major malformation was assessed and determined to be major or minor and whether this was done blinded to the ASM history, despite this being the primary study outcome. Therefore, an easily adopted improvement for research is to encourage the use of blinded, standardised assessments of the physical outcomes and use standardised classification approaches, such as those used by clinical geneticists, including the Human Phenotype Ontology (HPO) (http:// human‐phenotype‐ontology.github.io/about.html) to allow for more accurate comparison across studies.

Whilst research methodologies have become more refined over the years, for example, by reporting individual ASM types, rather than a single monotherapy group or recognising the importance of ASM dose, there are still several limitations in the approach to data reporting. The provision of an overall major malformation risk figure, for example, is unlikely to be reliable, as demonstrated for PB and TPM, and future data collection and analysis should implement automatic reporting at the specific malformation level, including this as supplementary information. To improve the data at this finer level, initiatives will require large cohorts and, therefore, there should be a movement towards standardised protocols and procedural alignment across research initiatives to allow for large enough datasets regarding specific malformation types for specific ASMs and specific doses.

Further investigations are also required into the factors which may modify the major malformation risk. This includes further consideration regarding folate supplementation and regarding the optimal dose for women with epilepsy. As cohorts increase in size, more nuanced investigations into dose associations are required by specific malformation types and future work should also consider any family risk factors. Observations have shown that some women who take ASMs, even at a very low dose, appear to be at higher risk of having a child with an ASM‐associated malformation. Further research focusing on identification of genomic variants which might modify how different women metabolise ASMs is crucial so that those who may be at higher risk of having a child with a major malformation, even when taking a lower dose of a specific ASM, can be identified and ASM treatment selected accordingly. Whilst this has proven difficult in the past, whole exome/genome sequencing, with careful selection of individuals for testing, is likely to make this more achievable (Ku 2011).

Finally, longitudinal work which also investigates the longer‐term health outcomes of children with ASM exposures should be undertaken to understand the true impact. Where possible, research initiatives which recruit pregnant women with epilepsy for the purpose of investigating major malformation outcomes should also seek to, where possible, utilise these populations to understand child health and neurodevelopmental outcomes.

## What's new

**Table:** 

**Date**	**Event**	**Description**
24 August 2023	New search has been performed	Searches updated 17 February 2022; 17 new studies have been included.
24 August 2023	New citation required but conclusions have not changed	Conclusions are unchanged.

## History

Protocol first published: Issue 11, 2012 Review first published: Issue 11, 2016

**Table:** 

**Date**	**Event**	**Description**
26 April 2017	Amended	Declarations of interest section updated.

## Appendices

### Appendix 1. CRS Web search strategy

1. MeSH DESCRIPTOR Pregnancy Explode All AND INSEGMENT

2. MeSH DESCRIPTOR Pregnancy Complications Explode All AND INSEGMENT

3. MeSH DESCRIPTOR Prenatal Exposure Delayed Effects Explode All AND INSEGMENT

4. fetal or foetal or fetus or foetus or prenatal or pregnant or pregnanc* AND INSEGMENT

5. newborn or infant AND INSEGMENT

6. MeSH DESCRIPTOR Teratogens Explode All AND INSEGMENT

7. teratogen* AND INSEGMENT

8. in NEXT utero AND INSEGMENT

9. "intra uterine" or intrauterine AND INSEGMENT

10. MeSH DESCRIPTOR Fetal Development Explode All AND INSEGMENT

11. MeSH DESCRIPTOR Infant, Newborn Explode All AND INSEGMENT

12. #1 OR #2 OR #3 OR #4 OR #5 OR #6 OR #7 OR #8 OR #9 OR #10 OR #11 AND INSEGMENT

13. MESH DESCRIPTOR Congenital Abnormalities EXPLODE ALL AND INSEGMENT

14. congenital NEAR2 defec* AND INSEGMENT

15. congenital NEAR2 malformation* AND INSEGMENT

16. congenital NEAR2 (anomal* or abnormal*) AND INSEGMENT

17. birth NEAR2 defec* AND INSEGMENT

18. minor NEAR2 (anomal* or abnormal* or malformation*) AND INSEGMENT

19. dysmorph* AND INSEGMENT

20. neural tube AND INSEGMENT

21. (cardiac or cardiovasc*) NEAR2 (defec* or malformation* or anomal* or abnormal*) AND INSEGMENT

22. (orofac* or craniofac*) NEAR2 (defec* or malformation* or anomal* or abnormal*) AND INSEGMENT

23. (skelet* or limb* or hip* or joint*) NEAR2 (defec* or malformation* or anomal* or abnormal*) AND INSEGMENT

24. talipes AND INSEGMENT

25. (eye* or ear* or nose* or nasal or nostril or mouth or lip*) NEAR2 (defec* or malformation* or anomal* or abnormal*) AND INSEGMENT

26. (epicanth* NEXT fold*) or hypertelorism* AND INSEGMENT

27. philtrum or microstomia AND INSEGMENT

28. (digit* or finger* or toe* or nail*) NEAR2 (defec* or malformation* or anomal* or abnormal*) AND INSEGMENT

29. hypoplasia or arachnodactyly AND INSEGMENT

30. hernia* or sacral dimple* AND INSEGMENT

31. #13 OR #14 OR #15 OR #16 OR #17 OR #18 OR #19 OR #20 OR #21 OR #22 OR #23 OR #24 OR #25 OR #26 OR #27 OR #28 OR #29 OR #30 AND INSEGMENT

32. #12 AND #31 AND INSEGMENT

33. >14/09/2015:CRSCREATED AND INSEGMENT

34. #32 AND #33 AND INSEGMENT

35. MeSH DESCRIPTOR Pregnancy Explode All AND CENTRAL:TARGET

36. MeSH DESCRIPTOR Pregnancy Complications Explode All AND CENTRAL:TARGET

37. MeSH DESCRIPTOR Prenatal Exposure Delayed Effects Explode All AND CENTRAL:TARGET

38. fetal or foetal or fetus or foetus or prenatal or pregnant or pregnanc* AND CENTRAL:TARGET

39. newborn or infant AND CENTRAL:TARGET

40. MeSH DESCRIPTOR Teratogens Explode All AND CENTRAL:TARGET

41. teratogen* AND CENTRAL:TARGET

42. in NEXT utero AND CENTRAL:TARGET

43. "intra uterine" or intrauterine AND CENTRAL:TARGET

44. MeSH DESCRIPTOR Fetal Development Explode All AND CENTRAL:TARGET

45. MeSH DESCRIPTOR Infant, Newborn Explode All AND CENTRAL:TARGET

46. #35 OR #36 OR #37 OR #38 OR #39 OR #40 OR #41 OR #42 OR #43 OR #44 OR #45 AND CENTRAL:TARGET

47. MESH DESCRIPTOR Congenital Abnormalities EXPLODE ALL AND CENTRAL:TARGET

48. congenital NEAR2 defec* AND CENTRAL:TARGET

49. congenital NEAR2 malformation* AND CENTRAL:TARGET

50. congenital NEAR2 (anomal* or abnormal*) AND CENTRAL:TARGET

51. birth NEAR2 defec* AND CENTRAL:TARGET

52. minor NEAR2 (anomal* or abnormal* or malformation*) AND CENTRAL:TARGET

53. dysmorph* AND CENTRAL:TARGET

54. neural tube AND CENTRAL:TARGET

55. (cardiac or cardiovasc*) NEAR2 (defec* or malformation* or anomal* or abnormal*) AND CENTRAL:TARGET

56. (orofac* or craniofac*) NEAR2 (defec* or malformation* or anomal* or abnormal*) AND CENTRAL:TARGET

57. (skelet* or limb* or hip* or joint*) NEAR2 (defec* or malformation* or anomal* or abnormal*) AND CENTRAL:TARGET

58. talipes AND CENTRAL:TARGET

59. (eye* or ear* or nose* or nasal or nostril or mouth or lip*) NEAR2 (defec* or malformation* or anomal* or abnormal*) AND CENTRAL:TARGET

60. (epicanth* NEXT fold*) or hypertelorism* AND CENTRAL:TARGET

61. philtrum or microstomia AND CENTRAL:TARGET

62. (digit* or finger* or toe* or nail*) NEAR2 (defec* or malformation* or anomal* or abnormal*) AND CENTRAL:TARGET

63. hypoplasia or arachnodactyly AND CENTRAL:TARGET

64. hernia* or sacral dimple* AND CENTRAL:TARGET

65. #47 OR #48 OR #49 OR #50 OR #51 OR #52 OR #53 OR #54 OR #55 OR #56 OR #57 OR #58 OR #59 OR #60 OR #61 OR #62 OR #63 OR #64 AND CENTRAL:TARGET

66. MeSH DESCRIPTOR Epilepsy Explode All WITH QUALIFIER DT AND CENTRAL:TARGET

67. MESH DESCRIPTOR Seizures EXPLODE ALL WITH QUALIFIER DT AND CENTRAL:TARGET

68. MeSH DESCRIPTOR Anticonvulsants Explode All AND CENTRAL:TARGET

69. (antiepilep* or anti‐epilep* or anticonvulsant* or anti‐convulsant* or antiseizure* or anti‐seizure* or AED or AEDs):AB,KW,KY,MC,MH,TI AND CENTRAL:TARGET

70. #66 OR #67 OR #68 OR #69 AND CENTRAL:TARGET

71. MeSH DESCRIPTOR Midazolam Explode All AND CENTRAL:TARGET

72. (Dalam OR Dormicum OR Dormire OR Epistatus OR Fulsed OR Garen OR Hypnovel OR Ipnovel OR Midazolam* OR Nocturna OR Setam OR Terap OR Versed):AB,KW,KY,MC,MH,TI AND CENTRAL:TARGET

73. #71 OR #72 AND CENTRAL:TARGET

74. MeSH DESCRIPTOR Methazolamide Explode All AND CENTRAL:TARGET

75. (Methazolamid* OR Methylacetazolamide OR Neptazane):AB,KW,KY,MC,MH,TI AND CENTRAL:TARGET

76. #74 OR #75 AND CENTRAL:TARGET

77. MeSH DESCRIPTOR Propofol Explode All AND CENTRAL:TARGET

78. (Anepol OR Diprivan OR Disoprivan OR Disoprofol OR Fresofol OR Hypro OR Lipuro OR Plofed OR Profol OR Propofil OR Propofol* OR Propolipid OR Propovan OR Propoven OR Provive OR Recofol):AB,KW,KY,MC,MH,TI AND CENTRAL:TARGET

79. #77 OR #78 AND CENTRAL:TARGET

80. MeSH DESCRIPTOR Temazepam Explode All AND CENTRAL:TARGET

81. (Dasuen OR Euhypnos OR Hydroxydiazepam OR Levanxol OR Methyloxazepam OR Nocturne OR Norkotral OR Normison OR Normitab OR Nortem OR Oxydiazepam OR Planum OR Pronervon OR Remestan OR Restoril OR Signopam OR Temaze OR Temazep* OR Temtabs OR Tenox):AB,KW,KY,MC,MH,TI AND CENTRAL:TARGET

82. #80 OR #81 AND CENTRAL:TARGET

83. MeSH DESCRIPTOR Thiopental Explode All AND CENTRAL:TARGET

84. (Bomathal OR Farmotal OR Nesdonal OR Penthiobarbit* OR Pentothal OR Sodipental OR Thiomebumal OR Thionembutal OR Thiopent* OR Tiobarbital OR Tiopental* OR Trapanal):AB,KW,KY,MC,MH,TI AND CENTRAL:TARGET

85. #83 OR #84 AND CENTRAL:TARGET

86. #70 OR #73 OR #76 OR #79 OR #82 OR #85 AND CENTRAL:TARGET

87. (Acemit OR Acetamide OR Acetazolamid* OR Avva OR Azm OR Azol OR Diacarb OR Diamox OR Diazomid OR Diluran OR Edemox OR Glaupax):AB,KW,KY,MC,MH,TI AND CENTRAL:TARGET

88. (Barbexaclon*):AB,KW,KY,MC,MH,TI AND CENTRAL:TARGET

89. (Beclamid* OR Chloracon OR Hibicon OR Posedrine OR Nydrane OR Seclar):AB,KW,KY,MC,MH,TI AND CENTRAL:TARGET

90. (Brivaracetam*):AB,KW,KY,MC,MH,TI AND CENTRAL:TARGET

91. (Bromide*):AB,KW,KY,MC,MH,TI AND CENTRAL:TARGET

92. (Carbamazepin* OR Carbamazepen* OR Carbamezepin* OR CBZ OR SPD417 OR "Apo‐Carbamazepine" OR Atretol OR Biston OR Calepsin OR Carbagen OR Carbatrol OR Carbazepin* OR Carbelan OR Epitol OR Equetro OR Finlepsin OR Karbamazepin OR Lexin OR Neurotop OR "Novo‐Carbamaz" OR "Nu‐Carbamazepine" OR Sirtal OR Stazepin* OR "Taro‐Carbamazepine" OR Tegretal OR Tegretol OR Telesmin OR Teril OR Timonil):AB,KW,KY,MC,MH,TI AND CENTRAL:TARGET

93. (Carisbamat* OR Comfyde OR "RWJ‐333369" OR "YKP 509"):AB,KW,KY,MC,MH,TI AND CENTRAL:TARGET

94. (Cenobamat* OR Xcopri OR YKP3089):AB,KW,KY,MC,MH,TI AND CENTRAL:TARGET

95. (Chlormethiazol* OR Distraneurin):AB,KW,KY,MC,MH,TI AND CENTRAL:TARGET

96. (Aedon OR Anxirloc OR Castilium OR Chlorepin OR Clarmyl OR Clobam OR Clobamax OR Clobator OR Clobazam* OR Clofritis OR Clopax OR Clorepin OR Frisium OR Grifoclobam OR Karidium OR Lucium OR Mystan OR Noiafren OR Onfi OR Sederlona OR Sentil OR Urbadan OR Urbanil OR Urbanol OR Urbanyl):AB,KW,KY,MC,MH,TI AND CENTRAL:TARGET

97. (Antelepsin OR Antilepsin OR Chlonazepam OR Cloazepam OR Clonazepam* OR Clonex OR Clonopin OR Iktorivil OR Klonopin OR Kriadex OR Landsen OR Paxam OR Petril OR Ravotril OR Rivatril OR Rivotril OR “ro 5‐4023” OR “ro 54023”):AB,KW,KY,MC,MH,TI AND CENTRAL:TARGET

98. (Calner OR Clorazepat* OR Justum OR Mendon OR "Novo‐Clopate" OR Tranxene OR Tranxilium):AB,KW,KY,MC,MH,TI AND CENTRAL:TARGET

99. (Diapam OR Diastat OR Diazemuls OR Diazepam* OR Nervium OR Relanium OR Valium):AB,KW,KY,MC,MH,TI AND CENTRAL:TARGET

100. (Dimethadion* OR Dimethyloxazolidinedione):AB,KW,KY,MC,MH,TI AND CENTRAL:TARGET

101. (Eslicarbazepin* OR Exalief OR Stedesa OR Zebinix):AB,KW,KY,MC,MH,TI AND CENTRAL:TARGET

102. (Esilgan OR Estazolam* OR Eurodin OR Nuctalon OR Prosom OR Tasedan):AB,KW,KY,MC,MH,TI AND CENTRAL:TARGET

103. (Ethadion*):AB,KW,KY,MC,MH,TI AND CENTRAL:TARGET

104. (Aethosuximid* OR Emeside OR Ethosucci* OR Ethosuxide OR Ethosuximid* OR Etosuximid* OR Zarontin):AB,KW,KY,MC,MH,TI AND CENTRAL:TARGET

105. (Ethotoin* OR Peganone):AB,KW,KY,MC,MH,TI AND CENTRAL:TARGET

106. (Felbamat* OR Felbatol OR Felbamyl OR Taloxa):AB,KW,KY,MC,MH,TI AND CENTRAL:TARGET

107. (Flunarizin* OR Sibelium):AB,KW,KY,MC,MH,TI AND CENTRAL:TARGET

108. (Cerebyx OR Fosphenytoin* OR Prodilantin):AB,KW,KY,MC,MH,TI AND CENTRAL:TARGET

109. (Gabapentin* OR Aclonium OR Fanatrex OR Gabapetin OR Gabarone OR GBP OR Gralise OR Neogab OR Neurontin OR "Novo‐Gabapentin" OR Nupentin):AB,KW,KY,MC,MH,TI AND CENTRAL:TARGET

110. ("CCD‐1042" OR Ganaxolon*):AB,KW,KY,MC,MH,TI AND CENTRAL:TARGET

111. (Erlosamide OR Harkoseride OR Lacosamid* OR Vimpat):AB,KW,KY,MC,MH,TI AND CENTRAL:TARGET

112. (Lamotrigin* OR Elmendos OR Epilepax OR "GW 273293" OR Lamictal OR Lamictin OR Lamitor OR Lamitrin OR Lamogine OR Lamotrine OR LTG):AB,KW,KY,MC,MH,TI AND CENTRAL:TARGET

113. (Levetiracetam* OR Keppra OR LEV OR Levitiracetam):AB,KW,KY,MC,MH,TI AND CENTRAL:TARGET

114. (Ativan OR Intensl OR Loraz OR Lorazepam* OR Lormetazepam* OR Temesta):AB,KW,KY,MC,MH,TI AND CENTRAL:TARGET

115. (Losigamon*):AB,KW,KY,MC,MH,TI AND CENTRAL:TARGET

116. ("Magnesium sulfat*" OR "Magnesium sulphat*"):AB,KW,KY,MC,MH,TI AND CENTRAL:TARGET

117. (Medazepam* OR Nobrium OR Rudotel OR Rusedal):AB,KW,KY,MC,MH,TI AND CENTRAL:TARGET

118. (Mephenytoin* OR Mesantoin):AB,KW,KY,MC,MH,TI AND CENTRAL:TARGET

119. (Dapaz OR Equanil OR Meprobamat* OR Meprospan OR Miltown OR Tranmep OR Visano):AB,KW,KY,MC,MH,TI AND CENTRAL:TARGET

120. (Celontin OR Mesuximid* OR Methsuximide OR Petinutin):AB,KW,KY,MC,MH,TI AND CENTRAL:TARGET

121. (Mephobarbit* OR Mebaral OR Mephyltaletten OR Methylphenobarbit* OR Metilfenobarbital OR Phemiton OR Prominal):AB,KW,KY,MC,MH,TI AND CENTRAL:TARGET

122. (Erimin OR Nimetazepam*):AB,KW,KY,MC,MH,TI AND CENTRAL:TARGET

123. (Alodorm OR Arem OR Insoma OR Mogadon OR Nitrados OR Nitrazadon OR Nitrazepam* OR Ormodon OR Paxadorm OR Remnos OR Somnite OR Pacisyn):AB,KW,KY,MC,MH,TI AND CENTRAL:TARGET

124. (Oxcarbazepin* OR Actinium OR Barzepin OR Carbox OR Deprectal OR "GP 47680" OR Lonazet OR OCBZ OR Oxalepsy OR OXC OR Oxcarbamazepine OR Oxetol OR Oxpin OR Oxrate OR Oxtellar OR Oxypine OR Pharozepine OR Prolepsi OR Timox OR Trexapin OR Trileptal OR Trileptin):AB,KW,KY,MC,MH,TI AND CENTRAL:TARGET

125. (Paraldehyd*):AB,KW,KY,MC,MH,TI AND CENTRAL:TARGET

126. (Paramethadion*):AB,KW,KY,MC,MH,TI AND CENTRAL:TARGET

127. (E2007 OR Fycompa OR Perampanel*):AB,KW,KY,MC,MH,TI AND CENTRAL:TARGET

128. (Phenacemid*):AB,KW,KY,MC,MH,TI AND CENTRAL:TARGET

129. (Ethylphenacemid* OR Pheneturid*):AB,KW,KY,MC,MH,TI AND CENTRAL:TARGET

130. (Adonal OR Aephenal OR Agrypnal OR Amylofene OR Aphenylbarbit OR Aphenyletten OR Barbenyl OR Barbinal OR Barbiphen* OR Barbipil OR Barbita OR Barbivis OR Barbonal OR Barbophen OR Bardorm OR Bartol OR Bialminal OR "Blu‐Phen" OR Cabronal OR Calmetten OR Calminal OR Cardenal OR Chinoin OR Codibarbita OR Coronaletta OR Cratecil OR Damoral OR Dezibarbitur OR Dormina OR Dormiral OR Dormital OR Doscalun OR Duneryl OR Ensobarb OR Ensodorm OR Epanal OR Epidorm OR Epilol OR Episedal OR Epsylone OR Eskabarb OR Etilfen OR Euneryl OR Fenbital OR Fenemal OR Fenobarbital OR Fenosed OR Fenylettae OR Gardenal OR Gardepanyl OR Glysoletten OR Haplopan OR Haplos OR Helional OR Hennoletten OR Henotal OR Hypnaletten OR Hypnette OR "Hypno‐Tablinetten" OR Hypnogen OR Hypnolone OR Hypnoltol OR Hysteps OR Lefebar OR Leonal OR Lephebar OR Lepinal OR Lepinaletten OR Linasen OR Liquital OR Lixophen OR Lubergal OR Lubrokal OR Lumen OR Lumesettes OR Lumesyn OR Luminal OR Lumofridetten OR Luphenil OR Luramin OR Molinal OR Neurobarb OR Nirvonal OR Noptil OR "Nova‐Pheno" OR Nunol OR Parkotal OR PB OR Pharmetten OR "Phen‐Bar" OR Phenaemal OR Phenemal* OR Phenobal OR Phenobarbit* OR Phenobarbyl OR Phenoluric OR Phenolurio OR Phenomet OR Phenonyl OR Phenoturic OR Phenylethylbarbit* OR Phenylethylmalonylurea OR Phenyletten OR Phenyral OR Phob OR Polcominal OR Prominal OR Promptonal OR "Seda‐Tablinen" OR Sedabar OR Sedicat OR Sedizorin OR Sedlyn OR Sedofen OR Sedonal OR Sedonettes OR Sevenal OR Sinoratox OR Solfoton OR "Solu‐Barb" OR Sombutol OR Somnolens OR Somnoletten OR Somnosan OR Somonal OR Spasepilin OR Starifen OR Starilettae OR Stental OR Talpheno OR Teolaxin OR Teoloxin OR Thenobarbital OR Theoloxin OR Triabarb OR Tridezibarbitur OR Triphenatol OR Versomnal OR Zadoletten OR Zadonal):AB,KW,KY,MC,MH,TI AND CENTRAL:TARGET

131. (Phensuximid*):AB,KW,KY,MC,MH,TI AND CENTRAL:TARGET

132. (Aleviatin OR Antisacer OR Auranile OR Causoin OR Citrullamon OR Citrulliamon OR Comital OR Comitoina OR Convul OR Danten OR Dantinal OR Dantoin* OR Denyl OR "Di‐Hydan" OR "Di‐Lan" OR "Di‐Phetine" OR Didan OR Difenilhidantoin* OR Difenin OR Difetoin OR Difhydan OR Dihycon OR Dihydantoin OR Dilabid OR Dilantin* OR Dillantin OR Dintoin* OR Diphantoin OR Diphedal OR Diphedan OR Diphenat OR Diphenin* OR Diphentoin OR Diphentyn OR Diphenylan OR Diphenylhydantoin* OR Diphenylhydatanoin OR Ditoinate OR Ekko OR Elepsindon OR Enkelfel OR Epamin OR Epanutin OR Epasmir OR Epdantoin* OR Epelin OR Epifenyl OR Epihydan OR Epilan OR Epilantin OR Epinat OR Epised OR Eptal OR Eptoin OR Fenantoin OR Fenidantoin OR Fenitoin* OR Fentoin OR Fenylepsin OR Fenytoin* OR "Gerot‐epilan‐D" OR Hidan OR Hidant* OR Hindatal OR Hydant* OR Ictalis OR Idantoi* OR Iphenylhydantoin OR Kessodanten OR Labopal OR Lehydan OR Lepitoin OR Lepsin OR Mesantoin OR Minetoin OR "Neos‐Hidantoina" OR Neosidantoina OR Novantoina OR Novophenytoin OR "Om‐hidantoina" OR "Om‐Hydantoine" OR Oxylan OR Phanantin* OR Phenatine OR Phenatoine OR Phenhydan* OR Phenitoin OR Phentoin OR Phentytoin OR Phenytek OR Phenytex OR Phenytoin* OR PHT OR Ritmenal OR Saceril OR Sanepil OR Silantin OR Sinergina OR Sodanthon OR Sodanto* OR Solantin OR Solantoin OR Solantyl OR Sylantoic OR Tacosal OR Thilophenyl OR TOIN OR Zentronal OR Zentropil):AB,KW,KY,MC,MH,TI AND CENTRAL:TARGET

133. (Lyrica OR Pregabalin*):AB,KW,KY,MC,MH,TI AND CENTRAL:TARGET

134. (Mysoline OR Primidon* OR Sertan):AB,KW,KY,MC,MH,TI AND CENTRAL:TARGET

135. (Gabrene OR Garene OR Halogabide OR Halogenide OR Progabid*):AB,KW,KY,MC,MH,TI AND CENTRAL:TARGET

136. (Ecovia OR Remacemid*):AB,KW,KY,MC,MH,TI AND CENTRAL:TARGET

137. ("D‐23129" OR "D23129" OR EZG OR Ezogabin* OR Retigabin* OR RTG OR Trobalt OR Potiga):AB,KW,KY,MC,MH,TI AND CENTRAL:TARGET

138. (Rilutek OR Riluzol* OR Trifluoromethoxybenzothiazol*):AB,KW,KY,MC,MH,TI AND CENTRAL:TARGET

139. (Inovelon OR Rufinamid* OR Xilep):AB,KW,KY,MC,MH,TI AND CENTRAL:TARGET

140. (Seletracetam*):AB,KW,KY,MC,MH,TI AND CENTRAL:TARGET

141. (Diacomit OR Stiripentol*):AB,KW,KY,MC,MH,TI AND CENTRAL:TARGET

142. (Sulthiam* OR Sultiam* OR Ospolot):AB,KW,KY,MC,MH,TI AND CENTRAL:TARGET

143. (Talampanel*):AB,KW,KY,MC,MH,TI AND CENTRAL:TARGET

144. (Tiagabin* OR Gabitril):AB,KW,KY,MC,MH,TI AND CENTRAL:TARGET

145. (Tiletamin*):AB,KW,KY,MC,MH,TI AND CENTRAL:TARGET

146. (Topiramat* OR Qudexy OR Tipiramate OR Topamax OR "Topiramic acid" OR TPM):AB,KW,KY,MC,MH,TI AND CENTRAL:TARGET

147. (Tridione OR Trimethadion*):AB,KW,KY,MC,MH,TI AND CENTRAL:TARGET

148. (Valnoctamid*):AB,KW,KY,MC,MH,TI AND CENTRAL:TARGET

149. (Avugane OR Baceca OR Convulex OR Delepsine OR Depacon OR Depakene OR Depakine OR Depakote OR Deproic OR Divalprax OR Divalproex* OR DPA OR Encorate OR Epiject OR Epilex OR Epilim OR Episenta OR Epival OR Ergenyl OR Mylproin OR Orfiril OR Orlept OR Selenica OR Stavzor OR Valance OR Valcote OR Valparin OR Valpro* OR VPA OR Zalkote):AB,KW,KY,MC,MH,TI AND CENTRAL:TARGET

150. (Depamide OR Valpromid*):AB,KW,KY,MC,MH,TI AND CENTRAL:TARGET

151. (GVG OR Sabril OR Vigabatrin*):AB,KW,KY,MC,MH,TI AND CENTRAL:TARGET

152. (Zonisamid* OR Exceglan OR Excegram OR Excegran OR ZNS OR Zonegran):AB,KW,KY,MC,MH,TI AND CENTRAL:TARGET

153. #86 OR #87 OR #88 OR #89 OR #90 OR #91 OR #92 OR #93 OR #94 OR #95 OR #96 OR #97 OR #98 OR #99 OR #100 OR #101 OR #102 OR #103 OR #104 OR #105 OR #106 OR #107 OR #108 OR #109 OR #110 OR #111 OR #112 OR #113 OR #114 OR #115 OR #116 OR #117 OR #118 OR #119 OR #120 OR #121 OR #122 OR #123 OR #124 OR #125 OR #126 OR #127 OR #128 OR #129 OR #130 OR #131 OR #132 OR #133 OR #134 OR #135 OR #136 OR #137 OR #138 OR #139 OR #140 OR #141 OR #142 OR #143 OR #144 OR #145 OR #146 OR #147 OR #148 OR #149 OR #150 OR #151 OR #152

154. #46 AND #65 AND #153

155. >14/09/2015:CRSINCENTRAL AND CENTRAL:TARGET

156. #154 AND #155

157. #34 OR #156

### Appendix 2. MEDLINE search strategy

1. exp Pregnancy/

2. exp Pregnancy Complications/

3. exp Prenatal Exposure Delayed Effects/

4. (fetal or foetal or fetus or foetus or prenatal or pregnant or pregnanc$).mp.

5. (newborn or infant).mp.

6. exp Teratogens/

7. teratogen$.mp.

8. (in adj utero).mp.

9. (intra uterine or intrauterine).mp.

10. exp Fetal Development/

11. exp Infant, Newborn/

12. or/1‐11

13. exp Congenital Abnormalities/

14. (congenital adj2 defec$).tw.

15. (congenital adj2 malformation$).tw.

16. (congenital adj2 (anomal$ or abnormal$)).tw.

17. (birth adj defec$).tw.

18. (minor adj2 (anomal$ or abnormal$ or malformation$)).tw.

19. dysmorph$.tw.

20. neural tube.tw.

21. ((cardiac or cardiovasc$) adj2 (defec$ or malformation$ or anomal$ or abnormal$)).tw.

22. ((orofac$ or craniofac$) adj2 (defec$ or malformation$ or anomal$ or abnormal$)).tw.

23. ((skelet$ or limb$ or hip$ or joint$) adj2 (defec$ or malformation$ or anomal$ or abnormal$)).tw.

24. talipes.tw.

25. ((eye$ or ear$ or nose$ or nasal or nostril or mouth or lip$) adj2 (defec$ or malformation$ or anomal$ or abnormal$)).tw.

26. ((epicanth* adj fold*) or hypertelorism*).tw.

27. (philtrum or microstomia).tw.

28. ((digit$ or finger$ or toe$ or nail$) adj2 (defec$ or malformation$ or anomal$ or abnormal$)).tw.

29. (hypoplasia or arachnodactyly).tw.

30. (hernia* or sacral dimple*).tw.

31. or/13‐30

32. exp *Epilepsy/dt [Drug Therapy]

33. exp Seizures/dt [Drug Therapy]

34. exp Anticonvulsants/

35. (antiepilep$ or anti‐epilep$ or anticonvulsant$ or anti‐convulsant$ or antiseizure$ or anti‐seizure$ or AED or AEDs).mp.

36. exp Midazolam/

37. (Dalam or Dormicum or Dormire or Epistatus or Fulsed or Garen or Hypnovel or Ipnovel or Midazolam* or Nocturna or Setam or Terap or Versed).mp.

38. exp Methazolamide/

39. (Methazolamid* or Methylacetazolamide or Neptazane).mp.

40. exp Propofol/

41. (Anepol or Diprivan or Disoprivan or Disoprofol or Fresofol or Hypro or Lipuro or Plofed or Profol or Propofil or Propofol* or Propolipid or Propovan or Propoven or Provive or Recofol).mp.

42. exp Temazepam/

43. (Dasuen or Euhypnos or Hydroxydiazepam or Levanxol or Methyloxazepam or Nocturne or Norkotral or Normison or Normitab or Nortem or Oxydiazepam or Planum or Pronervon or Remestan or Restoril or Signopam or Temaze or Temazep* or Temtabs or Tenox).mp.

44. exp Thiopental/

45. (Bomathal or Farmotal or Nesdonal or Penthiobarbit* or Pentothal or Sodipental or Thiomebumal or Thionembutal or Thiopent* or Tiobarbital or Tiopental* or Trapanal).mp.

46. (Acemit or Acetamide or Acetazolamid* or Avva or Azm or Azol or Diacarb or Diamox or Diazomid or Diluran or Edemox or Glaupax).mp.

47. Barbexaclon*.mp.

48. (Beclamid* or Chloracon or Hibicon or Posedrine or Nydrane or Seclar).mp.

49. Brivaracetam*.mp.

50. Bromide*.mp.

51. (Carbamazepin* or Carbamazepen* or Carbamezepin* or CBZ or SPD417 or "Apo‐Carbamazepine" or Atretol or Biston or Calepsin or Carbagen or Carbatrol or Carbazepin* or Carbelan or Epitol or Equetro or Finlepsin or Karbamazepin or Lexin or Neurotop or "Novo‐Carbamaz" or "Nu‐Carbamazepine" or Sirtal or Stazepin* or "Taro‐Carbamazepine" or Tegretal or Tegretol or Telesmin or Teril or Timonil).mp.

52. (Carisbamat* or Comfyde or "RWJ‐333369" or "YKP 509").mp.

53. (cenobamat* or Xcopri or YKP3089).mp.

54. (Chlormethiazol* or Distraneurin).mp.

55. (Aedon or Anxirloc or Castilium or Chlorepin or Clarmyl or Clobam or Clobamax or Clobator or Clobazam* or Clofritis or Clopax or Clorepin or Frisium or Grifoclobam or Karidium or Lucium or Mystan or Noiafren or Onfi or Sederlona or Sentil or Urbadan or Urbanil or Urbanol or Urbanyl).mp.

56. (Antelepsin or Antilepsin or Chlonazepam or Cloazepam or Clonazepam* or Clonex or Clonopin or Iktorivil or Klonopin or Kriadex or Landsen or Paxam or Petril or Ravotril or Rivatril or Rivotril or "ro 5‐4023" or "ro 54023").mp.

57. (Calner or Clorazepat* or Justum or Mendon or "Novo‐Clopate" or Tranxene or Tranxilium).mp.

58. (Diapam or Diastat or Diazemuls or Diazepam* or Nervium or Relanium or Valium).mp.

59. (Dimethadion* or Dimethyloxazolidinedione).mp.

60. (Eslicarbazepin* or Exalief or Stedesa or Zebinix).mp.

61. (Esilgan or Estazolam* or Eurodin or Nuctalon or Prosom or Tasedan).mp.

62. Ethadion*.mp.

63. (Aethosuximid* or Emeside or Ethosucci* or Ethosuxide or Ethosuximid* or Etosuximid* or Zarontin).mp.

64. (Ethotoin* or Peganone).mp.

65. (Felbamat* or Felbatol or Felbamyl or Taloxa).mp.

66. (Flunarizin* or Sibelium).mp.

67. (Cerebyx or Fosphenytoin* or Prodilantin).mp.

68. (Gabapentin* or Aclonium or Fanatrex or Gabapetin or Gabarone or GBP or Gralise or Neogab or Neurontin or "Novo‐Gabapentin" or Nupentin).mp.

69. ("CCD‐1042" or Ganaxolon*).mp.

70. (Erlosamide or Harkoseride or Lacosamid* or Vimpat).mp.

71. (Lamotrigin* or Elmendos or Epilepax or "GW 273293" or Lamictal or Lamictin or Lamitor or Lamitrin or Lamogine or Lamotrine or LTG).mp.

72. (Levetiracetam* or Keppra or LEV or Levitiracetam).mp.

73. (Ativan or Intensl or Loraz or Lorazepam* or Lormetazepam* or Temesta).mp.

74. Losigamon*.mp.

75. ("Magnesium sulfat*" or "Magnesium sulphat*").mp.

76. (Medazepam* or Nobrium or Rudotel or Rusedal).mp.

77. (Mephenytoin* or Mesantoin).mp.

78. (Dapaz or Equanil or Meprobamat* or Meprospan or Miltown or Tranmep or Visano).mp.

79. (Celontin or Mesuximid* or Methsuximide or Petinutin).mp.

80. (Mephobarbit* or Mebaral or Mephyltaletten or Methylphenobarbit* or Metilfenobarbital or Phemiton or Prominal).mp.

81. (Erimin or Nimetazepam*).mp.

82. (Alodorm or Arem or Insoma or Mogadon or Nitrados or Nitrazadon or Nitrazepam* or Ormodon or Paxadorm or Remnos or Somnite or Pacisyn).mp.

83. (Oxcarbazepin* or Actinium or Barzepin or Carbox or Deprectal or "GP 47680" or Lonazet or OCBZ or Oxalepsy or OXC or Oxcarbamazepine or Oxetol or Oxpin or Oxrate or Oxtellar or Oxypine or Pharozepine or Prolepsi or Timox or Trexapin or Trileptal or Trileptin).mp.

84. Paraldehyd*.mp.

85. Paramethadion*.mp.

86. (E2007 or Fycompa or Perampanel*).mp.

87. Phenacemid*.mp.

88. (Ethylphenacemid* or Pheneturid*).mp.

89. (Adonal or Aephenal or Agrypnal or Amylofene or Aphenylbarbit or Aphenyletten or Barbenyl or Barbinal or Barbiphen* or Barbipil or Barbita or Barbivis or Barbonal or Barbophen or Bardorm or Bartol or Bialminal or "Blu‐Phen" or Cabronal or Calmetten or Calminal or Cardenal or Chinoin or Codibarbita or Coronaletta or Cratecil or Damoral or Dezibarbitur or Dormina or Dormiral or Dormital or Doscalun or Duneryl or Ensobarb or Ensodorm or Epanal or Epidorm or Epilol or Episedal or Epsylone or Eskabarb or Etilfen or Euneryl or Fenbital or Fenemal or Fenobarbital or Fenosed or Fenylettae or Gardenal or Gardepanyl or Glysoletten or Haplopan or Haplos or Helional or Hennoletten or Henotal or Hypnaletten or Hypnette or "Hypno‐Tablinetten" or Hypnogen or Hypnolone or Hypnoltol or Hysteps or Lefebar or Leonal or Lephebar or Lepinal or Lepinaletten or Linasen or Liquital or Lixophen or Lubergal or Lubrokal or Lumen or Lumesettes or Lumesyn or Luminal or Lumofridetten or Luphenil or Luramin or Molinal or Neurobarb or Nirvonal or Noptil or "Nova‐Pheno" or Nunol or Parkotal or PB or Pharmetten or "Phen‐Bar" or Phenaemal or Phenemal* or Phenobal or Phenobarbit* or Phenobarbyl or Phenoluric or Phenolurio or Phenomet or Phenonyl or Phenoturic or Phenylethylbarbit* or Phenylethylmalonylurea or Phenyletten or Phenyral or Phob or Polcominal or Prominal or Promptonal or "Seda‐Tablinen" or Sedabar or Sedicat or Sedizorin or Sedlyn or Sedofen or Sedonal or Sedonettes or Sevenal or Sinoratox or Solfoton or "Solu‐Barb" or Sombutol or Somnolens or Somnoletten or Somnosan or Somonal or Spasepilin or Starifen or Starilettae or Stental or Talpheno or Teolaxin or Teoloxin or Thenobarbital or Theoloxin or Triabarb or Tridezibarbitur or Triphenatol or Versomnal or Zadoletten or Zadonal).mp.

90. Phensuximid*.mp.

91. (Aleviatin or Antisacer or Auranile or Causoin or Citrullamon or Citrulliamon or Comital or Comitoina or Convul or Danten or Dantinal or Dantoin* or Denyl or "Di‐Hydan" or "Di‐Lan" or "Di‐Phetine" or Didan or Difenilhidantoin* or Difenin or Difetoin or Difhydan or Dihycon or Dihydantoin or Dilabid or Dilantin* or Dillantin or Dintoin* or Diphantoin or Diphedal or Diphedan or Diphenat or Diphenin* or Diphentoin or Diphentyn or Diphenylan or Diphenylhydantoin* or Diphenylhydatanoin or Ditoinate or Ekko or Elepsindon or Enkelfel or Epamin or Epanutin or Epasmir or Epdantoin* or Epelin or Epifenyl or Epihydan or Epilan or Epilantin or Epinat or Epised or Eptal or Eptoin or Fenantoin or Fenidantoin or Fenitoin* or Fentoin or Fenylepsin or Fenytoin* or "Gerot‐epilan‐D" or Hidan or Hidant* or Hindatal or Hydant* or Ictalis or Idantoi* or Iphenylhydantoin or Kessodanten or Labopal or Lehydan or Lepitoin or Lepsin or Mesantoin or Minetoin or "Neos‐Hidantoina" or Neosidantoina or Novantoina or Novophenytoin or "Om‐hidantoina" or "Om‐Hydantoine" or Oxylan or Phanantin* or Phenatine or Phenatoine or Phenhydan* or Phenitoin or Phentoin or Phentytoin or Phenytek or Phenytex or Phenytoin* or PHT or Ritmenal or Saceril or Sanepil or Silantin or Sinergina or Sodanthon or Sodanto* or Solantin or Solantoin or Solantyl or Sylantoic or Tacosal or Thilophenyl or TOIN or Zentronal or Zentropil).mp.

92. (Lyrica or Pregabalin*).mp.

93. (Mysoline or Primidon* or Sertan).mp.

94. (Gabrene or Garene or Halogabide or Halogenide or Progabid*).mp.

95. (Ecovia or Remacemid*).mp.

96. ("D‐23129" or "D23129" or EZG or Ezogabin* or Retigabin* or RTG or Trobalt or Potiga).mp.

97. (Rilutek or Riluzol* or Trifluoromethoxybenzothiazol*).mp.

98. (Inovelon or Rufinamid* or Xilep).mp.

99. Seletracetam*.mp.

100. (Diacomit or Stiripentol*).mp.

101. (Sulthiam* or Sultiam* or Ospolot).mp.

102. Talampanel*.mp.

103. (Tiagabin* or Gabitril).mp.

104. Tiletamin*.mp.

105. (Topiramat* or Qudexy or Tipiramate or Topamax or "Topiramic acid" or TPM).mp.

106. (Tridione or Trimethadion*).mp.

107. Valnoctamid*.mp.

108. (Avugane or Baceca or Convulex or Delepsine or Depacon or Depakene or Depakine or Depakote or Deproic or Divalprax or Divalproex$ or DPA or Encorate or Epiject or Epilex or Epilim or Episenta or Epival or Ergenyl or Mylproin or Orfiril or Orlept or Selenica or Stavzor or Valance or Valcote or Valparin or Valpro$ or VPA or Zalkote).mp.

109. (Depamide or Valpromid*).mp.

110. (GVG or Sabril or Vigabatrin*).mp.

111. (Zonisamid* or Exceglan or Excegram or Excegran or ZNS or Zonegran).mp.

112. or/32‐111

113. 12 and 31 and 112

114. exp animals/ not humans.sh.

115. (animal or animals or mouse or mice or murine or rat or rats or rodent or rodents or zebrafish).ti.

116. 114 or 115

117. 113 not 116

118. (case adj (report? or study or studies)).ti.

119. 117 not 118

120. limit 119 to ed=20150910‐20220217

121. 119 not (1$ or 2$).ed.

122. 121 and (2015$ or 2016$ or 2017$ or 2018$ or 2019$ or 2020$ or 2021$ or 2022$).dt.

123. 120 or 122

124. remove duplicates from 123

### Appendix 3. SCOPUS search strategy

(((((TITLE‐ABS‐KEY(fetal or foetal or fetus or foetus or prenatal or pregnant or pregnanc*)) OR (TITLE‐ABS‐KEY({in utero} OR "intra uterine" OR intrauterine)) OR (TITLE‐ABS‐KEY(newborn OR infant OR teratogen*))) AND ((TITLE‐ABS‐KEY(congenital W/2 (abnormal* OR defec* OR malformation* OR anomal*))) OR (TITLE‐ABS‐KEY(birth W/2 defec*)) OR (TITLE‐ABS‐KEY(minor W/2 (anomal* OR abnormal* OR malformation*))) OR (TITLE‐ABS‐KEY(dysmorph* OR "neural tube" OR talipes OR philtrum OR microstomia)) OR (TITLE‐ABS‐KEY(hypoplasia OR arachnodactyly OR hernia* or "sacral dimple*")) OR (TITLE‐ABS‐KEY((cardiac OR cardiovasc*) W/2 (defec* or malformation* or anomal* or abnormal*))) OR (TITLE‐ABS‐KEY((orofac* or craniofac*) W/2 (defec* or malformation* or anomal* or abnormal*))) OR (TITLE‐ABS‐KEY((skelet* or limb* or hip* or joint*) W/2 (defec* or malformation* or anomal* or abnormal*))) OR (TITLE‐ABS‐KEY((eye* or ear* or nose* or nasal or nostril or mouth or lip*) W/2 (defec* or malformation* or anomal* or abnormal*))) OR (TITLE‐ABS‐KEY((digit* or finger* or toe* or nail*) W/2 (defec* or malformation* or anomal* or abnormal*))) OR (TITLE‐ABS‐KEY("epicanth* fold*" OR hypertelorism*)))) AND (TITLE‐ABS‐KEY(antiepilep* or anti‐epilep* or anticonvuls* or anti‐convuls* or antiseizure* or anti‐seizure* or AED or AEDs or Acetazolamid* or Alodorm or Antilepsin or Arem or Ativan or Avugane or Baceca or Barbexaclon* or Beclamid* or Biston or Brivaracetam* or Bromide* or Carbagen or Carbamazepen* or Carbamazepin* or Carbatrol or Carisbamat* or CBZ or Celontin or Cenobamat* or Cerebyx or Chlonazepam or Chloracon or Chlormethiazol* or Cloazepam or Clobazam* or Clonazepam* or Clonex or Clonopin or Clorazepat* or Convulex or Delepsine or Depacon or Depak* or Depamide or Deproic or Desitin or Diacomit or Diamox or Diastat or Diazepam* or Dilantin* or Dimethadion* or Diphenin* or Diphenylhydantoin* or Divalpr* or Dormicum or DPA or Ecovia or Emeside or Encorate or Epanutin or Epiject or Epilepax or Epilex or Epilim or Episenta or Epitol or Epival or Eptoin or Equetro or Ergenyl or Erimin or Eslicarbazepin* or Estazolam* or Ethadion* or Ethosuximid* or Ethotoin* or Ethylphenacemide or Exalief or Exceglan or Excegram or Excegran or Ezogabin* or Fanatrex or Felbamat* or Felbatol or Fenitoin* or Fenytoin* or Flunarizin* or Fosphenytoin* or Frisium or Fycompa or Gabapentin* or Gabarone or Gabitril or Gabrene or Ganaxolon* or Garene or GBP or Gralise or GVG or Halogabide or Halogenide or Hibicon or Hypnovel or Iktorivil or Inovelon or Insoma or Intensl or Keppra or Klonopin or Kriadex or Lacosamid* or Lamict* or Lamitor or Lamitrin or Lamogine or Lamotrigin* or Lamotrine or Landsen or LEV or Levetiracetam* or Liskantin or Loraz or Lorazepam* or Losigamon* or LTG or Luminal or Lyrica or "Magnesium sulfat*" or "Magnesium sulphat*"or Mebaral or Medazepam* or Mephenytoin* or Mephobarbit* or Mephyltaletten or Meprobamat* or Mesantoin or Mesuximide or Methazolamid* or Methsuximid* or Methylphenobarbit* or Midazolam* or Mogadon or Mylepsinum or Mylproin or Mysoline or Neogab or Neptazane or Neurontin or Neurotop or Nimetazepam* or Nitrados or Nitrazadon or Nitrazepam* or Normison or Novo‐Clopate or Nupentin or Nydrane or Onfi or Orfiril or Orlept or Ormodon or Ospolot or OXC or Oxcarbazepin* or Pacisyn or Paraldehyd* or Paramethadion* or Paxadorm or Paxam or PB or Peganone or Pentothal or Perampanel* or Petinutin or Petril or Phemiton or Phenacemid* or Pheneturid* or Phenobarbit* or Phensuximid* or Phenytek or Phenytoin* or PHT or Posedrine or Potiga or Pregabalin* or Primidon* or Prodilantin or Progabid* or Prominal or Propofol* or Prysoline or Qudexy or Ravotril or Remacemid* or Remnos or Resimatil or Restoril or Retigabin* or Rilutek or Riluzol* or Riv?tril or Rufinamid* or Sabril or Seclar or Selenica or Seletracetam* or Sertan or Somnite or Stavzor or Stedesa or Stiripentol* or Sulthiam* or Sultiam* or Talampanel* or Tegret?l or Temazep* or Temesta or Teril or Thiopent* or Tiagabin* or Tiletamin* or Timonil or Topamax or Topiramat* or Topiramic or TPM or Tranxene or Tridione or Trileptal or Trileptin or Trimethadion* or Trobalt or Urban?l or Valance or Valcote or Valium or Valnoctamid* or Valparin or Valpro* or Versed or Vigabatrin* or Vimpat or VPA or Xcopri or Xilep or YKP3089 or Zalkote or Zarontin or Zebinix or ZNS or Zonegran or Zonisamid*))) AND (((((TITLE‐ABS((randomiz* OR randomis* OR controlled OR placebo OR blind* OR unblind* OR "parallel group" OR crossover OR "cross over" OR cluster OR "head to head") W/4 (analy* OR design OR evaluat* OR investigat* OR method OR procedure OR study OR studies OR trial))) OR (TITLE‐ABS((prospective) W/4 (analys* OR cohort* OR data OR evaluat* OR investigat* OR series OR study OR studies OR trial)))) AND NOT (TITLE(animal OR mouse OR mice OR murine OR rat OR rodent OR dog OR canine OR zebrafish) AND NOT TITLE(human* OR patient OR child* OR infant* OR adolescen* OR adult OR elderly OR man OR men OR male OR wom?n OR female))) AND NOT (TITLE(case PRE/0 (report OR study OR studies)))) OR (TITLE‐ABS‐KEY((registr* OR register) W/4 (analy* OR data OR study OR studies OR trial))))) AND (PUBYEAR > 2013)

### Appendix 4. ClinicalTrials.gov search strategy

pregnant OR pregnancy OR fetus | Congenital Malformation OR Congenital Abnormalities | anticonvulsant OR antiepileptic OR antiseizure | First posted on or after 09/14/2015

### Appendix 5. ICTRP search strategy

(Congenital Malformation OR Congenital Abnormalities) AND (anticonvulsant OR antiepileptic OR antiseizure) AND (pregnant OR pregnancy OR fetus)

### Appendix 6. ROBINS‐I Adaptation and Rating Framework

The authors reviewed the Robins‐I framework and adpated it for use in this context. The original framework and the signalling questions were reviewed for suitability for classifying quality. Key confounder and mediating variables were determined based on a literature search and author knowledge. The wording of each of the signalling questions was adapted to reflect the issue of exposure to a medication. The criteria for each of the ratings was set by the authors and trialed on three included papers. This framework is available on request from the authors.

## Data and analyses

**Comparison 1:** CBZ vs Controls

Outcome or subgroup title	No. of studies	No. of participants	Statistical method	Effect size
1.1 CBZ vs Controls: All Major Malformations	33		Risk Ratio (M‐H, Fixed, 95% CI)	Subtotals only
1.1.1 CBZ vs Women Without Epilepsy (cohort studies)	13	5047	Risk Ratio (M‐H, Fixed, 95% CI)	2.30 [1.47, 3.59]
1.1.2 CBZ vs WWE ‐ No Medication (cohort studies)	20	5289	Risk Ratio (M‐H, Fixed, 95% CI)	1.44 [1.05, 1.96]
1.1.3 CBZ vs Women Without Epilepsy (database studies)	2	373094	Risk Ratio (M‐H, Fixed, 95% CI)	1.14 [0.80, 1.64]
1.1.4 CBZ vs WWE ‐ No Medication (database studies)	4	14334	Risk Ratio (M‐H, Fixed, 95% CI)	1.42 [1.10, 1.83]
1.2 CBZ vs Controls: Neural Tube Malformations	13		Risk Ratio (M‐H, Fixed, 95% CI)	Subtotals only
1.2.1 CBZ vs Women Without Epilepsy (cohort studies)	7	2070	Risk Ratio (M‐H, Fixed, 95% CI)	3.09 [0.38, 25.40]
1.2.2 CBZ vs WWE ‐ No Medication (cohort studies)	9	1873	Risk Ratio (M‐H, Fixed, 95% CI)	2.54 [0.63, 10.20]
1.3 CBZ vs Controls: Cardiac Malformations	14		Risk Ratio (M‐H, Fixed, 95% CI)	Subtotals only
1.3.1 CBZ vs Women Without Epilepsy (cohort studies)	7	2070	Risk Ratio (M‐H, Fixed, 95% CI)	1.46 [0.43, 4.99]
1.3.2 CBZ vs WWE ‐ No Medication (cohort studies)	11	1903	Risk Ratio (M‐H, Fixed, 95% CI)	0.87 [0.41, 1.84]
1.4 CBZ vs Controls: Oro‐Facial Cleft/Craniofacial Malformations	13		Risk Ratio (M‐H, Fixed, 95% CI)	Subtotals only
1.4.1 CBZ vs Women Without Epilepsy (cohort studies)	7	2070	Risk Ratio (M‐H, Fixed, 95% CI)	9.04 [2.16, 37.87]
1.4.2 CBZ vs WWE ‐ No Medication (cohort studies)	9	1056	Risk Ratio (M‐H, Fixed, 95% CI)	0.99 [0.27, 3.62]
1.5 CBZ vs Controls: Skeletal/Limb Malformations	13		Risk Ratio (M‐H, Fixed, 95% CI)	Subtotals only
1.5.1 CBZ vs Woment Without Epilepsy (cohort studies)	7	2070	Risk Ratio (M‐H, Fixed, 95% CI)	5.13 [0.52, 50.67]
1.5.2 CBZ vs WWE ‐ No Medication (cohort studies)	9	1873	Risk Ratio (M‐H, Fixed, 95% CI)	0.96 [0.33, 2.82]

**Comparison 2:** CZP vs Controls

Outcome or subgroup title	No. of studies	No. of participants	Statistical method	Effect size
2.1 CZP vs Controls: All Major Malformations	5		Risk Ratio (M‐H, Fixed, 95% CI)	Subtotals only
2.1.1 CZP vs Women Without Epilepsy (cohort studies)	2	569	Risk Ratio (M‐H, Fixed, 95% CI)	2.76 [0.55, 13.94]
2.1.2 CZP vs WWE ‐ No Medication (cohort studies)	3	555	Risk Ratio (M‐H, Fixed, 95% CI)	1.08 [0.21, 5.42]
2.1.3 CZP vs Women Without Epilepsy (database studies)	1	369380	Risk Ratio (M‐H, Fixed, 95% CI)	0.70 [0.18, 2.77]
2.1.4 CZP vs WWE ‐ No Medication (database studies)	1	2013	Risk Ratio (M‐H, Fixed, 95% CI)	0.69 [0.17, 2.79]

**Comparison 3:** GBP vs Controls

Outcome or subgroup title	No. of studies	No. of participants	Statistical method	Effect size
3.1 GBP vs Controls: All Major Malformations	4		Risk Ratio (M‐H, Fixed, 95% CI)	Subtotals only
3.1.1 GBP vs Women Without Epilepsy (cohort studies)	2	717	Risk Ratio (M‐H, Fixed, 95% CI)	1.78 [0.50, 6.29]
3.1.2 GBP vs WWE ‐ No Medication (cohort studies)	3	768	Risk Ratio (M‐H, Fixed, 95% CI)	1.77 [0.46, 6.90]
3.2 GBP vs Controls: Neural Tube Malformations	1		Risk Ratio (M‐H, Fixed, 95% CI)	Subtotals only
3.2.1 GBP vs Women Without Epilepsy (cohort studies)	1	0	Risk Ratio (M‐H, Fixed, 95% CI)	Not estimable
3.3 GBP vs Controls: Cardiac Malformations	1		Risk Ratio (M‐H, Fixed, 95% CI)	Subtotals only
3.3.1 GBP vs Women Without Epilepsy (cohort studies)	1	130	Risk Ratio (M‐H, Fixed, 95% CI)	129.00 [6.49, 2562.48]
3.3.2 GBP vs WWE ‐ No Medication (cohort studies)	1	6	Risk Ratio (M‐H, Fixed, 95% CI)	5.00 [0.29, 87.54]
3.4 GBP vs Controls: Oro‐Facial Cleft/Craniofacial Malformations	1		Risk Ratio (M‐H, Fixed, 95% CI)	Subtotals only
3.4.1 GBP vs Women Without Epilepsy (cohort studies)	1	0	Risk Ratio (M‐H, Fixed, 95% CI)	Not estimable
3.5 GBP vs Controls: Skeletal/Limb Malformations	1		Risk Ratio (M‐H, Fixed, 95% CI)	Subtotals only
3.5.1 GBP vs Women Without Epilepsy (cohort studies)	1	0	Risk Ratio (M‐H, Fixed, 95% CI)	Not estimable

**Comparison 4:** LEV vs Controls

Outcome or subgroup title	No. of studies	No. of participants	Statistical method	Effect size
4.1 LEV vs Controls: All Major Malformations	11		Risk Ratio (M‐H, Fixed, 95% CI)	Subtotals only
4.1.1 LEV vs Women Without Epilepsy (cohort studies)	4	1596	Risk Ratio (M‐H, Fixed, 95% CI)	2.20 [0.98, 4.93]
4.1.2 LEV vs WWE ‐ No Medication (cohort studies)	6	1825	Risk Ratio (M‐H, Fixed, 95% CI)	0.71 [0.39, 1.28]
4.1.3 LEV vs Women Without Epilepsy (database studies)	1	369385	Risk Ratio (M‐H, Fixed, 95% CI)	0.67 [0.17, 2.66]
4.1.4 LEV vs WWE ‐ No Medication (database studies)	2	10625	Risk Ratio (M‐H, Fixed, 95% CI)	0.82 [0.39, 1.71]
4.2 LEV vs Controls: Neural Tube Malformations	4		Risk Ratio (M‐H, Fixed, 95% CI)	Subtotals only
4.2.1 LEV vs Women Without Epilepsy (cohort studies)	2	0	Risk Ratio (M‐H, Fixed, 95% CI)	Not estimable
4.2.2 LEV vs WWE ‐ No Medication (cohort studies)	2	0	Risk Ratio (M‐H, Fixed, 95% CI)	Not estimable
4.3 LEV vs Controls: Cardiac Malformations	5		Risk Ratio (M‐H, Fixed, 95% CI)	Subtotals only
4.3.1 LEV vs Women Without Epilepsy (cohort studies)	2	488	Risk Ratio (M‐H, Fixed, 95% CI)	3.92 [0.57, 27.07]
4.3.2 LEV vs WWE ‐ No Medication (cohort studies)	4	665	Risk Ratio (M‐H, Fixed, 95% CI)	0.90 [0.31, 2.60]
4.4 LEV vs Controls: Oro‐Facial Cleft/Craniofacial Malformations	4		Risk Ratio (M‐H, Fixed, 95% CI)	Subtotals only
4.4.1 LEV vs Women Without Epilepsy (cohort studies)	2	0	Risk Ratio (M‐H, Fixed, 95% CI)	Not estimable
4.4.2 LEV vs WWE ‐ No Medication (cohort studies)	3	230	Risk Ratio (M‐H, Fixed, 95% CI)	0.14 [0.01, 3.18]
4.5 LEV vs Controls: Skeletal/Limb Malformations	4		Risk Ratio (M‐H, Fixed, 95% CI)	Subtotals only
4.5.1 LEV vs Women Without Epilepsy (cohort studies)	2	0	Risk Ratio (M‐H, Fixed, 95% CI)	Not estimable
4.5.2 LEV vs WWE ‐ No Medication (cohort studies)	3	648	Risk Ratio (M‐H, Fixed, 95% CI)	3.21 [0.46, 22.50]

**Comparison 5:** LTG vs Controls

Outcome or subgroup title	No. of studies	No. of participants	Statistical method	Effect size
5.1 LTG vs Controls: All Major Malformations	16		Risk Ratio (M‐H, Fixed, 95% CI)	Subtotals only
5.1.1 LTG vs Women Without Epilepsy (cohort studies)	7	4862	Risk Ratio (M‐H, Fixed, 95% CI)	1.99 [1.16, 3.39]
5.1.2 LTG vs WWE ‐ No Medication (cohort studies)	8	3918	Risk Ratio (M‐H, Fixed, 95% CI)	1.04 [0.66, 1.63]
5.1.3 LTG vs Women Without Epilepsy (database studies)	2	373288	Risk Ratio (M‐H, Fixed, 95% CI)	1.19 [0.86, 1.64]
5.1.4 LTG vs WWE ‐ No Medication (database studies)	3	13445	Risk Ratio (M‐H, Fixed, 95% CI)	1.00 [0.79, 1.28]
5.2 LTG vs Controls: Neural Tube Malformations	8		Risk Ratio (M‐H, Fixed, 95% CI)	Subtotals only
5.2.1 LTG vs Women Without Epilepsy (cohort studies)	5	1967	Risk Ratio (M‐H, Fixed, 95% CI)	7.55 [1.05, 54.09]
5.2.2 LTG vs WWE ‐ No Medication (cohort studies)	5	0	Risk Ratio (M‐H, Fixed, 95% CI)	Not estimable
5.3 LTG vs Controls: Cardiac Malformations	9		Risk Ratio (M‐H, Fixed, 95% CI)	Subtotals only
5.3.1 LTG vs Women Without Epilepsy (cohort studies)	5	2006	Risk Ratio (M‐H, Fixed, 95% CI)	2.71 [1.05, 6.98]
5.3.2 LTG vs WWE ‐ No Medication (cohort studies)	6	1112	Risk Ratio (M‐H, Fixed, 95% CI)	0.97 [0.28, 3.32]
5.4 LTG vs Controls: Oro‐Facial Cleft/Crainofacial Malformations	7		Risk Ratio (M‐H, Fixed, 95% CI)	Subtotals only
5.4.1 LTG vs Women Without Epilepsy (cohort studies)	4	0	Risk Ratio (M‐H, Fixed, 95% CI)	Not estimable
5.4.2 LTG vs WWE ‐ No Medication (cohort studies)	5	813	Risk Ratio (M‐H, Fixed, 95% CI)	1.37 [0.29, 6.56]
5.5 LTG vs Controls: Skeletal/Limb Malformations	8		Risk Ratio (M‐H, Fixed, 95% CI)	Subtotals only
5.5.1 LTG vs Women Without Epilepsy (cohort studies)	5	1965	Risk Ratio (M‐H, Fixed, 95% CI)	11.29 [2.37, 53.91]
5.5.2 LTG vs WWE ‐ No Medication (cohort studies)	5	1084	Risk Ratio (M‐H, Fixed, 95% CI)	0.75 [0.20, 2.89]

**Comparison 6:** OXC vs Controls

Outcome or subgroup title	No. of studies	No. of participants	Statistical method	Effect size
6.1 OXC vs Controls: All Major Malformations	11		Risk Ratio (M‐H, Fixed, 95% CI)	Subtotals only
6.1.1 OXC vs Women Without Epilepsy (cohort studies)	3	951	Risk Ratio (M‐H, Fixed, 95% CI)	2.20 [0.67, 7.27]
6.1.2 OXC vs WWE ‐ No Medication (cohort studies)	6	922	Risk Ratio (M‐H, Fixed, 95% CI)	1.40 [0.68, 2.91]
6.1.3 OXC vs Women Without Epilepsy (database studies)	1	369324	Risk Ratio (M‐H, Fixed, 95% CI)	0.70 [0.10, 4.86]
6.1.4 OXC vs WWE ‐ No Medication (database studies)	3	11819	Risk Ratio (M‐H, Fixed, 95% CI)	1.75 [1.22, 2.52]
6.2 OXC vs Controls: Neural Tube Malformations	2		Risk Ratio (M‐H, Fixed, 95% CI)	Subtotals only
6.2.1 OXC vs WWE ‐ No Medication (cohort studies)	2	0	Risk Ratio (M‐H, Fixed, 95% CI)	Not estimable
6.3 OXC vs Controls: Cardiac Malformations	4		Risk Ratio (M‐H, Fixed, 95% CI)	Subtotals only
6.3.1 OXC vs Women Without Epilepsy (cohort studies)	1	0	Risk Ratio (M‐H, Fixed, 95% CI)	Not estimable
6.3.2 OXC vs WWE ‐ No Medication (cohort studies)	4	479	Risk Ratio (M‐H, Fixed, 95% CI)	1.10 [0.36, 3.35]
6.4 OXC vs Controls: Oro‐Facial Cleft/Craniofacial Malformations	2		Risk Ratio (M‐H, Fixed, 95% CI)	Subtotals only
6.4.1 OXC vs WWE ‐ No Medication (cohort studies)	2	63	Risk Ratio (M‐H, Fixed, 95% CI)	0.75 [0.04, 14.71]
6.5 OXC vs Controls: Skeletal/Limb Malformations	2		Risk Ratio (M‐H, Fixed, 95% CI)	Subtotals only
6.5.1 OXC vs WWE ‐ No Medication (cohort studies)	2	463	Risk Ratio (M‐H, Fixed, 95% CI)	2.39 [0.22, 26.05]

**Comparison 7:** PB vs Controls

Outcome or subgroup title	No. of studies	No. of participants	Statistical method	Effect size
7.1 PB vs Controls: All Major Malformations	19		Risk Ratio (M‐H, Fixed, 95% CI)	Subtotals only
7.1.1 PB vs Women Without Epilepsy (cohort studies)	8	2395	Risk Ratio (M‐H, Fixed, 95% CI)	3.22 [1.84, 5.65]
7.1.2 PB vs WWE ‐ No Medication (cohort studies)	13	1437	Risk Ratio (M‐H, Fixed, 95% CI)	1.64 [0.94, 2.83]
7.1.3 PB vs Women Without Epilepsy (database studies)	1	369294	Risk Ratio (M‐H, Fixed, 95% CI)	2.94 [0.77, 11.15]
7.1.4 PB vs WWE ‐ No Medication (database studies)	1	1927	Risk Ratio (M‐H, Fixed, 95% CI)	2.87 [0.74, 11.21]
7.2 PB vs Controls: Neural Tube Malformations	4		Risk Ratio (M‐H, Fixed, 95% CI)	Subtotals only
7.2.1 PB vs Women Without Epilepsy (cohort studies)	2	0	Risk Ratio (M‐H, Fixed, 95% CI)	Not estimable
7.2.2 PB vs WWE ‐ No Medication (cohort studies)	3	658	Risk Ratio (M‐H, Fixed, 95% CI)	3.85 [0.47, 31.26]
7.3 PB vs Controls: Cardiac Malformations	4		Risk Ratio (M‐H, Fixed, 95% CI)	Subtotals only
7.3.1 PB vs Women Without Epilepsy (cohort studies)	2	251	Risk Ratio (M‐H, Fixed, 95% CI)	7.80 [0.36, 168.52]
7.3.2 PB vs WWE ‐ No Medication (cohort studies)	4	665	Risk Ratio (M‐H, Fixed, 95% CI)	1.80 [0.69, 4.71]
7.4 PB vs Controls: Oro‐Facial Cleft/Craniofacial Malformations	3		Risk Ratio (M‐H, Fixed, 95% CI)	Subtotals only
7.4.1 PB vs Women Without Epilepsy (cohort studies)	2	251	Risk Ratio (M‐H, Fixed, 95% CI)	3.34 [0.20, 56.35]
7.4.2 PB vs WWE ‐ No Medication (cohort studies)	2	0	Risk Ratio (M‐H, Fixed, 95% CI)	Not estimable
7.5 PB vs Controls: Skeletal/Limb Malformations	4		Risk Ratio (M‐H, Fixed, 95% CI)	Subtotals only
7.5.1 PB vs Women Without Epilepsy (cohort studies)	2	251	Risk Ratio (M‐H, Fixed, 95% CI)	7.80 [0.36, 168.52]
7.5.2 PB vs WWE ‐ No Medication (cohort studies)	3	658	Risk Ratio (M‐H, Fixed, 95% CI)	3.01 [0.56, 16.07]

**Comparison 8:** PHT vs Controls

Outcome or subgroup title	No. of studies	No. of participants	Statistical method	Effect size
8.1 PHT vs Controls: All Major Malformations	19		Risk Ratio (M‐H, Fixed, 95% CI)	Subtotals only
8.1.1 PHT vs Women Without Epilepsy (cohort studies)	8	1893	Risk Ratio (M‐H, Fixed, 95% CI)	3.81 [1.91, 7.57]
8.1.2 PHT vs WWE ‐ No Medication (cohort studies)	15	2338	Risk Ratio (M‐H, Fixed, 95% CI)	2.01 [1.29, 3.12]
8.2 PHT vs Controls: Neural Tube Malformations	8		Risk Ratio (M‐H, Fixed, 95% CI)	Subtotals only
8.2.1 PHT vs Women Without Epilepsy (cohort studies)	4	638	Risk Ratio (M‐H, Fixed, 95% CI)	13.17 [0.58, 299.00]
8.2.2 PHT vs WWE ‐ No Medication (cohort studies)	6	847	Risk Ratio (M‐H, Fixed, 95% CI)	2.56 [0.64, 10.17]
8.3 PHT vs Controls: Cardiac Malformations	8		Risk Ratio (M‐H, Fixed, 95% CI)	Subtotals only
8.3.1 PHT vs Women Without Epilepsy (cohort studies)	4	638	Risk Ratio (M‐H, Fixed, 95% CI)	6.31 [0.75, 52.91]
8.3.2 PHT vs WWE ‐ No Medication (cohort studies)	7	852	Risk Ratio (M‐H, Fixed, 95% CI)	1.86 [0.72, 4.80]
8.4 PHT vs Controls: Oro‐Facial Cleft/Craniofacial Malformations	7		Risk Ratio (M‐H, Fixed, 95% CI)	Subtotals only
8.4.1 PHT vs Women Without Epilepsy (cohort studies)	4	638	Risk Ratio (M‐H, Fixed, 95% CI)	0.67 [0.04, 12.54]
8.4.2 PHT vs WWE ‐ No Medication (cohort studies)	5	0	Risk Ratio (M‐H, Fixed, 95% CI)	Not estimable
8.5 PHT vs Controls: Skeletal/Limb Malformations	8		Risk Ratio (M‐H, Fixed, 95% CI)	Subtotals only
8.5.1 PHT vs Women Without Epilepsy (cohort studies)	4	638	Risk Ratio (M‐H, Fixed, 95% CI)	1.56 [0.07, 37.19]
8.5.2 PHT vs WWE ‐ No Medication (cohort studies)	6	847	Risk Ratio (M‐H, Fixed, 95% CI)	1.57 [0.31, 7.95]

**Comparison 9:** PRM vs Controls

Outcome or subgroup title	No. of studies	No. of participants	Statistical method	Effect size
9.1 PRM vs Controls: All Major Malformations	6		Risk Ratio (M‐H, Fixed, 95% CI)	Subtotals only
9.1.1 PRM vs Women Without Epilepsy (cohort studies)	1	137	Risk Ratio (M‐H, Fixed, 95% CI)	0.48 [0.03, 8.43]
9.1.2 PRM vs WWE ‐ No Medication (cohort studies)	6	681	Risk Ratio (M‐H, Fixed, 95% CI)	3.61 [1.41, 9.23]

**Comparison 10:** TPM vs Controls

Outcome or subgroup title	No. of studies	No. of participants	Statistical method	Effect size
10.1 TPM vs Controls: All Major Malformations	8		Risk Ratio (M‐H, Fixed, 95% CI)	Subtotals only
10.1.1 TPM vs Women Without Epilepsy (cohort studies)	3	1192	Risk Ratio (M‐H, Fixed, 95% CI)	4.07 [1.64, 10.14]
10.1.2 TPM vs WWE ‐ No Medication (cohort studies)	5	1219	Risk Ratio (M‐H, Fixed, 95% CI)	1.37 [0.57, 3.27]
10.1.3 TPM vs Women Without Epilepsy (database studies)	1	369315	Risk Ratio (M‐H, Fixed, 95% CI)	1.65 [0.43, 6.42]
10.1.4 TPM vs WWE ‐ No Medication (database studies)	1	1948	Risk Ratio (M‐H, Fixed, 95% CI)	1.62 [0.40, 6.45]
10.2 TPM vs Controls: Neural Tube Malformations	4		Risk Ratio (M‐H, Fixed, 95% CI)	Subtotals only
10.2.1 TPM vs Women Without Epilepsy (cohort studies)	2	0	Risk Ratio (M‐H, Fixed, 95% CI)	Not estimable
10.2.2 TPM vs WWE ‐ No Medication (cohort studies)	3	0	Risk Ratio (M‐H, Fixed, 95% CI)	Not estimable
10.3 TPM vs Controls: Cardiac Malformations	5		Risk Ratio (M‐H, Fixed, 95% CI)	Subtotals only
10.3.1 TPM vs Women Without Epilepsy (cohort studies)	2	391	Risk Ratio (M‐H, Fixed, 95% CI)	20.71 [2.64, 162.72]
10.3.2 TPM vs WWE ‐ No Medication (cohort studies)	4	570	Risk Ratio (M‐H, Fixed, 95% CI)	2.48 [0.49, 12.49]
10.4 TPM vs Controls: Oro‐Facial Cleft/Craniofacial Malformations	4		Risk Ratio (M‐H, Fixed, 95% CI)	Subtotals only
10.4.1 TPM vs Women Without Epilepsy (cohort studies)	2	0	Risk Ratio (M‐H, Fixed, 95% CI)	Not estimable
10.4.2 TPM vs WWE ‐ No Medication (cohort studies)	3	221	Risk Ratio (M‐H, Fixed, 95% CI)	1.50 [0.09, 24.92]
10.5 TPM vs Controls: Skeletal/Limb Malformations	4		Risk Ratio (M‐H, Fixed, 95% CI)	Subtotals only
10.5.1 TPM vs Women Without Epilepsy (cohort studies)	2	0	Risk Ratio (M‐H, Fixed, 95% CI)	Not estimable
10.5.2 TPM vs WWE ‐ No Medication (cohort studies)	3	561	Risk Ratio (M‐H, Fixed, 95% CI)	2.06 [0.24, 17.42]

**Comparison 11:** VPA vs Controls

Outcome or subgroup title	No. of studies	No. of participants	Statistical method	Effect size
11.1 VPA vs Controls: All Major Malformations	29		Risk Ratio (M‐H, Fixed, 95% CI)	Subtotals only
11.1.1 VPA vs Women Without Epilepsy (cohort studies)	10	3135	Risk Ratio (M‐H, Fixed, 95% CI)	5.53 [3.29, 9.29]
11.1.2 VPA vs WWE ‐ No Med Controls (cohort studies)	17	3998	Risk Ratio (M‐H, Fixed, 95% CI)	2.77 [2.03, 3.79]
11.1.3 VPA vs Women Without Epilepsy (database studies)	3	373649	Risk Ratio (M‐H, Fixed, 95% CI)	2.29 [1.71, 3.08]
11.1.4 VPA vs WWE ‐ No Med Controls (database studies)	4	13369	Risk Ratio (M‐H, Fixed, 95% CI)	3.01 [2.42, 3.75]
11.2 VPA vs Controls: Neural Tube Malformations	11		Risk Ratio (M‐H, Fixed, 95% CI)	Subtotals only
11.2.1 VPA vs Women Without Epilepsy (cohort studies)	4	940	Risk Ratio (M‐H, Fixed, 95% CI)	6.05 [0.94, 38.81]
11.2.2 VPA vs WWE ‐ No Medication (cohort studies)	8	1478	Risk Ratio (M‐H, Fixed, 95% CI)	5.64 [1.37, 23.24]
11.2.3 VPA vs WWE ‐ No Medication (database studies)	1	1127	Risk Ratio (M‐H, Fixed, 95% CI)	8.02 [1.48, 43.50]
11.3 VPA vs Controls: Cardiac Malformations	11		Risk Ratio (M‐H, Fixed, 95% CI)	Subtotals only
11.3.1 VPA vs Women without Medication (cohort studies)	4	940	Risk Ratio (M‐H, Fixed, 95% CI)	11.89 [2.88, 49.08]
11.3.2 VPA vs WWE ‐ No Medication (cohort studies)	10	1497	Risk Ratio (M‐H, Fixed, 95% CI)	2.71 [1.42, 5.17]
11.4 VPA vs Controls: Oro‐Facial Cleft/Craniofacial Malformations	10		Risk Ratio (M‐H, Fixed, 95% CI)	Subtotals only
11.4.1 VPA vs Women Without Epilepsy (cohort studies)	4	940	Risk Ratio (M‐H, Fixed, 95% CI)	2.76 [0.31, 24.78]
11.4.2 VPA vs WWE ‐ No Medication (cohort studies)	8	806	Risk Ratio (M‐H, Fixed, 95% CI)	4.44 [1.14, 17.27]
11.5 VPA vs Controls: Skeletal/Limb Malformations	10		Risk Ratio (M‐H, Fixed, 95% CI)	Subtotals only
11.5.1 VPA vs Women Without Epilepsy (cohort study)	4	940	Risk Ratio (M‐H, Fixed, 95% CI)	16.48 [2.46, 110.49]
11.5.2 VPA vs WWE ‐ No Medication (cohort study)	8	1478	Risk Ratio (M‐H, Fixed, 95% CI)	2.38 [0.93, 6.12]

**Comparison 12:** ZNS vs Controls

Outcome or subgroup title	No. of studies	No. of participants	Statistical method	Effect size
12.1 ZNS vs Controls: All Major Malformations	3		Risk Ratio (M‐H, Fixed, 95% CI)	Subtotals only
12.1.1 ZNS vs Women Without Epilepsy (cohort studies)	2	651	Risk Ratio (M‐H, Fixed, 95% CI)	1.13 [0.21, 6.11]
12.1.2 ZNS vs WWE ‐ No Medication (cohort studies)	2	595	Risk Ratio (M‐H, Fixed, 95% CI)	3.20 [1.09, 9.43]
12.2 ZNS vs Controls: Neural Tube Malformations	1		Risk Ratio (M‐H, Fixed, 95% CI)	Subtotals only
12.2.1 ZNS vs Women Without Epilepsy (cohort study)	1	0	Risk Ratio (M‐H, Fixed, 95% CI)	Not estimable
12.2.2 ZNS vs WWE ‐ No Medication (cohort study)	1	0	Risk Ratio (M‐H, Fixed, 95% CI)	Not estimable
12.3 ZNS vs Controls: Cardiac Malformations	1		Risk Ratio (M‐H, Fixed, 95% CI)	Subtotals only
12.3.1 ZNS vs Women Without Epilepsy (cohort study)	1	119	Risk Ratio (M‐H, Fixed, 95% CI)	2.55 [0.11, 59.56]
12.3.2 ZNS vs WWE ‐ No Medication (cohort study)	1	0	Risk Ratio (M‐H, Fixed, 95% CI)	Not estimable
12.4 ZNS vs Controls: Oro‐Facial Cleft/Craniofacial Malformations	1		Risk Ratio (M‐H, Fixed, 95% CI)	Subtotals only
12.4.1 ZNS vs Women Without Epilepsy (cohort study)	1	0	Risk Ratio (M‐H, Fixed, 95% CI)	Not estimable
12.4.2 ZNS vs WWE ‐ No Medication (cohort study)	1	0	Risk Ratio (M‐H, Fixed, 95% CI)	Not estimable
12.5 ZNS vs Controls: Skeletal/Limb Malformations	1		Risk Ratio (M‐H, Fixed, 95% CI)	Subtotals only
12.5.1 ZNS vs Women without Epilepsy (cohort study)	1	0	Risk Ratio (M‐H, Fixed, 95% CI)	Not estimable
12.5.2 ZNS vs WWE ‐ No Medication (cohort study)	1	0	Risk Ratio (M‐H, Fixed, 95% CI)	Not estimable

**Comparison 13:** CBZ vs CZP

Outcome or subgroup title	No. of studies	No. of participants	Statistical method	Effect size
13.1 CBZ vs CZP: All Major Malformations	6		Risk Ratio (M‐H, Fixed, 95% CI)	Subtotals only
13.1.1 CBZ vs CZP (cohort studies)	4	1406	Risk Ratio (M‐H, Fixed, 95% CI)	1.82 [0.63, 5.26]
13.1.2 CBZ vs CZP (database studies)	2	1549	Risk Ratio (M‐H, Fixed, 95% CI)	1.29 [0.47, 3.51]

**Comparison 14:** CBZ vs GBP

Outcome or subgroup title	No. of studies	No. of participants	Statistical method	Effect size
14.1 CBZ vs GBP: All Major Malformations	5		Risk Ratio (M‐H, Fixed, 95% CI)	Subtotals only
14.1.1 CBZ vs GBP (cohort studies)	4	3304	Risk Ratio (M‐H, Fixed, 95% CI)	1.55 [0.57, 4.26]
14.1.2 CBZ vs GBP (database studies)	1	721	Risk Ratio (M‐H, Fixed, 95% CI)	1.54 [0.10, 24.27]
14.2 CBZ vs GBP: Neural Tube Malformations	1		Risk Ratio (M‐H, Fixed, 95% CI)	Subtotals only
14.2.1 CBZ vs GBP (cohort studies)	1	375	Risk Ratio (M‐H, Fixed, 95% CI)	0.12 [0.01, 2.93]
14.3 CBZ vs GBP: Cardiac Malformations	2		Risk Ratio (M‐H, Fixed, 95% CI)	Subtotals only
14.3.1 CBZ vs GBP (cohort studies)	2	390	Risk Ratio (M‐H, Fixed, 95% CI)	0.13 [0.02, 0.95]
14.4 CBZ vs GBP: Oro‐Facial Cleft/Craniofacial Malformations	1		Risk Ratio (M‐H, Fixed, 95% CI)	Subtotals only
14.4.1 CBZ vs GBP (cohort studies)	1	375	Risk Ratio (M‐H, Fixed, 95% CI)	0.37 [0.02, 6.62]
14.5 CBZ vs GBP: Skeletal/Limb Malformations	1		Risk Ratio (M‐H, Fixed, 95% CI)	Subtotals only
14.5.1 CBZ vs GBP (cohort studies)	1	375	Risk Ratio (M‐H, Fixed, 95% CI)	0.21 [0.01, 4.13]

**Comparison 15:** CBZ vs LEV

Outcome or subgroup title	No. of studies	No. of participants	Statistical method	Effect size
15.1 CBZ vs LEV: All Major Malformations	13		Risk Ratio (M‐H, Fixed, 95% CI)	Subtotals only
15.1.1 CBZ vs LEV (cohort studies)	11	5056	Risk Ratio (M‐H, Fixed, 95% CI)	1.51 [1.01, 2.26]
15.1.2 CBZ vs LEV (database studies)	2	1248	Risk Ratio (M‐H, Fixed, 95% CI)	1.73 [0.78, 3.83]
15.2 CBZ vs LEV: Neural Tube Malformations	10		Risk Ratio (M‐H, Fixed, 95% CI)	Subtotals only
15.2.1 CBZ vs LEV (cohort studies)	10	4879	Risk Ratio (M‐H, Fixed, 95% CI)	1.57 [0.41, 6.08]
15.3 CBZ vs LEV: Cardiac Malformations	11		Risk Ratio (M‐H, Fixed, 95% CI)	Subtotals only
15.3.1 CBZ vs LEV (cohort studies)	11	4892	Risk Ratio (M‐H, Fixed, 95% CI)	1.20 [0.57, 2.52]
15.4 CBZ vs LEV: Oro‐Facial Cleft/Craniofacial Malformations	10		Risk Ratio (M‐H, Fixed, 95% CI)	Subtotals only
15.4.1 CBZ vs LEV (cohort studies)	10	4296	Risk Ratio (M‐H, Fixed, 95% CI)	1.79 [0.43, 7.41]
15.5 CBZ vs LEV: Skeletal/Limb Malformations	10		Risk Ratio (M‐H, Fixed, 95% CI)	Subtotals only
15.5.1 CBZ vs LEV (cohort studies)	10	4878	Risk Ratio (M‐H, Fixed, 95% CI)	0.99 [0.37, 2.68]

**Comparison 16:** CBZ vs LTG

Outcome or subgroup title	No. of studies	No. of participants	Statistical method	Effect size
16.1 CBZ vs LTG: All Major Malformations	17		Risk Ratio (M‐H, Fixed, 95% CI)	Subtotals only
16.1.1 CBZ vs LTG (cohort studies)	13	8568	Risk Ratio (M‐H, Fixed, 95% CI)	1.37 [1.06, 1.77]
16.1.2 CBZ vs LTG (database studies)	4	4503	Risk Ratio (M‐H, Fixed, 95% CI)	1.21 [0.88, 1.67]
16.2 CBZ vs LTG: Neural Tube Malformations	12		Risk Ratio (M‐H, Fixed, 95% CI)	Subtotals only
16.2.1 CBZ vs LTG (cohort studies)	12	8341	Risk Ratio (M‐H, Fixed, 95% CI)	2.19 [0.76, 6.33]
16.3 CBZ vs LTG: Cardiac Malformations	12		Risk Ratio (M‐H, Fixed, 95% CI)	Subtotals only
16.3.1 CBZ vs LTG (cohort studies)	12	8340	Risk Ratio (M‐H, Fixed, 95% CI)	1.48 [0.87, 2.51]
16.4 CBZ vs LTG: Oro‐Facial Cleft/Crainofacial Malformations	11		Risk Ratio (M‐H, Fixed, 95% CI)	Subtotals only
16.4.1 CBZ vs LTG (cohort studies)	11	7800	Risk Ratio (M‐H, Fixed, 95% CI)	1.22 [0.57, 2.61]
16.5 CBZ vs LTG: Skeletal/Limb Malformations	12		Risk Ratio (M‐H, Fixed, 95% CI)	Subtotals only
16.5.1 CBZ vs LTG (cohort studies)	12	8341	Risk Ratio (M‐H, Fixed, 95% CI)	1.86 [0.82, 4.22]

**Comparison 17:** CBZ vs OXC

Outcome or subgroup title	No. of studies	No. of participants	Statistical method	Effect size
17.1 CBZ vs OXC: All Major Malformations	15		Risk Ratio (M‐H, Fixed, 95% CI)	Subtotals only
17.1.1 CBZ vs OXC (cohort studies)	11	2877	Risk Ratio (M‐H, Fixed, 95% CI)	1.26 [0.74, 2.15]
17.1.2 CBZ vs OXC (database studies)	4	3015	Risk Ratio (M‐H, Fixed, 95% CI)	0.64 [0.44, 0.91]
17.2 CBZ vs OXC: Neural Tube Malformations	9		Risk Ratio (M‐H, Fixed, 95% CI)	Subtotals only
17.2.1 CBZ vs OXC (cohort studies)	9	2767	Risk Ratio (M‐H, Fixed, 95% CI)	0.93 [0.22, 3.96]
17.3 CBZ vs OXC: Cardiac Malformations	11		Risk Ratio (M‐H, Fixed, 95% CI)	Subtotals only
17.3.1 CBZ vs OXC (cohort studies)	11	2789	Risk Ratio (M‐H, Fixed, 95% CI)	0.56 [0.23, 1.38]
17.4 CBZ vs OXC: Oro‐Facial Cleft/Craniofacial Malformations	9		Risk Ratio (M‐H, Fixed, 95% CI)	Subtotals only
17.4.1 CBZ vs OXC (cohort studies)	9	2214	Risk Ratio (M‐H, Fixed, 95% CI)	0.52 [0.12, 2.26]
17.5 CBZ vs OXC: Skeletal/Limb Malformations	9		Risk Ratio (M‐H, Fixed, 95% CI)	Subtotals only
17.5.1 CBZ vs OXC (cohort studies)	9	2767	Risk Ratio (M‐H, Fixed, 95% CI)	0.53 [0.17, 1.66]

**Comparison 18:** CBZ vs PB

Outcome or subgroup title	No. of studies	No. of participants	Statistical method	Effect size
18.1 CBZ vs PB: All Major Malformations	26		Risk Ratio (M‐H, Fixed, 95% CI)	Subtotals only
18.1.1 CBZ vs PB (cohort studies)	24	4067	Risk Ratio (M‐H, Fixed, 95% CI)	0.83 [0.61, 1.13]
18.1.2 CBZ vs PB (database studies)	2	1422	Risk Ratio (M‐H, Fixed, 95% CI)	0.35 [0.12, 1.09]
18.2 CBZ vs PB: Neural Tube Malformations	15		Risk Ratio (M‐H, Fixed, 95% CI)	Subtotals only
18.2.1 CBZ vs PB (cohort studies)	15	2890	Risk Ratio (M‐H, Fixed, 95% CI)	1.28 [0.35, 4.75]
18.3 CBZ vs PB: Cardiac Malformations	15		Risk Ratio (M‐H, Fixed, 95% CI)	Subtotals only
18.3.1 CBZ vs PB (cohort studies)	15	2890	Risk Ratio (M‐H, Fixed, 95% CI)	0.26 [0.14, 0.47]
18.4 CBZ vs PB: Oro‐Facial Cleft/Craniofacial Malformations	15		Risk Ratio (M‐H, Fixed, 95% CI)	Subtotals only
18.4.1 CBZ vs PB (cohort studies)	15	2279	Risk Ratio (M‐H, Fixed, 95% CI)	0.18 [0.07, 0.48]
18.5 CBZ vs PB: Skeletal/Limb Malformation	15		Risk Ratio (M‐H, Fixed, 95% CI)	Subtotals only
18.5.1 CBZ vs PB (cohort studies)	15	2890	Risk Ratio (M‐H, Fixed, 95% CI)	1.08 [0.45, 2.61]

**Comparison 19:** CBZ vs PHT

Outcome or subgroup title	No. of studies	No. of participants	Statistical method	Effect size
19.1 CBZ vs PHT: All Major Malformations	24		Risk Ratio (M‐H, Fixed, 95% CI)	Subtotals only
19.1.1 CBZ vs PHT (cohort studies)	23	6046	Risk Ratio (M‐H, Fixed, 95% CI)	0.83 [0.62, 1.11]
19.1.2 CBZ vs PHT (database studies)	1	806	Risk Ratio (M‐H, Fixed, 95% CI)	0.59 [0.26, 1.31]
19.2 CBZ vs PHT: Neural Tube Malformations	16		Risk Ratio (M‐H, Fixed, 95% CI)	Subtotals only
19.2.1 CBZ vs PHT (cohort studies)	16	5346	Risk Ratio (M‐H, Fixed, 95% CI)	1.12 [0.45, 2.83]
19.3 CBZ vs PHT: Cardiac Malformations	16		Risk Ratio (M‐H, Fixed, 95% CI)	Subtotals only
19.3.1 CBZ vs PHT (cohort studies)	16	5346	Risk Ratio (M‐H, Fixed, 95% CI)	0.44 [0.23, 0.84]
19.4 CBZ vs PHT: Oro‐Facial Cleft/Craniofacial Malformations	16		Risk Ratio (M‐H, Fixed, 95% CI)	Subtotals only
19.4.1 CBZ vs PHT (cohort studies)	16	4749	Risk Ratio (M‐H, Fixed, 95% CI)	0.81 [0.32, 2.08]
19.5 CBZ vs PHT: Skeletal/Limb Malformation	16		Risk Ratio (M‐H, Fixed, 95% CI)	Subtotals only
19.5.1 CBZ vs PHT (cohort studies)	16	5346	Risk Ratio (M‐H, Fixed, 95% CI)	0.88 [0.43, 1.82]

**Comparison 20:** CBZ vs PRM

Outcome or subgroup title	No. of studies	No. of participants	Statistical method	Effect size
20.1 CBZ vs PRM: All Major Malformations	8		Risk Ratio (M‐H, Fixed, 95% CI)	Subtotals only
20.1.1 CBZ vs PRM (cohort studies)	7	1188	Risk Ratio (M‐H, Fixed, 95% CI)	0.78 [0.41, 1.48]
20.1.2 CBZ vs PRM (database studies)	1	706	Risk Ratio (M‐H, Fixed, 95% CI)	0.32 [0.02, 4.44]
20.2 CBZ vs PRM: Neural Tube Malformations	2		Risk Ratio (M‐H, Fixed, 95% CI)	Subtotals only
20.2.1 CBZ vs PRM (cohort studies)	2	158	Risk Ratio (M‐H, Fixed, 95% CI)	0.95 [0.04, 22.75]
20.3 CBZ vs PRM: Cardiac Malformations	2		Risk Ratio (M‐H, Fixed, 95% CI)	Subtotals only
20.3.1 CBZ vs PRM (cohort studies)	2	158	Risk Ratio (M‐H, Fixed, 95% CI)	0.11 [0.00, 2.53]
20.4 CBZ vs PRM: Oro‐Facial Cleft/Craniofacial Malformations	2		Risk Ratio (M‐H, Fixed, 95% CI)	Subtotals only
20.4.1 CBZ vs PRM (cohort studies)	2	0	Risk Ratio (M‐H, Fixed, 95% CI)	Not estimable
20.5 CBZ vs PRM: Skeletal/Limb Malformations	2		Risk Ratio (M‐H, Fixed, 95% CI)	Subtotals only
20.5.1 CBZ vs PRM (cohort studies)	2	158	Risk Ratio (M‐H, Fixed, 95% CI)	2.84 [0.16, 51.53]

**Comparison 21:** CBZ vs TPM

Outcome or subgroup title	No. of studies	No. of participants	Statistical method	Effect size
21.1 CBZ vs TPM: All Major Malformations	10		Risk Ratio (M‐H, Fixed, 95% CI)	Subtotals only
21.1.1 CBZ vs TPM (cohort studies)	8	4156	Risk Ratio (M‐H, Fixed, 95% CI)	0.83 [0.51, 1.33]
21.1.2 CBZ vs TPM (database studies)	2	1437	Risk Ratio (M‐H, Fixed, 95% CI)	0.59 [0.17, 2.06]
21.2 CBZ vs TPM: Neural Tube Malformations	7		Risk Ratio (M‐H, Fixed, 95% CI)	Subtotals only
21.2.1 CBZ vs TPM (cohort studies)	7	4064	Risk Ratio (M‐H, Fixed, 95% CI)	0.91 [0.18, 4.51]
21.3 CBZ vs TPM: Cardiac Malformations	8		Risk Ratio (M‐H, Fixed, 95% CI)	Subtotals only
21.3.1 CBZ vs TPM (cohort studies)	8	4070	Risk Ratio (M‐H, Fixed, 95% CI)	0.73 [0.25, 2.12]
21.4 CBZ vs TPM: Oro‐Facial Cleft/Craniofacial Malformations	7		Risk Ratio (M‐H, Fixed, 95% CI)	Subtotals only
21.4.1 CBZ vs TPM (cohort studies)	7	3571	Risk Ratio (M‐H, Fixed, 95% CI)	0.33 [0.13, 0.82]
21.5 CBZ vs TPM: Skeletal/Limb Malformations	7		Risk Ratio (M‐H, Fixed, 95% CI)	Subtotals only
21.5.1 CBZ vs TPM (cohort studies)	7	4064	Risk Ratio (M‐H, Fixed, 95% CI)	0.34 [0.12, 0.94]

**Comparison 22:** CBZ vs VPA

Outcome or subgroup title	No. of studies	No. of participants	Statistical method	Effect size
22.1 CBZ vs VPA: All Major Malformations	34		Risk Ratio (M‐H, Fixed, 95% CI)	Subtotals only
22.1.1 CBZ vs VPA (cohort studies)	29	8090	Risk Ratio (M‐H, Fixed, 95% CI)	0.44 [0.37, 0.53]
22.1.2 CBZ vs VPA (database studies)	5	4157	Risk Ratio (M‐H, Fixed, 95% CI)	0.42 [0.33, 0.54]
22.2 CBZ vs VPA: Neural Tube Malformations	22		Risk Ratio (M‐H, Fixed, 95% CI)	Subtotals only
22.2.1 CBZ vs VPA (cohort studies)	21	7459	Risk Ratio (M‐H, Fixed, 95% CI)	0.24 [0.14, 0.41]
22.2.2 CBZ vs VPA (database studies)	1	971	Risk Ratio (M‐H, Fixed, 95% CI)	0.19 [0.02, 2.09]
22.3 CBZ vs VPA: Cardiac Malformations	23		Risk Ratio (M‐H, Fixed, 95% CI)	Subtotals only
22.3.1 CBZ vs VPA (cohort studies)	22	7465	Risk Ratio (M‐H, Fixed, 95% CI)	0.40 [0.28, 0.58]
22.3.2 CBZ vs VPA (database studies)	1	971	Risk Ratio (M‐H, Fixed, 95% CI)	0.38 [0.13, 1.08]
22.4 CBZ vs VPA: Oro‐Facial Cleft/Craniofacial Malformations	23		Risk Ratio (M‐H, Fixed, 95% CI)	Subtotals only
22.4.1 CBZ vs VPA (cohort studies)	22	6647	Risk Ratio (M‐H, Fixed, 95% CI)	0.31 [0.18, 0.54]
22.4.2 CBZ vs VPA (database studies)	1	971	Risk Ratio (M‐H, Fixed, 95% CI)	0.15 [0.03, 0.78]
22.5 CBZ vs VPA: Skeletal/Limb Malformations	22		Risk Ratio (M‐H, Fixed, 95% CI)	Subtotals only
22.5.1 CBZ vs VPA (cohort studies)	21	7459	Risk Ratio (M‐H, Fixed, 95% CI)	0.31 [0.19, 0.51]
22.5.2 CBZ vs VPA (database studies)	1	971	Risk Ratio (M‐H, Fixed, 95% CI)	0.38 [0.02, 6.07]

**Comparison 23:** CBZ vs ZNS

Outcome or subgroup title	No. of studies	No. of participants	Statistical method	Effect size
23.1 CBZ vs ZNS: All Major Malformations	4		Risk Ratio (M‐H, Random, 95% CI)	Subtotals only
23.1.1 CBZ vs ZNS (cohort studies)	4	2841	Risk Ratio (M‐H, Random, 95% CI)	0.86 [0.07, 10.35]
23.2 CBZ vs ZNS: Neural Tube Malformations	3		Risk Ratio (M‐H, Fixed, 95% CI)	Subtotals only
23.2.1 CBZ vs ZNS (cohort studies)	3	1718	Risk Ratio (M‐H, Fixed, 95% CI)	0.06 [0.01, 0.54]
23.3 CBZ vs ZNS: Cardiac Malformations	3		Risk Ratio (M‐H, Fixed, 95% CI)	Subtotals only
23.3.1 CBZ vs ZNS (cohort studies)	3	1718	Risk Ratio (M‐H, Fixed, 95% CI)	0.47 [0.03, 7.72]
23.4 CBZ vs ZNS: Oro‐Facial Cleft/Craniofacial Malformations	3		Risk Ratio (M‐H, Fixed, 95% CI)	Subtotals only
23.4.1 CBZ vs ZNS (cohort studies)	3	1718	Risk Ratio (M‐H, Fixed, 95% CI)	0.15 [0.01, 2.66]
23.5 CBZ vs ZNS: Skeletal/Limb Malformations	3		Risk Ratio (M‐H, Fixed, 95% CI)	Subtotals only
23.5.1 CBZ vs ZNS (cohort studies)	3	1718	Risk Ratio (M‐H, Fixed, 95% CI)	0.15 [0.01, 2.66]

**Comparison 24:** GBP vs LTG

Outcome or subgroup title	No. of studies	No. of participants	Statistical method	Effect size
24.1 GPB vs LTG: All Major Malformations	5		Risk Ratio (M‐H, Fixed, 95% CI)	Subtotals only
24.1.1 GBP vs LTG (cohort studies)	4	4295	Risk Ratio (M‐H, Fixed, 95% CI)	0.92 [0.34, 2.47]
24.1.2 GBP vs LTG (database studies)	1	108	Risk Ratio (M‐H, Fixed, 95% CI)	0.53 [0.03, 9.48]
24.2 GPB vs LTG: Neural Tube Malformations	1		Risk Ratio (M‐H, Fixed, 95% CI)	Subtotals only
24.2.1 GBP vs LTG (cohort studies)	1	0	Risk Ratio (M‐H, Fixed, 95% CI)	Not estimable
24.3 GPB vs LTG: Cardiac Malformations	2		Risk Ratio (M‐H, Fixed, 95% CI)	Subtotals only
24.3.1 GBP vs LTG (cohort studies)	2	368	Risk Ratio (M‐H, Fixed, 95% CI)	9.57 [1.69, 54.15]
24.4 GPB vs LTG: Oro‐Facial Cleft/Craniofacial Malformations	1		Risk Ratio (M‐H, Fixed, 95% CI)	Subtotals only
24.4.1 GBP vs LTG (cohort studies)	1	329	Risk Ratio (M‐H, Fixed, 95% CI)	1.92 [0.11, 33.05]
24.5 GPB vs LTG: Skeletal/Limb Malformations	1		Risk Ratio (M‐H, Fixed, 95% CI)	Subtotals only
24.5.1 GBP vs LTG (cohort studies)	1	0	Risk Ratio (M‐H, Fixed, 95% CI)	Not estimable

**Comparison 25:** GBP vs OXC

Outcome or subgroup title	No. of studies	No. of participants	Statistical method	Effect size
25.1 GBP vs OXC: All Major Malformations	4		Risk Ratio (M‐H, Fixed, 95% CI)	Subtotals only
25.1.1 GBP vs OXC (cohort studies)	3	363	Risk Ratio (M‐H, Fixed, 95% CI)	0.53 [0.13, 2.17]
25.1.2 GBP vs OXC (database study)	1	0	Risk Ratio (M‐H, Fixed, 95% CI)	Not estimable
25.2 GBP vs OXC: Neural Tube Malformations	1		Risk Ratio (M‐H, Fixed, 95% CI)	Subtotals only
25.2.1 GBP vs OXC (cohort studies)	1	0	Risk Ratio (M‐H, Fixed, 95% CI)	Not estimable
25.3 GBP vs OXC: Cardiac Malformations	2		Risk Ratio (M‐H, Fixed, 95% CI)	Subtotals only
25.3.1 GBP vs OXC (cohort studies)	2	28	Risk Ratio (M‐H, Fixed, 95% CI)	3.00 [0.24, 37.67]
25.4 GBP vs OXC: Oro‐Facial Cleft/Craniofacial Malformations	1		Risk Ratio (M‐H, Fixed, 95% CI)	Subtotals only
25.4.1 GBP vs OXC (cohort studies)	1	0	Risk Ratio (M‐H, Fixed, 95% CI)	Not estimable
25.5 GBP vs OXC: Skeletal/Limb Malformations	1		Risk Ratio (M‐H, Fixed, 95% CI)	Subtotals only
25.5.1 GBP vs OXC (cohort studies)	1	0	Risk Ratio (M‐H, Fixed, 95% CI)	Not estimable

**Comparison 26:** GBP vs PB

Outcome or subgroup title	No. of studies	No. of participants	Statistical method	Effect size
26.1 GBP vs PB: All Major Malformations	4		Risk Ratio (M‐H, Fixed, 95% CI)	Subtotals only
26.1.1 GBP vs PB (cohort studies)	3	365	Risk Ratio (M‐H, Fixed, 95% CI)	0.30 [0.08, 1.14]
26.1.2 GBP vs PB (database studies)	1	25	Risk Ratio (M‐H, Fixed, 95% CI)	0.14 [0.01, 3.09]
26.2 GBP vs PB: Neural Tube Malformations	1		Risk Ratio (M‐H, Fixed, 95% CI)	Subtotals only
26.2.1 GBP vs PB (cohort studies)	1	0	Risk Ratio (M‐H, Fixed, 95% CI)	Not estimable
26.3 GBP vs PB: Cardiac Malformations	2		Risk Ratio (M‐H, Fixed, 95% CI)	Subtotals only
26.3.1 GBP vs PB (cohort studies)	2	24	Risk Ratio (M‐H, Fixed, 95% CI)	4.00 [0.24, 67.71]
26.4 GBP vs PB: Oro‐Facial Cleft/Craniofacial Malformations	1		Risk Ratio (M‐H, Fixed, 95% CI)	Subtotals only
26.4.1 GBP vs PB (cohort studies)	1	0	Risk Ratio (M‐H, Fixed, 95% CI)	Not estimable
26.5 GBP vs PB: Skeletal/Limb Malformations	1		Risk Ratio (M‐H, Fixed, 95% CI)	Subtotals only
26.5.1 GBP vs PB (cohort studies)	1	0	Risk Ratio (M‐H, Fixed, 95% CI)	Not estimable

**Comparison 27:** GBP vs PRM

Outcome or subgroup title	No. of studies	No. of participants	Statistical method	Effect size
27.1 GBP vs PRM: All Major Malformations	1		Risk Ratio (M‐H, Fixed, 95% CI)	Subtotals only
27.1.1 GBP vs PRM (database studies)	1	0	Risk Ratio (M‐H, Fixed, 95% CI)	Not estimable

**Comparison 28:** GBP vs TPM

Outcome or subgroup title	No. of studies	No. of participants	Statistical method	Effect size
28.1 GBP vs TPM: All Major Malformations	4		Risk Ratio (M‐H, Fixed, 95% CI)	Subtotals only
28.1.1 GBP vs TPM (cohort studies)	3	672	Risk Ratio (M‐H, Fixed, 95% CI)	0.32 [0.09, 1.19]
28.1.2 GBP vs TPM (database studies)	1	0	Risk Ratio (M‐H, Fixed, 95% CI)	Not estimable
28.2 GBP vs TPM: Neural Tube Malformations	1		Risk Ratio (M‐H, Fixed, 95% CI)	Subtotals only
28.2.1 GBP vs TPM (cohort studies)	1	0	Risk Ratio (M‐H, Fixed, 95% CI)	Not estimable
28.3 GBP vs TPM: Cardiac Malformations	1		Risk Ratio (M‐H, Fixed, 95% CI)	Subtotals only
28.3.1 GBP vs TPM (cohort studies)	1	0	Risk Ratio (M‐H, Fixed, 95% CI)	Not estimable
28.4 GBP vs TPM: Oro‐Facial Cleft/Craniofacial Malformations	1		Risk Ratio (M‐H, Fixed, 95% CI)	Subtotals only
28.4.1 GBP vs TPM (cohort studies)	1	0	Risk Ratio (M‐H, Fixed, 95% CI)	Not estimable
28.5 GBP vs TPM: Skeletal/Limb Malformations	1		Risk Ratio (M‐H, Fixed, 95% CI)	Subtotals only
28.5.1 GBP vs TPM (cohort studies)	1	0	Risk Ratio (M‐H, Fixed, 95% CI)	Not estimable

**Comparison 29:** GBP vs ZNS

Outcome or subgroup title	No. of studies	No. of participants	Statistical method	Effect size
29.1 GBP vs ZNS: All Major Malformations	2		Risk Ratio (M‐H, Fixed, 95% CI)	Subtotals only
29.1.1 GBP vs ZNS (cohort studies)	2	292	Risk Ratio (M‐H, Fixed, 95% CI)	0.53 [0.10, 2.76]

**Comparison 30:** LEV vs GBP

Outcome or subgroup title	No. of studies	No. of participants	Statistical method	Effect size
30.1 LEV vs GBP: All Major Malformations	3		Risk Ratio (M‐H, Fixed, 95% CI)	Subtotals only
30.1.1 LEV vs GBP (cohort studies)	3	1083	Risk Ratio (M‐H, Fixed, 95% CI)	1.61 [0.46, 5.63]
30.2 LEV vs GBP: Neural Tube Malformations	1		Risk Ratio (M‐H, Fixed, 95% CI)	Subtotals only
30.2.1 LEV vs GBP (cohort studies)	1	0	Risk Ratio (M‐H, Fixed, 95% CI)	Not estimable
30.3 LEV vs GBP: Cardiac Malformations	1		Risk Ratio (M‐H, Fixed, 95% CI)	Subtotals only
30.3.1 LEV vs GBP (cohort studies)	1	77	Risk Ratio (M‐H, Fixed, 95% CI)	0.70 [0.03, 16.42]
30.4 LEV vs GBP: Oro‐Facial Cleft/Craniofacial Malformations	1		Risk Ratio (M‐H, Fixed, 95% CI)	Subtotals only
30.4.1 LEV vs GBP (cohort studies)	1	77	Risk Ratio (M‐H, Fixed, 95% CI)	0.70 [0.03, 16.42]
30.5 LEV vs GBP: Skeletal/Limb Malformation	1		Risk Ratio (M‐H, Fixed, 95% CI)	Subtotals only
30.5.1 LEV vs GBP (cohort studies)	1	0	Risk Ratio (M‐H, Fixed, 95% CI)	Not estimable

**Comparison 31:** LEV vs LTG

Outcome or subgroup title	No. of studies	No. of participants	Statistical method	Effect size
31.1 LEV vs LTG: All Major Malformations	12		Risk Ratio (M‐H, Fixed, 95% CI)	Subtotals only
31.1.1 LEV vs LTG (cohort studies)	10	5612	Risk Ratio (M‐H, Fixed, 95% CI)	0.90 [0.58, 1.39]
31.1.2 LEV vs LTG (database studies)	2	2316	Risk Ratio (M‐H, Fixed, 95% CI)	0.79 [0.37, 1.69]
31.2 LEV vs LTG: Neural Tube Malformations	9		Risk Ratio (M‐H, Fixed, 95% CI)	Subtotals only
31.2.1 LEV vs LTG (cohort studies)	9	5373	Risk Ratio (M‐H, Fixed, 95% CI)	1.59 [0.24, 10.38]
31.3 LEV vs LTG: Cardiac Malformations	9		Risk Ratio (M‐H, Fixed, 95% CI)	Subtotals only
31.3.1 LEV vs LTG (cohort studies)	9	5371	Risk Ratio (M‐H, Fixed, 95% CI)	1.20 [0.51, 2.85]
31.4 LEV vs LTG: Oro‐Facial Cleft/Craniofacial Malformations	8		Risk Ratio (M‐H, Fixed, 95% CI)	Subtotals only
31.4.1 LEV vs LTG (cohort studies)	8	5215	Risk Ratio (M‐H, Fixed, 95% CI)	0.63 [0.15, 2.68]
31.5 LEV vs LTG: Skeletal/Limb Malformation	9		Risk Ratio (M‐H, Fixed, 95% CI)	Subtotals only
31.5.1 LEV vs LTG (cohort studies)	9	5373	Risk Ratio (M‐H, Fixed, 95% CI)	1.36 [0.45, 4.13]

**Comparison 32:** LEV vs OXC

Outcome or subgroup title	No. of studies	No. of participants	Statistical method	Effect size
32.1 LEV vs OXC: All Major Malformations	10		Risk Ratio (M‐H, Fixed, 95% CI)	Subtotals only
32.1.1 LEV vs OXC (cohort studies)	8	1166	Risk Ratio (M‐H, Fixed, 95% CI)	1.04 [0.51, 2.09]
32.1.2 LEV vs OXC (database studies)	2	621	Risk Ratio (M‐H, Fixed, 95% CI)	1.17 [0.45, 3.06]
32.2 LEV vs OXC: Neural Tube Malformations	7		Risk Ratio (M‐H, Fixed, 95% CI)	Subtotals only
32.2.1 LEV vs OXC (cohort studies)	7	1058	Risk Ratio (M‐H, Fixed, 95% CI)	1.22 [0.05, 29.74]
32.3 LEV vs OXC: Cardiac Malformations	8		Risk Ratio (M‐H, Fixed, 95% CI)	Subtotals only
32.3.1 LEV vs OXC (cohort studies)	8	1070	Risk Ratio (M‐H, Fixed, 95% CI)	0.93 [0.31, 2.76]
32.4 LEV vs OXC: Oro‐Facial Cleft/Craniofacial Malformations	7		Risk Ratio (M‐H, Fixed, 95% CI)	Subtotals only
32.4.1 LEV vs OXC (cohort studies)	7	893	Risk Ratio (M‐H, Fixed, 95% CI)	0.25 [0.03, 2.12]
32.5 LEV vs OXC: Skeletal/Limb Malformations	7		Risk Ratio (M‐H, Fixed, 95% CI)	Subtotals only
32.5.1 LEV vs OXC (cohort studies)	7	1058	Risk Ratio (M‐H, Fixed, 95% CI)	0.80 [0.20, 3.29]

**Comparison 33:** LEV vs PB

Outcome or subgroup title	No. of studies	No. of participants	Statistical method	Effect size
33.1 LEV vs PB: All Major Malformations	6		Risk Ratio (M‐H, Fixed, 95% CI)	Subtotals only
33.1.1 LEV vs PB (cohort studies)	5	1067	Risk Ratio (M‐H, Fixed, 95% CI)	0.54 [0.29, 1.02]
33.1.2 LEV vs PB (database studies)	1	145	Risk Ratio (M‐H, Fixed, 95% CI)	0.23 [0.03, 1.55]
33.2 LEV vs PB: Neural Tube Malformations	5		Risk Ratio (M‐H, Fixed, 95% CI)	Subtotals only
33.2.1 LEV vs PB (cohort studies)	5	994	Risk Ratio (M‐H, Fixed, 95% CI)	0.74 [0.08, 6.51]
33.3 LEV vs PB: Cardiac Malformations	5		Risk Ratio (M‐H, Fixed, 95% CI)	Subtotals only
33.3.1 LEV vs PB (cohort studies)	5	994	Risk Ratio (M‐H, Fixed, 95% CI)	0.33 [0.12, 0.88]
33.4 LEV vs PB: Oro‐Facial Cleft/Craniofacial Malformations	4		Risk Ratio (M‐H, Fixed, 95% CI)	Subtotals only
33.4.1 LEV vs PB (cohort studies)	4	751	Risk Ratio (M‐H, Fixed, 95% CI)	0.08 [0.01, 0.67]
33.5 LEV vs PB: Skeletal/Limb Malformation	5		Risk Ratio (M‐H, Fixed, 95% CI)	Subtotals only
33.5.1 LEV vs PB (cohort studies)	5	994	Risk Ratio (M‐H, Fixed, 95% CI)	0.67 [0.15, 2.94]

**Comparison 34:** LEV vs PHT

Outcome or subgroup title	No. of studies	No. of participants	Statistical method	Effect size
34.1 LEV vs PHT: All Major Malformations	5		Risk Ratio (M‐H, Fixed, 95% CI)	Subtotals only
34.1.1 LEV vs PHT (cohort studies)	5	1705	Risk Ratio (M‐H, Fixed, 95% CI)	0.58 [0.34, 0.97]
34.2 LEV vs PHT: Neural Tube Malformations	4		Risk Ratio (M‐H, Fixed, 95% CI)	Subtotals only
34.2.1 LEV vs PHT (cohort studies)	4	1574	Risk Ratio (M‐H, Fixed, 95% CI)	0.68 [0.13, 3.44]
34.3 LEV vs PHT: Cardiac Malformations	4		Risk Ratio (M‐H, Fixed, 95% CI)	Subtotals only
34.3.1 LEV vs PHT (cohort studies)	4	1572	Risk Ratio (M‐H, Fixed, 95% CI)	0.43 [0.16, 1.13]
34.4 LEV vs PHT: Oro‐Facial Cleft/Craniofacial Malformations	3		Risk Ratio (M‐H, Fixed, 95% CI)	Subtotals only
34.4.1 LEV vs PHT (cohort studies)	3	1349	Risk Ratio (M‐H, Fixed, 95% CI)	0.37 [0.09, 1.61]
34.5 LEV vs PHT: Skeletal/Limb Malformations	4		Risk Ratio (M‐H, Fixed, 95% CI)	Subtotals only
34.5.1 LEV vs PHT (cohort studies)	4	1574	Risk Ratio (M‐H, Fixed, 95% CI)	0.46 [0.11, 1.96]

**Comparison 35:** LEV vs PRM

Outcome or subgroup title	No. of studies	No. of participants	Statistical method	Effect size
35.1 LEV vs PRM: All Major Malformations	1		Risk Ratio (M‐H, Fixed, 95% CI)	Subtotals only
35.1.1 LEV vs PRM (cohort studies)	1	141	Risk Ratio (M‐H, Fixed, 95% CI)	0.24 [0.02, 3.37]

**Comparison 36:** LEV vs TPM

Outcome or subgroup title	No. of studies	No. of participants	Statistical method	Effect size
36.1 LEV vs TPM: All Major Malformations	9		Risk Ratio (M‐H, Fixed, 95% CI)	Subtotals only
36.1.1 LEV vs TPM (cohort studies)	8	1629	Risk Ratio (M‐H, Fixed, 95% CI)	0.57 [0.32, 1.04]
36.1.2 LEV vs TPM (database studies)	1	166	Risk Ratio (M‐H, Fixed, 95% CI)	0.41 [0.06, 2.81]
36.2 LEV vs TPM: Neural Tube Malformations	7		Risk Ratio (M‐H, Fixed, 95% CI)	Subtotals only
36.2.1 LEV vs TPM (cohort studies)	7	1526	Risk Ratio (M‐H, Fixed, 95% CI)	2.39 [0.10, 58.61]
36.3 LEV vs TPM: Cardiac Malformations	8		Risk Ratio (M‐H, Fixed, 95% CI)	Subtotals only
36.3.1 LEV vs TPM (cohort studies)	8	1536	Risk Ratio (M‐H, Fixed, 95% CI)	0.72 [0.21, 2.53]
36.4 LEV vs TPM: Oro‐Facial Cleft/Craniofacial Malformations	7		Risk Ratio (M‐H, Fixed, 95% CI)	Subtotals only
36.4.1 LEV vs TPM (cohort studies)	7	1421	Risk Ratio (M‐H, Fixed, 95% CI)	0.19 [0.05, 0.70]
36.5 LEV vs TPM: Skeletal/Limb Malformations	7		Risk Ratio (M‐H, Fixed, 95% CI)	Subtotals only
36.5.1 LEV vs TPM (cohort studies)	7	1526	Risk Ratio (M‐H, Fixed, 95% CI)	0.12 [0.02, 0.98]

**Comparison 37:** LEV vs ZNS

Outcome or subgroup title	No. of studies	No. of participants	Statistical method	Effect size
37.1 LEV vs ZNS: All Major Malformations	4		Risk Ratio (M‐H, Fixed, 95% CI)	Subtotals only
37.1.1 LEV vs ZNS (cohort studies)	4	995	Risk Ratio (M‐H, Fixed, 95% CI)	0.66 [0.25, 1.71]
37.2 LEV vs ZNS: Neural Tube Malformations	3		Risk Ratio (M‐H, Fixed, 95% CI)	Subtotals only
37.2.1 LEV vs ZNS (cohort studies)	3	455	Risk Ratio (M‐H, Fixed, 95% CI)	0.03 [0.00, 0.71]
37.3 LEV vs ZNS: Cardiac Malformations	3		Risk Ratio (M‐H, Fixed, 95% CI)	Subtotals only
37.3.1 LEV vs ZNS (cohort studies)	3	455	Risk Ratio (M‐H, Fixed, 95% CI)	0.98 [0.05, 17.99]
37.4 LEV vs ZNS: Oro‐Facial Cleft/Craniofacial Malformations	3		Risk Ratio (M‐H, Fixed, 95% CI)	Subtotals only
37.4.1 LEV vs ZNS (cohort studies)	3	0	Risk Ratio (M‐H, Fixed, 95% CI)	Not estimable
37.5 LEV vs ZNS: Skeletal/Limb Malformations	3		Risk Ratio (M‐H, Fixed, 95% CI)	Subtotals only
37.5.1 LEV vs ZNS (cohort studies)	3	0	Risk Ratio (M‐H, Fixed, 95% CI)	Not estimable

**Comparison 38:** LTG vs CZP

Outcome or subgroup title	No. of studies	No. of participants	Statistical method	Effect size
38.1 LTG vs CZP: All Major Malformations	5		Risk Ratio (M‐H, Fixed, 95% CI)	Subtotals only
38.1.1 LTG vs CZP (cohort studies)	3	2112	Risk Ratio (M‐H, Fixed, 95% CI)	0.92 [0.29, 2.91]
38.1.2 LTG vs CZP (database studies)	2	1084	Risk Ratio (M‐H, Fixed, 95% CI)	1.54 [0.53, 4.54]

**Comparison 39:** LTG vs LAC

Outcome or subgroup title	No. of studies	No. of participants	Statistical method	Effect size
39.1 LTG vs LAC: All Major Malformations	1		Risk Ratio (M‐H, Fixed, 95% CI)	Subtotals only
39.1.1 LTG vs LAC (cohort studies)	1	0	Risk Ratio (M‐H, Fixed, 95% CI)	Not estimable
39.2 LTG vs LAC: Neural Tube Malformations	1		Risk Ratio (M‐H, Fixed, 95% CI)	Subtotals only
39.2.1 LTG vs LAC (cohort studies)	1	0	Risk Ratio (M‐H, Fixed, 95% CI)	Not estimable
39.3 LTG vs LAC: Cardiac Malformations	1		Risk Ratio (M‐H, Fixed, 95% CI)	Subtotals only
39.3.1 LTG vs LAC (cohort studies)	1	0	Risk Ratio (M‐H, Fixed, 95% CI)	Not estimable
39.4 LTG vs LAC: Oro‐Facial Cleft/Craniofacial Malformations	1		Risk Ratio (M‐H, Fixed, 95% CI)	Subtotals only
39.4.1 LTG vs LAC (cohort studies)	1	0	Risk Ratio (M‐H, Fixed, 95% CI)	Not estimable
39.5 LTG vs LAC: Skeletal/Limb Malformations	1		Risk Ratio (M‐H, Fixed, 95% CI)	Subtotals only
39.5.1 LTG vs LAC (cohort studies)	1	0	Risk Ratio (M‐H, Fixed, 95% CI)	Not estimable

**Comparison 40:** LTG vs OXC

Outcome or subgroup title	No. of studies	No. of participants	Statistical method	Effect size
40.1 LTG vs OXC: All Major Malformations	11		Risk Ratio (M‐H, Fixed, 95% CI)	Subtotals only
40.1.1 LTG vs OXC (cohort studies)	8	2541	Risk Ratio (M‐H, Fixed, 95% CI)	0.73 [0.33, 1.62]
40.1.2 LTG vs OXC (database studies)	3	2535	Risk Ratio (M‐H, Fixed, 95% CI)	1.24 [0.67, 2.30]
40.2 LTG vs OXC: Neural Tube Malformations	6		Risk Ratio (M‐H, Fixed, 95% CI)	Subtotals only
40.2.1 LTG vs OXC (cohort studies)	6	2346	Risk Ratio (M‐H, Fixed, 95% CI)	0.59 [0.03, 12.15]
40.3 LTG vs OXC: Cardiac Malformation	8		Risk Ratio (M‐H, Fixed, 95% CI)	Subtotals only
40.3.1 LTG vs OXC (cohort studies)	8	2407	Risk Ratio (M‐H, Fixed, 95% CI)	0.59 [0.15, 2.30]
40.4 LTG vs OXC: Oro‐Facial Cleft/Craniofacial Malformations	6		Risk Ratio (M‐H, Fixed, 95% CI)	Subtotals only
40.4.1 LTG vs OXC (cohort studies)	6	2248	Risk Ratio (M‐H, Fixed, 95% CI)	0.64 [0.12, 3.46]
40.5 LTG vs OXC: Skeletal/Limb Malformation	6		Risk Ratio (M‐H, Fixed, 95% CI)	Subtotals only
40.5.1 LTG vs OXC (cohort studies)	6	2346	Risk Ratio (M‐H, Fixed, 95% CI)	0.29 [0.06, 1.56]

**Comparison 41:** LTG vs PB

Outcome or subgroup title	No. of studies	No. of participants	Statistical method	Effect size
41.1 LTG vs PB: All Major Malformations	9		Risk Ratio (M‐H, Fixed, 95% CI)	Subtotals only
41.1.1 LTG vs PB (cohort studies)	7	2577	Risk Ratio (M‐H, Fixed, 95% CI)	0.32 [0.17, 0.59]
41.1.2 LTG vs PB (database studies)	2	957	Risk Ratio (M‐H, Fixed, 95% CI)	0.41 [0.13, 1.28]
41.2 LTG vs PB: Neural Tube Malformations	6		Risk Ratio (M‐H, Fixed, 95% CI)	Subtotals only
41.2.1 LTG vs PB (cohort studies)	6	2422	Risk Ratio (M‐H, Fixed, 95% CI)	0.76 [0.09, 6.88]
41.3 LTG vs PB: Cardiac Malformations	5		Risk Ratio (M‐H, Fixed, 95% CI)	Subtotals only
41.3.1 LTG vs PB (cohort studies)	5	2401	Risk Ratio (M‐H, Fixed, 95% CI)	0.21 [0.08, 0.56]
41.4 LTG vs PB: Oro‐Facial Cleft/Craniofacial Malformations	4		Risk Ratio (M‐H, Fixed, 95% CI)	Subtotals only
41.4.1 LTG vs PB (cohort studies)	4	2214	Risk Ratio (M‐H, Fixed, 95% CI)	0.22 [0.07, 0.68]
41.5 LTG vs PB: Skeletal/Limb Malformations	6		Risk Ratio (M‐H, Fixed, 95% CI)	Subtotals only
41.5.1 LTG vs PB (cohort studies)	6	2422	Risk Ratio (M‐H, Fixed, 95% CI)	0.38 [0.06, 2.58]

**Comparison 42:** LTG vs PHT

Outcome or subgroup title	No. of studies	No. of participants	Statistical method	Effect size
42.1 LTG vs PHT: All Major Malformations	7		Risk Ratio (M‐H, Fixed, 95% CI)	Subtotals only
42.1.1 LTG vs PHT (cohort studies)	6	4993	Risk Ratio (M‐H, Fixed, 95% CI)	0.55 [0.35, 0.87]
42.1.2 LTG vs PHT (database studies)	1	193	Risk Ratio (M‐H, Fixed, 95% CI)	0.65 [0.20, 2.16]
42.2 LTG vs PHT: Neural Tube Malformations	6		Risk Ratio (M‐H, Fixed, 95% CI)	Subtotals only
42.2.1 LTG vs PHT (cohort studies)	6	4845	Risk Ratio (M‐H, Fixed, 95% CI)	0.40 [0.11, 1.51]
42.3 LTG vs PHT: Cardiac Malformations	6		Risk Ratio (M‐H, Fixed, 95% CI)	Subtotals only
42.3.1 LTG vs PHT (cohort studies)	6	4845	Risk Ratio (M‐H, Fixed, 95% CI)	0.41 [0.17, 0.98]
42.4 LTG vs PHT: Oro‐Facial Cleft/Craniofacial Malformations	5		Risk Ratio (M‐H, Fixed, 95% CI)	Subtotals only
42.4.1 LTG vs PHT (cohort studies)	5	4676	Risk Ratio (M‐H, Fixed, 95% CI)	0.73 [0.23, 2.28]
42.5 LTG vs PHT: Skeletal/Limb Malformations	6		Risk Ratio (M‐H, Fixed, 95% CI)	Subtotals only
42.5.1 LTG vs PHT (cohort studies)	6	4845	Risk Ratio (M‐H, Fixed, 95% CI)	0.28 [0.09, 0.86]

**Comparison 43:** LTG vs PRM

Outcome or subgroup title	No. of studies	No. of participants	Statistical method	Effect size
43.1 LTG vs PRM: All Major Malformations	2		Risk Ratio (M‐H, Fixed, 95% CI)	Subtotals only
43.1.1 LTG vs PRM (cohort studies)	1	408	Risk Ratio (M‐H, Fixed, 95% CI)	0.30 [0.02, 3.93]
43.1.2 LTG vs PRM (database studies)	1	93	Risk Ratio (M‐H, Fixed, 95% CI)	0.40 [0.03, 6.16]

**Comparison 44:** LTG vs TPM

Outcome or subgroup title	No. of studies	No. of participants	Statistical method	Effect size
44.1 LTG vs TPM: All Major Malformations	10		Risk Ratio (M‐H, Fixed, 95% CI)	Subtotals only
44.1.1 LTG vs TPM (cohort studies)	8	4780	Risk Ratio (M‐H, Fixed, 95% CI)	0.59 [0.36, 0.96]
44.1.2 LTG vs TPM (database studies)	2	972	Risk Ratio (M‐H, Fixed, 95% CI)	0.68 [0.20, 2.37]
44.2 LTG vs TPM: Neural Tube Malformations	7		Risk Ratio (M‐H, Fixed, 95% CI)	Subtotals only
44.2.1 LTG vs TPM (cohort studies)	7	4627	Risk Ratio (M‐H, Fixed, 95% CI)	0.62 [0.08, 4.94]
44.3 LTG vs TPM: Cardiac Malformations	8		Risk Ratio (M‐H, Fixed, 95% CI)	Subtotals only
44.3.1 LTG vs TPM (cohort studies)	8	4648	Risk Ratio (M‐H, Fixed, 95% CI)	0.58 [0.19, 1.81]
44.4 LTG vs TPM: Oro‐Facial Cleft/Craniofacial Malformations	7		Risk Ratio (M‐H, Random, 95% CI)	Subtotals only
44.4.1 LTG vs TPM (cohort studies)	7	4589	Risk Ratio (M‐H, Random, 95% CI)	0.22 [0.03, 1.48]
44.5 LTG vs TPM: Skeletal/Limb Malformations	7		Risk Ratio (M‐H, Fixed, 95% CI)	Subtotals only
44.5.1 LTG vs TPM (cohort studies)	7	4627	Risk Ratio (M‐H, Fixed, 95% CI)	0.17 [0.06, 0.52]

**Comparison 45:** LTG vs ZNS

Outcome or subgroup title	No. of studies	No. of participants	Statistical method	Effect size
45.1 LTG vs ZNS: All Major Malformations	4		Risk Ratio (M‐H, Fixed, 95% CI)	Subtotals only
45.1.1 LTG vs ZNS (cohort studies)	4	3922	Risk Ratio (M‐H, Fixed, 95% CI)	0.66 [0.26, 1.65]
45.2 LTG vs ZNS: Neural Tube Malformations	3		Risk Ratio (M‐H, Fixed, 95% CI)	Subtotals only
45.2.1 LTG vs ZNS (cohort studies)	3	2270	Risk Ratio (M‐H, Fixed, 95% CI)	0.02 [0.00, 0.26]
45.3 LTG vs ZNS: Cardiac Malformations	2		Risk Ratio (M‐H, Fixed, 95% CI)	Subtotals only
45.3.1 LTG vs ZNS (cohort studies)	2	2250	Risk Ratio (M‐H, Fixed, 95% CI)	0.30 [0.04, 2.52]
45.4 LTG vs ZNS: Oro‐Facial Cleft/Craniofacial Malformations	2		Risk Ratio (M‐H, Fixed, 95% CI)	Subtotals only
45.4.1 LTG vs ZNS (cohort studies)	2	2250	Risk Ratio (M‐H, Fixed, 95% CI)	0.06 [0.00, 1.31]
45.5 LTG vs ZNS: Skeletal/Limb Malformations	3		Risk Ratio (M‐H, Fixed, 95% CI)	Subtotals only
45.5.1 LTG vs ZNS (cohort studies)	3	2270	Risk Ratio (M‐H, Fixed, 95% CI)	0.22 [0.03, 1.93]

**Comparison 46:** PHT vs GBP

Outcome or subgroup title	No. of studies	No. of participants	Statistical method	Effect size
46.1 PHT vs GBP: All Major Malformations	5		Risk Ratio (M‐H, Fixed, 95% CI)	Subtotals only
46.1.1 PHT vs GBP (cohort studies)	4	759	Risk Ratio (M‐H, Fixed, 95% CI)	2.15 [0.69, 6.73]
46.1.2 PHT vs GBP (database studies)	1	121	Risk Ratio (M‐H, Fixed, 95% CI)	2.74 [0.16, 46.00]
46.2 PHT vs GBP: Neural Tube Malformations	2		Risk Ratio (M‐H, Fixed, 95% CI)	Subtotals only
46.2.1 PHT vs GBP (cohort studies)	2	61	Risk Ratio (M‐H, Fixed, 95% CI)	1.00 [0.04, 23.26]
46.3 PHT vs GBP: Cardiac Malformations	2		Risk Ratio (M‐H, Fixed, 95% CI)	Subtotals only
46.3.1 PHT vs GBP (cohort studies)	2	61	Risk Ratio (M‐H, Fixed, 95% CI)	0.69 [0.09, 5.17]
46.4 PHT vs GBP: Oro‐Facial Cleft/Craniofacial Malformations	2		Risk Ratio (M‐H, Fixed, 95% CI)	Subtotals only
46.4.1 PHT vs GBP (cohort studies)	2	0	Risk Ratio (M‐H, Fixed, 95% CI)	Not estimable
46.5 PHT vs GBP: Skeletal/Limb Malformations	2		Risk Ratio (M‐H, Fixed, 95% CI)	Subtotals only
46.5.1 PHT vs GBP (cohort studies)	2	0	Risk Ratio (M‐H, Fixed, 95% CI)	Not estimable

**Comparison 47:** PHT vs OXC

Outcome or subgroup title	No. of studies	No. of participants	Statistical method	Effect size
47.1 PHT vs OXC: All Major Malformations	7		Risk Ratio (M‐H, Fixed, 95% CI)	Subtotals only
47.1.1 PHT vs OXC (cohort studies)	6	989	Risk Ratio (M‐H, Fixed, 95% CI)	0.94 [0.48, 1.85]
47.1.2 PHT vs OXC (database studies)	1	107	Risk Ratio (M‐H, Fixed, 95% CI)	0.72 [0.05, 10.93]
47.2 PHT vs OXC: Neural Tube Malformations	4		Risk Ratio (M‐H, Fixed, 95% CI)	Subtotals only
47.2.1 PHT vs OXC (cohort studies)	4	974	Risk Ratio (M‐H, Fixed, 95% CI)	1.16 [0.13, 10.29]
47.3 PHT vs OXC: Cardiac Malformations	5		Risk Ratio (M‐H, Fixed, 95% CI)	Subtotals only
47.3.1 PHT vs OXC (cohort studies)	5	976	Risk Ratio (M‐H, Fixed, 95% CI)	1.33 [0.43, 4.17]
47.4 PHT vs OXC: Oro‐Facial Cleft/Craniofacial Malformations	3		Risk Ratio (M‐H, Fixed, 95% CI)	Subtotals only
47.4.1 PHT vs OXC (cohort studies)	3	784	Risk Ratio (M‐H, Fixed, 95% CI)	0.62 [0.10, 4.05]
47.5 PHT vs OXC: Skeletal/Limb Malformations	4		Risk Ratio (M‐H, Fixed, 95% CI)	Subtotals only
47.5.1 PHT vs OXC (cohort studies)	4	974	Risk Ratio (M‐H, Fixed, 95% CI)	1.20 [0.23, 6.35]

**Comparison 48:** PHT vs PB

Outcome or subgroup title	No. of studies	No. of participants	Statistical method	Effect size
48.1 PHT vs PB: All Major Malformations	21		Risk Ratio (M‐H, Fixed, 95% CI)	Subtotals only
48.1.1 PHT vs PB (cohort studies)	20	1729	Risk Ratio (M‐H, Fixed, 95% CI)	0.84 [0.57, 1.23]
48.1.2 PHT vs PB (database studies)	1	110	Risk Ratio (M‐H, Fixed, 95% CI)	0.48 [0.07, 3.35]
48.2 PHT vs PB: Neural Tube Malformations	11		Risk Ratio (M‐H, Fixed, 95% CI)	Subtotals only
48.2.1 PHT vs PB (cohort studies)	11	1182	Risk Ratio (M‐H, Fixed, 95% CI)	0.79 [0.10, 5.94]
48.3 PHT vs PB: Cardiac Malformations	11		Risk Ratio (M‐H, Fixed, 95% CI)	Subtotals only
48.3.1 PHT vs PB (cohort studies)	11	1182	Risk Ratio (M‐H, Fixed, 95% CI)	0.56 [0.29, 1.07]
48.4 PHT vs PB: Oro‐Facial Cleft/Craniofacial Malformations	11		Risk Ratio (M‐H, Fixed, 95% CI)	Subtotals only
48.4.1 PHT vs PB (cohort studies)	11	940	Risk Ratio (M‐H, Fixed, 95% CI)	0.25 [0.07, 0.82]
48.5 PHT vs PB: Skeletal/Limb Malformations	11		Risk Ratio (M‐H, Fixed, 95% CI)	Subtotals only
48.5.1 PHT vs PB (cohort studies)	11	1182	Risk Ratio (M‐H, Fixed, 95% CI)	1.31 [0.39, 4.39]

**Comparison 49:** PHT vs PRM

Outcome or subgroup title	No. of studies	No. of participants	Statistical method	Effect size
49.1 PHT vs PRM: All Major Malformations	7		Risk Ratio (M‐H, Fixed, 95% CI)	Subtotals only
49.1.1 PHT vs PRM (cohort studies)	6	463	Risk Ratio (M‐H, Fixed, 95% CI)	0.78 [0.39, 1.56]
49.1.2 PHT vs PRM (database studies)	1	106	Risk Ratio (M‐H, Fixed, 95% CI)	0.58 [0.04, 8.44]
49.2 PHT vs PRM: Neural Tube Malformations	2		Risk Ratio (M‐H, Fixed, 95% CI)	Subtotals only
49.2.1 PHT vs PRM (cohort studies)	2	0	Risk Ratio (M‐H, Fixed, 95% CI)	Not estimable
49.3 PHT vs PRM: Cardiac Malformations	2		Risk Ratio (M‐H, Fixed, 95% CI)	Subtotals only
49.3.1 PHT vs PRM (cohort studies)	2	75	Risk Ratio (M‐H, Fixed, 95% CI)	0.38 [0.02, 8.88]
49.4 PHT vs PRM: Oro‐Facial Cleft/Craniofacial Malformations	2		Risk Ratio (M‐H, Fixed, 95% CI)	Subtotals only
49.4.1 PHT vs PRM (cohort studies)	2	0	Risk Ratio (M‐H, Fixed, 95% CI)	Not estimable
49.5 PHT vs PRM: Skeletal/Limb Malformations	2		Risk Ratio (M‐H, Fixed, 95% CI)	Subtotals only
49.5.1 PHT vs PRM (cohort studies)	2	75	Risk Ratio (M‐H, Fixed, 95% CI)	3.38 [0.14, 79.95]

**Comparison 50:** PHT vs TPM

Outcome or subgroup title	No. of studies	No. of participants	Statistical method	Effect size
50.1 PHT vs TPM: All Major Malformations	5		Risk Ratio (M‐H, Fixed, 95% CI)	Subtotals only
50.1.1 PHT vs TPM (cohort studies)	4	1176	Risk Ratio (M‐H, Fixed, 95% CI)	0.88 [0.48, 1.61]
50.1.2 PHT vs TPM (database studies)	1	104	Risk Ratio (M‐H, Fixed, 95% CI)	0.29 [0.02, 3.51]
50.2 PHT vs TPM: Neural Tube Malformations	4		Risk Ratio (M‐H, Fixed, 95% CI)	Subtotals only
50.2.1 PHT vs TPM (cohort studies)	4	1144	Risk Ratio (M‐H, Fixed, 95% CI)	1.23 [0.17, 8.87]
50.3 PHT vs TPM: Cardiac Malformations	4		Risk Ratio (M‐H, Fixed, 95% CI)	Subtotals only
50.3.1 PHT vs TPM (cohort studies)	4	1144	Risk Ratio (M‐H, Fixed, 95% CI)	2.46 [0.65, 9.36]
50.4 PHT vs TPM: Oro‐Facial Cleft/Craniofacial Malformations	3		Risk Ratio (M‐H, Fixed, 95% CI)	Subtotals only
50.4.1 PHT vs TPM (cohort studies)	3	1016	Risk Ratio (M‐H, Fixed, 95% CI)	0.37 [0.10, 1.42]
50.5 PHT vs TPM: Skeletal/Limb Malformations	4		Risk Ratio (M‐H, Fixed, 95% CI)	Subtotals only
50.5.1 PHT vs TPM (cohort studies)	4	1144	Risk Ratio (M‐H, Fixed, 95% CI)	0.63 [0.19, 2.09]

**Comparison 51:** PHT vs ZNS

Outcome or subgroup title	No. of studies	No. of participants	Statistical method	Effect size
51.1 PHT vs ZNS: All Major Malformations	2		Risk Ratio (M‐H, Fixed, 95% CI)	Subtotals only
51.1.1 PHT vs ZNS (cohort studies)	2	638	Risk Ratio (M‐H, Fixed, 95% CI)	1.28 [0.42, 3.93]
51.2 PHT vs ZNS: Neural Tube Malformations	1		Risk Ratio (M‐H, Fixed, 95% CI)	Subtotals only
51.2.1 PHT vs ZNS (cohort studies)	1	108	Risk Ratio (M‐H, Fixed, 95% CI)	0.11 [0.00, 2.58]
51.3 PHT vs ZNS: Cardiac Malformations	1		Risk Ratio (M‐H, Fixed, 95% CI)	Subtotals only
51.3.1 PHT vs ZNS (cohort studies)	1	108	Risk Ratio (M‐H, Fixed, 95% CI)	0.98 [0.04, 23.26]
51.4 PHT vs ZNS: Oro‐Facial Cleft/Craniofacial Malformations	1		Risk Ratio (M‐H, Fixed, 95% CI)	Subtotals only
51.4.1 PHT vs ZNS (cohort studies)	1	108	Risk Ratio (M‐H, Fixed, 95% CI)	0.98 [0.04, 23.26]
51.5 PHT vs ZNS: Skeletal/Limb Malformations	1		Risk Ratio (M‐H, Fixed, 95% CI)	Subtotals only
51.5.1 PHT vs ZNS (cohort studies)	1	0	Risk Ratio (M‐H, Fixed, 95% CI)	Not estimable

**Comparison 52:** PB vs OXC

Outcome or subgroup title	No. of studies	No. of participants	Statistical method	Effect size
52.1 PB vs OXC: All Major Malformations	10		Risk Ratio (M‐H, Fixed, 95% CI)	Subtotals only
52.1.1 PB vs OXC (cohort studies)	8	676	Risk Ratio (M‐H, Fixed, 95% CI)	1.61 [0.83, 3.14]
52.1.2 PB vs OXC (database studies)	2	95	Risk Ratio (M‐H, Fixed, 95% CI)	3.07 [0.50, 18.92]
52.2 PB vs OXC: Neural Tube Malformations	6		Risk Ratio (M‐H, Fixed, 95% CI)	Subtotals only
52.2.1 PB vs OXC (cohort studies)	6	654	Risk Ratio (M‐H, Fixed, 95% CI)	1.57 [0.06, 37.94]
52.3 PB vs OXC: Cardiac Malformations	7		Risk Ratio (M‐H, Fixed, 95% CI)	Subtotals only
52.3.1 PB vs OXC (cohort studies)	7	658	Risk Ratio (M‐H, Fixed, 95% CI)	2.58 [0.94, 7.09]
52.4 PB vs OXC: Oro‐Facial Cleft/Craniofacial Malformations	5		Risk Ratio (M‐H, Fixed, 95% CI)	Subtotals only
52.4.1 PB vs OXC (cohort studies)	5	446	Risk Ratio (M‐H, Fixed, 95% CI)	3.66 [0.41, 32.43]
52.5 PB vs OXC: Skeletal/Limb Malformations	6		Risk Ratio (M‐H, Fixed, 95% CI)	Subtotals only
52.5.1 PB vs OXC (cohort studies)	6	654	Risk Ratio (M‐H, Fixed, 95% CI)	0.98 [0.16, 5.97]

**Comparison 53:** PB vs PRM

Outcome or subgroup title	No. of studies	No. of participants	Statistical method	Effect size
53.1 PB vs PRM: All Major Malformations	7		Risk Ratio (M‐H, Fixed, 95% CI)	Subtotals only
53.1.1 PB vs PRM (cohort studies)	6	351	Risk Ratio (M‐H, Fixed, 95% CI)	0.50 [0.21, 1.16]
53.1.2 PB vs PRM (database studies)	1	10	Risk Ratio (M‐H, Fixed, 95% CI)	1.50 [0.08, 29.15]
53.2 PB vs PRM: Neural Tube Malformations	2		Risk Ratio (M‐H, Fixed, 95% CI)	Subtotals only
53.2.1 PB vs PRM (cohort studies)	2	0	Risk Ratio (M‐H, Fixed, 95% CI)	Not estimable
53.3 PB vs PRM: Cardiac Malformations	2		Risk Ratio (M‐H, Fixed, 95% CI)	Subtotals only
53.3.1 PB vs PRM (cohort studies)	2	134	Risk Ratio (M‐H, Fixed, 95% CI)	0.42 [0.03, 6.55]
53.4 PB vs PRM: Oro‐Facial Cleft/Craniofacial Malformations	2		Risk Ratio (M‐H, Fixed, 95% CI)	Subtotals only
53.4.1 PB vs PRM (cohort studies)	2	0	Risk Ratio (M‐H, Fixed, 95% CI)	Not estimable
53.5 PB vs PRM: Skeletal/Limb Malformations	2		Risk Ratio (M‐H, Fixed, 95% CI)	Subtotals only
53.5.1 PB vs PRM (cohort studies)	2	134	Risk Ratio (M‐H, Fixed, 95% CI)	1.29 [0.05, 30.82]

**Comparison 54:** PB vs TPM

Outcome or subgroup title	No. of studies	No. of participants	Statistical method	Effect size
54.1 PB vs TPM: All Major Malformations	6		Risk Ratio (M‐H, Fixed, 95% CI)	Subtotals only
54.1.1 PB vs TPM (cohort studies)	4	766	Risk Ratio (M‐H, Fixed, 95% CI)	1.38 [0.68, 2.81]
54.1.2 PB vs TPM (database studies)	2	83	Risk Ratio (M‐H, Fixed, 95% CI)	1.41 [0.30, 6.68]
54.2 PB vs TPM: Neural Tube Malformations	4		Risk Ratio (M‐H, Fixed, 95% CI)	Subtotals only
54.2.1 PB vs TPM (cohort studies)	4	760	Risk Ratio (M‐H, Fixed, 95% CI)	0.22 [0.01, 5.00]
54.3 PB vs TPM: Cardiac Malformations	4		Risk Ratio (M‐H, Fixed, 95% CI)	Subtotals only
54.3.1 PB vs TPM (cohort studies)	4	760	Risk Ratio (M‐H, Fixed, 95% CI)	4.44 [0.98, 20.12]
54.4 PB vs TPM: Oro‐Facial Cleft/Craniofacial Malformations	3		Risk Ratio (M‐H, Fixed, 95% CI)	Subtotals only
54.4.1 PB vs TPM (cohort studies)	3	614	Risk Ratio (M‐H, Fixed, 95% CI)	1.44 [0.39, 5.31]
54.5 PB vs TPM: Skeletal/Limb Malformations	4		Risk Ratio (M‐H, Fixed, 95% CI)	Subtotals only
54.5.1 PB vs TPM (cohort studies)	4	760	Risk Ratio (M‐H, Fixed, 95% CI)	0.36 [0.06, 2.19]

**Comparison 55:** PB vs ZNS

Outcome or subgroup title	No. of studies	No. of participants	Statistical method	Effect size
55.1 PB vs ZNS: All Major Malformations	2		Risk Ratio (M‐H, Fixed, 95% CI)	Subtotals only
55.1.1 PB vs ZNS (cohort studies)	2	292	Risk Ratio (M‐H, Fixed, 95% CI)	10.46 [0.62, 175.67]
55.2 PB vs ZNS: Neural Tube Malformations	1	0	Risk Ratio (M‐H, Fixed, 95% CI)	Not estimable
55.2.1 PB vs ZNS (cohort studies)	1	0	Risk Ratio (M‐H, Fixed, 95% CI)	Not estimable
55.3 PB vs ZNS: Cardiac Malformations	1	0	Risk Ratio (M‐H, Fixed, 95% CI)	Not estimable
55.3.1 PB vs ZNS (cohort studies)	1	0	Risk Ratio (M‐H, Fixed, 95% CI)	Not estimable
55.4 PB vs ZNS: Oro‐Facial Cleft/Craniofacial Malformations	1	0	Risk Ratio (M‐H, Fixed, 95% CI)	Not estimable
55.4.1 PB vs ZNS (cohort studies)	1	0	Risk Ratio (M‐H, Fixed, 95% CI)	Not estimable
55.5 PB vs ZNS: Skeletal/Limb Malformations	1	0	Risk Ratio (M‐H, Fixed, 95% CI)	Not estimable
55.5.1 PB vs ZNS (cohort studies)	1	0	Risk Ratio (M‐H, Fixed, 95% CI)	Not estimable

**Comparison 56:** TPM vs ZNS

Outcome or subgroup title	No. of studies	No. of participants	Statistical method	Effect size
56.1 TPM vs ZNS: All Major Malformations	4		Risk Ratio (M‐H, Fixed, 95% CI)	Subtotals only
56.1.1 TPM vs ZNS (cohort studies)	4	570	Risk Ratio (M‐H, Fixed, 95% CI)	1.59 [0.54, 4.66]
56.2 TPM vs ZNS: Neural Tube Malformations	2		Risk Ratio (M‐H, Fixed, 95% CI)	Subtotals only
56.2.1 TPM vs ZNS (cohort studies)	2	0	Risk Ratio (M‐H, Fixed, 95% CI)	Not estimable
56.3 TPM vs ZNS: Cardiac Malformations	3		Risk Ratio (M‐H, Fixed, 95% CI)	Subtotals only
56.3.1 TPM vs ZNS (cohort studies)	3	121	Risk Ratio (M‐H, Fixed, 95% CI)	6.00 [0.28, 129.16]
56.4 TPM vs ZNS: Oro‐Facial Cleft/Craniofacial Malformations	3		Risk Ratio (M‐H, Fixed, 95% CI)	Subtotals only
56.4.1 TPM vs ZNS (cohort studies)	3	121	Risk Ratio (M‐H, Fixed, 95% CI)	1.90 [0.09, 38.34]
56.5 TPM vs ZNS: Skeletal/Limb Malformations	3		Risk Ratio (M‐H, Fixed, 95% CI)	Subtotals only
56.5.1 TPM vs ZNS (cohort studies)	3	0	Risk Ratio (M‐H, Fixed, 95% CI)	Not estimable

**Comparison 57:** TPM vs LAC

Outcome or subgroup title	No. of studies	No. of participants	Statistical method	Effect size
57.1 TPM vs LAC: All Major Malformations	1		Risk Ratio (M‐H, Fixed, 95% CI)	Subtotals only
57.1.1 TPM vs LAC (cohort studies)	1	0	Risk Ratio (M‐H, Fixed, 95% CI)	Not estimable
57.2 TPM vs LAC: Neural Tube Malformations	1		Risk Ratio (M‐H, Fixed, 95% CI)	Subtotals only
57.2.1 TPM vs LAC (cohort studies)	1	0	Risk Ratio (M‐H, Fixed, 95% CI)	Not estimable
57.3 TPM vs LAC: Cardiac Malformations	1		Risk Ratio (M‐H, Fixed, 95% CI)	Subtotals only
57.3.1 TPM vs LAC (cohort studies)	1	0	Risk Ratio (M‐H, Fixed, 95% CI)	Not estimable
57.4 TPM vs LAC: Oro‐Facial Cleft/Craniofacial Malformations	1		Risk Ratio (M‐H, Fixed, 95% CI)	Subtotals only
57.4.1 TPM vs LAC (cohort studies)	1	0	Risk Ratio (M‐H, Fixed, 95% CI)	Not estimable
57.5 TPM vs LAC: Skeletal/Limb Malformations	1		Risk Ratio (M‐H, Fixed, 95% CI)	Subtotals only
57.5.1 TPM vs LAC (cohort studies)	1	0	Risk Ratio (M‐H, Fixed, 95% CI)	Not estimable

**Comparison 58:** VPA vs GBP

Outcome or subgroup title	No. of studies	No. of participants	Statistical method	Effect size
58.1 VPA vs GBP: All Major Malformations	5		Risk Ratio (M‐H, Fixed, 95% CI)	Subtotals only
58.1.1 VPA vs GBP (cohort studies)	4	2031	Risk Ratio (M‐H, Fixed, 95% CI)	4.27 [1.60, 11.35]
58.1.2 VPA vs GBP (database studies)	1	286	Risk Ratio (M‐H, Fixed, 95% CI)	3.74 [0.24, 59.08]
58.2 VPA vs GBP: Neural Tube Malformations	2		Risk Ratio (M‐H, Fixed, 95% CI)	Subtotals only
58.2.1 VPA vs GBP (cohort studies)	2	293	Risk Ratio (M‐H, Fixed, 95% CI)	0.83 [0.05, 13.81]
58.3 VPA vs GBP: Cardiac Malformations	2		Risk Ratio (M‐H, Fixed, 95% CI)	Subtotals only
58.3.1 VPA vs GBP (cohort studies)	2	293	Risk Ratio (M‐H, Fixed, 95% CI)	0.46 [0.08, 2.70]
58.4 VPA vs GBP: Oro‐Facial Cleft/Craniofacial Malformations	2		Risk Ratio (M‐H, Fixed, 95% CI)	Subtotals only
58.4.1 VPA vs GBP (cohort studies)	2	293	Risk Ratio (M‐H, Fixed, 95% CI)	1.38 [0.09, 22.19]
58.5 VPA vs GBP: Skeletal/Limb Malformations	2		Risk Ratio (M‐H, Fixed, 95% CI)	Subtotals only
58.5.1 VPA vs GBP (cohort studies)	2	293	Risk Ratio (M‐H, Fixed, 95% CI)	0.72 [0.04, 12.14]

**Comparison 59:** VPA vs LAC

Outcome or subgroup title	No. of studies	No. of participants	Statistical method	Effect size
59.1 VPA vs LAC: All Major Malformations	1		Risk Ratio (M‐H, Fixed, 95% CI)	Subtotals only
59.1.1 VPA vs LAC (cohort studies)	1	18	Risk Ratio (M‐H, Fixed, 95% CI)	1.00 [0.08, 12.56]
59.2 VPA vs LAC: Neural Tube Malformations	1		Risk Ratio (M‐H, Fixed, 95% CI)	Subtotals only
59.2.1 VPA vs LAC (cohort studies)	1	18	Risk Ratio (M‐H, Fixed, 95% CI)	0.33 [0.02, 5.75]
59.3 VPA vs LAC: Cardiac Malformations	1		Risk Ratio (M‐H, Fixed, 95% CI)	Subtotals only
59.3.1 VPA vs LAC (cohort studies)	1	18	Risk Ratio (M‐H, Fixed, 95% CI)	0.33 [0.02, 5.75]
59.4 VPA vs LAC: Oro‐Facial Cleft/Craniofacial Malformations	1		Risk Ratio (M‐H, Fixed, 95% CI)	Subtotals only
59.4.1 VPA vs LAC (cohort studies)	1	18	Risk Ratio (M‐H, Fixed, 95% CI)	0.33 [0.02, 5.75]
59.5 VPA vs LAC: Skeletal/Limb Malformations	1		Risk Ratio (M‐H, Fixed, 95% CI)	Subtotals only
59.5.1 VPA vs LAC (cohort studies)	1	0	Risk Ratio (M‐H, Fixed, 95% CI)	Not estimable

**Comparison 60:** VPA vs LEV

Outcome or subgroup title	No. of studies	No. of participants	Statistical method	Effect size
60.1 VPA vs LEV: All Major Malformations	12		Risk Ratio (M‐H, Fixed, 95% CI)	Subtotals only
60.1.1 VPA vs LEV (cohort studies)	10	3485	Risk Ratio (M‐H, Fixed, 95% CI)	3.77 [2.48, 5.74]
60.1.2 VPA vs LEV (database studies)	2	911	Risk Ratio (M‐H, Fixed, 95% CI)	3.26 [1.51, 7.03]
60.2 VPA vs LEV: Neural Tube Malformations	9		Risk Ratio (M‐H, Fixed, 95% CI)	Subtotals only
60.2.1 VPA vs LEV (cohort studies)	9	3346	Risk Ratio (M‐H, Fixed, 95% CI)	3.76 [1.22, 11.55]
60.3 VPA vs LEV: Cardiac Malformations	10		Risk Ratio (M‐H, Fixed, 95% CI)	Subtotals only
60.3.1 VPA vs LEV (cohort studies)	10	3356	Risk Ratio (M‐H, Fixed, 95% CI)	3.04 [1.46, 6.34]
60.4 VPA vs LEV: Oro‐Facial Cleft/Craniofacial Malformations	9		Risk Ratio (M‐H, Fixed, 95% CI)	Subtotals only
60.4.1 VPA vs LEV (cohort studies)	9	2909	Risk Ratio (M‐H, Fixed, 95% CI)	3.75 [1.19, 11.77]
60.5 VPA vs LEV: Skeletal/Limb Malformations	9		Risk Ratio (M‐H, Fixed, 95% CI)	Subtotals only
60.5.1 VPA vs LEV (cohort studies)	9	3346	Risk Ratio (M‐H, Fixed, 95% CI)	2.41 [0.99, 5.85]

**Comparison 61:** VPA vs LTG

Outcome or subgroup title	No. of studies	No. of participants	Statistical method	Effect size
61.1 VPA vs LTG: All Major Malformations	16		Risk Ratio (M‐H, Fixed, 95% CI)	Subtotals only
61.1.1 VPA vs LTG (cohort studies)	12	6896	Risk Ratio (M‐H, Fixed, 95% CI)	3.50 [2.76, 4.46]
61.1.2 VPA vs LTG (database studies)	4	3590	Risk Ratio (M‐H, Fixed, 95% CI)	2.49 [1.86, 3.35]
61.2 VPA vs LTG: Neural Tube Malformations	11		Risk Ratio (M‐H, Fixed, 95% CI)	Subtotals only
61.2.1 VPA vs LTG (cohort studies)	11	6708	Risk Ratio (M‐H, Fixed, 95% CI)	7.48 [3.27, 17.13]
61.3 VPA vs LTG: Cardiac Malformations	12		Risk Ratio (M‐H, Fixed, 95% CI)	Subtotals only
61.3.1 VPA vs LTG (cohort studies)	12	6729	Risk Ratio (M‐H, Fixed, 95% CI)	3.39 [2.06, 5.60]
61.4 VPA vs LTG: Oro‐Facial Cleft/Craniofacial Malformations	11		Risk Ratio (M‐H, Fixed, 95% CI)	Subtotals only
61.4.1 VPA vs LTG (cohort studies)	11	6338	Risk Ratio (M‐H, Fixed, 95% CI)	4.16 [2.14, 8.08]
61.5 VPA vs LTG: Skeletal/Limb Malformations	11		Risk Ratio (M‐H, Fixed, 95% CI)	Subtotals only
61.5.1 VPA vs LTG (cohort studies)	11	6708	Risk Ratio (M‐H, Fixed, 95% CI)	6.09 [2.91, 12.76]

**Comparison 62:** VPA vs TPM

Outcome or subgroup title	No. of studies	No. of participants	Statistical method	Effect size
62.1 VPA vs TPM: All Major Malformations	9		Risk Ratio (M‐H, Fixed, 95% CI)	Subtotals only
62.1.1 VPA vs TPM (cohort studies)	7	2723	Risk Ratio (M‐H, Fixed, 95% CI)	2.47 [1.50, 4.08]
62.1.2 VPA vs TPM (database studies)	2	650	Risk Ratio (M‐H, Fixed, 95% CI)	1.27 [0.36, 4.39]
62.2 VPA vs TPM: Neural Tube Malformations	6		Risk Ratio (M‐H, Fixed, 95% CI)	Subtotals only
62.2.1 VPA vs TPM (cohort studies)	6	2665	Risk Ratio (M‐H, Fixed, 95% CI)	2.39 [0.73, 7.80]
62.3 VPA vs TPM: Cardiac Malformations	6		Risk Ratio (M‐H, Fixed, 95% CI)	Subtotals only
62.3.1 VPA vs TPM (cohort studies)	6	2670	Risk Ratio (M‐H, Fixed, 95% CI)	3.48 [1.16, 10.48]
62.4 VPA vs TPM: Oro‐Facial Cleft/Craniofacial Malformations	6		Risk Ratio (M‐H, Fixed, 95% CI)	Subtotals only
62.4.1 VPA vs TPM (cohort studies)	6	2317	Risk Ratio (M‐H, Fixed, 95% CI)	0.89 [0.37, 2.13]
62.5 VPA vs TPM: Skeletal/Limb Malformation	6		Risk Ratio (M‐H, Fixed, 95% CI)	Subtotals only
62.5.1 VPA vs TPM (cohort studies)	6	2689	Risk Ratio (M‐H, Fixed, 95% CI)	1.45 [0.55, 3.82]

**Comparison 63:** VPA vs OXC

Outcome or subgroup title	No. of studies	No. of participants	Statistical method	Effect size
63.1 VPA vs OXC: All Major Malformations	15		Risk Ratio (M‐H, Fixed, 95% CI)	Subtotals only
63.1.1 VPA vs OXC (cohort studies)	11	1561	Risk Ratio (M‐H, Fixed, 95% CI)	2.48 [1.42, 4.31]
63.1.2 VPA vs OXC (database studies)	4	1701	Risk Ratio (M‐H, Fixed, 95% CI)	1.60 [1.11, 2.29]
63.2 VPA vs OXC: Neural Tube Malformations	9		Risk Ratio (M‐H, Fixed, 95% CI)	Subtotals only
63.2.1 VPA vs OXC (cohort studies)	9	1497	Risk Ratio (M‐H, Fixed, 95% CI)	1.55 [0.49, 4.89]
63.3 VPA vs OXC: Cardiac Malformations	11		Risk Ratio (M‐H, Fixed, 95% CI)	Subtotals only
63.3.1 VPA vs OXC (cohort studies)	11	1597	Risk Ratio (M‐H, Fixed, 95% CI)	1.80 [0.84, 3.88]
63.4 VPA vs OXC: Oro‐Facial Cleft/Craniofacial Malformations	9		Risk Ratio (M‐H, Fixed, 95% CI)	Subtotals only
63.4.1 VPA vs OXC (cohort studies)	9	1178	Risk Ratio (M‐H, Fixed, 95% CI)	2.14 [0.76, 6.06]
63.5 VPA vs OXC: Skeletal/Limb Malformations	9		Risk Ratio (M‐H, Fixed, 95% CI)	Subtotals only
63.5.1 VPA vs OXC (cohort studies)	9	1497	Risk Ratio (M‐H, Fixed, 95% CI)	1.37 [0.42, 4.49]

**Comparison 64:** VPA vs PB

Outcome or subgroup title	No. of studies	No. of participants	Statistical method	Effect size
64.1 VPA vs PB: All Major Malformations	25		Risk Ratio (M‐H, Fixed, 95% CI)	Subtotals only
64.1.1 VPA vs PB (cohort studies)	23	2316	Risk Ratio (M‐H, Fixed, 95% CI)	1.49 [1.08, 2.07]
64.1.2 VPA vs PB (database studies)	2	635	Risk Ratio (M‐H, Fixed, 95% CI)	0.79 [0.26, 2.42]
64.2 VPA vs PB: Neural Tube Malformations	14		Risk Ratio (M‐H, Fixed, 95% CI)	Subtotals only
64.2.1 VPA vs PB (cohort studies)	14	1720	Risk Ratio (M‐H, Fixed, 95% CI)	3.04 [1.27, 7.30]
64.3 VPA vs PB: Cardiac Malformations	14		Risk Ratio (M‐H, Fixed, 95% CI)	Subtotals only
64.3.1 VPA vs PB (cohort studies)	14	1720	Risk Ratio (M‐H, Fixed, 95% CI)	0.84 [0.50, 1.43]
64.4 VPA vs PB: Oro‐Facial Cleft/Craniofacial Malformations	14		Risk Ratio (M‐H, Fixed, 95% CI)	Subtotals only
64.4.1 VPA vs PB (cohort studies)	14	1257	Risk Ratio (M‐H, Fixed, 95% CI)	0.54 [0.23, 1.27]
64.5 VPA vs PB: Skeletal/Limb Malformations	14		Risk Ratio (M‐H, Fixed, 95% CI)	Subtotals only
64.5.1 VPA vs PB (cohort studies)	14	1720	Risk Ratio (M‐H, Fixed, 95% CI)	1.62 [0.70, 3.74]

**Comparison 65:** VPA vs PHT

Outcome or subgroup title	No. of studies	No. of participants	Statistical method	Effect size
65.1 VPA vs PHT: All Major Malformations	22		Risk Ratio (M‐H, Fixed, 95% CI)	Subtotals only
65.1.1 VPA vs PHT (cohort studies)	21	3897	Risk Ratio (M‐H, Fixed, 95% CI)	1.92 [1.44, 2.56]
65.1.2 VPA vs PHT (database studies)	1	371	Risk Ratio (M‐H, Fixed, 95% CI)	1.43 [0.64, 3.19]
65.2 VPA vs PHT: Neural Tube Malformations	14		Risk Ratio (M‐H, Fixed, 95% CI)	Subtotals only
65.2.1 VPA vs PHT (cohort studies)	14	3393	Risk Ratio (M‐H, Fixed, 95% CI)	3.75 [1.57, 8.94]
65.3 VPA vs PHT: Cardiac Malformations	14		Risk Ratio (M‐H, Fixed, 95% CI)	Subtotals only
65.3.1 VPA vs PHT (cohort studies)	14	3393	Risk Ratio (M‐H, Fixed, 95% CI)	1.90 [1.07, 3.36]
65.4 VPA vs PHT: Oro‐Facial Cleft/Craniofacial Malformations	14		Risk Ratio (M‐H, Fixed, 95% CI)	Subtotals only
65.4.1 VPA vs PHT (cohort studies)	14	2944	Risk Ratio (M‐H, Fixed, 95% CI)	2.24 [0.89, 5.58]
65.5 VPA vs PHT: Skeletal/Limb Malformations	14		Risk Ratio (M‐H, Fixed, 95% CI)	Subtotals only
65.5.1 VPA vs PHT (cohort studies)	14	3394	Risk Ratio (M‐H, Fixed, 95% CI)	2.12 [1.01, 4.45]

**Comparison 66:** VPA vs ZNS

Outcome or subgroup title	No. of studies	No. of participants	Statistical method	Effect size
66.1 VPA vs ZNS: All Major Malformations	3		Risk Ratio (M‐H, Fixed, 95% CI)	Subtotals only
66.1.1 VPA vs ZNS (cohort studies)	3	1677	Risk Ratio (M‐H, Fixed, 95% CI)	2.34 [0.95, 5.80]
66.2 VPA vs ZNS: Neural Tube Malformations	2		Risk Ratio (M‐H, Fixed, 95% CI)	Subtotals only
66.2.1 VPA vs ZNS (cohort studies)	2	1264	Risk Ratio (M‐H, Fixed, 95% CI)	0.29 [0.06, 1.51]
66.3 VPA vs ZNS: Cardiac Malformations	2		Risk Ratio (M‐H, Fixed, 95% CI)	Subtotals only
66.3.1 VPA vs ZNS (cohort studies)	2	1264	Risk Ratio (M‐H, Fixed, 95% CI)	0.49 [0.07, 3.65]
66.4 VPA vs ZNS: Oro‐Facial Cleft/Craniofacial Malformations	2		Risk Ratio (M‐H, Fixed, 95% CI)	Subtotals only
66.4.1 VPA vs ZNS (cohort studies)	2	1264	Risk Ratio (M‐H, Fixed, 95% CI)	0.47 [0.06, 3.49]
66.5 VPA vs ZNS: Skeletal/Limb Malformations	2		Risk Ratio (M‐H, Fixed, 95% CI)	Subtotals only
66.5.1 VPA vs ZNS (cohort studies)	2	1264	Risk Ratio (M‐H, Fixed, 95% CI)	0.46 [0.03, 7.72]

**Comparison 67:** CZP vs VPA

Outcome or subgroup title	No. of studies	No. of participants	Statistical method	Effect size
67.1 CZP vs VPA: All Major Malformations	6		Risk Ratio (M‐H, Fixed, 95% CI)	Subtotals only
67.1.1 CZP vs VPA (cohort studies)	4	1050	Risk Ratio (M‐H, Fixed, 95% CI)	0.29 [0.09, 0.90]
67.1.2 CZP vs VPA (database studies)	2	762	Risk Ratio (M‐H, Fixed, 95% CI)	0.34 [0.13, 0.94]
67.2 CZP vs VPA: Neural Tube Malformations	1		Risk Ratio (M‐H, Fixed, 95% CI)	Subtotals only
67.2.1 CZP vs VPA (cohort studies)	1	345	Risk Ratio (M‐H, Fixed, 95% CI)	9.77 [0.58, 165.35]
67.3 CZP vs VPA: Cardiac Malformations	1		Risk Ratio (M‐H, Fixed, 95% CI)	Subtotals only
67.3.1 CZP vs VPA (cohort studies)	1	345	Risk Ratio (M‐H, Fixed, 95% CI)	1.67 [0.12, 23.92]
67.4 CZP vs VPA: Skeletal/Limb Malformations	1		Risk Ratio (M‐H, Fixed, 95% CI)	Subtotals only
67.4.1 CZP vs VPA (cohort studies)	1	345	Risk Ratio (M‐H, Fixed, 95% CI)	7.60 [0.47, 123.14]

**Comparison 68:** CZP vs LEV

Outcome or subgroup title	No. of studies	No. of participants	Statistical method	Effect size
68.1 CZP vs LEV: All Major Malformations	4		Risk Ratio (M‐H, Fixed, 95% CI)	Subtotals only
68.1.1 CZP vs LEV (cohort studies)	3	789	Risk Ratio (M‐H, Fixed, 95% CI)	1.06 [0.32, 3.44]
68.1.2 CZP vs LEV (database studies)	1	231	Risk Ratio (M‐H, Fixed, 95% CI)	1.04 [0.15, 7.29]

**Comparison 69:** OXC vs PRM

Outcome or subgroup title	No. of studies	No. of participants	Statistical method	Effect size
69.1 OXC vs PRM: All Major Malformations	3		Risk Ratio (M‐H, Fixed, 95% CI)	Subtotals only
69.1.1 OXC vs PRM (cohort studies)	2	36	Risk Ratio (M‐H, Fixed, 95% CI)	0.58 [0.08, 4.03]
69.1.2 OXC vs PRM (database studies)	1	0	Risk Ratio (M‐H, Fixed, 95% CI)	Not estimable

**Comparison 70:** OXC vs TPM

Outcome or subgroup title	No. of studies	No. of participants	Statistical method	Effect size
70.1 OXC vs TPM: All Major Malformations	7		Risk Ratio (M‐H, Fixed, 95% CI)	Subtotals only
70.1.1 OXC vs TPM (cohort studies)	5	706	Risk Ratio (M‐H, Fixed, 95% CI)	0.71 [0.28, 1.77]
70.1.2 OXC vs TPM (database studies)	2	110	Risk Ratio (M‐H, Fixed, 95% CI)	0.42 [0.04, 4.50]
70.2 OXC vs TPM: Neural Tube Malformations	4		Risk Ratio (M‐H, Fixed, 95% CI)	Subtotals only
70.2.1 OXC vs TPM (cohort studies)	4	0	Risk Ratio (M‐H, Fixed, 95% CI)	Not estimable
70.3 OXC vs PRM: Cardiac Malformations	5		Risk Ratio (M‐H, Fixed, 95% CI)	Subtotals only
70.3.1 OXC vs TPM (cohort studies)	5	688	Risk Ratio (M‐H, Fixed, 95% CI)	0.80 [0.09, 6.81]
70.4 OXC vs PRM: Oro‐Facial Cleft/Craniofacial Malformations	4		Risk Ratio (M‐H, Fixed, 95% CI)	Subtotals only
70.4.1 OXC vs TPM (cohort studies)	4	608	Risk Ratio (M‐H, Fixed, 95% CI)	0.39 [0.05, 3.35]
70.5 OXC vs PRM: Skeletal/Limb Malformations	4		Risk Ratio (M‐H, Fixed, 95% CI)	Subtotals only
70.5.1 OXC vs TPM (cohort studies)	4	684	Risk Ratio (M‐H, Fixed, 95% CI)	0.40 [0.07, 2.44]

**Comparison 71:** OXC vs ZNS

Outcome or subgroup title	No. of studies	No. of participants	Statistical method	Effect size
71.1 OXC vs ZNS: All Major Malformations	2		Risk Ratio (M‐H, Fixed, 95% CI)	Subtotals only
71.1.1 OXC vs ZNS (cohort studies)	2	277	Risk Ratio (M‐H, Fixed, 95% CI)	4.48 [0.24, 82.23]
71.2 OXC vs ZNS: Neural Tube Malformations	1		Risk Ratio (M‐H, Fixed, 95% CI)	Subtotals only
71.2.1 OXC vs ZNS (cohort studies)	1	0	Risk Ratio (M‐H, Fixed, 95% CI)	Not estimable
71.3 OXC vs ZNS: Cardiac Malformations	1		Risk Ratio (M‐H, Fixed, 95% CI)	Subtotals only
71.3.1 OXC vs ZNS (cohort studies)	1	0	Risk Ratio (M‐H, Fixed, 95% CI)	Not estimable
71.4 OXC vs ZNS: Oro‐Facial Cleft/Craniofacial Malformations	1		Risk Ratio (M‐H, Fixed, 95% CI)	Subtotals only
71.4.1 OXC vs ZNS (cohort studies)	1	0	Risk Ratio (M‐H, Fixed, 95% CI)	Not estimable
71.5 OXC vs ZNS: Skeletal/Limb Malformations	1		Risk Ratio (M‐H, Fixed, 95% CI)	Subtotals only
71.5.1 OXC vs ZNS (cohort studies)	1	0	Risk Ratio (M‐H, Fixed, 95% CI)	Not estimable

**Comparison 72:** PRM vs TPM

Outcome or subgroup title	No. of studies	No. of participants	Statistical method	Effect size
72.1 PRM vs TPM: All Major Malformations	2		Risk Ratio (M‐H, Fixed, 95% CI)	Subtotals only
72.1.1 PRM vs TPM (cohort studies)	1	55	Risk Ratio (M‐H, Fixed, 95% CI)	6.00 [0.30, 118.36]
72.1.2 PRM vs TPM (database studies)	1	0	Risk Ratio (M‐H, Fixed, 95% CI)	Not estimable

**Comparison 73:** PRM vs VPA

Outcome or subgroup title	No. of studies	No. of participants	Statistical method	Effect size
73.1 PRM vs VPA: All Major Malformations	7		Risk Ratio (M‐H, Fixed, 95% CI)	Subtotals only
73.1.1 PRM vs VPA (cohort studies)	6	594	Risk Ratio (M‐H, Fixed, 95% CI)	0.74 [0.39, 1.40]
73.1.2 PRM vs VPA (database studies)	1	271	Risk Ratio (M‐H, Fixed, 95% CI)	1.27 [0.09, 17.39]
73.2 PRM vs VPA: Neural Tube Malformations	2		Risk Ratio (M‐H, Fixed, 95% CI)	Subtotals only
73.2.1 PRM vs VPA (cohort studies)	2	84	Risk Ratio (M‐H, Fixed, 95% CI)	0.11 [0.01, 1.99]
73.3 PRM vs VPA: Cardiac Malformations	2		Risk Ratio (M‐H, Fixed, 95% CI)	Subtotals only
73.3.1 PRM vs VPA (cohort studies)	2	84	Risk Ratio (M‐H, Fixed, 95% CI)	3.75 [0.16, 89.32]
73.4 PRM vs VPA: Oro‐Facial Cleft/Craniofacial Malformations	2		Risk Ratio (M‐H, Fixed, 95% CI)	Subtotals only
73.4.1 PRM vs VPA (cohort studies)	2	0	Risk Ratio (M‐H, Fixed, 95% CI)	Not estimable
73.5 PRM vs VPA: Skeletal/Limb Malformations	2		Risk Ratio (M‐H, Fixed, 95% CI)	Subtotals only
73.5.1 PRM vs VPA (cohort studies)	2	84	Risk Ratio (M‐H, Fixed, 95% CI)	0.42 [0.02, 9.92]

**Comparison 74:** LEV vs LAC

Outcome or subgroup title	No. of studies	No. of participants	Statistical method	Effect size
74.1 LEV vs LAC: All Major Malformations	1		Risk Ratio (M‐H, Fixed, 95% CI)	Subtotals only
74.1.1 LEV vs LAC (cohort studies)	1	0	Risk Ratio (M‐H, Fixed, 95% CI)	Not estimable

**Comparison 75:** CBZ vs LAC

Outcome or subgroup title	No. of studies	No. of participants	Statistical method	Effect size
75.1 CBZ vs LAC: All Major Malformations	1		Risk Ratio (M‐H, Fixed, 95% CI)	Subtotals only
75.1.1 CBZ vs LAC (cohort studies)	1	0	Risk Ratio (M‐H, Fixed, 95% CI)	Not estimable

**Comparison 76:** OXC vs LAC

Outcome or subgroup title	No. of studies	No. of participants	Statistical method	Effect size
76.1 OXC vs LAC: All Major Malformations	1		Risk Ratio (M‐H, Fixed, 95% CI)	Subtotals only
76.1.1 OXC vs LAC (cohort studies)	1	0	Risk Ratio (M‐H, Fixed, 95% CI)	Not estimable

**Comparison 77:** PB vs LAC

Outcome or subgroup title	No. of studies	No. of participants	Statistical method	Effect size
77.1 PB vs LAC: All Major Malformations	1		Risk Ratio (M‐H, Fixed, 95% CI)	Subtotals only
77.1.1 PB vs LAC (cohort studies)	1	0	Risk Ratio (M‐H, Fixed, 95% CI)	Not estimable

**Comparison 78:** LAC vs ZNS

Outcome or subgroup title	No. of studies	No. of participants	Statistical method	Effect size
78.1 LAC vs ZNS: All Major Malformations	1		Risk Ratio (M‐H, Fixed, 95% CI)	Subtotals only
78.1.1 LAC vs ZNS (cohort studies)	1	0	Risk Ratio (M‐H, Fixed, 95% CI)	Not estimable

**Comparison 79:** GBP vs PGB

Outcome or subgroup title	No. of studies	No. of participants	Statistical method	Effect size
79.1 GBP vs PGB: All Major Malformations	1		Risk Ratio (M‐H, Fixed, 95% CI)	Subtotals only
79.1.1 GBP vs PGB (cohort studies)	1	0	Risk Ratio (M‐H, Fixed, 95% CI)	Not estimable

**Comparison 80:** GBP vs CZP

Outcome or subgroup title	No. of studies	No. of participants	Statistical method	Effect size
80.1 GBP vs CZP: All Major Malformations	1		Risk Ratio (M‐H, Fixed, 95% CI)	Subtotals only
80.1.1 GBP vs CZP (database studies)	1	66	Risk Ratio (M‐H, Fixed, 95% CI)	0.52 [0.03, 10.25]

**Comparison 81:** VPA vs BNZ

Outcome or subgroup title	No. of studies	No. of participants	Statistical method	Effect size
81.1 VPA vs BNZ: All Major Malformations	2		Risk Ratio (M‐H, Fixed, 95% CI)	Subtotals only
81.1.1 VPA vs BNZ (cohort studies)	2	49	Risk Ratio (M‐H, Fixed, 95% CI)	1.50 [0.11, 21.31]

**Comparison 82:** LTG vs BNZ

Outcome or subgroup title	No. of studies	No. of participants	Statistical method	Effect size
82.1 LTG vs BNZ: All Major Malformations	2		Risk Ratio (M‐H, Fixed, 95% CI)	Subtotals only
82.1.1 LTG vs BNZ (cohort studies)	2	0	Risk Ratio (M‐H, Fixed, 95% CI)	Not estimable

**Comparison 83:** LEV vs BNZ

Outcome or subgroup title	No. of studies	No. of participants	Statistical method	Effect size
83.1 LEV vs BNZ: All Major Malformations	2		Risk Ratio (M‐H, Fixed, 95% CI)	Subtotals only
83.1.1 LEV vs BNZ (cohort studies)	2	0	Risk Ratio (M‐H, Fixed, 95% CI)	Not estimable

**Comparison 84:** CBZ vs BNZ

Outcome or subgroup title	No. of studies	No. of participants	Statistical method	Effect size
84.1 CBZ vs BNZ: All Major Malformations	2		Risk Ratio (M‐H, Fixed, 95% CI)	Subtotals only
84.1.1 CBZ vs BNZ (cohort studies)	2	48	Risk Ratio (M‐H, Fixed, 95% CI)	0.32 [0.02, 6.71]

**Comparison 85:** OXC vs BNZ

Outcome or subgroup title	No. of studies	No. of participants	Statistical method	Effect size
85.1 OXC vs BNZ: All Major Malformations	1		Risk Ratio (M‐H, Fixed, 95% CI)	Subtotals only
85.1.1 OXC vs BNZ (cohort studies)	1	0	Risk Ratio (M‐H, Fixed, 95% CI)	Not estimable

**Comparison 86:** PB vs BNZ

Outcome or subgroup title	No. of studies	No. of participants	Statistical method	Effect size
86.1 PB vs BNZ: All Major Malformations	1		Risk Ratio (M‐H, Fixed, 95% CI)	Subtotals only
86.1.1 PB vs BNZ (cohort studies)	1	0	Risk Ratio (M‐H, Fixed, 95% CI)	Not estimable

**Comparison 87:** LAC vs BNZ

Outcome or subgroup title	No. of studies	No. of participants	Statistical method	Effect size
87.1 LAC vs BNZ: All Major Malformations	1		Risk Ratio (M‐H, Fixed, 95% CI)	Subtotals only
87.1.1 LAC vs BNZ (cohort studies)	1	0	Risk Ratio (M‐H, Fixed, 95% CI)	Not estimable

**Comparison 88:** ZNS vs BNZ

Outcome or subgroup title	No. of studies	No. of participants	Statistical method	Effect size
88.1 ZNS vs BNZ: All Major Malformations	1		Risk Ratio (M‐H, Fixed, 95% CI)	Subtotals only
88.1.1 ZNS vs BNZ (cohort studies)	1	0	Risk Ratio (M‐H, Fixed, 95% CI)	Not estimable

**Comparison 89:** CZP vs TPM

Outcome or subgroup title	No. of studies	No. of participants	Statistical method	Effect size
89.1 CZP vs TPM: All Major Malformations	3		Risk Ratio (M‐H, Fixed, 95% CI)	Subtotals only
89.1.1 CZP vs TPM (cohort studies)	1	79	Risk Ratio (M‐H, Fixed, 95% CI)	0.67 [0.03, 15.83]
89.1.2 CZP vs TPM (database studies)	2	210	Risk Ratio (M‐H, Fixed, 95% CI)	0.37 [0.07, 1.87]

**Comparison 90:** CZP vs OXC

Outcome or subgroup title	No. of studies	No. of participants	Statistical method	Effect size
90.1 CZP vs OXC: All Major Malformations	3		Risk Ratio (M‐H, Fixed, 95% CI)	Subtotals only
90.1.1 CZP vs OXC (cohort studies)	1	45	Risk Ratio (M‐H, Fixed, 95% CI)	0.25 [0.01, 5.75]
90.1.2 CZP vs OXC (database studies)	2	222	Risk Ratio (M‐H, Fixed, 95% CI)	0.81 [0.13, 5.06]

**Comparison 91:** CZP vs COZ

Outcome or subgroup title	No. of studies	No. of participants	Statistical method	Effect size
91.1 CZP vs COZ: All Major Malformations	1		Risk Ratio (M‐H, Fixed, 95% CI)	Subtotals only
91.1.1 CZP vs COZ (cohort studies)	1	0	Risk Ratio (M‐H, Fixed, 95% CI)	Not estimable

**Comparison 92:** CZP vs ESM

Outcome or subgroup title	No. of studies	No. of participants	Statistical method	Effect size
92.1 CZP vs ESM: All Major Malformations	2		Risk Ratio (M‐H, Fixed, 95% CI)	Subtotals only
92.1.1 CZP vs ESM (cohort studies)	1	0	Risk Ratio (M‐H, Fixed, 95% CI)	Not estimable
92.1.2 CZP vs ESM (database studies)	1	56	Risk Ratio (M‐H, Fixed, 95% CI)	0.92 [0.05, 17.58]

**Comparison 93:** CZP vs PRG

Outcome or subgroup title	No. of studies	No. of participants	Statistical method	Effect size
93.1 CZP vs PRG: All Major Malformations	1		Risk Ratio (M‐H, Fixed, 95% CI)	Subtotals only
93.1.1 CZP vs PRG (cohort studies)	1	0	Risk Ratio (M‐H, Fixed, 95% CI)	Not estimable

**Comparison 94:** CZP vs PRM

Outcome or subgroup title	No. of studies	No. of participants	Statistical method	Effect size
94.1 CZP vs PRM: All Major Malformations	2		Risk Ratio (M‐H, Fixed, 95% CI)	Subtotals only
94.1.1 CZP vs PRM (cohort studies)	1	0	Risk Ratio (M‐H, Fixed, 95% CI)	Not estimable
94.1.2 CZP vs PRM (database studies)	1	51	Risk Ratio (M‐H, Fixed, 95% CI)	0.41 [0.02, 7.13]

**Comparison 95:** CZP vs VGB

Outcome or subgroup title	No. of studies	No. of participants	Statistical method	Effect size
95.1 CZP vs VGB: All Major Malformations	2		Risk Ratio (M‐H, Fixed, 95% CI)	Subtotals only
95.1.1 CZP vs VGB (cohort studies)	1	0	Risk Ratio (M‐H, Fixed, 95% CI)	Not estimable
95.1.2 CZP vs VGB (database studies)	1	51	Risk Ratio (M‐H, Fixed, 95% CI)	0.41 [0.02, 7.13]

**Comparison 96:** TPM vs BNZ

Outcome or subgroup title	No. of studies	No. of participants	Statistical method	Effect size
96.1 TPM vs BNZ: All Major Malformations	2		Risk Ratio (M‐H, Fixed, 95% CI)	Subtotals only
96.1.1 TPM vs BNZ (cohort studies)	2	0	Risk Ratio (M‐H, Fixed, 95% CI)	Not estimable

**Comparison 97:** ESM vs VPA

Outcome or subgroup title	No. of studies	No. of participants	Statistical method	Effect size
97.1 ESM vs VPA: All Major Malformations	2		Risk Ratio (M‐H, Fixed, 95% CI)	Subtotals only
97.1.1 ESM vs VPA (cohort studies)	1	295	Risk Ratio (M‐H, Fixed, 95% CI)	0.56 [0.04, 8.03]
97.1.2 ESM vs VPA (database studies)	1	276	Risk Ratio (M‐H, Fixed, 95% CI)	0.56 [0.04, 8.54]

**Comparison 98:** ESM vs CBZ

Outcome or subgroup title	No. of studies	No. of participants	Statistical method	Effect size
98.1 ESM vs CBZ: All Major Malformations	2		Risk Ratio (M‐H, Fixed, 95% CI)	Subtotals only
98.1.1 ESM vs CBZ (cohort studies)	1	414	Risk Ratio (M‐H, Fixed, 95% CI)	1.39 [0.10, 20.37]
98.1.2 ESM vs CBZ (database studies)	1	711	Risk Ratio (M‐H, Fixed, 95% CI)	1.37 [0.09, 20.78]

**Comparison 99:** ESM vs PRM

Outcome or subgroup title	No. of studies	No. of participants	Statistical method	Effect size
99.1 ESM vs PRM: All Major Malformations	2		Risk Ratio (M‐H, Fixed, 95% CI)	Subtotals only
99.1.1 ESM vs PRM (cohort studies)	1	0	Risk Ratio (M‐H, Fixed, 95% CI)	Not estimable
99.1.2 ESM vs PRM (database studies)	1	0	Risk Ratio (M‐H, Fixed, 95% CI)	Not estimable

**Comparison 100:** ESM vs PB

Outcome or subgroup title	No. of studies	No. of participants	Statistical method	Effect size
100.1 ESM vs PB: All Major Malformations	2		Risk Ratio (M‐H, Fixed, 95% CI)	Subtotals only
100.1.1 ESM vs PB (cohort studies)	1	0	Risk Ratio (M‐H, Fixed, 95% CI)	Not estimable
100.1.2 ESM vs PB (database studies)	1	15	Risk Ratio (M‐H, Fixed, 95% CI)	0.30 [0.01, 6.29]

**Comparison 101:** ESM vs PHT

Outcome or subgroup title	No. of studies	No. of participants	Statistical method	Effect size
101.1 ESM vs PHT: All Major Malformations	2		Risk Ratio (M‐H, Fixed, 95% CI)	Subtotals only
101.1.1 ESM vs PHT (cohort studies)	1	49	Risk Ratio (M‐H, Fixed, 95% CI)	2.50 [0.11, 54.68]
101.1.2 ESM vs PHT (database studies)	1	111	Risk Ratio (M‐H, Fixed, 95% CI)	0.77 [0.05, 12.42]

**Comparison 102:** ESM vs OXC

Outcome or subgroup title	No. of studies	No. of participants	Statistical method	Effect size
102.1 ESM vs OXC: All Major Malformations	2		Risk Ratio (M‐H, Fixed, 95% CI)	Subtotals only
102.1.1 ESM vs OXC (cohort studies)	1	24	Risk Ratio (M‐H, Fixed, 95% CI)	1.11 [0.05, 23.88]
102.1.2 ESM vs OXC (database studies)	1	0	Risk Ratio (M‐H, Fixed, 95% CI)	Not estimable

**Comparison 103:** ESM vs VGB

Outcome or subgroup title	No. of studies	No. of participants	Statistical method	Effect size
103.1 ESM vs VGB: All Major Malformations	2		Risk Ratio (M‐H, Fixed, 95% CI)	Subtotals only
103.1.1 ESM vs VGB (cohort studies)	1	0	Risk Ratio (M‐H, Fixed, 95% CI)	Not estimable
103.1.2 ESM vs VGB (database studies)	1	0	Risk Ratio (M‐H, Fixed, 95% CI)	Not estimable

**Comparison 104:** ESM vs LTG

Outcome or subgroup title	No. of studies	No. of participants	Statistical method	Effect size
104.1 ESM vs LTG: All Major Malformations	2		Risk Ratio (M‐H, Fixed, 95% CI)	Subtotals only
104.1.1 ESM vs LTG (cohort studies)	1	411	Risk Ratio (M‐H, Fixed, 95% CI)	1.65 [0.11, 24.30]
104.1.2 ESM vs LTG (database studies)	1	98	Risk Ratio (M‐H, Fixed, 95% CI)	1.12 [0.07, 19.24]

**Comparison 105:** ESM vs TPM

Outcome or subgroup title	No. of studies	No. of participants	Statistical method	Effect size
105.1 ESM vs TPM: All Major Malformations	2		Risk Ratio (M‐H, Fixed, 95% CI)	Subtotals only
105.1.1 ESM vs TPM (cohort studies)	1	58	Risk Ratio (M‐H, Fixed, 95% CI)	3.00 [0.14, 65.77]
105.1.2 ESM vs TPM (database studies)	1	0	Risk Ratio (M‐H, Fixed, 95% CI)	Not estimable

**Comparison 106:** ESM vs GBP

Outcome or subgroup title	No. of studies	No. of participants	Statistical method	Effect size
106.1 ESM vs GBP: All Major Malformations	1		Risk Ratio (M‐H, Fixed, 95% CI)	Subtotals only
106.1.1 ESM vs GBP (database studies)	1	0	Risk Ratio (M‐H, Fixed, 95% CI)	Not estimable

**Comparison 107:** VGB vs VPA

Outcome or subgroup title	No. of studies	No. of participants	Statistical method	Effect size
107.1 VGB vs VPA: All Major Malformations	2		Risk Ratio (M‐H, Fixed, 95% CI)	Subtotals only
107.1.1 VGB vs VPA (cohort studies)	1	291	Risk Ratio (M‐H, Fixed, 95% CI)	1.67 [0.15, 18.73]
107.1.2 VGB vs VPA (database studies)	1	271	Risk Ratio (M‐H, Fixed, 95% CI)	1.27 [0.09, 17.39]

**Comparison 108:** VGB vs CBZ

Outcome or subgroup title	No. of studies	No. of participants	Statistical method	Effect size
108.1 VGB vs CBZ: All Major Malformations	2		Risk Ratio (M‐H, Fixed, 95% CI)	Subtotals only
108.1.1 VGB vs CBZ (cohort studies)	1	410	Risk Ratio (M‐H, Fixed, 95% CI)	4.18 [0.37, 47.57]
108.1.2 VGB vs CBZ (database studies)	1	706	Risk Ratio (M‐H, Fixed, 95% CI)	3.09 [0.23, 42.31]

**Comparison 109:** VGB vs PRM

Outcome or subgroup title	No. of studies	No. of participants	Statistical method	Effect size
109.1 VGB vs PRM: All Major Malformations	2		Risk Ratio (M‐H, Fixed, 95% CI)	Subtotals only
109.1.1 VGB vs PRM (cohort studies)	1	0	Risk Ratio (M‐H, Fixed, 95% CI)	Not estimable
109.1.2 VGB vs PRM (database studies)	1	0	Risk Ratio (M‐H, Fixed, 95% CI)	Not estimable

**Comparison 110:** VGB vs PB

Outcome or subgroup title	No. of studies	No. of participants	Statistical method	Effect size
110.1 VGB vs PB: All Major Malformations	1		Risk Ratio (M‐H, Fixed, 95% CI)	Subtotals only
110.1.1 VGB vs PB (database studies)	1	10	Risk Ratio (M‐H, Fixed, 95% CI)	0.67 [0.03, 12.96]

**Comparison 111:** VGB vs PHT

Outcome or subgroup title	No. of studies	No. of participants	Statistical method	Effect size
111.1 VGB vs PHT: All Major Malformations	2		Risk Ratio (M‐H, Fixed, 95% CI)	Subtotals only
111.1.1 VGB vs PHT (cohort studies)	1	45	Risk Ratio (M‐H, Fixed, 95% CI)	7.50 [0.43, 132.30]
111.1.2 VGB vs PHT (database studies)	1	106	Risk Ratio (M‐H, Fixed, 95% CI)	1.73 [0.12, 25.35]

**Comparison 112:** VGB vs OXC

Outcome or subgroup title	No. of studies	No. of participants	Statistical method	Effect size
112.1 VGB vs OXC: All Major Malformations	2		Risk Ratio (M‐H, Fixed, 95% CI)	Subtotals only
112.1.1 VGB vs OXC (cohort studies)	1	20	Risk Ratio (M‐H, Fixed, 95% CI)	3.33 [0.19, 57.71]
112.1.2 VGB vs OXC (database studies)	1	0	Risk Ratio (M‐H, Fixed, 95% CI)	Not estimable

**Comparison 113:** VGB vs LTG

Outcome or subgroup title	No. of studies	No. of participants	Statistical method	Effect size
113.1 VGB vs LTG: All Major Malformations	2		Risk Ratio (M‐H, Fixed, 95% CI)	Subtotals only
113.1.1 VGB vs LTG (cohort studies)	1	408	Risk Ratio (M‐H, Fixed, 95% CI)	3.31 [0.25, 43.03]
113.1.2 VGB vs LTG (database studies)	1	93	Risk Ratio (M‐H, Fixed, 95% CI)	2.53 [0.16, 39.34]

**Comparison 114:** VGB vs TPM

Outcome or subgroup title	No. of studies	No. of participants	Statistical method	Effect size
114.1 VGB vs TPM: All Major Malformations	2		Risk Ratio (M‐H, Fixed, 95% CI)	Subtotals only
114.1.1 VGB vs TPM (cohort studies)	1	54	Risk Ratio (M‐H, Fixed, 95% CI)	9.00 [0.51, 159.15]
114.1.2 VGB vs TPM (database studies)	1	0	Risk Ratio (M‐H, Fixed, 95% CI)	Not estimable

**Comparison 115:** VGB vs GBP

Outcome or subgroup title	No. of studies	No. of participants	Statistical method	Effect size
115.1 VGB vs GBP: All Major Malformations	1		Risk Ratio (M‐H, Fixed, 95% CI)	Subtotals only
115.1.1 VGB vs GBP (database studies)	1	0	Risk Ratio (M‐H, Fixed, 95% CI)	Not estimable

**Comparison 116:** CZP vs PB

Outcome or subgroup title	No. of studies	No. of participants	Statistical method	Effect size
116.1 CZP vs PB: All Major Malformations	4		Risk Ratio (M‐H, Fixed, 95% CI)	Subtotals only
116.1.1 CZP vs PB (cohort studies)	2	33	Risk Ratio (M‐H, Fixed, 95% CI)	0.83 [0.05, 13.02]
116.1.2 CZP vs PB (database studies)	2	195	Risk Ratio (M‐H, Fixed, 95% CI)	0.26 [0.06, 1.12]

**Comparison 117:** CZP vs PHT

Outcome or subgroup title	No. of studies	No. of participants	Statistical method	Effect size
117.1 CZP vs PHT: All Major Malformations	3		Risk Ratio (M‐H, Fixed, 95% CI)	Subtotals only
117.1.1 CZP vs PHT (cohort studies)	2	93	Risk Ratio (M‐H, Fixed, 95% CI)	0.71 [0.10, 5.11]
117.1.2 CZP vs PHT (database studies)	1	151	Risk Ratio (M‐H, Fixed, 95% CI)	0.61 [0.13, 2.84]

**Comparison 118:** ESM vs LEV

Outcome or subgroup title	No. of studies	No. of participants	Statistical method	Effect size
118.1 ESM vs LEV: All Major Malformations	1		Risk Ratio (M‐H, Fixed, 95% CI)	Subtotals only
118.1.1 ESM vs LEV (cohort studies)	1	144	Risk Ratio (M‐H, Fixed, 95% CI)	2.12 [0.13, 34.10]

**Comparison 119:** ESM vs Controls

Outcome or subgroup title	No. of studies	No. of participants	Statistical method	Effect size
119.1 ESM vs Controls: All Major Malformations	1		Risk Ratio (M‐H, Fixed, 95% CI)	Subtotals only
119.1.1 ESM vs WWE ‐ No Medication (cohort studies)	1	181	Risk Ratio (M‐H, Fixed, 95% CI)	2.68 [0.17, 43.16]

**Comparison 120:** VGB vs Controls

Outcome or subgroup title	No. of studies	No. of participants	Statistical method	Effect size
120.1 VGB vs Controls: All Major Malformations	1		Risk Ratio (M‐H, Fixed, 95% CI)	Subtotals only
120.1.1 VGB vs WWE ‐ No Medication (cohort studies)	1	177	Risk Ratio (M‐H, Fixed, 95% CI)	8.05 [0.64, 101.76]

## Characteristics of studies

### Characteristics of included studies [ordered by study ID]

**Table:** Al Bunyan 1999

***Study characteristics***		
Methods	Prospective cohort study	
Participants	Intervention group: Women on AED monotherapy or polytherapy Control group: Women with epilepsy not taking AEDs	
Interventions	Intervention groups (monotherapy): 1) CBZ (N = 31) 2) PHT (N = 9) 3) VPA (N = 5) 4) PB (N = 2) 5) CZP (N = 1) Control group: 1) Women with epilepsy not taking any AEDs (N = 10)	
Outcomes	Congenital malformations	
Funding	Not reported	
Country	Saudi Arabia	
Notes

**Table:** AlSheikh 2020

***Study characteristics***		
Methods	Prospective cohort study	
Participants	Intervention group: women on AED monotherapy or polytherapy Control group: women with epilepsy not taking AEDs	
Interventions	Intervention groups (monotherapy): 1) LTG (N = 20) 2) LEV (N = 9) 3) CBZ (N = 5) 4) OXC (N = 3) 5) TPM (N = 1) 6) VPA (N = 1) 7) Polytherapy (N = 21) Control group: 1) Women with epilepsy not taking AED (N = 8)	
Outcomes	Major congenital malformations	
Funding	None reported	
Country	Saudi Arabia	
Notes

**Table:** Australian Epilepsy and Pregnancy Register

***Study characteristics***		
Methods	Prospective cohort study	
Participants	Intervention group: Women with epilepsy treated with AED Control group: Women with epilepsy not taking AEDs	
Interventions	Intervention groups (monotherapy): 1) CBZ (N = 409) 2) VPA (N = 290) 3) LTG (N = 406) 4) TPM (N = 53) 5) PHT (N = 44) 6) LEV (N = 139) 7) OXC (N = 19) 8) PB (N = 2) 9) CZP (N = 26) 10) CLB (N =2) 11) ETX (N = 5) 12) PRG (N =1) 13) PRM (N =2) 14) TGB (N = 1) 15) VGB (N =1) 16) GBP (N = 14) Control group: 1) Women with epilepsy not taking AEDs (N = 176)	
Outcomes	Incidence of malformations	
Funding	Pharma companies, Epilepsy Society of Australia and Epilepsy Action	
Country	Australia	
Notes	Protocol received. Personal communication received regarding number of specific malformations by monotherapy

**Table:** Bag 1989

***Study characteristics***		
Methods	Prospective cohort study	
Participants	Intervention group: Women on AED monotherapy or polytherapy	
Interventions	Intervention groups (monotherapy): 1) PHT (N = 20) 2) CBZ (N = 4)	
Outcomes	Congenital malformations	
Funding	Not reported	
Country	India	
Notes	There were 2 spontaneous abortions. Study authors' contact details could not be found.

**Table:** Barqawi 2005

***Study characteristics***		
Methods	Prospective cohort study	
Participants	Intervention group: Women on AED monotherapy or polytherapy Control group: Women with epilepsy not taking AEDs	
Interventions	Intervention groups (monotherapy): 1) CBZ (N = 16) Control group: 1) Women with epilepsy not taking AEDs (N = 18)	
Outcomes	Major congenital abnormalities	
Funding	Not reported	
Country	Jordan	
Notes	Protocol requested ‐ no response received.

**Table:** Cassina 2013

***Study characteristics***		
Methods	Prospective study	
Participants	Intervention group: Women on AED monotherapy or polytherapy Control group: 1) Women with epilepsy not taking AEDs 2) Non‐epileptic women taking AED 3) Healthy women without epilepsy	
Interventions	Intervention group (monotherapy, with known malformation outcomes, limited to women with epilepsy): 1) VPA (N = 45) 2) CBZ (N = 88) 3) PB (N = 67) 4) LTG (N = 26) Control group: 1) Healthy women without epilepsy (N = 867)	
Outcomes	Major congenital malformation	
Funding	Not reported	
Country	Italy	
Notes	Protocol requested ‐ protocol received

**Table:** D'Souza 1991

***Study characteristics***		
Methods	Prospective cohort study	
Participants	Intervention group: Women on AED monotherapy or polytherapy Control group: 1) Women with epilepsy not taking AEDs 2) Women without epilepsy	
Interventions	Intervention group (monotherapy): 1) PHT (N = 22) 2) CBZ (N = 3) 3) PB (N = 4) 4) VPA (N= 1) 5) CZP (N = 1) Control group: 1) Women with epilepsy not taking AEDs (N = 8) 2) Women without epilepsy (N = 62)	
Outcomes	Congenital abnormalities	
Funding	North Western Regional Health Authority	
Country	UK	
Notes	Protocol requested ‐ authors unable to provide protocol but description of study plan given

**Table:** Delmiš 1991

***Study characteristics***		
Methods	Prospective cohort study	
Participants	Intervention group: Women on AED monotherapy or polytherapy Control group: Women with epilepsy not taking AEDs	
Interventions	Intervention group (monotherapy): 1) PB (N = 58) 2) CBZ (N = 18) 3) PRM (N = 9) Control group: 1) Women with epilepsy not taking any AEDs (N = 10)	
Outcomes	Major congenital malformation	
Funding		
Country	Croatia	
Notes	Study authors' contact details could not be found.

**Table:** Denmark Health Record Registers

***Study characteristics***		
Methods	Population database study	
Participants	Intervention group: Women on AED monotherapy or polytherapy Control group: Women with epilepsy not taking AEDs	
Interventions	Intervention groups 1) CBZ (N = 315) 2) OXC (N = 316) 3) VPA (N = 330) 4) LTG (N = 1235) 5) LEV (N = 130) Control group 1) Unexposed to AED (N = 8477)	
Outcomes	Major congenital malformations	
Funding	Danish Epilepsy Association Novo Nordisk Foundation Independent Research Fund Denmark	
Country	Denmark	
Notes

**Table:** Eroglu 2008

***Study characteristics***		
Methods	Prospective cohort study	
Participants	Intervention group: Women on AED monotherapy or polytherapy	
Interventions	Intervention group (monotherapy): 1) CBZ (N = 46) 2) PHT (N = 14) 3) VPA (N = 15) 4) PB (N = 5)	
Outcomes	Congenital malformations	
Funding	Not reported	
Country	Turkey	
Notes	Protocol requested ‐ no response received

**Table:** EURAP 2018

***Study characteristics***		
Methods	Prospective database study	
Participants	Intervention group: Women on AED monotherapy or polytherapy	
Interventions	Intervention groups (monotherapy): 1) CBZ (N = 1957) 2) LTG (N = 2514) 3) PB (N = 294) 4) VPA (N = 1381) 5) LEV (N = 599) 6) OXC (N = 333) 7) TPM (N = 152) 8) PHT (N = 125)	
Outcomes	Congenital malformations	
Funding	Bial, Eisai, GlaxoSmithKline, Janssen‐Cilag, Novartis, Pfizer, Sanofi‐Aventis, UCB, the Netherlands Epilepsy Foundation, Stockholm County Council	
Country	42 countries	
Notes	Protocol requested ‐ no response received. Not included in meta‐analysis due to overlap with other studies (e.g. UK Epilepsy and Pregnancy Register)

**Table:** Fairgrieve 2000

***Study characteristics***		
Methods	Prospective cohort study	
Participants	Intervention group: Women on AED monotherapy or polytherapy Control group: Women with epilepsy not taking AEDs	
Interventions	Intervention group (monotherapy): 1) CBZ (N = 109) 2) VPA (N = 74) Control group: 1) Women with epilepsy not taking AEDs (N = 48)	
Outcomes	Major malformations	
Funding	Wellbeing, Purchasers Clinical Auditors Group	
Country	UK	
Notes	Protocol requested ‐ protocol unavailable

**Table:** Finland Health Record Registers

***Study characteristics***		
Methods	Population database study	
Participants	Intervention group: Women on AED monotherapy or polytherapy Control group: Women with epilepsy not taking AEDs	
Interventions	Intervention groups (monotherapy): 1) CBZ (N = 805) 2) OXC (N = 130) 3) VPA (N = 263) Control group: 1) Women without epilepsy (N = 939)	
Outcomes	Major congenital malformations	
Funding	Ministry of Education	
Country	Finland	
Notes

**Table:** Fröscher 1991

***Study characteristics***		
Methods	Prospective cohort study	
Participants	Intervention group: Women on AED monotherapy or polytherapy	
Interventions	Intervention group (monotherapy): 1) CBZ (N = 31) 2) VPA (N = 12) 3) PB (N = 5) 4) PHT (N = 3)	
Outcomes	Major congenital malformations	
Funding		
Country	Germany	
Notes	Protocol requested ‐ author could not provide protocol but summarised the aims of the study.

**Table:** Garza‐Morales 1996

***Study characteristics***		
Methods	Prospective observational study	
Participants	Intervention group: Women on AED monotherapy or polytherapy Control group: Women with epilepsy not taking AEDs	
Interventions	Intervention group (monotherapy): 1) PHT (N = 27) 2) CBZ (N = 24) 3) VPA (N = 5) Control group 1) Women with epilepsy not taking any AEDs (N = 18)	
Outcomes	Major malformations	
Funding	Not reported	
Country	Spain	
Notes	Study authors' contact details could not be found.

**Table:** Hosny 2021

***Study characteristics***		
Methods	Prospective cohort study	
Participants	Intervention group: Women on AED monotherapy or polytherapy	
Interventions	Intervention groups: 1) LEV (N = 67) 2) CBZ (N = 8)	
Outcomes	Major congenital malformations	
Funding	None	
Country	Egypt	
Notes

**Table:** Israeli Teratogen Service

***Study characteristics***		
Methods	Prospective cohort study	
Participants	Intervention group: Women on AED monotherapy or polytherapy Control group: Women not taking AEDs	
Interventions	Intervention group (monotherapy): 1) VPA (N = 89) 2) CBZ (N = 108) 3) TPM (N = 57) 4) LTG (N = 117) Control group: 1) Pregnant women not taking AEDs (N = 1315)	
Outcomes	Major congenital anomalies	
Funding	None	
Country	Israel	
Notes	Protocol requested ‐ protocol received. Data could not be included in the meta‐analysis for VPA and TPM as number of women taking these AED for non‐epilepsy conditions was > 10%. In the paper on CBZ, data were specifically reported for the women with epilepsy on CBZ and therefore these data could contribute to the meta‐analysis.

**Table:** Italian Lombardy Region Health Register

***Study characteristics***		
Methods	Database study	
Participants	Intervention group: Women on AED monotherapy or polytherapy Control group: Women receiving other pharmacological treatment	
Interventions	Intervention groups (monotherapy): 1) CBZ (N = 154) 2) VPA (N = 131) 3) LTG (N = 56) 4) PRG (N = 63) Control group 1) Non AED exposed (N = 3682)	
Outcomes	Major congenital malformations	
Funding	Part of Project EPIFARM funded by the Lombardy Region	
Country	Italy	
Notes

**Table:** Jimenez 2020

***Study characteristics***		
Methods	Prospective cohort study	
Participants	Intervention group: Women on AED monotherapy or polytherapy	
Interventions	Intervention groups (monotherapy): 1) VPA (N = 17) 2) LTG (N = 19) 3) LEV (N = 12) 4) CBZ (N = 7) 5) TPM (N = 5) 6) OXC (N = 4) 7) PB (N = 2) 8) BNZ (N = 2) 9) LAC (N = 1) 10) ZNS (N = 1)	
Outcomes	Major congenital malformations	
Funding	Not reported	
Country	Spain	
Notes

**Table:** Kaaja 2003

***Study characteristics***		
Methods	Prospective cohort study	
Participants	Intervention group: Women on AED monotherapy or polytherapy Control group: Women with epilepsy not taking AEDs	
Interventions	Intervention group (monotherapy): 1) CBZ (N = 363) 2) PHT (N = 124) 3) VPA (N = 61) 4) PB (N = 5) 5) PRM (N = 6) 6) OXC (N = 9) Control group: 1) Women with epilepsy who were not taking AEDs (N = 237)	
Outcomes	Major malformations	
Funding	Not reported	
Country	Finland	
Notes	Study authors' contact details could not be found.

**Table:** Kaneko 1999

***Study characteristics***		
Methods	Prospective cohort study	
Participants	Intervention group: Women on AED monotherapy or polytherapy Control group: Women with epilepsy not taking AEDs	
Interventions	Intervention group (monotherapy): 1) VPA (N = 81) 2) CBZ (N = 158) 3) PRM (N = 35) 4) PB (N = 79) 5) PHT (N = 132) Control group: 1) Women with epilepsy who were not taking AEDs (N = 98)	
Outcomes	Incidence of congenital malformations	
Funding	Japanese Ministry of Education, Science and Culture, Japan Epilepsy Research Foundation	
Country	Japan, Italy, Canada	
Notes	Study authors' contact details could not be found.

**Table:** Kaur 2020

***Study characteristics***		
Methods	Prospective cohort study	
Participants	Intervention group: Women on AED monotherapy or polytherapy Control group: Women without epilepsy not taking AEDs	
Interventions	Intervention groups (monotherapy): 1) LEV (N = 19) 2) CBZ (N = 7) 3) VPA (N = 3) 4) PHT (N = 2) 5) OXC (N = 1) 6) PB (N = 1) Control group 1) Women without epilepsy (N = 197)	
Outcomes	Major congenital malformations	
Funding	None reported	
Country	India	
Notes

**Table:** Kelly 1984

***Study characteristics***		
Methods	Prospective cohort study	
Participants	Intervention group: Women on AED monotherapy or polytherapy Control group: Women with epilepsy not taking AEDs	
Interventions	Intervention group (monotherapy): 1) PHT (N = 24) 2) PB (N = 6) 3) VPA (N = 4) Control group: 1) Women with untreated epilepsy (N = 23)	
Outcomes	Major abnormality	
Funding	Not reported	
Country	USA	
Notes	Study authors' contact details could not be found.

**Table:** Kerala Epilepsy and Pregnancy Registry

***Study characteristics***		
Methods	Prospective registry study	
Participants	Intervention group: Women on AED monotherapy or polytherapy Control group: Women with epilepsy not taking AEDs	
Interventions	Interevention group (monotherapy): 1) PB (N = 137) 2) CBZ (N = 490) 3) VPA (N = 341) 4) PHT (N = 119) 5) OXC (N = 71) 6) LTG (N = 50) 7) LEV (N = 106) Control group: 1) Women with epilepsy not taking any AEDs (N = 340)	
Outcomes	Congenital malformations	
Funding	Not reported	
Country	India	
Notes	Protocol requested ‐ no response received. Data reported across two papers. The more recent paper reported outcomes pertaining to heart defects only and therefore the numbers available for meta‐analysis for heart defects is substantially higher than that for overall malformation risk and other specific malformation types.

**Table:** Koch 1992

***Study characteristics***		
Methods	Prospective cohort study	
Participants	Intervention group: Women on AED monotherapy or polytherapy Control group: Women without epilepsy not taking AEDs	
Interventions	Intervention group (monotherapy): 1) PB (N = 4) 2) PRM (N = 21) 3) PHT (N = 24) 4) CBZ (N = 9) 5) VPA (N = 14) Control group: 1) Women without epilepsy not taking AEDs (N = 116)	
Outcomes	Major malformations	
Funding		
Country	Germany	
Notes	Study authors' contact details could not be found.

**Table:** Lindhout 1992

***Study characteristics***		
Methods	Prospective cohort study	
Participants	Intervention group: Women on AED monotherapy or polytherapy Control group: Women with epilepsy not taking AEDs	
Interventions	Intervention group (monotherapy): 1) VPA (N = 66) 2) PB (N = 26) 3) CBZ (N = 50) 4) PHT (N = 17) Control group: 1) Women with epilepsy not taking AEDs (N = 28)	
Outcomes	Congenital malformations	
Funding	Ciba‐Geigy, Sanofi, Chemische Industrie Katwijk	
Country	Germany	
Notes	Study authors' details could not be found.

**Table:** Martinez Ferri 2018

***Study characteristics***		
Methods	Prospective cohort study	
Participants	Intervention group: Women on AED monotherapy or polytherapy	
Interventions	Intervention groups (monotherapy): 1) CBZ (N = 148) 2) VPA (N = 112) 3) LTG (N = 111) 4) PB (N = 32)	
Outcomes	Major malformations	
Funding	Not reported	
Country	Spain	
Notes	Protocol requested ‐ no response received

**Table:** Mawer 2010

***Study characteristics***		
Methods	Prospective cohort study	
Participants	Intervention group: Women on AED monotherapy or polytherapy Control group: 1) Women with epilepsy not taking AEDs 2) Women without epilepsy not taking AEDs	
Interventions	Intervention groups (monotherapy): 1) CBZ (N = 74) 2) VPA (N = 57) 3) LTG (N = 40) 4) PHT (N = 7) Control group: 1) Women with epilepsy not taking AEDs (N = 46) 2) Women without epilepsy not taking AEDs (N = 315)	
Outcomes	Major congenital malformations	
Funding	Epilepsy Research UK, US National Institutes of Health, Sanofi Aventis, UK National Institute of Health Research	
Country	UK	
Notes	Protocol requested ‐ protocol received. Overlap in data with NEAD study. Data combined in meta‐analysis along with NEAD data were non‐NEAD data from this study.

**Table:** Meador 2006

***Study characteristics***		
Methods	Prospective cohort study	
Participants	Intervention group: Women on AED monotherapy or polytherapy	
Interventions	Intervention group (monotherapy): 1) CBZ (N = 110) 2) LTG (N = 98) 3) PHT (N = 56) 4) VPA (N = 69)	
Outcomes	Major congenital malformations	
Funding	NIH/NINDS, UK Epilepsy Research Foundation	
Country	USA and UK	
Notes	Protocol requested ‐ protocol received

**Table:** Meischenguiser 2004

***Study characteristics***		
Methods	Prospective registry study	
Participants	Intervention group: Women on AED monotherapy or polytherapy	
Interventions	Intervention group (monotherapy): 1) OXC (N = 35) 2) VPA (N = 21) 3) CBZ (N = 16) 4) PB (N = 5)	
Outcomes	Major malformations	
Funding	Not reported	
Country	Argentina	
Notes	Protocol requested ‐ no response received

**Table:** Melikova 2020

***Study characteristics***		
Methods	Prospective cohort study	
Participants	Intervention group: Women on AED monotherapy or polytherapy Control group: Women without epilepsy not taking AEDs	
Interventions	Intervention groups (monotherapy): 1) CBZ (N = 36) 2) VPA (N = 27) 3) LTG (N = 7) 4) LEV (N = 6) 5) BNZ (N = 3) 6) TPM (N = 2) Control groups: 1) Women without epilepsy not taking AEDs = 277	
Outcomes	Major congenital malformations	
Funding	Not reported	
Country	Azerbaijan	
Notes

**Table:** Milan Study 1999

***Study characteristics***		
Methods	Prospective cohort study	
Participants	Intervention group: Women on AED monotherapy or polytherapy Control group: Women with epilepsy not taking AEDs	
Interventions	Intervention group (monotherapy): 1) PB (N = 83) 2) CBZ (N = 113) 3) PRM (N = 35) 4) PHT (N = 31) 5) VPA (N = 44) 6) CZP (N = 6) Control group: 1) Women with epilepsy not taking AEDs (N = 25)	
Outcomes	Malformations specific to a) cardiac, b) gastrointestinal, c) neural tube defects	
Funding	Not reported	
Country	Italy	
Notes	58 pregnancies that had ended with early spontaneous (N = 38) or early voluntary (N = 20) abortions were excluded from the analysis. Linked to Battino 1992 and Battino 1999 Study authors' contact details count not be found.

**Table:** Miskov 2016

***Study characteristics***		
Methods	Prospective cohort study	
Participants	Intervention group: Women on AED monotherapy or polytherapy Control group: Women without epilepsy not taking AEDs	
Interventions	Intervention groups (monotherapy): 1) LTG (N = 37) 2) VPA (N = 6) 3) PHT (N = 1) 4) PB (N = 3) 5) GBP (N = 2) 6) CBZ (N = 13) 7) OXC (N = 1) Control group: 1) Women without epilepsy (N = 128)	
Outcomes	Major congenital malformations	
Funding	Not reported	
Country	Croatia	
Notes

**Table:** MONEAD 2020

***Study characteristics***		
Methods	Prospective cohort study	
Participants	Intervention group: Women on AED monotherapy or polytherapy Control group: 1) Women with epilepsy not taking AEDs 2) Women without epilepsy not taking AEDs	
Interventions	Intervention groups (monotherapy) 1) CBZ (N = 14) 2) LTG (N = 113) 3) LEV (N = 99) 4) TPM (N = 6) 5) ZNS (N = 13) Control groups: 1) Women with epilepsy not taking AEDs (N = 15) 2) Women without epilepsy not taking AEDs (N = 106)	
Outcomes	Major congenital malformations	
Funding	NIH	
Country	USA	
Notes

**Table:** Montreal Series

***Study characteristics***		
Methods	Prospective cohort study	
Participants	Intervention group: Women on AED monotherapy or polytherapy Control group: Women with epilepsy not taking AEDs	
Interventions	Intervention group (monotherapy): 1) CBZ (N = 32) 2) PHT (N = 44) 3) VPA (N = 15) 4) PB (N = 10) Control group: 1) Women with epilepsy not taking any AEDs (N = 8)	
Outcomes	Congenital malformations	
Funding	Not reported	
Country	Canada	
Notes	Protocol requested ‐ no response received

**Table:** Motherisk Registry

***Study characteristics***		
Methods	Prospective cohort study	
Participants	Intervention group: Women on AED monotherapy or polytherapy Control group: 1) Women with epilepsy not taking AEDs 2) Women without epilepsy not taking medication	
Interventions	Intervention group (monotherapy): 1) PHT (N = 34) 2) CBZ (N = 36) Control group: 1) Women with epilepsy not taking any AEDs (N = 9) 2) Women without epilepsy not taking medication (N = 79)	
Outcomes	Major malformations	
Funding		
Country	Canada	
Notes	Protocol requested ‐ no response received. Data not included in meta‐analysis as non‐epilepsy cases > 10%

**Table:** North American Epilepsy and Pregnancy Register

***Study characteristics***		
Methods	Prospective cohort study	
Participants	Intervention group: Women on AED monotherapy or polytherapy Control group: Women without epilepsy not taking AEDs	
Interventions	Intervention groups (monotherapy): 1) CBZ (N = 1033) 2) LTG (N = 1562) 3) PHT (N = 416) 4) LEV (N = 450) 5) TPM (N = 359) 6) VPA (N = 323) 7) PB (N = 199) 8) OXC (N = 182) 9) GBP (N = 145) 10) ZNS (N = 90) Control group: 1) Women without epilepsy not taking AEDs (N = 442)	
Outcomes	1) Major congenital malformations, most commonly: hypospadias, neural tube defects, cardiovascular anomalies and oral clefts	
Funding		
Country	USA	
Notes	Protocol requested ‐ no response received. Data not available for specific malformations for GBP or ZNS

**Table:** Norwegian Health Record Registers

***Study characteristics***		
Methods	Population database study	
Participants	Women taking ASMs	
Interventions	VPA, CBZ, PB, CZP, LTG, LEV, OXC, TPM	
Outcomes	Major congenital malformations and specific congenital malformations	
Funding	Not reported	
Country	Norway	
Notes	Control rates of MCM came from Veiby and colleagues 2009 paper as specific numbers with MCM were not reported in the 2014 paper.

**Table:** Omtzigt 1992

***Study characteristics***		
Methods	Prospective cohort study	
Participants	Intervention group: Women on AED monotherapy or polytherapy	
Interventions	Intervention group (monotherapy): 1) VPA (N = 60) 2) CBZ (114) 3) PHT (N = 28) 4) PB (N = 18)	
Outcomes	Malformations	
Funding	Not reported	
Country	Netherlands	
Notes	Study authors' contact details could not be found.

**Table:** Pardi 1982

***Study characteristics***		
Methods	Prospective study	
Participants	Intervention group: Women on AED monotherapy or polytherapy	
Interventions	Intervention group (monotherapy): 1) CBZ (N = 2) 2) PB (N = 12) 3) PHT (N = 5) 4) PRM (N = 4) 5) VPA (N = 1)	
Outcomes	Major malformations	
Funding	Not reported	
Country	Italy	
Notes	Study authors' contact details could not be found.

**Table:** Samren 1997

***Study characteristics***		
Methods	Prospective cohort study	
Participants	Intervention group: Women on AED monotherapy or polytherapy Control group: Women without epilepsy not taking AEDs	
Interventions	Intervention groups (monotherapy): 1) CBZ (N = 280) 2) PB (N = 48) 3) PHT (N = 141) 4) PRM (N = 43) 5) VPA (N = 184) Control group: 1) Women without epilepsy N = 158)`	
Outcomes	Major malformations	
Funding	Commissie Landelijk Epilepsie Onderzoek/Nationaal Epilepsie Fonds, and the International League Against Epilepsy through a grant from the Klingenstein Foundation	
Country	Finland, Germany, Netherlands	
Notes	Study authors' contact details could not be found. Not included in meta‐analysis due to overlap with other included studies; reviewed narratively

**Table:** Steegers‐Theunissen 1994

***Study characteristics***		
Methods	Prospective cohort study	
Participants	Intervention group: Women on AED monotherapy or polytherapy Control group: 1) Women with epilepsy not taking AEDs 2) Women without epilepsy who were not taking any medication.	
Interventions	Intervention group (monotherapy): 1) CBZ (N = 39) 2) VPA (N = 19) 3) PB (N = 12) 4) PHT (N = 8) Control group: 1) Women with epilepsy not taking any AEDs (N = 126) 2) no medication (in women without epilepsy) (N = 106)	
Outcomes	Major congenital malformations	
Funding	Dutch ‘Praeventie Fonds'	
Country	Netherlands	
Notes	Protocol requested ‐ no response received

**Table:** Sweden Health Record Registers

***Study characteristics***		
Methods	Database study	
Participants	Intervention group: Women on AED monotherapy or polytherapy	
Interventions	Intervention groups (monotherapy): 1) VPA (N = 268) 2) CBZ (N = 703) 3) PRM (N = 3) 4) PB (N = 7) 5) PHT (N = 103) 6) ETX (N = 8) 7) CZP (N = 48) 8) OXC (N = 4) 9) VGB (N = 3) 10) LTG (N = 90) 11) TPM (N = 1) 12) GBP (N = 18)	
Outcomes	Major malformations	
Funding	Åke Wibergs Stiftelse and KA Wallenbergs Stiftelse to BK, Swedish Medical Research Council, the Pediatric Research Foundation of the Free‐masons in Sweden, and the May Flower Foundation to KW	
Country	Sweden	
Notes

**Table:** Tanganelli 1992

***Study characteristics***		
Methods	Prospective cohort study	
Participants	Intervention group: Women on AED monotherapy or polytherapy Control group: Women without epilepsy not taking AEDs	
Interventions	Intervention groups (monotherapy): 1) PB (N = 63) 2) CBZ (N = 9) 3) VPA (N = 6) Control group: 1) Women without epilepsy not taking AEDs (N = 124)	
Outcomes	Presence of major congenital malformations	
Funding	Not reported	
Country	Italy	
Notes	Study authors' contact details could not be found.

**Table:** UK and Ireland Epilepsy and Pregnancy Register

***Study characteristics***		
Methods	Prospective registry study	
Participants	Intervention group: Women on AED monotherapy or polytherapy Control group: Women with epilepsy not taking AEDs	
Interventions	Intervention groups (monotherapy): 1) CBZ (N = 1657) 2) VPZ (N = 1220) 3) LTG (N = 2098) 4) PHT (N = 106) 5) GBP (N = 31) 6) TPM (N = 70) 7) LEV (N = 304) 8) ZNS (N = 26) Control group: 1) Women with epilepsy who were not taking AEDs (N = 541)	
Outcomes	Congenital malformations	
Funding	Epilepsy Research Foundation, Parke Davis, Glaxo Smith Kline, Eisai, Novartis, Sanofi‐Aventis, Pfizer, Janssen‐Cilag and UCB	
Country	UK	
Notes	Personal communication from the authors provided up‐to‐date figures for PHT and controls. Protocol requested ‐ protocol received

**Table:** UK Clinical Research Practice Database

***Study characteristics***		
Methods	Database study	
Participants	Intervention group: Women on AED monotherapy or polytherapy	
Interventions	Intervention groups (monotherapy): 1) CBZ (N = 311) 2) LTG (N = 98) 3) VPA (N = 225)	
Outcomes	Major congenital malformations	
Funding	GlaxoSmithKline, University of Bath	
Country	UK	
Notes	50‐60% overlap in database coverage with the THIN Network. Narrative review only

**Table:** UK Health Record THIN Register

***Study characteristics***		
Methods	Database study	
Participants	Intervention group: Women on AED monotherapy or polytherapy Control group: Women without epilepsy	
Interventions	Intervention groups (monotherapy): 1) CBZ (N = 334) 2) LTG (N = 357) 3) VPA (N = 229) Control group: 1) Women without epilepsy (N = 239,151)	
Outcomes	Major congenital malformations	
Funding	National Institute for Health Research Health Technology Assessment Program	
Country	UK	
Notes

**Table:** US Medicaid Registers

***Study characteristics***		
Methods	Database study	
Participants	Intervention group: Women on gabapentin Control group: Women with epilepsy not taking AEDs	
Interventions	Intervention group: 1) GBP (N = 347) Control group 1) Non exposed (N = 11,861)	
Outcomes	Major congenital malformations	
Funding	National Institute of Mental Health	
Country	USA	
Notes

**Table:** Waters 1994

***Study characteristics***		
Methods	Prospective cohort study	
Participants	Intervention group: Women on AED monotherapy or polytherapy Control group: Women with epilepsy not taking AEDs	
Interventions	Intervention groups (monotherapy): 1) CBZ (N = 33) 2) PHT (N = 28) 3) PB (N = 21) Control group: 1) Women with epilepsy who were not taking AEDs (N = 15)	
Outcomes	Major malformations	
Funding	Not reported	
Country	USA	
Notes	Protocol requested ‐ author unable to provide protocol	

AED: anti‐epileptic drugs BNZ: benzodiazepine CBZ: carbamazepine CLB: clobazam CZP: clonazepam ETX: ethosuximide GBP: gabapentin LEV: levetiracetam  LTG: lamotrigine OXC: oxcarbazepine PB: phenobarbital  PHT: phenytoin PRG: pregabalin PRM: primidone TGB: tiagabine TPM: topiramate VGB: vigabatrin VPA: sodium valproate

### Characteristics of excluded studies [ordered by study ID]

**Table:**

Study	Reason for exclusion
Annegers 1974	Retrospective methodology
Arteaga‐Vazques 2012	Case‐control study
Arulmozhi 2006	Malformation outcome was not reported by specific AED group.
Baermig 1973	Retrospective methodology
Borthen 2009	No data on malformation outcomes
Bozhinov 2009	Did not report number of women on specific AED monotherapies
Canun‐Serrano 1986	Retrospective methodology
Castilla‐Puentes 2014	Pharmaceutical post‐marketing report with no control group
Diaz‐Romero 1999	Did not report major malformations
Dobos 1985	Retrospective methodology
Dravet 1992	Birth defect register study
Elshove 1971	Mixed prospective and retrospective methodology
EMPiRE Study	No report of malformation outcome by specific AED type
Finland Cohort Study	Did not provide monotherapy AED malformation rates
Fujji 2013	Large numbers of women where the indication was not epilepsy
Galappatty 2018	Did not report malformation outcome by monotherapy AED group
Goujard 1974	Did not provide malformation data for specific AEDs
Hill 1974	Did not provide information on monotherapy malformation cases
Holmes 1994	Retrospective methodology
Jacobsen 2014	Did not include major congential malformation outcomes
Jedrzejczak 2022	No major malformation outcome data
Jones 1989	Did not provide malformation rates by monotherapy exposure
Knight 1975	Did not report ASM‐specific major malformation outcomes
Lamotrigine Pregnancy Registry	No control or comparator group
Laskowska 2002	Did not provide specific monotherapy ASM data
Miskov 2009	No control or comparator group
Monson 1973	Did not report ASM monotherapy major malformation outcomes
Montouris 2003	Mixed prospective and retrospective methodology
Mostacci 2018	Malformation outcomes were not reported for specific ASM groups.
Nakane 1980	Mixed prospective and retrospective methodology
Pearse 1992	No control or comparator group
Richmond 2004	Major malformation rates were not reported by specific monotherapy ASM group.
Robert 1983	Case‐control study
Sabers 2004	Major malformation rates for specific monotherapy ASM groups not reported
Scheuerle 2019	No control or comparator group
Shapiro 1976	Study was congential anomaly case‐control study.
Starveld‐Zimmerman 1975	Retrospective methodology
Tennis 2015	Limited number of women with epilepsy as indication
Torres 1995	Major malformation outcome was not reported for specific monotherapy ASM groups.
Wide 2000	Did not report major malformations
Yeh 2017	No report on malformation outcome by ASM type
Yerby 1992	Did not provide monotherapy major malformation information

AED: anti‐epileptic drugs

### Characteristics of studies awaiting classification [ordered by study ID]

**Table:** Babic 2014

Methods	Prospective, observational, single‐centre study (Serbia)
Participants	21 women with juvenile myoclonic epilepsy (25 pregnancies, mean age 26.4, ranged 22‐34 years)
Interventions	1) Valproate (N = 6) 2) Lamotrigine (N = 8) 3) Topiramate (N = 2) 4) Levetiracetam (N = 4) 5) Polytherapy (N = 5)
Outcomes	1) Congenital malformations 2) Miscarriage 3) Mode of delivery 4) APGAR score
Notes

**Table:** Kaabi 2013

Methods	Retrospective cohort study (Tunisia)
Participants	19 women exposed to AEDs during pregnancy were involved in the study.
Interventions	1) Valproic acid (N = 7) 2) Carbamazepine (N = 5) 3) Phenobarbital (N = 2) 4) Phenytoin (N = 1)
Outcomes	1) Birthweight 2) Malformations
Notes

**Table:** Kutlu 2013

Methods	Prospective cohort study (Canada). Duration: 10 years
Participants	87 pregnancies from 83 women with epilepsy: 1) focal onset with secondary generalised seizures (N = 52) 2) generalised seizures (N = 31)
Interventions	AEDs
Outcomes	1) Spontaneous abortions 2) Major malformations
Notes

**Table:** Lazzaroni Fossati 1986

Methods	Cohort study (Italy)
Participants	36 women with epilepsy
Interventions	1) Phenobarbital 2) Benzodiazepines 3) Diphenylhydantoin 4) Sodium valproate 5) Primidone 6) Carbamazepine 7) Sultiame
Outcomes	1) Congenital malformations
Notes

**Table:** Midi 2014

Methods	Prospective cohort study (Canada). Duration: 1 year
Participants	43 pregnant women with epilepsy
Interventions	1) Lamotrigine 2) Carbamazepine
Outcomes	1) Malformations 2) Spontaneous abortion
Notes

**Table:** Shvartzman 1986

Methods	Cohort study (Hebrew paper)
Participants	14 women with epilepsy
Interventions	1) Hydantoin + phenobarbitone 2) Phenobarbitone 3) Hydantoin 4) Primidone 5) Methosuximide 6) Carbamazepine 7) Diazepam 8) No treatment
Outcomes	1) Congenital malformations 2) Development
Notes

**Table:** Vlasov 2014

Methods	Cohort study (Russia)
Participants	162 pregnant women (49 in 1998 and 113 in 2013) with: 1) Focal epilepsy (N = 124; 38 in 1998 and 86 in 2013) 2) Ideopathic generalised epilepsy (N = 31; 6 in 1998 and 25 in 2013) 3) Undetermined epilepsy (N = 7; 5 in 1998 and 2 in 2013)
Interventions	1) Carbamazepine (N = 48) 2) Valproate (N = 26) 3) Barbiturates (N = 8) 4) Levetiracetam (N = 13) 5) Other drugs (N = 34)
Outcomes	1) Mode of delivery
Notes	

AED: anti‐epileptic drugs APGAR: appearance, pulse, grimace, activity and respiration

## Differences between protocol and review

This update has been undertaken with four alterations to the original protocol.

Firstly, in accordance with the protocol (Pulman 2012), in the first (Adab 2004) and second versions (Weston 2016) of this review, where possible, we conducted meta‐analysis at the monotherapy group level. However, given the clear differentiated pattern of risk for specific ASMs in the previous version of the review, we considered that this approach was no longer reliable and could lead to a misrepresentation of the evidence. Therefore, the inclusion criteria for this review were altered at the current update to report outcomes for specific monotherapy ASM types only and not a group of heterogeneous monotherapy exposures.

Secondly, in the original protocol, we stated that we would also review outcomes by polytherapy combinations, however, given the already numerous comparisons of monotherapies included in this review, outcomes by polytherapy combinations was not feasible here. A separate piece of work is required to delineate the very limited data currently available for specific polytherapy combinations and infant major congenital malformation outcomes.

Thirdly, in the protocol, it was stated that we would look at the specific malformations of a genitourinary and gastrointestinal nature, however, at the point of data extraction, it became apparent that grouping of malformations into this classification was too heterogeneous to do in a way which was worthwhile. After consideration of the included studies, the four most commonly reported specific malformation types were selected and reported on. This will be considered again at the next update.

Finally, due to the small amount of data pertaining to minor malformations identified from the published literature in the second version of this review (Weston 2016), minor malformations were not included in the updated version of the review.

## References

[CD010224-bib-0001] Al Bunyan M, Abo-Talib Z. Outcome of pregnancies in epileptic women: a study in Saudi Arabia. Seizure 1999;8(1):26-9. [PMID: 10091844] 10.1053/seiz.1998.0239

[CD010224-bib-0002] AlSheikh MH. Prevalence of epilepsy in Saudi pregnant women and possible effects of anti-epileptic drugs on pregnancy outcomes. Neurosciences 2020;25(1):32-7. [PMID: 31982897] 10.17712/nsj.2020.1.20190077 PMC8015625

[CD010224-bib-0003] Jazayeri D, Graham J, Hitchcock A, O'Brien TJ, Vajda FJ. Outcomes of pregnancies in women taking antiepileptic drugs for non-epilepsy indications. Seizure 2018;56:111-4. [PMID: 29471258] 10.1016/j.seizure.2018.02.009

[CD010224-bib-0004] Vajda F, Lander C, O'Brien T, Hitchcock A, Graham J, Solinas C, et al. Australian pregnancy registry of women taking antiepileptic drugs. Epilepsia 2004;45(11):1466. [PMID: 15509252] 10.1111/j.0013-9580.2004.451103.x

[CD010224-bib-0005] Vajda F, O'Brien T, Graham J, Lander C, Eadie M. Dose dependence of fetal malformations associated with valproate. Neurology 2013;81(11):999-1003. [PMID: 23911758] 10.1212/WNL.0b013e3182a43e81

[CD010224-bib-0006] Vajda FJ, Eadie MJ. Maternal valproate dosage and foetal malformations. Acta Neurologica Scandinavica 2005;112(3):137-43. [PMID: 16097954] 10.1111/j.1600-0404.2005.00458.x

[CD010224-bib-0007] Vajda FJ, Graham J, Lander CM, O'Brien TJ, Eadie M. Teratogenicity of the newer antiepileptic drugs - the Australian experience. Journal of Clinical Neuroscience 2012;19(1):57-9. [PMID: 22104350] 10.1016/j.jocn.2011.08.003

[CD010224-bib-0008] Vajda FJ, Graham JE, Hitchcock AA, Lander CM, O'Brien TJ, Eadie MJ. Antiepileptic drugs and foetal malformation: analysis of 20 years of data in a pregnancy register. Seizure 2019;65:6-11. [PMID: 30593875] 10.1016/j.seizure.2018.12.006

[CD010224-bib-0009] Vajda FJ, Graham JE, Hitchcock AA, O'Brien TJ, Lander CM, Eadie MJ. Is lamotrigine a significant human teratogen? Observations from the Australian Pregnancy Register. Seizure 2010;19(9):558-61. [PMID: 20739196] 10.1016/j.seizure.2010.07.019

[CD010224-bib-0010] Vajda FJ, Hitchcock A, Graham J, O'Brien T, Lander C, Eadie M. The Australian Register of Antiepileptic Drugs in Pregnancy: the first 1002 pregnancies. Australian and New Zealand Journal of Obstetrics and Gynaecology 2007;47(6):468-74. [PMID: 17991111] 10.1111/j.1479-828X.2007.00781.x

[CD010224-bib-0011] Vajda FJ, Lander CM, Hitchcock A, Graham J, Solinas C, O'Brien T, et al. Changing Australian prescribing patterns for antiepileptic drugs in pregnancy and their possible consequences. Journal of Clinical Neuroscience 2007;14(7):611-7. [PMID: 17400456] 10.1016/j.jocn.2006.04.025

[CD010224-bib-0012] Vajda FJ, O'Brien TJ, Graham J, Lander CM, Eadie MJ. Associations between particular types of fetal malformation and antiepileptic drug exposure in utero. Acta Neurologica Scandinavica 2013;128(4):228-34. [PMID: 23461556] 10.1111/ane.12115

[CD010224-bib-0013] Vajda FJ, O'Brien TJ, Graham J, Lander CM, Eadie MJ. Is carbamazepine a human teratogen? Journal of Clinical Neuroscience 2016;23:34-7. [PMID: 26521756] 10.1016/j.jocn.2015.07.011

[CD010224-bib-0014] Vajda FJ, O'Brien TJ, Graham J, Lander CM, Eadie MJ. Prediction of the hazard of foetal malformation in pregnant women with epilepsy. Epilepsy Research 2014;108(6):1013-7. [PMID: 24880523] 10.1016/j.eplepsyres.2014.04.005

[CD010224-bib-0015] Vajda FJ, O'Brien TJ, Graham JE, Hitchcock AA, Lander CM, Eadie MJ. Antiepileptic drugs, foetal malformations and spontaneous abortions. Acta Neurologica Scandinavica 2017;135(3):360-5. [PMID: 27573510] 10.1111/ane.12672

[CD010224-bib-0016] Vajda FJ, O'Brien TJ, Graham JE, Hitchcock AA, Lander CM, Eadie MJ. Preexisting illness, fetal malformation, and seizure control rates in pregnant women with epilepsy. Epilepsy & Behavior 2020;103(Pt A):106481. [PMID: 31711866] 10.1016/j.yebeh.2019.106481

[CD010224-bib-0017] Vajda FJ, O'Brien TJ, Graham JE, Hitchcock AA, Lander CM, Eadie MJ. Pregnancy after valproate withdrawal - fetal malformations and seizure control. Epilepsia 2020;61(5):944-50. [PMID: 32314363] 10.1111/epi.16505

[CD010224-bib-0018] Vajda FJ, O'Brien TJ, Graham JE, Hitchcock AA, Lander CM, Eadie MJ. Valproate-associated foetal malformations - rates of occurrence, risks in attempted avoidance. Acta Neurologica Scandinavica 2019;139(1):42-8. [PMID: 30109700] 10.1111/ane.13005

[CD010224-bib-0019] Vajda FJ, O'Brien TJ, Hitchcock A, Graham J, Cook M, Lander C, et al. Critical relationship between sodium valproate dose and human teratogenicity: results of the Australian register of anti-epileptic drugs in pregnancy. Journal of Clinical Neuroscience 2004;11(8):854-8. [PMID: 15519862] 10.1016/j.jocn.2004.05.003

[CD010224-bib-0020] Vajda FJ, O'Brien TJ, Hitchcock A, Graham J, Lander C. The Australian Registry of Anti-Epileptic Drugs in Pregnancy: experience after 30 months. Journal of Clinical Neuroscience 2003;10(5):543-9. [PMID: 12948456] 10.1016/s0967-5868(03)00158-9

[CD010224-bib-0021] Vajda FJ, O'Brien TJ, Lander CM, Graham J, Eadie MJ. Antiepileptic drug combinations not involving valproate and the risk of fetal malformations. Epilepsia 2016;57(7):1048-52. [PMID: 27265509] 10.1111/epi.13415

[CD010224-bib-0022] Vajda FJ, O'Brien TJ, Lander CM, Graham J, Roten A, Eadie MJ. Teratogenesis in repeated pregnancies in antiepileptic drug-treated women. Epilepsia 2013;54(1):181-6. [PMID: 22882134] 10.1111/j.1528-1167.2012.03625.x

[CD010224-bib-0023] Bag S, Behari M, Ahuja GK, Karmarkar MG. Pregnancy and epilepsy. Journal of Neurology 1989;236(5):311-3. [PMID: 2760652] 10.1007/BF00314466

[CD010224-bib-0024] Barqawi R. Evaluation of antiepileptic drugs in pregnancy in a Jordanian army hospital. Eastern Mediterranean Health Journal 2005;11(4):601-5. [PMID: 16700374]

[CD010224-bib-0025] Cassina M, Dilaghi A, Di Gianantonio E, Cesari E, De Santis M, Mannaioni G, et al. Pregnancy outcome in women exposed to antiepileptic drugs: teratogenic role of maternal epilepsy and its pharmacologic treatment. Reproductive Toxicology 2013;39:50-7. [PMID: 23591043] 10.1016/j.reprotox.2013.04.002

[CD010224-bib-0026] D'Souza SW, Robertson IG, Donnai D, Mawer G. Fetal phenytoin exposure, hypoplastic nails, and jitteriness. Archives of Disease in Childhood 1991;65(3):320-4. [PMID: 2025009] 10.1136/adc.66.3.320 PMC1792889

[CD010224-bib-0027] Đelmiš J, Dražančić A, Tkalčević T, Ivanišević M. Epilepsy in pregnancy [Epilepsija i trudnoća]. Jugoslavenska Ginekologija i Perinatologija 1991;31(1-2):23-6. [PMID: 1875716]

[CD010224-bib-0028] Christensen J, Trabjerg BB, Sun Y, Gilhus NE, Bjork M-H, Tomson T, et al. Prenatal exposure to valproate and risk of congenital malformations - could we have known earlier? A population-based cohort study. Epilepsia 2021;62(12):2981-93. [PMID: 34585373] 10.1111/epi.17085

[CD010224-bib-0029] Mølgaard-Nielsen D, Hviid A. Newer-generation antiepileptic drugs and the risk of major birth defects. JAMA 2011;305(19):1996-2002. [PMID: 21586715] 10.1001/jama.2011.624

[CD010224-bib-0030] Eroğlu E, Gökçil Z, Bek S, Ulaş UH, Odabaşi Z. Pregnancy and teratogenicity of antiepileptic drugs. Acta Neurologica Belgica 2008;108(2):53-7. [PMID: 18795597]

[CD010224-bib-0031] Huber-Mollema Y, Van Iterson L, Oort FJ, Lindhout D, Rodenburg R. Neurocognition after prenatal levetiracetam, lamotrigine, carbamazepine or valproate exposure. Journal of Neurology 2020;267(6):1724-36. [PMID: 32112258] 10.1007/s00415-020-09764-w PMC7293688

[CD010224-bib-0032] Tomson T, Battino D, Bonizzoni E, Craig J, Lindhout D, Perucca E, et al. Comparative risk of major congenital malformations with eight different antiepileptic drugs: a prospective cohort study of the EURAP registry. Lancet Neurology 2018;17(6):530-8. [PMID: 29680205] 10.1016/S1474-4422(18)30107-8

[CD010224-bib-0033] Tomson T, Battino D, Bonizzoni E, Craig J, Lindhout D, Perucca E, et al. Declining malformation rates with changed antiepileptic drug prescribing: an observational study. Neurology 2019;93(9):e831-40. [PMID: 31391249] 10.1212/WNL.0000000000008001

[CD010224-bib-0034] Tomson T, Battino D, Bonizzoni E, Craig J, Lindhout D, Perucca E, et al. Dose-dependent teratogenicity of valproate in mono- and polytherapy: an observational study. Neurology 2015;85(10):866-72. [PMID: 26085607] 10.1212/WNL.0000000000001772

[CD010224-bib-0035] Tomson T, Battino D, Bonizzoni E, Craig J, Lindhout D, Sabers A, et al. Dose-dependent risk of malformations with antiepileptic drugs: an analysis of data from the EURAP epilepsy and pregnancy registry. Lancet Neurology 2011;10(7):609-17. [PMID: 21652013] 10.1016/S1474-4422(11)70107-7

[CD010224-bib-0036] Tomson T, Battino D, Bonizzoni E, Craig JJ, Lindhout D, Perucca E, et al. Antiepileptic drugs and intrauterine death: a prospective observational study from EURAP. Neurology 2015;85(7):580-8. [PMID: 26187231] 10.1212/WNL.0000000000001840

[CD010224-bib-0037] Fairgrieve SD, Jackson M, Jonas P, Walshaw D, White K, Montgomery TL, et al. Population based, prospective study of the care of women with epilepsy in pregnancy. British Medical Journal 2000;321(7262):674-5. [PMID: 10987772] 10.1136/bmj.321.7262.674 PMC27482

[CD010224-bib-0038] Artama M, Auvinen A, Raudaskoski T, Isojärvi I, Isojärvi J. Antiepileptic drug use of women with epilepsy and congenital malformations in offspring. Neurology 2005;64(11):1874-8. [PMID: 15955936] 10.1212/01.WNL.0000163771.96962.1F

[CD010224-bib-0039] Artama M, Ritvanen A, Gissler M, Isojärvi J, Auvinen A. Congenital structural anomalies in offspring of women with epilepsy - a population-based cohort study in Finland. International Journal of Epidemiology 2006;35(2):280-7. [PMID: 16280367] 10.1093/ije/dyi234

[CD010224-bib-0040] Fröscher W, Herrmann R, Niesen M, Bülau P, Penin H, Hildenbrand G. The course of pregnancy and teratogenicity of antiepileptic agents in 66 patients with epilepsy [Untersuchungen zum schwangerschaftsverlauf und zur teratogenität der antiepileptika bei 66 epilepsie-patientinnen]. Schweizer Archiv fur Neurologie und Psychiatrie 1991;142(5):389-407. [PMID: 1721236]

[CD010224-bib-0041] Garza-Morales S, Ibarra-Puig JM, Poblano-Luna A, Gilda Mayén-Molina D, Córdova-López S. Epilepsy and pregnancy: prospective study of 100 cases [Epilepsia y embarazo. Estudio prospectivo 100 casos]. Ginecología y Obstetricia de México 1996;64:449-54. [PMID: 8974948]

[CD010224-bib-0042] Hosny H, Elkattan M, Zaki MA, Ramzy GM, Magdy R, Abo Al-Azayem S. Risk factors of fetal deaths and major birth defects in newborns of women with epilepsy: an Egyptian prospective study. Epilepsy & Behavior 2021;123:108251. [PMID: 34411949] 10.1016/j.yebeh.2021.108251

[CD010224-bib-0043] Diav-Citrin O, Shechtman S, Arnon J, Ornoy A. Is carbamazepine teratogenic? A prospective controlled study of 210 pregnancies. Neurology 2001;57(2):321-4. [PMID: 11468320] 10.1212/wnl.57.2.321

[CD010224-bib-0044] Diav-Citrin O, Shechtman S, Bar-Oz B, Cantrell D, Arnon J, Ornoy A. Pregnancy outcome after in utero exposure to valproate: evidence of dose relationship in teratogenic effect. CNS Drugs 2008;22(4):325-34. [PMID: 18336060] 10.2165/00023210-200822040-00004

[CD010224-bib-0045] Diav-Citrin O, Shechtman S, Zvi N, Finkel-Pekarsky V, Ornoy A. Is it safe to use lamotrigine during pregnancy? A prospective comparative observational study. Birth Defects Research 2017;109(15):1196-1203. [PMID: 28657171] 10.1002/bdr2.1058

[CD010224-bib-0046] Ornoy A, Cohen E. Outcome of children born to epileptic mothers treated with carbamazepine during pregnancy. Archives of Disease in Childhood 1996;75(6):517-20. [PMID: 9014606] 10.1136/adc.75.6.517 PMC1511819

[CD010224-bib-0047] Ornoy A, Zvi N, Arnon J, Wajnberg R, Shechtman S, Diav-Citrin O. The outcome of pregnancy following topiramate treatment: a study on 52 pregnancies. Reproductive Toxicology 2008;25(3):388-9. [PMID: 18424066] 10.1016/j.reprotox.2008.03.001

[CD010224-bib-0048] Putignano D, Clavenna A, Campi R, Canevini MP, Vignoli A, Battino D, et al. Perinatal outcome and healthcare resource utilization in the first year of life after antiepileptic exposure during pregnancy. Epilepsy & Behavior 2019;92:14-7. [PMID: 30599457] 10.1016/j.yebeh.2018.09.033

[CD010224-bib-0049] Jiménez M, Grau-López L, Ciurans J, García-Esperón C, Fumanal A, Barambio S, Chíes E, Codina M, Becerra JL. Epilepsy and pregnancy. Factors associated with epileptic seizures during pregnancy. Neurologia (Engl Ed) 2020 [Epub ahead of print];38(2):106-113. [DOI: 10.1016/j.nrl.2020.04.024] [PMID: 36162697]

[CD010224-bib-0050] Kaaja E, Kaaja R, Hiilesmaa V. Major malformations in offspring of women with epilepsy. Neurology 2003;60(4):575-9. [PMID: 12601095] 10.1212/01.wnl.0000044157.28073.dc

[CD010224-bib-0051] Fukushima Y, Nakamura Y, Ogawa Y, Saito Y, Kan R, Kumashiro H, et al. Teratogenicity of antiepileptic drugs: is the prevention possible? Japanese Journal of Psychiatry and Neurology 1991;45(2):478-81. [PMID: 1762253] 10.1111/j.1440-1819.1991.tb02526.x

[CD010224-bib-0052] Kaneko S, Battino D, Andermann E, Wada K, Kan R, Takeda A, et al. Congenital malformations due to antiepileptic drugs. Epilepsy Research 1999;33(2-3):141-58. [PMID: 10094426] 10.1016/s0920-1211(98)00084-9

[CD010224-bib-0053] Kaneko S, Otani K, Fukushima Y, Ogawa Y, Nomura Y, Ono T, et al. Teratogenicity of antiepileptic drugs: analysis of possible risk factors. Epilepsia 1988;29(4):459-67. [PMID: 3134192] 10.1111/j.1528-1157.1988.tb03746.x

[CD010224-bib-0054] Kaneko S, Otani K, Kondo T, Fukushima Y, Kan R, Takeda A, et al. Teratogenicity of antiepileptic drugs and drug specific malformations. Japanese Journal of Psychiatry and Neurology 1993;47(2):306-8. [PMID: 8271575] 10.1111/j.1440-1819.1993.tb02084.x

[CD010224-bib-0055] Kaneko S, Otani K, Kondo T, Fukushima Y, Nakamura Y, Ogawa Y, et al. Malformation in infants of mothers with epilepsy receiving antiepileptic drugs. Neurology 1992;42(4 Suppl 5):68-74. [PMID: 1574179]

[CD010224-bib-0056] Kaur TP, Sahu L, Rathore AM, Bhasin S. Obstetric outcomes in pregnant women with seizure disorder: a hospital-based, longitudinal study. Turkish Journal of Obstetrics & Gynecology 2020;17(3):161-9. [PMID: 33072419] 10.4274/tjod.galenos.2020.87300 PMC7538818

[CD010224-bib-0057] Kelly TE, Edwards P, Rein M, Miller JQ, Dreifuss FE. Teratogenicity of anticonvulsant drugs II: a prospective study. American Journal of Medical Genetics 1984;19(3):435-43. [PMID: 6507489] 10.1002/ajmg.1320190303

[CD010224-bib-0058] Begum S, Sarma SP, Thomas SV. Malformation in index pregnancy in women with epilepsy is not followed by recurrence in subsequent pregnancy. Epilepsia 2013;54(12):e163-7. [PMID: 24138300] 10.1111/epi.12411

[CD010224-bib-0059] Keni RR, Jose M, Reshma AS, Baishya J, Sankara Sarma P, Thomas SV. Anti-epileptic drug and folic acid usage during pregnancy, seizure and malformation outcomes: changes over two decades in the Kerala Registry of Epilepsy and Pregnancy. Epilepsy Research 2020;159:106250. [PMID: 31855827] 10.1016/j.eplepsyres.2019.106250

[CD010224-bib-0060] Keni RR, Jose M, Sarma PS, Thomas SV, Kerala Registry of Epilepsy and Pregnancy Study Group. Teratogenicity of antiepileptic dual therapy: dose-dependent, drug-specific, or both? Neurology 2018;90(9):e790-6. [PMID: 29429975] 10.1212/WNL.0000000000005031

[CD010224-bib-0061] Seshachala BB, Jose M, Lathikakumari AM, Murali S, Kumar AS, Thomas SV. Valproate usage in pregnancy: an audit from the Kerala Registry of Epilepsy and Pregnancy. Epilepsia 2021;62(5):1141-7. [PMID: 33782943] 10.1111/epi.16882

[CD010224-bib-0062] Thomas SV, Ajaykumar B, Sindhu K, Francis E, Namboodiri N, Sivasankaran S, et al. Cardiac malformations are increased in infants of mothers with epilepsy. Pediatric Cardiology 2008;29(3):604-8. [PMID: 18188637] 10.1007/s00246-007-9161-4

[CD010224-bib-0063] Thomas SV, Indrani L, Devi GC, Jacob S, Beegum J, Jacob PP, et al. Pregnancy in women with epilepsy: preliminary results of Kerala registry of epilepsy and pregnancy. Neurology India 2001;49(1):60-6. [PMID: 11303244]

[CD010224-bib-0064] Thomas SV, Jeemon P, Pillai R, Jose M, Lalithakumari AM, Murali S, et al. Malformation risk of new anti-epileptic drugs in women with epilepsy; observational data from the Kerala Registry of Epilepsy and Pregnancy (KREP). Seizure 2021;93:127-32. [PMID: 34740142] 10.1016/j.seizure.2021.10.015

[CD010224-bib-0065] Thomas SV, Jose M, Divakaran S, Sankara Sarma P. Malformation risk of antiepileptic drug exposure during pregnancy in women with epilepsy: results from a pregnancy registry in South India. Epilepsia 2017;58(2):274-81. [PMID: 28084641] 10.1111/epi.13632

[CD010224-bib-0066] Jäger-Roman E, Deichl A, Jakob S, Hartmann AM, Koch S, Rating D, et al. Fetal growth, major malformations, and minor anomalies in infants born to women receiving valproic acid. Journal of Pediatrics 1986;108(6):997-1004. [PMID: 3086531] 10.1016/s0022-3476(86)80949-0

[CD010224-bib-0067] Koch S, Gopfert-Geyer I, Jager-Roman E, Jakob S, Huth H, Hartmann A, et al. Anti-epileptic agents during pregnancy: a prospective study on the course of pregnancy, malformations and child development [Antiepileptika wahrend der schwangerschaft. Eine prospektive studie uber schwangerschaftsverlauf, fehlbildungen und kindliche entwicklung]. Deutsche Medizinische Wochenschrift 1983;108(7):250-7. [PMID: 6402356] 10.1055/s-2008-1069536

[CD010224-bib-0068] Koch S, Lösche G, Jager-Romän E, Jakob S, Rating D, Deichl A, et al. Major and minor birth malformations and antiepileptic drugs. Neurology 1992;42(4 Suppl 5):83-8. [PMID: 1574183]

[CD010224-bib-0069] Kuhnz W, Jäger-Roman E, Rating D, Deichl A, Kunze J, Helge H, et al. Carbamazepine and carbamazepine-10, -11 epoxide during pregnancy and postnatal period in epileptic mother and their nursed infants: pharmacokinetics and clinical effects. Pediatric Pharmacology 1983;3(3-4):199-208. [PMID: 6677873]

[CD010224-bib-0070] Kuhnz W, Koch S, Jakob S, Hartmann A, Helge H, Nau H. Ethosuximide in epileptic women during pregnancy and lactation period: placental transfer, serum concentrations in nursed infants and clinical status. British Journal of Clinical Pharmacology 1984;18(5):671-7. [PMID: 6508976] 10.1111/j.1365-2125.1984.tb02528.x PMC1463560

[CD010224-bib-0071] Rating D, Jager-Roman E, Koch S, Deichl A, Hartmann H, Jakob S, et al. Major malformations and minor anomalies in the offspring of epileptic parents: the role of antiepileptic drugs. In: Nau H, Scott WJ Jr, editors(s). Pharmacokinetics in Teratogenesis. Vol. 1. CRC Press, 1987:205-23. [ISBN: 978-0849368738]

[CD010224-bib-0072] Rating D, Nau H, Jäger-Roman E, Göpfert-Geyer I, Koch S, Beck-Mannagetta G, et al. Teratogenic and pharmacokinetic studies of primidone during pregnancy and in the offspring of epileptic women. Acta Paediatrica Scandinavia 1982;71(2):301-11. [PMID: 7136638] 10.1111/j.1651-2227.1982.tb09418.x

[CD010224-bib-0073] Lindhout D, Höppener RJ, Meinardi H. Teratogenicity of antiepileptic drug combinations with special emphasis on epoxidation (of carbamazepine). Epilepsia 1984;25(1):77-83. [PMID: 6692794] 10.1111/j.1528-1157.1984.tb04158.x

[CD010224-bib-0074] Lindhout D, Meinardi H, Meijer JW, Nau H. Antiepileptic drugs and teratogenesis in two consecutive cohorts: changes in prescription policy paralleled by changes in pattern of malformations. Neurology 1992;42(4 Suppl 5):94-110. [PMID: 1574185]

[CD010224-bib-0075] Martinez Ferri M, Pena Mayor P, Perez Lopez-Fraile I, Escartin Siquier A, Martin Moro M, Forcadas Berdusan M, et al. Comparative study of antiepileptic drug use during pregnancy over a period of 12 years in Spain. Efficacy of the newer antiepileptic drugs lamotrigine, levetiracetam, and oxcarbazepine. Neurologia 2018;33(2):78-84. [PMID: 27452623] 10.1016/j.nrl.2016.05.004

[CD010224-bib-0076] Martinez Ferri M, Pena Mayor P, Perez Loppez-Fraile I, Castro Vilanova MD, Escartin Siquier A, Martin Moro M, et al. Malformations and fetal death in the Spanish antiepileptic drug and pregnancy registry: results at 6 years [Malformaciones y muerte fetal en el registro espanol de farmacos antiepilepticos y embarazo: resultados a los 6 anos]. Neurologia 2009;24(6):360-5. [PMID: 19798601]

[CD010224-bib-0077] Mawer G, Briggs M, Baker GA, Bromley R, Coyle H, Eatock J, et al. Pregnancy with epilepsy: obstetric and neonatal outcome of a controlled study. Seizure 2010;19(2):112-9. [PMID: 20036166] 10.1016/j.seizure.2009.11.008 PMC2823982

[CD010224-bib-0078] Meador KJ, Baker GA, Finnell RH, Kalayjian LA, Liporace JD, Loring DW, et al. In utero antiepileptic drug exposure: fetal death and malformations. Neurology 2006;67(3):407-12. [PMID: 16894099] 10.1212/01.wnl.0000227919.81208.b2 PMC1986655

[CD010224-bib-0079] Meischenguiser R, D'Giano CH, Ferraro SM. Oxcarbazepine in pregnancy: clinical experience in Argentina. Epilepsy & Behaviour 2004;5(2):163-7. [PMID: 15123016] 10.1016/j.yebeh.2003.11.020

[CD010224-bib-0080] Melikova S, Bagirova H, Magalov S. The impact of maternal epilepsy on delivery and neonatal outcomes. Child's Nervous System 2020;36(4):775-82. [PMID: 31786631] 10.1007/s00381-019-04435-2

[CD010224-bib-0081] Battino D, Binelli S, Caccamo ML, Canevini MP, Canger R, Como ML, et al. Malformations in offspring of 305 epileptic women: a prospective study. Acta Neurologica Scandinavica 1992;85(3):204-7. [PMID: 1575005] 10.1111/j.1600-0404.1992.tb04029.x

[CD010224-bib-0082] Battino D, Kaneko S, Andermann E, Avanzini G, Canevini MP, Canger R, et al. Intrauterine growth in the offspring of epileptic women: a prospective multicentre study. Epilepsy Research 1999;36(1):53-60. [PMID: 10463850] 10.1016/s0920-1211(99)00020-0

[CD010224-bib-0083] Canger R, Battino D, Canevini MP, Fumarola C, Guidolin L, Vignoli A, et al. Malformations in offspring of women with epilepsy: a prospective study. Epilepsia 1999;40(9):1231-6. [PMID: 10487185] 10.1111/j.1528-1157.1999.tb00851.x

[CD010224-bib-0084] Miskov S, Gjergja Juraski R, Mikula I, Basic S, Bosnjak Pasic M, Kosec V, et al. The Croatian model of integrative prospective management of epilepsy and pregnancy. Acta Clinica Croatica 2016;55(4):535-48. [PMID: 29116720] 10.20471/acc.2016.55.04.02

[CD010224-bib-0085] Meador KJ, Pennell PB, May RC, Van Marter L, McElrath TF, Brown C, et al. Fetal loss and malformations in the MONEAD study of pregnant women with epilepsy. Neurology 2020;94(14):e1502-11. [PMID: 31806691] 10.1212/WNL.0000000000008687 PMC7251524

[CD010224-bib-0086] Van Marter LJ, Pennell PB, Brown C, Hartman AL, May RC, McElrath T, et al. Neonatal outcomes in the MONEAD study of pregnant women with epilepsy. Journal of Pediatrics 2021;7:100073. [DOI: 10.1016/j.ympdx.2021.100073] PMC10210636 37234096

[CD010224-bib-0087] Dansky LV, Andermann E, Rosenblatt D, Sherwin AL, Andermann F. Anticonvulsants, folate levels, and pregnancy outcome: a prospective study. Annals of Neurology 1987;21(2):176-82. [PMID: 3827226] 10.1002/ana.410210210

[CD010224-bib-0088] Oguni M, Dansky L, Andermann E, Sherwin A, Andermann F. Improved pregnancy outcome in epileptic women in the last decade: relationship to maternal anticonvulsant therapy. Brain & Development 1992;14(6):371-80. [PMID: 1492649] 10.1016/s0387-7604(12)80343-3

[CD010224-bib-0089] Gladstone DJ, Bologa M, Maguire C, Pastuszak A, Koren G. Course of pregnancy and fetal outcome following maternal exposure to carbamazepine and phenytoin: a prospective study. Reproductive Toxicology 1992;6(3):257-61. [PMID: 1591483] 10.1016/0890-6238(92)90181-r

[CD010224-bib-0090] Nulman I, Scolnik D, Chitayat D, Farkas LD, Koren G. Findings in children exposed in utero to phenytoin and carbamazepine monotherapy: independent effects of epilepsy and medications. American Journal of Medical Genetics 1997;68(1):18-24. [PMID: 8986270]

[CD010224-bib-0091] Bokhari A, Coull BA, Holmes LB. Effect of prenatal exposure to anticonvulsant drugs on dermal ridge patterns of fingers. Teratology 2002;66(1):19-23. [PMID: 12115776] 10.1002/tera.10044

[CD010224-bib-0092] Bromfield EB, Dworetzky BA, Wyszynski DF, Smith CR, Baldwin EJ, Holmes LB. Valproate teratogenicity and epilepsy syndrome. Epilepsia 2008;49(12):2122-4. [PMID: 18557775] 10.1111/j.1528-1167.2008.01696.x

[CD010224-bib-0093] Hernandez-Diaz S, Smith CR, Shen A, Mittendorf R, Hauser WA, Yerby M, et al. Comparative safety of antiepileptic drugs during pregnancy. Neurology 2012;78(21):1692-9. [PMID: 22551726] 10.1212/WNL.0b013e3182574f39

[CD010224-bib-0094] Hernández-Díaz S, Mittendorf R, Smith CR, Hauser WA, Yerby M, Holmes LB, et al. Association between topiramate and zonisamide use during pregnancy and low birth weight. Obstetrics and Gynecology 2014;123(1):21-8. [PMID: 24463659] 10.1097/AOG.0000000000000018

[CD010224-bib-0095] Holmes LB, Baldwin EJ, Smith CR, Habecker E, Glassman L, Wong SL, et al. Increased frequency of isolated cleft palate in infants exposed to lamotrigine during pregnancy. Neurology 2008;70(22 Pt 2):2152-8. [PMID: 18448870] 10.1212/01.wnl.0000304343.45104.d6

[CD010224-bib-0096] Holmes LB, Coull BA, Dorfman J, Rosenberger PB. The correlation of deficits in IQ with midface and digit hypoplasia in children exposed in utero to anticonvulsant drugs. Journal of Pediatrics 2005;146(1):118-22. [PMID: 15644835] 10.1016/j.jpeds.2004.08.048

[CD010224-bib-0097] Holmes LB, Mittendorf R, Shen A, Smith CR, Hernandez-Diaz S. Fetal effects of anticonvulsant polytherapies: different risks from different drug combinations. Archives of Neurology 2011;68(10):1275-81. [PMID: 21670385] 10.1001/archneurol.2011.133

[CD010224-bib-0098] Holmes LB, Rosenberger PB, Harvey EA, Khoshbin S, Ryan L. Intelligence and physical features of children of women with epilepsy. Teratology 2000;61(3):196-202. [PMID: 10661909] 10.1002/(SICI)1096-9926(200003)61:3<196::AID-TERA7>3.0.CO;2-T

[CD010224-bib-0099] Holmes LB, Wyszynski DF. North American Antiepileptic Drug Pregnancy Registry. Epilepsia 2004;45(11):1465. [PMID: 15509251] 10.1111/j.0013-9580.2004.451102.x

[CD010224-bib-0100] Wyszynski DF, Nambisan M, Surve T, Alsdorf RM, Smith CR, Holmes LB, et al. Increased rate of major malformations in offspring exposed to valproate during pregnancy. Neurology 2005;64(6):961-5. [PMID: 15781808] 10.1212/01.WNL.0000154516.43630.C5

[CD010224-bib-0101] Borthen I, Eide MG, Veiby G, Daltveit AK, Gilhus NE. Complications during pregnancy in women with epilepsy: population-based cohort study. British Journal of Obstetrics and Gynecology 2009;116(13):1736–42. [PMID: 19781049] 10.1111/j.1471-0528.2009.02354.x

[CD010224-bib-0102] Veiby G, Daltveit AK, Engelsen BA, Gilhus NE. Fetal growth restriction and birth defects with newer and older antiepileptic drugs during pregnancy. Journal of Neurology 2014;261(13):579-88. [PMID: 24449062] 10.1007/s00415-013-7239-x

[CD010224-bib-0103] Veiby G, Daltveit AK, Engelsen BA, Gilhus NE. Pregnancy, delivery, and outcome for the child in maternal epilepsy. Epilepsia 2009;50(9):2130-9. [PMID: 19490036] 10.1111/j.1528-1167.2009.02147.x

[CD010224-bib-0104] Omtzigt JG, Los FJ, Grobbee DE, Pijpers L, Jahoda MG, Brandenburg H, et al. The risk of spina bifida aperta after first-trimester exposure to valproate in a prenatal cohort. Neurology 1992;42(4 Suppl 5):119-25. [PMID: 1574165]

[CD010224-bib-0105] Omtzigt JG, Los FJ, Hagenaars AM, Stewart PA, Sachs ES, Lindhout D. Prenatal diagnosis of spina bifida aperta after first-trimester valproate exposure. Prenatal Diagnosis 1992;12(11):893-7. [PMID: 1283633] 10.1002/pd.1970121107

[CD010224-bib-0106] Pardi G, Como ML, De Giambattista M, Oldrini A, Pifarotti G. Epilepsy and pregnancy: obstetrical aspects of a prospective multidisciplinary study [Epilessia e gravidanza: aspetti ostetrici di uno studio prospettico multidisciplinare]. Annali di Ostetricia, Ginecologia, Medicina Perinatale 1982;103(4):254-63. [PMID: 7181355]

[CD010224-bib-0107] Samrén EB, Van Duijn CM, Koch S, Hiilesmaa VK, Klepel H, Bardy AH, et al. Maternal use of antiepileptic drugs and the risk of major congenital malformations: a joint European prospective study of human teratogenesis associated with maternal epilepsy. Epilepsia 1997;38(9):981-90. [PMID: 9579936] 10.1111/j.1528-1157.1997.tb01480.x

[CD010224-bib-0108] Steegers-Theunissen RP, Renier WO, Borm GF, Thomas CM, Merkus HM, Op de Coul DA, et al. Factors influencing the risk of abnormal pregnancy outcome in epileptic women: a multi-centre prospective study. Epilepsy Research 1994;18(3):261-9. [PMID: 7805647] 10.1016/0920-1211(94)90046-9

[CD010224-bib-0109] Källén B. A register study of maternal epilepsy and delivery outcome with special reference to drug use. Acta Neurologica Scandinavica 1986;73(3):253-9. [PMID: 3716762] 10.1111/j.1600-0404.1986.tb03271.x

[CD010224-bib-0110] Wide K, Winbladh B, Källén B. Major malformations in infants exposed to antiepileptic drugs in utero, with emphasis on carbamazepine and valproic acid: a nation-wide, population-based register study. Acta Paediatrica 2004;93(2):174-6. [PMID: 15046269] 10.1080/08035250310021118

[CD010224-bib-0111] Regesta G, Tanganelli P. The risk of malformations and developmental disturbances in children exposed to antiepileptic drugs: a prospective controlled study. Bollettino Lega Italiana contro l'Epilessia 1996;95/96:351-4.

[CD010224-bib-0112] Tanganelli P, Regesta G. Epilepsy, pregnancy, and major birth anomalies: an Italian prospective, controlled study. Neurology 1992;42(4 Suppl 5):89-93. [PMID: 1574184]

[CD010224-bib-0113] Campbell E, Devenney E, Morrow J, Russell A, Smithson WH, Parsons L, et al. Recurrence risk of congenital malformations in infants exposed to antiepileptic drugs in utero. Epilepsia 2013;54(1):165-71. [PMID: 23167802] 10.1111/epi.12001

[CD010224-bib-0114] Campbell E, Kennedy F, Russell A, Smithson WH, Parsons L, Morrison PJ, et al. Malformation risks of antiepileptic drug monotherapies in pregnancy: updated results from the UK and Ireland Epilepsy and Pregnancy Registers. Journal of Neurology, Neurosurgery & Psychiatry 2014;85(9):1029-34. [PMID: 24444855] 10.1136/jnnp-2013-306318

[CD010224-bib-0115] Hunt S, Craig J, Russell A, Guthrie E, Parsons L, Robertson I, et al. Levetiracetam in pregnancy: preliminary experience from the UK Epilepsy and Pregnancy Register. Neurology 2006;67(10):1876-9. [PMID: 17130430] 10.1212/01.wnl.0000244491.48937.55

[CD010224-bib-0116] Hunt S, Russell A, Smithson WH, Parsons L, Robertson I, Waddell R, et al. Topiramate in pregnancy. Neurology 2008;71(4):272-6. [PMID: 18645165] 10.1212/01.wnl.0000318293.28278.33

[CD010224-bib-0117] Kinney MO, Morrow J, Patterson CC, Campbell E, Russell A, Smithson HW, et al. Changing antiepilepsy drug-prescribing trends in women with epilepsy in the UK and Ireland and the impact on major congenital malformations. Journal of Neurology, Neurosurgery, and Psychiatry 2018;89(12):1320-3. [PMID: 29661925] 10.1136/jnnp-2017-317368

[CD010224-bib-0118] Mawhinney E, Campbell J, Craig J, Russell A, Smithson W, Parsons L, et al. Valproate and the risk for congenital malformations: is formulation and dosage regime important? Seizure 2012;21(3):215-8. [PMID: 22364656] 10.1016/j.seizure.2012.01.005

[CD010224-bib-0119] Mawhinney E, Craig J, Morrow J, Russell A, Smithson WH, Parsons L, et al. Levetiracetam in pregnancy: results from the UK and Ireland epilepsy and pregnancy registers. Neurology 2013;80(4):400-5. [PMID: 23303847] 10.1212/WNL.0b013e31827f0874

[CD010224-bib-0120] McCluskey G, Kinney MO, Russell A, Smithson WH, Parsons L, Morrison PJ, et al. Zonisamide safety in pregnancy: data from the UK and Ireland epilepsy and pregnancy register. Seizure 2021;91:311-5. [PMID: 34273670] 10.1016/j.seizure.2021.07.002

[CD010224-bib-0121] Morrow J, Russell A, Guthrie E, Parsons L, Robertson I, Waddell R, et al. Malformation risks of antiepileptic drugs in pregnancy: a prospective study from the UK Epilepsy and Pregnancy Register. Journal of Neurology, Neurosurgery, and Psychiatry 2006;77(2):193-8. [PMID: 16157661] 10.1136/jnnp.2005.074203 PMC2077578

[CD010224-bib-0122] Charlton RA, Weil JG, Cunnington MC, De Vries CS. Identifying major congenital malformations in the UK General Practice Research Database (GPRD): a study reporting on the sensitivity and added value of photocopied medical records and free text in the GPRD. Drug Safety 2010;33(9):741-50. [PMID: 20701407] 10.2165/11536820-000000000-00000

[CD010224-bib-0123] Charlton RA, Weil JG, Cunnington MC, Ray S, De Vries CS. Comparing the General Practice Research Database and the UK Epilepsy and Pregnancy Register as tools for postmarketing teratogen surveillance: anticonvulsants and the risk of major congenital malformations. Drug Safety 2011;34(2):157-71. [PMID: 21247222] 10.2165/11584970-000000000-00000

[CD010224-bib-0124] Ban L, Fleming KM, Doyle P, Smeeth L, Hubbard RB, Fiaschi L, et al. Congenital anomalies in children of mothers taking antiepileptic drugs with and without periconceptional high dose folic acid use: a population-based cohort study. PLOS One 2015;10(7):e0131130. [PMID: 26147467] 10.1371/journal.pone.0131130 PMC4492893

[CD010224-bib-0125] Petersen I, Collings S-L, McCrea RL, Nazareth I, Osborn DP, Cowen PJ, et al. Antiepileptic drugs prescribed in pregnancy and prevalence of major congenital malformations: comparative prevalence studies. Clinical Epidemiology 2017;9:95-103. [PMID: 28243149] 10.2147/CLEP.S118336 PMC5317245

[CD010224-bib-0126] Hernandez-Diaz S, Huybrechts KF, Desai RJ, Cohen JM, Mogun H, Pennell PB, et al. Topiramate use early in pregnancy and the risk of oral clefts: a pregnancy cohort study. Neurology 2018;90(4):e342-51. [PMID: 29282333] 10.1212/WNL.0000000000004857 PMC5798655

[CD010224-bib-0127] Patorno E, Hernandez-Diaz S, Huybrechts KF, Desai RJ, Cohen JM, Mogun H, et al. Gabapentin in pregnancy and the risk of adverse neonatal and maternal outcomes: a population-based cohort study nested in the US Medicaid Analytic eXtract dataset. PLOS Medicine 2020;17(9):e1003322. [PMID: 32870921] 10.1371/journal.pmed.1003322 PMC7462308

[CD010224-bib-0128] Waters CH, Belai Y, Gott PS, Shen P, De Giorgio CM. Outcomes of pregnancy associated with antiepileptic drugs. Archives of Neurology 1994;51(3):250-3. [PMID: 8129635] 10.1001/archneur.1994.00540150044014

[CD010224-bib-0129] Annegers JF, Elveback LR, Hauser WA, Kurland LT. Do anticonvulsants have a teratogenic effect? Archives of Neurology 1974;31(6):364-73. [PMID: 4441320] 10.1001/archneur.1974.00490420030002

[CD010224-bib-0130] Annegers JF, Elveback LR, Hauser WA, Kurland LT. Epilepsy anticonvulsants and malformations. Birth Defects original article series 1975;11(5):157-60. [PMID: 1218208]

[CD010224-bib-0131] Arteaga-Vazquez J, Luna-Munoz L, Mutchinick OM. Congenital malformations in the offspring of epileptic mothers with and without anticonvulsant treatment [Malformaciones congenitas en hijos de madres epilepticas con y sin tratmiento con anticonvulsivantes]. Salud Publica Mexico 2012;54(6):579-86. [PMID: 23318894] 10.1590/s0036-36342012000600006

[CD010224-bib-0132] Arulmozhi T, Dhanaraj M, Rangaraj R, Vengatesan A. Physical growth and psychomotor development of infants exposed to antiepileptic drugs in utero. Neurology India 2006;54(1):42-6; discussion 47. [PMID: 16679642] 10.4103/0028-3886.24701

[CD010224-bib-0133] Bärmig H. Epilepsy and pregnancy [Epilepsie und schwangerschaft]. Geburtshilfe und Frauenheilkunde 1973;33(3):203-4. [PMID: 4692245]

[CD010224-bib-0134] Borthen I, Eide MG, Veiby G, Daltveit AK, Gilhus NE. Complications during pregnancy in women with epilepsy: population-based cohort study. BJOG 2009;116(13):1736-42. [PMID: 19781049] 10.1111/j.1471-0528.2009.02354.x

[CD010224-bib-0135] Bozhinov P, Bozhinova C, Markova C. [Fetal malformations in women with epilepsy]. Akusherstvo i Ginekologiia 2009;48(1):16-21. [PMID: 19496458]

[CD010224-bib-0136] Bozhinova S, Bozhinov P. The course of pregnancy and labor in patients with epilepsy [Protichane na bremennostta i razhdaneto pri bremenni s epilepsiia]. Akusherstvo i Ginekologiia 1998;37(4):12-4. [PMID: 10360042]

[CD010224-bib-0137] Canún-Serrano S, Zafra de la Rosa G, Landeros-Velázquez G, Givaudan-Moreno M. Anticonvulsants and pregnancy [Anticonvulsivos y embarazo]. Boletín Médico del Hospital Infantil de México 1986;43(4):219-27. [PMID: 3707704]

[CD010224-bib-0138] Castilla-Puentes R, Ford L, Manera L, Kwarta RF Jr, Ascher S, Li Q. Topiramate monotherapy use in women with and without epilepsy: pregnancy and neonatal outcomes. Epilepsy Research 2014;108(4):717-24. [PMID: 24598456] 10.1016/j.eplepsyres.2014.01.021

[CD010224-bib-0139] Díaz-Romero RM, Garza-Morales S, Mayén-Molina DG, Ibarra-Puig J, Avila-Rosas H. Facial anthropometric measurements in offspring of epileptic mothers. Archives of Medical Research 1999;30(3):186–9. [PMID: 10427868] 10.1016/s0188-0128(99)00007-x

[CD010224-bib-0140] Dobos M, Schuler D, Marosfi S, Bogáthy B. Congenital developmental anomalies in the offspring of epileptic mothers [Veleszületett fejlödési rendellenességek vizsgálata epilepsziás anyák utódaiban]. Orvosi Hetilap 1985;126(37):2267-72. [PMID: 4047631]

[CD010224-bib-0141] Dravet C, Julian C, Legras C, Magaudda A, Guerrini R, Genton P, et al. Epilepsy, antiepileptic drugs, and malformations in children of women with epilepsy: a French prospective cohort study. Neurology 1992;42(4 Suppl 5):75-82. [PMID: 1574181]

[CD010224-bib-0142] Elshove J, Van Eck JH. Congenital abnormalities, cleft lip and cleft palate in particular, in children of epileptic mothers [Aangeboren misvormingen, met name gespleten lip met of zonder gespleten verhemelte, bij kinderen van moeders met epilepsie]. Nederlands Tijdschrift voor Geneeskunde 1971;115(33):1371-5. [PMID: 5565182]

[CD010224-bib-0143] Thangaratinam S, Marlin N, Newton S, Weckesser A, Bagary M, Greenhill L, et al. AntiEpileptic drug Monitoring in PREgnancy (EMPiRE): a double-blind randomised trial on effectiveness and acceptability of monitoring strategies. Southampton (UK): NIHR Journals Library (Health Technology Assessment, No. 22.23), 2018. [DOI: 10.3310/hta22230] [PMID: 29737274] PMC5960819

[CD010224-bib-0144] Gaily E, Granström ML, Hiilesmaa V, Bardy A. Minor anomalies in offspring of epileptic mothers. Journal of Pediatrics 1988;112(4):520-9. [PMID: 3351676] 10.1016/s0022-3476(88)80162-8

[CD010224-bib-0145] Gaily E. Distal phalangeal hypoplasia in children with prenatal phenytoin exposure: results of a controlled anthropometric study. American Journal of Medical Genetics 1990;35(4):574-8. [PMID: 2333888] 10.1002/ajmg.1320350425

[CD010224-bib-0146] Gaily EK, Granström ML, Hiilesmaa VK, Bardy AH. Head circumference in children of epileptic mothers: contributions of drug exposure and genetic background. Epilepsy Research 1990;5(3):217-22. [PMID: 2384077] 10.1016/0920-1211(90)90041-s

[CD010224-bib-0147] Hiilesmaa VK, Bardy A, Teramo K. Obstetric outcome in women with epilepsy. American Journal of Obstetrics and Gynecology 1985;152(5):499-504. [PMID: 4014343] 10.1016/0002-9378(85)90615-5

[CD010224-bib-0148] Hiilesmaa VK, Teramo K, Granström ML, Bardy AH. Serum folate concentrations during pregnancy in women with epilepsy: relation to antiepileptic drug concentrations, number of seizures, and fetal outcome. British Medical Journal 1983;287(6392):577-9. [PMID: 6411231] 10.1136/bmj.287.6392.577 PMC1549018

[CD010224-bib-0149] Fujii H, Goel A, Bernard N, Pistelli A, Yates LM, Stephens S, et al. Pregnancy outcomes following gabapentin use: results of a prospective comparative cohort study. Neurology 2013;80(17):1565-70. [PMID: 23553472] 10.1212/WNL.0b013e31828f18c1 PMC3662323

[CD010224-bib-0150] Galappatthy P, Liyanage CK, Lucas MN, Jayasekara DTLM, Abhayaratna SA, Weeraratne C, et al. Obstetric outcomes and effects on babies born to women treated for epilepsy during pregnancy in a resource limited setting: a comparative cohort study. BMC Pregnancy & Childbirth 2018;18(1):230. [PMID: 29898689] 10.1186/s12884-018-1857-3 PMC6000926

[CD010224-bib-0151] Goujard J, Huel G, Rumeau-Rouquette C. Antiepileptics and congenital malformations [Antiepileptiques et malformations congenitales]. Journal de Gynécologie, Obstétrique et Biologie de la Reproduction 1974;3(6):831-42. [PMID: 4470757]

[CD010224-bib-0152] Hill RM, Verniaud WM, Horning MG, McCulley LB, Morgan NF. Infants exposed in utero to antiepileptic drugs: a prospective study. American Journal of Diseases of Children 1974;127(5):645-53. [PMID: 4825584] 10.1001/archpedi.1974.02110240031002

[CD010224-bib-0153] Holmes LB, Harvey EA, Brown KS, Hayes AM, Khoshbin S. Anticonvulsant teratogenesis: 1. A study design for newborn infants. Teratology 1994;49(3):202-7. [PMID: 8059427] 10.1002/tera.1420490316

[CD010224-bib-0154] Jacobsen PE, Henriksen TB, Haubek D, Ostergaard JR. Developmental enamel defects in children prenatally exposed to anti-epileptic drugs. PLOS One 2013;8(3):e58213. [PMID: 23520494] 10.1371/journal.pone.0058213 PMC3592922

[CD010224-bib-0155] Jacobsen PE, Henriksen TB, Haubek D, Ostergaard JR. Prenatal exposure to antiepileptic drugs and dental agenesis. PLOS One 2014;9(1):e84420. [PMID: 24416231] 10.1371/journal.pone.0084420 PMC3885552

[CD010224-bib-0156] Jedrzejczak J, Majkowska-Zwolinska B. Clinical predictors for breastfeeding initiation among women with epilepsy. Seizure 2022;96:59-65. [PMID: 35123234] 10.1016/j.seizure.2022.01.013

[CD010224-bib-0157] Jones KL, Lacro RV, Johnson KA, Adams J. Pattern of malformations in the children of women treated with carbamazepine during pregnancy. New England Journal of Medicine 1989;320(25):1661-6. [PMID: 2725616] 10.1056/NEJM198906223202505

[CD010224-bib-0158] Knight AH, Rhind EG. Epilepsy and pregnancy: a study of 153 pregnancies in 59 patients. Epilepsia 1975;16(1):99-110. [PMID: 804404] 10.1111/j.1528-1157.1975.tb04726.x

[CD010224-bib-0159] Cunnington M, Ferber S, Quartey G, International Lamotrigine Pregnancy Registry Scientific Advisory Committee. Effect of dose on the frequency of major birth defects following fetal exposure to lamotrigine monotherapy in an international observational study. Epilepsia 2007;48(6):1207-10. [PMID: 17381445] 10.1111/j.1528-1167.2007.01021.x

[CD010224-bib-0160] Cunnington MC. The International Lamotrigine Pregnancy Registry update for the Epilepsy Foundation. Epilepsia 2004;45(11):1468. [PMID: 15509254] 10.1111/j.0013-9580.2004.451105.x

[CD010224-bib-0161] Lamotrigine Pregnancy Registry. Final report: 1 September 1992–31 March 2010. pregnancyregistry.gsk.com/documents/lam_spring_2010_final_report.pdf 2010 (accessed prior to 4 Feb 2023).

[CD010224-bib-0162] Tennis P, Eldridge RR, International Lamotrigine Pregnancy Registry Scientific Advisory Committee. Preliminary results on pregnancy outcomes in women using lamotrigine. Epilepsia 2002;43(10):1161-7. [PMID: 12366730] 10.1046/j.1528-1157.2002.45901.x

[CD010224-bib-0163] Laskowska M, Leszczyńska-Gorzelak B, Oleszczuk J. Evaluation of antiepileptic therapy during pregnancy [Ocena terapii przeciwpadaczkowej w okresie ciazy]. Ginekologia Polska 2002;73(1):35-42. [PMID: 12001761]

[CD010224-bib-0164] Miskov S, Gjergja-Juraski R, Cvitanovic-Sojat L, Bakulic TI, Fucic A, Bosnjak-Pasic M, et al. Prospective surveillance of Croatian pregnant women on lamotrigine monotherapy - aspects of pre-pregnancy counseling and drug monitoring. Acta Clinica Croatica 2009;48(3):271-81. [PMID: 20055248]

[CD010224-bib-0165] Monson RR, Rosenberg L, Hartz SC, Shapiro S, Heinonen OP, Slone D. Diphenylhydantoin and selected congenital malformations. New England Journal of Medicine 1973;289(20):1049-52. [PMID: 4742220] 10.1056/NEJM197311152892001

[CD010224-bib-0166] Montouris G. Gabapentin exposure in human pregnancy: results from the Gabapentin Pregnancy Registry. Epilepsy & Behavior 2003;4(3):310-7. [PMID: 12791334] 10.1016/s1525-5050(03)00110-0

[CD010224-bib-0167] Mostacci B, Bisulli F, Poluzzi E, Cocchi G, Piccinni C, Curti A, et al. Emilia-Romagna Study on Pregnancy and Exposure to Antiepileptic drugs (ESPEA): a population-based study on prescription patterns, pregnancy outcomes and fetal health. Journal of Neurology, Neurosurgery & Psychiatry 2018;89(9):983-8. [PMID: 29549194] 10.1136/jnnp-2017-317833 PMC6109238

[CD010224-bib-0168] Mostacci B, Piccinni C, Bisulli F, Poluzzi E, Naldi I, Accetta G, et al. Prevalence of antiepileptic drugs exposure in pregnant women in the Emilia Romagna region (Italy): results from the ESPEA (Emilia Romagna Study on Pregnancy and Exposure to Antiepileptic Drugs). Epilepsia 2014;55(Suppl 2):131-2, Abstract no: p400.

[CD010224-bib-0169] Murasaki O, Yoshitake K, Tachiki H, Nakane Y, Kaneko S. Reexamination of the teratological effect of antiepileptic drugs. Japanese Journal of Psychiatry and Neurology 1988;42(3):592-3. [PMID: 3241485] 10.1111/j.1440-1819.1988.tb01370.x

[CD010224-bib-0170] Nakane Y, Okuma T, Takahashi R, Sato Y, Wada T, Sato T, et al. Multi-institutional study on the teratogenicity and fetal toxicity of antiepileptic drugs: a report of a collaborative study group in Japan. Epilepsia 1980;21(6):663-80. [PMID: 7439133] 10.1111/j.1528-1157.1980.tb04320.x

[CD010224-bib-0171] Nakane Y. Congenital malformation among infants of epileptic mothers treated during pregnancy: the report of a collaborative study group in Japan. Folia Psychiatrica et Neurologica Japonica 1979;33(3):363-9. [PMID: 520954] 10.1111/j.1440-1819.1979.tb00771.x

[CD010224-bib-0172] Pearse SB, Garcia Rodriguez LA, Hartwell C, Russell G. A pregnancy register of patients receiving carbamazepine in the UK. Pharmacoepidemiology and Drug Safety 1992;1(6):321-5. [DOI: 10.1002/pds.2630010603]

[CD010224-bib-0173] Richmond JR, Krishnamoorthy P, Andermann E, Benjamin A. Epilepsy and pregnancy: an obstetric perspective. American Journal of Obstetrics and Gynecology 2004;190(2):371-9. [PMID: 14981376] 10.1016/j.ajog.2003.09.020

[CD010224-bib-0174] Robert E, Robert JM, Lapras C. Is valproic acid teratogenic? [L'acide valproïque est-il tératogène?]. Revue Neurologique 1983;139(6-7):445-7. [PMID: 6412342]

[CD010224-bib-0175] Sabers A, Dam M, A-Rogvi-Hansen B, Boas J, Sidenius P, Laue Friis M, et al. Epilepsy and pregnancy: lamotrigine as main drug used. Acta Neurologica Scandinavica 2004;109(1):9-13. 10.1034/j.1600-0404.2003.00200.x 14653845

[CD010224-bib-0176] Scheuerle AE, Holmes LB, Albano JD, Badalamenti V, Battino D, Covington D, et al. Levetiracetam Pregnancy Registry: final results and a review of the impact of registry methodology and definitions on the prevalence of major congenital malformations. Birth Defects Research 2019;111(13):872-87. [PMID: 31124321] 10.1002/bdr2.1526

[CD010224-bib-0177] Shapiro S, Hartz SC, Siskind V, Mitchell AA, Slone D, Rosenberg L, et al. Anticonvulsants and parental epilepsy in the development of birth defects. Lancet 1976;1(7954):272-5. [PMID: 55587] 10.1016/s0140-6736(76)91403-3

[CD010224-bib-0178] Starveld-Zimmerman A, Van der Kolk W, Elshove J, Meinardi H. Teratogenicity of antiepileptic drugs. Clinical Neurology and Neurosurgery 1975;77(2):81-95. [PMID: 1132213] 10.1016/s0303-8467(74)80001-6

[CD010224-bib-0179] Tennis P, Chan KA, Curkendall SM, Li D-K, Mines D, Peterson C, et al. Topiramate use during pregnancy and major congenital malformations in multiple populations. Birth Defects Research 2015;103(4):269-75. [PMID: 25776342] 10.1002/bdra.23357

[CD010224-bib-0180] Torres LC, Félix R, Canún S, Mazón JJ. Epilepsy and pregnancy: risks and benefits of anticonvulsant treatments [Epilepsia y embarazo. Riegos y beneficios del tratamiento anticonvulsivo]. Ginecologia y Obstetricia de Mexico 1995;63:282-6. [PMID: 7665113]

[CD010224-bib-0181] Wide K, Winbladh B, Tomson T, Sars-Zimmer K, Berggren E. Psychomotor development and minor anomalies in children exposed to antiepileptic drugs in utero: a prospective population-based study. Developmental Medicine and Child Neurology 2000;42(2):87-92. [PMID: 10698324] 10.1017/s0012162200000177

[CD010224-bib-0182] Yeh CC, Lussier EC, Sun YT, Lan TY, Yu HY, Chang TY. Antiepileptic drug use among women from the Taiwanese Registry of Epilepsy and Pregnancy: obstetric complications and fetal malformation outcomes. PLOS One 2017;12(12):e0189497. [PMID: 29253023] 10.1371/journal.pone.0189497 PMC5734752

[CD010224-bib-0183] Yerby MS, Leavitt A, Erickson DM, McCormick KB, Loewenson RB, Sells CJ, et al. Antiepileptics and the development of congenital anomalies. Neurology 1992;42(4 Suppl 5):132-40. [PMID: 1574169]

[CD010224-bib-0184] Babic N, Jovic M. Postnatal concerns in children born to women with juvenile myoclonic epilepsy. Epilepsia 2014;55(Suppl 2):128, Abstract no: p389.

[CD010224-bib-0185] Kaabi W, El Aidli S, Kastalli S, Lakhoua G, Zaiem A, Srairi S, et al. Pregnancy outcomes in women using antiepileptic drugs. Drug Safety 2013;36(9):844, Abstract no: ISP3556-47.

[CD010224-bib-0186] Kutlu G, Erdal A, Aydogan S, Gomceli YB, Inan LE. Follow up and treatment of women with epilepsy during pregnancy. Epilepsia 2013;54(Suppl 3):127-8, Abstract no: P399.

[CD010224-bib-0187] Lazzaroni Fossati F, De Toni T, Magnani M, Repetto E, Calvi A, Di Siena G. Intrauterine exposure to drugs: analysis of a sample of newborn infants pretreated with anticonvulsants [Esposizione in utero a farmaci. Analisi di un campione di neonati pretrattati con anticonvulsivanti]. Minerva Pediatrica 1986;38(3-4):75-81. [PMID: 3702846]

[CD010224-bib-0188] Midi I, Bulut B, Ozbek D, Ozden HO, Agan K. Antiepileptic drug usage and the effects of them on the foetus in epileptic pregnant woman. Epilepsia 2014;55(Suppl 2):132, Abstract no: p401.

[CD010224-bib-0189] Midi I, Cetinkaya DO, Ozden HO, Agan K. Pregnant women with epilepsy: 43 patients results in 1 year period. Epilepsia 2013;54(Suppl 3):131, Abstract no: P410.

[CD010224-bib-0190] Shvartzman P, Oren B, Keinan A, Adar H. [Congenital malformations and anticonvulsant therapy in pregnancy]. Harefuah 1986;110(8):377-80. [PMID: 3744149]

[CD010224-bib-0191] Vlasov P, Petrukhin V, Karlov V, Krasnopolski V, Melnikov A, Tsivtsivadze E. Antiepileptic drug therapy during pregnancy and obstetric outcomes in Moscow region: comparing of 1998 and 2013 years. Epilepsia 2014;55(Suppl 2):130, Abstract no: p396.

[CD010224-bib-0192] Ackers R, Besag FM, Wade A, Murray ML, Wong IC. Changing trends in antiepileptic drug prescribing in girls of child-bearing potential. Archives of Disease in Childhood 2009;94(6):443–7. [PMID: 19307197] 10.1136/adc.2008.144386

[CD010224-bib-0193] Alsaad AM, Chaudhry SA, Koren G. First trimester exposure to topiramate and the risk of oral clefts in the offspring: a systematic review and meta-analysis. Reproductive Toxicology 2015;53:45-50. [PMID: 25797654] 10.1016/j.reprotox.2015.03.003

[CD010224-bib-0194] Ardinger HH, Atkin JF, Blackston RD, Elsas LJ, Clarren SK, Livingstone S, et al. Verification of the fetal valproate syndrome phenotype. American Journal of Medical Genetics 1988;29(1):171-85. [PMID: 3125743] 10.1002/ajmg.1320290123

[CD010224-bib-0195] Brent RL. Environmental causes of human congenital malformations: the pediatrician's role in dealing with these complex clinical problems caused by a multiplicity of environmental and genetic factors. Pediatrics 2004;113(4 Suppl):957-68. [PMID: 15060188]

[CD010224-bib-0196] Bromley R, Weston J, Adab N, Greenhalgh J, Sanniti A, McKay AJ, et al. Treatment for epilepsy in pregnancy: neurodevelopmental outcomes in the child. Cochrane Database of Systematic Reviews 2014, Issue 10. Art. No: CD010236. [DOI: 10.1002/14651858.CD010236.pub2] PMC7390020 25354543

[CD010224-bib-0197] Charlton RA, Cunnington MC, De Vries CS, Weil JG. Data resources for investigating drug exposure during pregnancy and associated outcomes: the General Practice Research Database (GPRD) as an alternative to pregnancy registries. Drug Safety 2008;31(1):39-51. [PMID: 18095745] 10.2165/00002018-200831010-00004

[CD010224-bib-0198] Chaudhry SA, Jong G, Koren G. The fetal safety of levetiracetam: a systematic review. Reproductive Toxicology 2014;46:40-5. [PMID: 24602560] 10.1016/j.reprotox.2014.02.004

[CD010224-bib-0199] Christensen J, Grønborg TK, Sørensen MJ, Schendel D, Parner ET, Pedersen LH, et al. Prenatal valproate exposure and risk of autism spectrum disorders and childhood autism. JAMA 2013;309(16):1696-703. [PMID: 23613074] 10.1001/jama.2013.2270 PMC4511955

[CD010224-bib-0200] Christensen J, Pedersen L, Sun Y, Dreier JW, Brikell I, Dalsgaard S. Association of prenatal exposure to valproate and other antiepileptic drugs with risk for attention-deficit/hyperactivity disorder in offspring. JAMA Network Open 2019;2(1):e186606. [PMID: 30646190] 10.1001/jamanetworkopen.2018.6606 PMC6324310

[CD010224-bib-0201] Clayton-Smith J, Bromley R, Dean J, Journel H, Odent S, Wood A, et al. Diagnosis and management of individuals with Fetal Valproate Spectrum Disorder; a consensus statement from the European Reference Network for Congenital Malformations and Intellectual Disability. Orphanet Journal of Rare Diseases 2019;14(1):180. [PMID: 31324220] 10.1186/s13023-019-1064-y PMC6642533

[CD010224-bib-0202] Dean JC, Moore SJ, Turnpenny PD. Developing diagnostic criteria for the fetal anticonvulsant syndromes. Seizure 2000;9(3):233–4. [PMID: 10775521] 10.1053/seiz.2000.0392

[CD010224-bib-0203] DiLiberti JH, Farndon PA, Dennis NR, Curry CJ. The fetal valproate syndrome. American Journal of Medical Genetics 1984;19(3):473-81. [PMID: 6439041] 10.1002/ajmg.1320190308

[CD010224-bib-0204] EUROCAT European Surveillance of Congenital Anomalies. EUROCAT guide 1.3 and reference documents: instructions for the registration and surveillance of congenital anomalies. eu-rd-platform.jrc.ec.europa.eu/sites/default/files/EUROCAT-Guide-1.3.pdf (accessed August 2015).

[CD010224-bib-0205] Fiest KM, Sauro KM, Wiebe S, Patten SB, Kwon CS, Dykeman J, et al. Prevalence and incidence of epilepsy: a systematic review and meta-analysis of international studies. Neurology 2017;88(3):296-303. [PMID: 27986877] 10.1212/WNL.0000000000003509 PMC5272794

[CD010224-bib-0206] Guyatt GH, Oxman AD, Vist G, Kunz R, Falck-Ytter Y, Alonso-Coello P, et al. GRADE: an emerging consensus on rating quality of evidence and strength of recommendations. BMJ 2008;336(7650):924-6. [PMID: 18436948] 10.1136/bmj.39489.470347.AD PMC2335261

[CD010224-bib-0207] Higgins JPT, Green S (editors). *Cochrane Handbook for Systematic Reviews of Interventions* Version 5.1.0 [updated March 2011]. The Cochrane Collaboration, 2011. Available from training.cochrane.org/handbook/archive/v5.1/.

[CD010224-bib-0208] Jentink J, Loane MA, Dolk H, Barisic I, Garne E, Morris JK, et al. Valproic acid monotherapy in pregnancy and major congenital malformations. New England Journal of Medicine 2010;362(23):2185-93. [PMID: 20558369] 10.1056/NEJMoa0907328

[CD010224-bib-0209] Jentink J, Dolk H, Loane MA, Morris JK, Wellesley D, Garne E, et al. Intrauterine exposure to carbamazepine and specific congenital malformations: systematic review and case-control study. BMJ 2010;341:c6581. [PMID: 21127116] 10.1136/bmj.c6581 PMC2996546

[CD010224-bib-0210] Ku CS, Naidoo N, Wu M, Soong R. Studying the epigenome using next generation sequencing. Journal of Medical Genetics 2011;48(11):721-30. [PMID: 21825079] 10.1136/jmedgenet-2011-100242

[CD010224-bib-0211] Man SL, Petersen I, Thompson M, Nazareth I. Antiepileptic drugs during pregnancy in primary care: a UK population based study. PLOS One 2012;7:e52339. [PMID: 23272239] 10.1371/journal.pone.0052339 PMC3525559

[CD010224-bib-0212] Margulis AV, Mitchell AA, Gilboa SM, Werler MM, Mittleman MA, Glynn RJ, et al, National Birth Defects Prevention Study. Use of topiramate in pregnancy and risk of oral clefts. American Journal of Obstetrics and Gynecology 2012;207(5):405.e1-7. [PMID: 22917484] 10.1016/j.ajog.2012.07.008 PMC3484193

[CD010224-bib-0213] Marson AG, Al-Kharusi AM, Alwaidh M, Appleton R, Baker GA, Chadwick DW, et al, SANAD Study group. The SANAD study of effectiveness of valproate, lamotrigine, or topiramate for generalised and unclassifiable epilepsy: an unblinded randomised controlled trial. Lancet 2007;369(9566):1016-26. [PMID: 17382828] 10.1016/S0140-6736(07)60461-9 PMC2039891

[CD010224-bib-0214] Meador K, Reynolds MW, Crean S, Fahrbach K, Probst C. Pregnancy outcomes in women with epilepsy: a systematic review and meta-analysis of published pregnancy registries and cohorts. Epilepsy Research 2008;81(1):1-13. [PMID: 18565732] 10.1016/j.eplepsyres.2008.04.022 PMC2660205

[CD010224-bib-0215] Meador KJ, Penovich P, Baker GA, Pennell PB, Bromfield E, Pack A, et al, NEAD Study Group. Antiepileptic drug use in women of childbearing age. Epilepsy and Behaviour 2009;15(3):339-43. [PMID: 19410654] 10.1016/j.yebeh.2009.04.026 PMC2741411

[CD010224-bib-0216] Mines D, Tennis P, Curkendall SM, Li DK, Peterson C, Andrews EB, et al. Topiramate use in pregnancy and the birth prevalence of oral clefts. Pharmacoepidemiology and Drug Safety 2014;23(10):1017–25. [PMID: 24692316] 10.1002/pds.3612

[CD010224-bib-0217] National Institute for Clinical Excellence (NICE). Epilepsies in children, young people and adults. NICE guideline [NG217]. www.nice.org.uk/guidance/ng217 (accessed prior to 4 Feb 2023).

[CD010224-bib-0218] Oommen KJ, Mathews S. Zonisamide: a new antiepileptic drug. Clinical Neuropharmacology 1999;22(4):192-200. [PMID: 10442247]

[CD010224-bib-0219] Sterne JA, Hernán MA, Reeves BC, Savović J, Berkman ND, Viswanathan M, et al. ROBINS-I: a tool for assessing risk of bias in non-randomised studies of interventions. BMJ 2016;355:i4919. [PMID: 27733354] 10.1136/bmj.i4919 PMC5062054

[CD010224-bib-0220] Tetro N, Moushaev S, Shmuel M, Eyal S. Antiseizure medications and fetal nutrients: effects on choline transporters in a human placental cell line. Epilepsia 2021;62(6):1451-9. [PMID: 33890297] 10.1111/epi.16905

[CD010224-bib-0221] Tomson T, Battino D, Bonizzoni E, Craig J, Lindhout D, Sabers A, et al, EURAP study group. Dose-dependent risk of malformations with antiepileptic drugs: an analysis of data from the EURAP epilepsy and pregnancy registry. Lancet Neurology 2011;10(7):609-17. [PMID: 21652013] 10.1016/S1474-4422(11)70107-7

[CD010224-bib-0222] Tomson T, Marson A, Boon P, Canevini MP, Covanis A, Gaily E, et al. Valproate in the treatment of epilepsy in girls and women of childbearing potential. Epilepsia 2015;56(7):1006-19. [PMID: 25851171] 10.1111/epi.13021

[CD010224-bib-0223] Veroniki AA, Cogo E, Rios P, Straus SE, Finkelstein Y, Kealey R, et al. Comparative safety of anti-epileptic drugs during pregnancy: a systematic review and network meta-analysis of congenital malformations and prenatal outcomes. BMC Medicine 2017;15(1):95. [PMID: 28472982] 10.1186/s12916-017-0845-1 PMC5418725

[CD010224-bib-0224] Wen X, Meador KJ, Hartzema A. Antiepileptic drug use by pregnant women enrolled in Florida Medicaid. Neurology 2015;84(9):944-50. [PMID: 25653296] 10.1212/WNL.0000000000001304 PMC4351665

[CD010224-bib-0225] Adab N, Tudur SC, Vinten J, Williamson P, Winterbottom J. Common antiepileptic drugs in pregnancy in women with epilepsy. Cochrane Database of Systematic Reviews 2004, Issue 3. Art. No: CD004848. [DOI: 10.1002/14651858.CD004848] 15266543

[CD010224-bib-0226] Pulman J, Bromley R, Adab N, Greenhalgh J, McKay AJ, Tudur Smith C, et al. Treatment for epilepsy in pregnancy: congenital malformation outcomes in the child. Cochrane Database of Systematic Reviews 2012, Issue 11. Art. No: CD010224. [DOI: 10.1002/14651858.CD010224] PMC6465055 27819746

[CD010224-bib-0227] Weston J, Bromley R, Jackson CF, Adab N, Clayton-Smith J, Greenhalgh J, et al. Monotherapy treatment of epilepsy in pregnancy: congenital malformation outcomes in the child. Cochrane Database of Systematic Reviews 2016, Issue 11. Art. No: CD010224. [DOI: 10.1002/14651858.CD010224.pub2] PMC6465055 27819746

